# Immobilization of Trifluoromethyl-Substituted Pyridine-Oxazoline Ligand and Its Application in Asymmetric Continuous Flow Synthesis of Benzosultams

**DOI:** 10.1021/acs.joc.3c01671

**Published:** 2023-10-12

**Authors:** Martin Kocúrik, Jan Bartáček, Pavel Drabina, Jiří Váňa, Jan Svoboda, Lenka Husáková, Vladimír Finger, Michaela Hympánová, Miloš Sedlák

**Affiliations:** † Institute of Organic Chemistry and Technology, Faculty of Chemical Technology, University of Pardubice, Studentská 573, Pardubice, CZ 532 10, Czech Republic; ‡ Department of Analytical Chemistry, Faculty of Chemical Technology, University of Pardubice, Studentská 573, Pardubice, CZ 532 10, Czech Republic; § Faculty of Pharmacy in Hradec Králové, Charles University, Akademika Heyrovského 1203, 50005, Hradec Králové, CZ 500 05, Czech Republic; ∥ Biomedical Research Center, University Hospital Hradec Králové, Sokolská 581, Hradec Králové, CZ 500 05, Czech Republic; ⊥ Faculty of Military Health Sciences, University of Defence, Trebešská 1575, Hradec Králové, CZ 500 01, Czech Republic

## Abstract

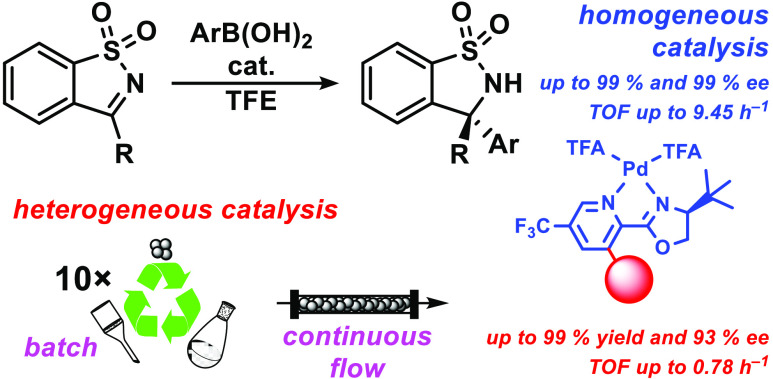

This study presents an improved synthetic route to ligand (*S*)-4-(*tert*-butyl)-2-(5-(trifluoromethyl)pyridin-2-yl)-4,5-dihydrooxazole and its application as a highly active and enantioselective catalyst in the addition of arylboronic acids to cyclic *N*-sulfonylketimines. Immobilization of such a ligand was achieved using a commercially available starting material and a PS–PEG TentaGel S NH_2_ support, resulting in a stable heterogeneous catalyst. Although the anchored catalyst exhibited a slight reduction in enantioselectivity and a 4-fold decrease in reaction rate, it displayed remarkable stability, enabling 10 consecutive reaction cycles. Furthermore, the successful transition to a continuous flow system demonstrated even higher turnover numbers compared to batch arrangements. These findings provide valuable insights into the development of efficient flow reactors for continuous synthesis of benzosultams, further advancing the field of asymmetric catalysis.

## Introduction

**Figure 1:**
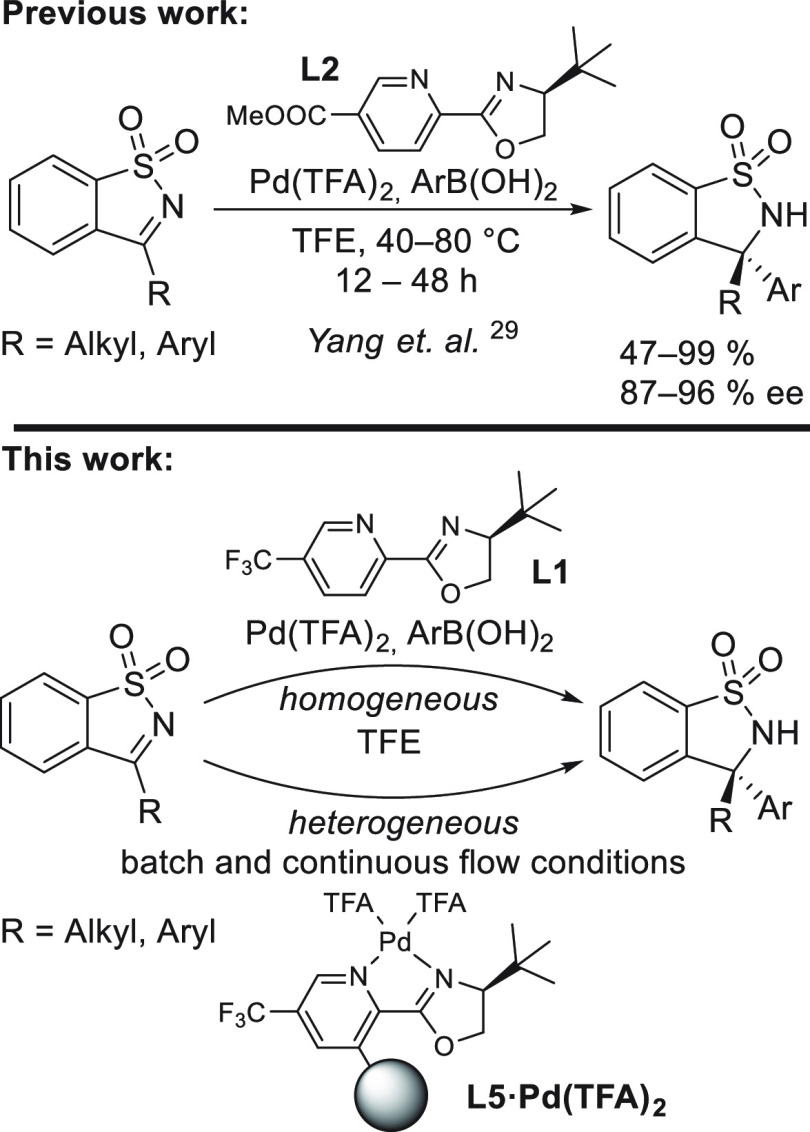
Aim of the work and previous work.

Pyridine-oxazoline (PyOx) type ligands are widely recognized for their privileged role in asymmetric catalysis by transition metal complexes. Their extensive applicability has been extensively reviewed.^1,2^ Electronic properties play a crucial role in their effectiveness, and among the electron-poor ligands, those featuring trifluoromethyl (**L1**) or methoxycarbonyl (**L2**) groups at the 5-position of the pyridine ring are frequently employed (Figure 1). The first advantage of **L1** over **L2** lies in its convenient synthesis from readily available precursors, making it attractive for large-scale syntheses. Pd complexes of **L1** have demonstrated utility in various reactions including conjugated addition of boronic acids to enones,^3^ redox-relay Heck reactions,^4−9^ Heck–Matsuda arylation,^10,11^ arylboration,^12^ and several Heck-related cascade reactions.^13,14^ Additionally, the Cu(I) complex of **L1** has been employed as a catalytic system for conjugate addition to isocyanoalkenes,^15^ Ni(I) complex for hydroalkynylation,^16^ and Ni(0) complex for asymmetric reductive coupling.^17^ In addition, analogous ligands featuring different substitutions at the oxazoline moiety and maintaining the trifluoromethyl group at the 5-position of the pyridine ring have been employed in various catalytic transformations.^18−27^

**Scheme 1:**
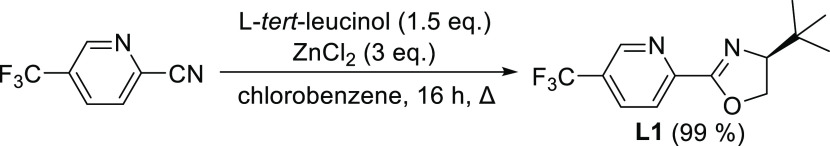
Optimized Synthesis of **L1**

On the other hand, the synthesis of ligand **L2** is more laborious and provides lower yields.^28^ Nevertheless, it has been found to be a valuable ligand in the preparation of optically enriched cyclic sulfonamides containing a bis-benzylic quaternary stereogenic center through the addition of arylboronic acids to saccharin-derived cyclic ketimines^29^ (Scheme 1). The addition of boronic acids to cyclic *N*-sulfonylketimines has been extensively studied under Pd catalytic conditions,^29−36^ as well as with other transition metals such as Rh, Ni, or Co.^37^ The resulting products, sulfonamides bearing a quaternary stereogenic center, have been utilized for the synthesis of biologically active tertiary amines,^38,39^ sulfinyl hydroxylamines,^40^ or subjected to further transformations such as heterocyclic ring expansion.^41^ Moreover, these compounds, known as benzosultams, exhibit interesting biological properties^42^ and can serve as valuable chiral auxiliaries in organic synthesis.^43^ Hence, this work aims to investigate the possibility of using the more readily accessible ligand **L1** for the synthesis of this intriguing class of compounds.

Considering the potential of benzosultams as pharmaceutically relevant compounds and their possible synthesis on a large scale, it is worth exploring their synthesis using continuous flow reactors. Flow reactors necessitate catalyst immobilization and the development of synthetic protocols under heterogeneous conditions.

**Figure 2:**
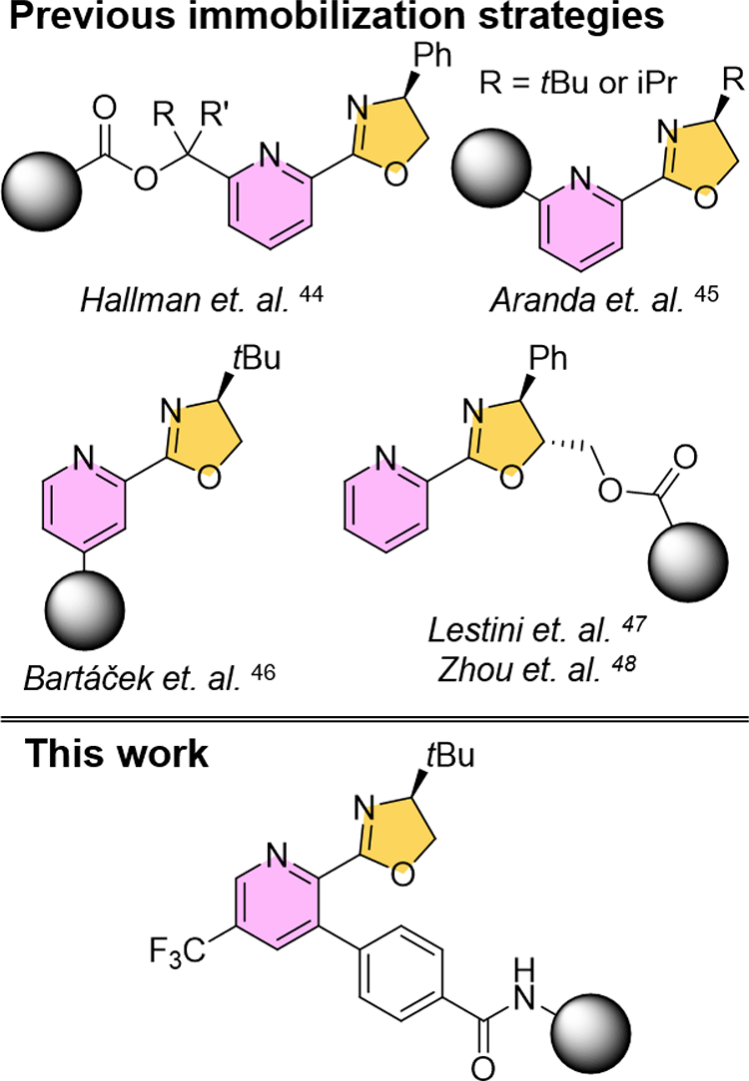
Immobilization strategies for PyOx-type ligands.^44−48^

Previous studies have successfully reported immobilization strategies for various pyridine-oxazoline type ligands^44−48^ (Figure 2). However, most investigations have primarily focused on immobilizing the unsubstituted pyridine-oxazoline skeleton. The 6-position on the pyridine ring has been employed as an anchoring site for reactions that require a bulky PyOx ligand, such as allylic substitution^44^ and cyclopropanation.^45^ Alternatively, the 4-position has been utilized for the conjugated addition of arylboronic acids to cyclic enones.^46^ Another approach involves anchoring via the 5-position of the oxazoline ring through an ester bond, enabling the preparation of micellar nanoreactors for the conjugated addition of arylboronic acids to chromones.^47,48^

Based on the findings of these prior works, we have designed an immobilization strategy for the available **L1** ligand and aim to test it under heterogeneous batch and continuous flow conditions, which constitutes the second objective of this study.

## Results and Discussion

### Homogeneous Catalysis

Initially, we focused on optimizing the synthesis of ligand **L1**. Previous literature^49^ reported a 71% yield for the condensation reaction of 5-(trifluoromethyl)picolinonitrile with L-*tert*-Leucinol using 20 mol % Zn(OTf)_2_ catalyst. However, when this condensation was performed under 300 mol % of ZnCl_2_ catalysis, ligand **L1** was obtained in nearly quantitative yield (Scheme 1).

To assess the effectiveness of ligand **L1** in comparison to the previously published **L2**, we conducted reactions using various arylboronic acids with 3-butylbenzo[*d*]isothiazole 1,1-dioxide (S1) under conditions similar to the original protocol,^29^ with a reaction time of 12 h. If complete conversion was not achieved within 12 h, we extended the reaction time to 24 h. For reactions that achieved complete conversion within 12 h, we monitored the reaction at shorter intervals to determine the exact time required for complete conversion.

**Figure 3:**
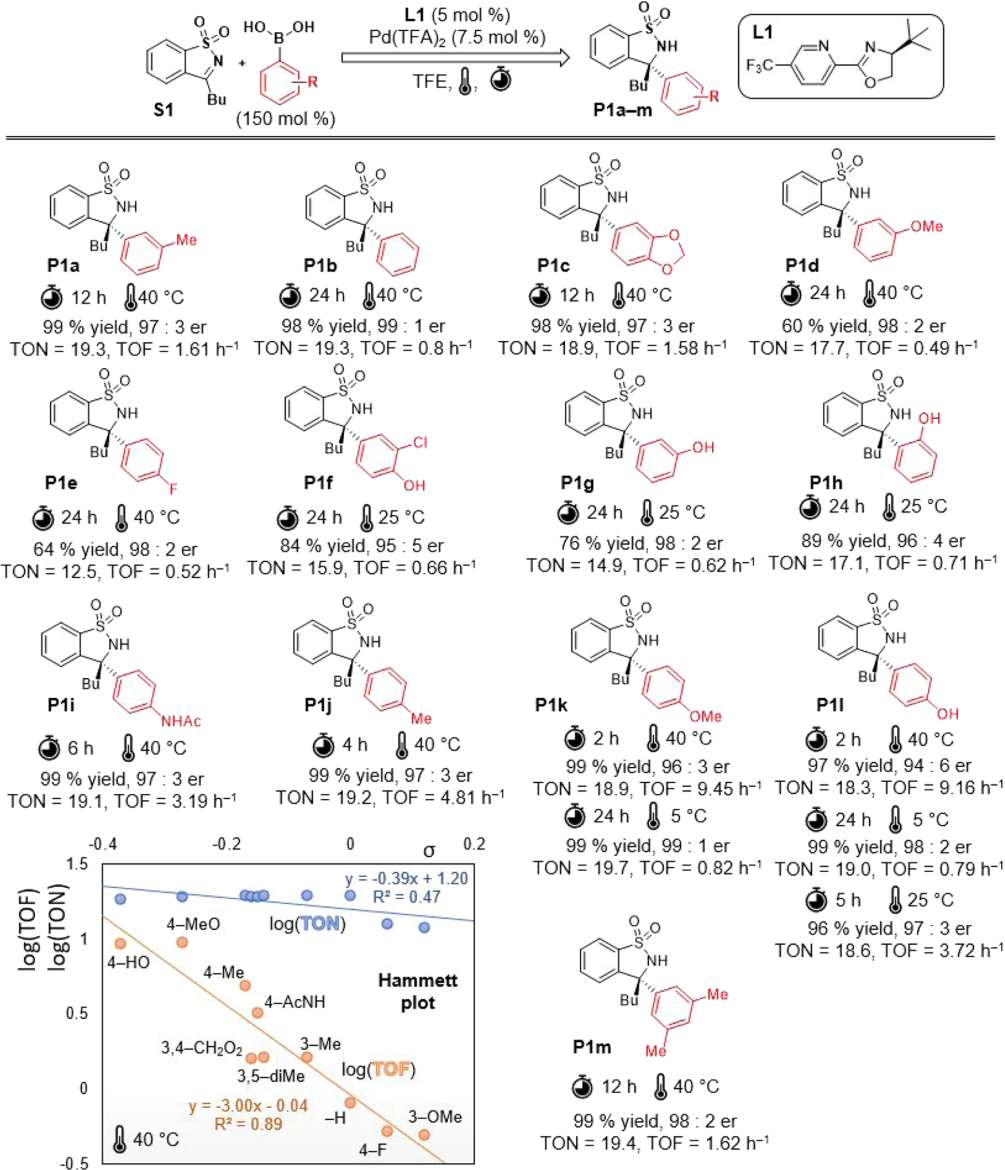
Results of homogeneous catalysis using ligand **L1**.

**Figure 4:**
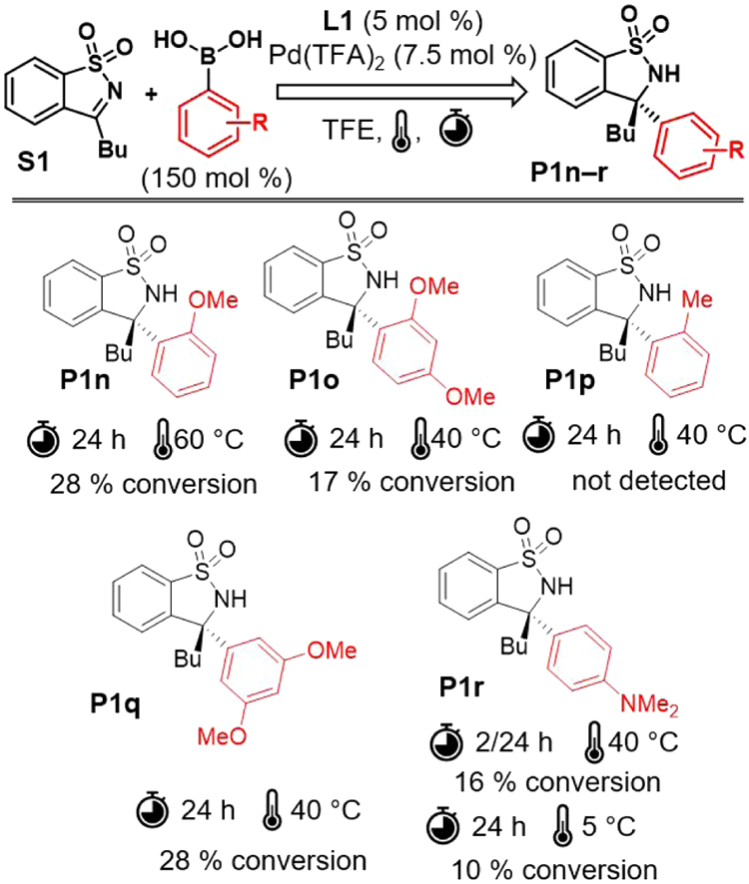
Less successful and unsuccessful results of catalysis using **L1**.

The results (Figure 3) indicated that the presence of electron-donating groups on arylboronic acids in the meta- and para-positions accelerated the reaction. The highest turnover frequency (TOF) was observed when 4-hydroxyphenylboronic acid was used (Figure 3; **P1l**). However, when we further increased the electron-donating character using 4-(dimethylamino)phenylboronic acid, we observed only minimal conversion (<14%) and the major product was the protodeboronation product *N*,*N*-dimethylaniline within the temperature range of 5–40 °C (Figure 4; **P1r**). The reactivity was significantly reduced for boronic acids with electron-withdrawing substituents such as 3-methoxyphenylboronic acid (Figure 3; **P 1d**) and 4-fluorophenylboronic acid (Figure 3; **P1e**), and the reaction with 3,5-dimethoxyphenylboronic acid was even more sluggish (Figure 4; **P1q**). *Ortho*-substituted boronic acids presented the greatest challenge as the reactivity was fundamentally limited by *ortho* substitution with a methyl or methoxy group, and this limitation could not be compensated even with an electron-donor at position 4- (Figure 4; **P1n**, **P1o**, **P 1p**).

Interestingly, the product of addition of 2-hydroxyphenylboronic acid was isolated in unexpectedly high yield and enantioselectivity (Figure 3; **P1h**). We assume that this is due to possible hydrogen bond formation, which is not possible in the case of methyl or methoxy substitution. However, DFT calculations based on known energy profiles^34^ suggest that the transition state of the rate-determining step does not prefer the formation of hydrogen bonds (for more details, see S3).

The negative ρ-value and the overall reactivity trend (Figure 3) suggested that stabilizing the arylpalladium cation with electron donors was advantageous in the rate-limiting step. These findings were consistent with the proposed mechanism, which postulated that migratory insertion is both the rate-determining and enantioselectivity-determining step.^34^

**Figure 5:**
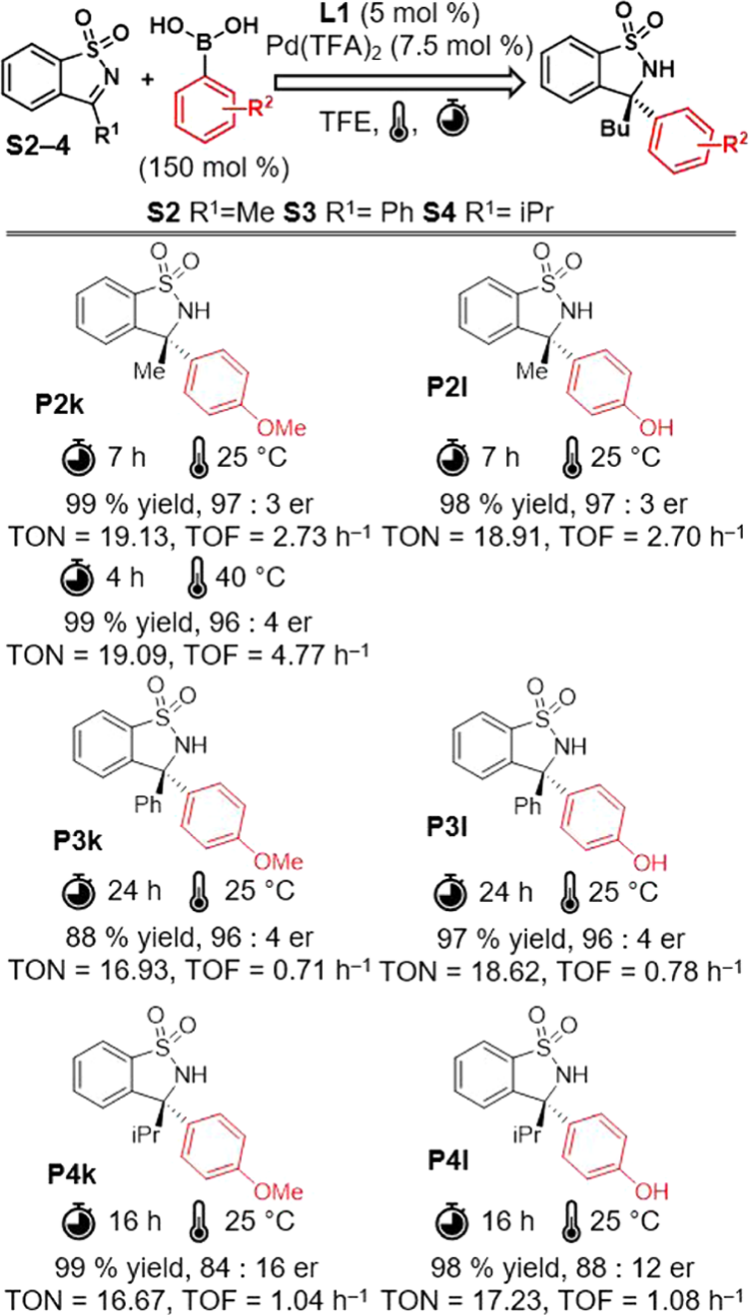
Substrate scope of variously substituted cyclic ketimines.

Furthermore, we expanded the substrate scope to include variously substituted ketimines (S2–4) in addition reactions with 4-hydroxyphenylboronic and 4-methoxyphenylboronic acids (Figure 5). High reactivity and enantioselectivity were observed except for the substrate bearing an isopropyl group (S4).

### Immobilization Strategy

**Scheme 2:**
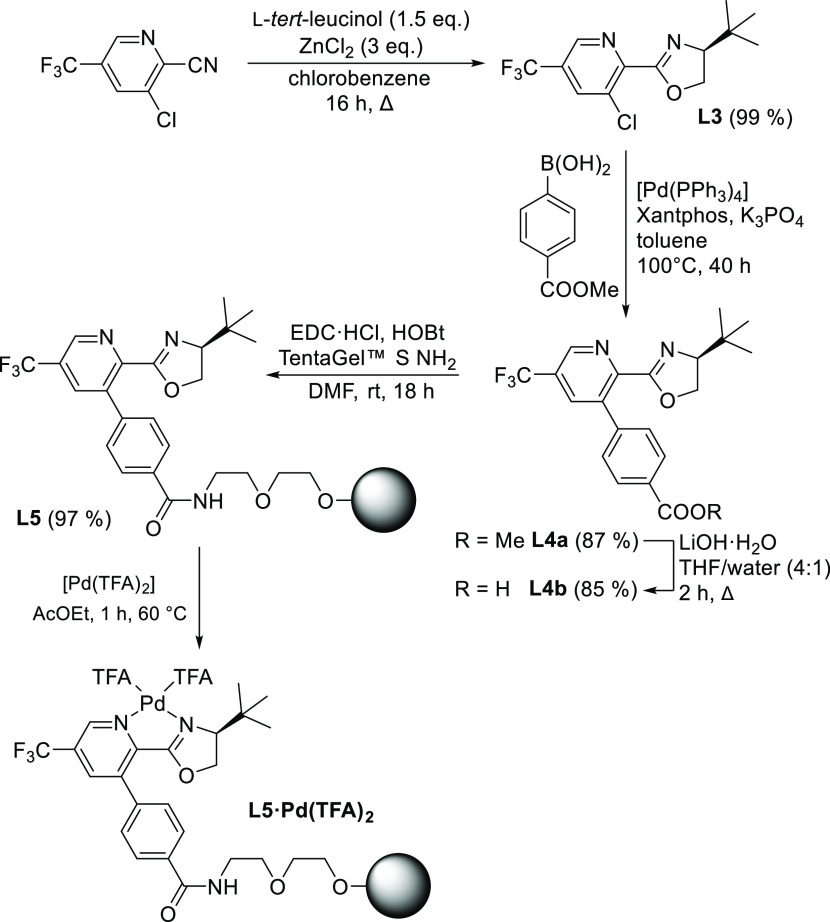
Synthetic Pathway to Polymer Supported Pyridine-Oxazoline Ligand **L5** and Its Complex **L5·Pd(TFA)**_**2**_

Immobilization strategy is based on commercially available 3-chloro-5-(trifluoromethyl)picolinonitrile, which is a precursor for the fungicide fluopicolide.^50^ By condensing it with L-*tert*-leucinol, we obtain **L3** bearing a reactive chlorine atom, which was then substituted by a 4-methoxycarbonylphenyl group (**L3a**) through a Suzuki-Miyaura cross-coupling reaction (Scheme 2). Subsequently, **L4a** was hydrolyzed to yield free carboxylic acid (**L4b**). The actual anchoring onto the polymeric carrier was accomplished through amidation between **L4b** and commercially available PS–PEG resin (TentaGel S NH_2_), which is generally suitable as a carrier for reactions in a highly polar environment.^51^

From the prepared polymer-supported ligand **L5**, its complex with palladium(II) trifluoroacetate **L5·Pd(TFA)**_**2**_ was prepared (Scheme 2). Ethyl acetate was the solvent of choice due to the limited solubility of Pd(TFA)_2_ in other organic solvents.

**Figure 6:**
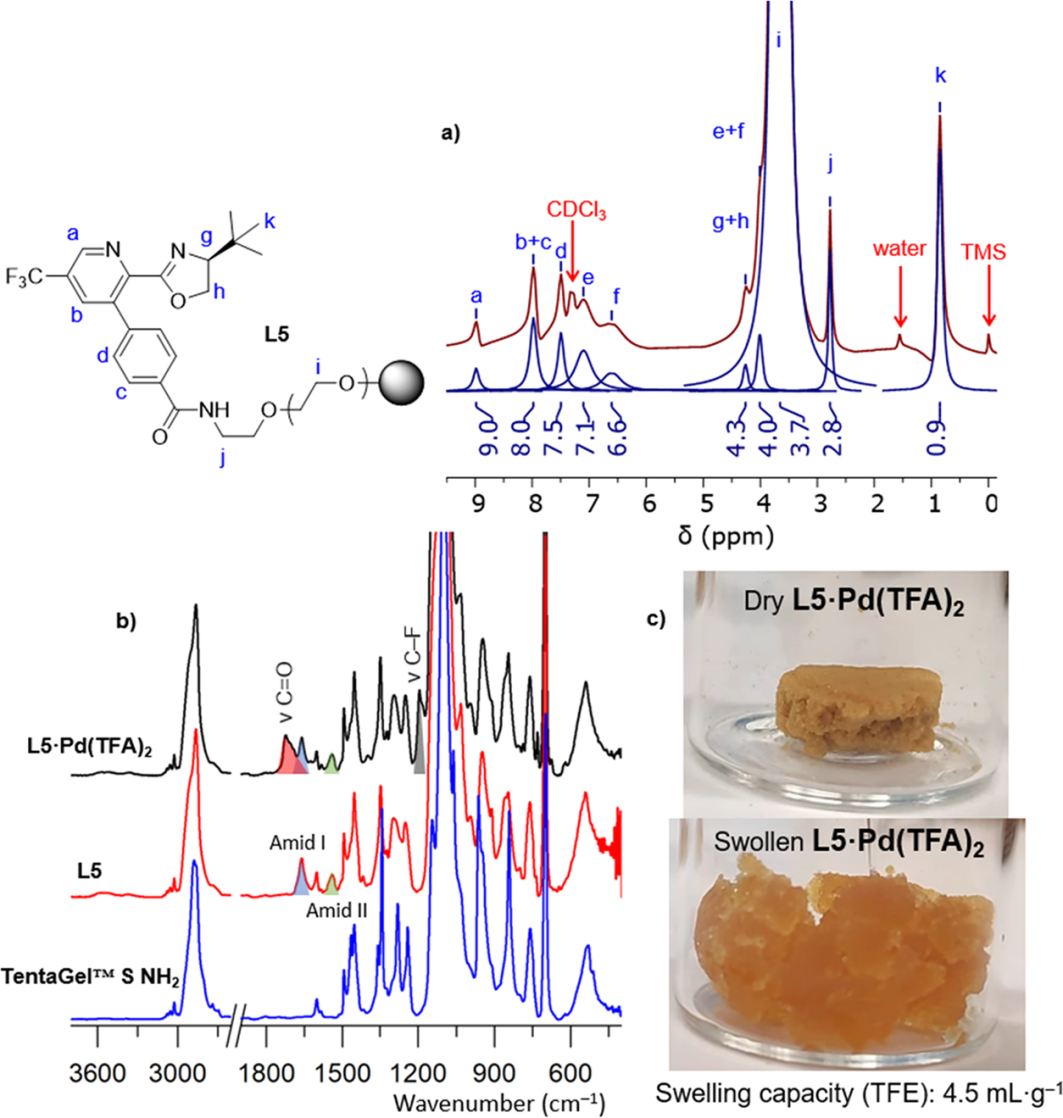
(a) ^1^H gel-phase NMR of **L5** (b) FT-IR spectra of commercial resin, **L5** and its complex **L5·Pd(TFA)**_**2**_ (c) photography of dry and swollen **L5·Pd(TFA)**_**2**_.

The prepared **L5** was characterized by using FT-IR spectroscopy, microanalysis, and gel-phase ^1^H and ^13^C NMR. The ligand’s anchoring is observable in the IR spectra due to the appearance of new amide vibration bands (Figure 6b). The TentaGel resin’s swelling properties (Figure 6c) allowed for gel-phase NMR measurements, further supporting the structure of **L5** (Figure 6a). After complexation reaction with Pd(TFA)_2_, the prepared **L5·Pd(TFA)**_**2**_ was further characterized using FT-IR spectroscopy (Figure 6b). A change in the infrared spectra is particularly noticeable in the signals corresponding to both the C=O and C–F vibrations (Figure 6b). The exact Pd content was determined through ICP-MS analysis.

### Testing the Catalytic Performance of L5·Pd(TFA)_2_ in Batch Arrangement

**Figure 7:**
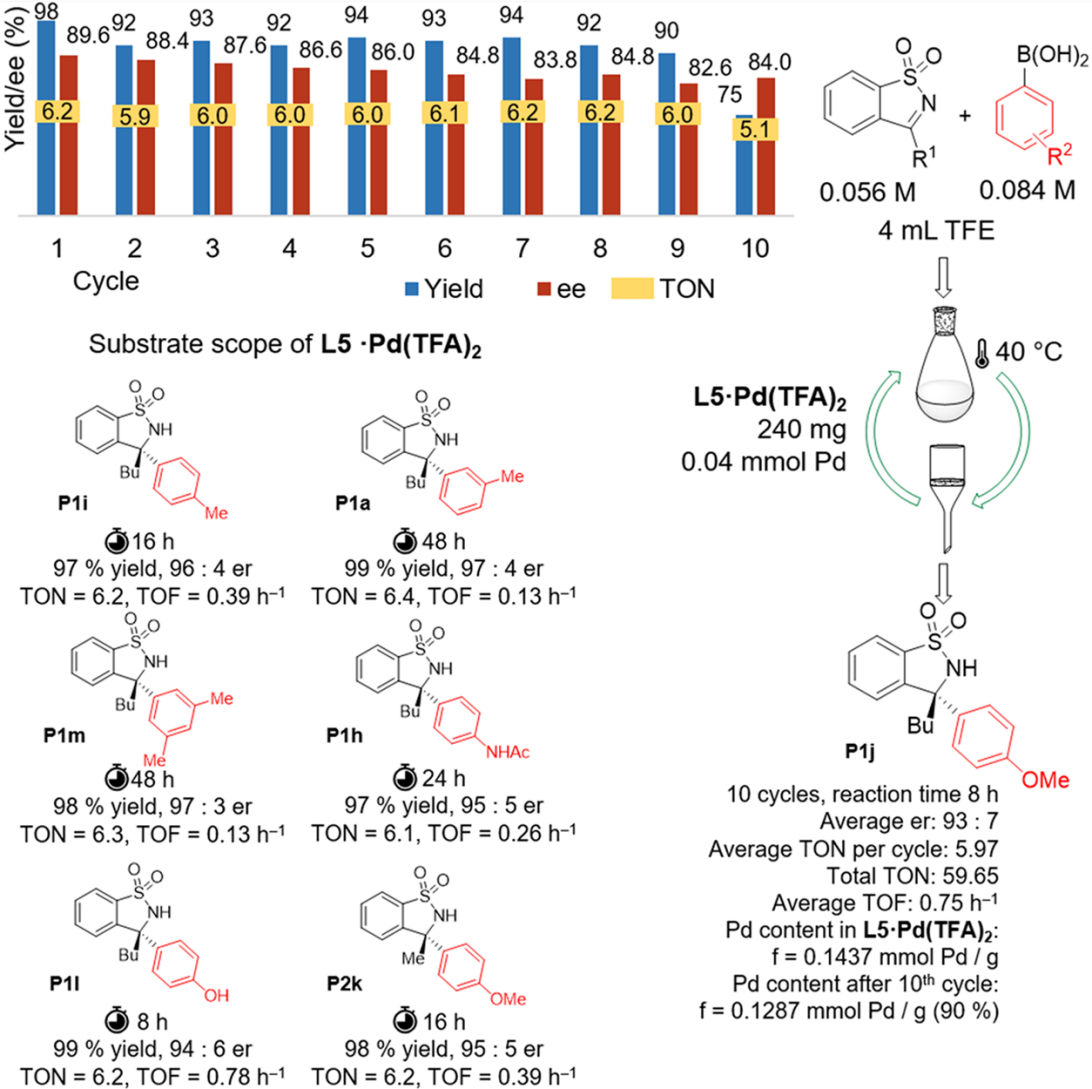
Results of catalytic experiments with **L5·Pd(TFA)**_**2**_ in batch arrangement.

After successfully synthesizing the polymer-supported complex **L5·Pd(TFA)**_**2**_, its effectiveness for addition reaction of 4-methoxyphenylboronic acid to ketimine **S1** was studied with a focus on its reusability in repeated reaction cycles (Figure 7).

The results revealed that immobilization led to a reduction in the catalytic reaction rate, necessitating a 4-fold increase in reaction time (from 2 to 8 h) and an increase in the loading of the catalyst from 5 to 15 mol % to achieve yields comparable to those obtained with homogeneously catalyzed reactions (Figure 7). At the same time, a slight decrease in the level of observed enantioselectivity was evident. After each reaction cycle, the catalyst was filtered, washed, and used again. It was possible to use the catalyst up to 10 times; however, a slight deactivation of the catalyst was observed with continued recycling cycles. This can be explained by Pd leaching, where the Pd content decreased by 10% during the 10 uses compared with the Pd content of the freshly prepared **L5·Pd(TFA)**_**2**_ complex.

Thus, the efficiency of the prepared **L5·Pd(TFA)**_**2**_ on the model reaction can be expressed as TON ∼ 59 and TOF 0.75 h^–1^, while TON ∼ 19 and TOF = 9.45 h^–1^ were obtained for catalysis in an analogous homogeneous system.

A study of the catalytic properties of **L5·Pd(TFA)**_**2**_ on a series of substrates was also carried out. While the trend of reactivity was generally the same as in the homogeneous environment, a 4-fold increase in reaction time and 3-fold increase in catalyst loading was required to achieve a conversion comparable to the homogeneous catalysis conditions. The results of Hammett correlation analysis are analogous to a reaction catalyzed in a homogeneous environment (see S4).

### Testing the Catalytic Performance of L5·Pd(TFA)_2_ in Continuous Flow Arrangement

After optimizing the conditions for performing the reaction under batch conditions, we focused on the assessment of the catalytic performance of **L5·Pd(TFA)**_**2**_ in a continuous flow arrangement. To simulate the continuous flow system, we prepared a reactor column by pressurizing swollen **L5·Pd(TFA)**_**2**_. Mechanical pressing using a piston and dosing pump was used to pressurize the column. Prior to use, the column was saturated in solvent to ensure optimum polymer swelling.

Operating at higher pressures compared with the batch experiments was essential in establishing tight contact between the polymer and the reactor tube wall. It prevented the desorption of dissolved oxygen in the reaction mixture, ensuring a consistent catalytic performance. Maintaining a steady pressure in the column was crucial to preventing oxygen desorption and local drying of the polymer, which could hinder catalytic reactions. Thus, saturating the mixture of starting materials with pressure before entering the pump was necessary to facilitate oxygen dissolution and the pump-filling process.

To avoid desorption of oxygen in the pump and subsequent flow stoppage, it was important to ensure the pressure saturation of the mixture in the reservoir. The flow rate of the reaction mixture through the reactor was optimized based on desired conversion and pressure. Increasing the flow rate resulted in higher pressure, facilitating diffusion of reactants into the swelling polymer. This pressure-induced diffusion advantage provided a notable improvement over that of the batch system.

**Figure 8:**
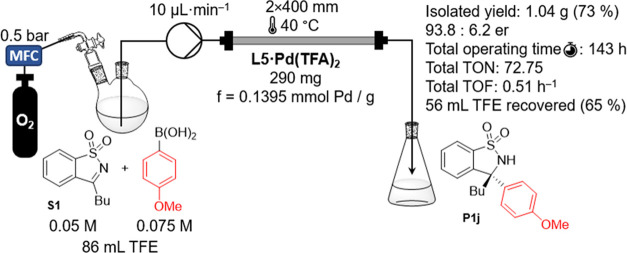
Schematic view and result summarization of continuous flow synthesis of **P1j**.

Flow system parameters were optimized to achieve 95+ % conversion of **S1** to **P1j** (see S5 for more details). The optimized conditions obtained were used to conduct the synthesis of **P1j** under continuous flow conditions (Figure 8). During the 143 h experimental period, the prepared **L5·Pd(TFA)**_**2**_ exhibited an efficiency of TON ∼ 73 and TOF 0.51 h^–1^. Comparing batch and continuous flow conditions, we observed higher catalyst efficiency under flow conditions (Table 1).

**Table 1:**
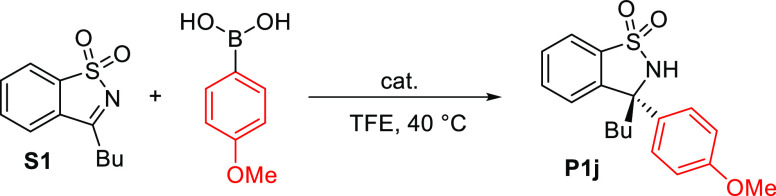
Comparison of Homogeneous and Heterogeneous Catalysis Efficiency

	**catalyst**		
	**L1** + Pd(TFA)_2_	**L5·Pd(TFA)**_**2**_ (batch)	**L5·Pd(TFA)**_**2**_ (continuous flow)
e.r.	95.5:4.5	92.9:7.1	93.8:6.2
ee	91	85.8	87.6
TON (−)	18.90	56.56	72.75
TOF (h^–1^)	9.45	0.71	0.51

However, there was a slight decrease in the reaction rate (TOF) in the continuous flow system. Notably, the continuous flow setup allowed for a 65% reduction in the usage of TFE.

## Conclusions

In conclusion, this work has presented an improved synthetic route to the ligand (*S*)-4-(*tert*-butyl)-2-(5-(trifluoromethyl)pyridin-2-yl)-4,5-dihydrooxazole (**L1**) and its successful utilization as a catalyst in the addition of arylboronic acids to cyclic *N*-sulfonylketimines. The ligand **L1**, in the form of a complex with palladium(II) trifluoroacetate, demonstrated high catalytic activity and enantioselectivity, showcasing its potential as a valuable tool in asymmetric synthesis.

Moreover, an immobilization strategy for ligand **L1** was developed using a commercially available starting material and a PS–PEG TentaGel S NH_2_ type support, leading to the preparation of a heterogeneous catalyst. Although a 4-fold slowing of the reaction rate and a slight reduction in enantioselectivity were observed after anchoring, the immobilized catalyst exhibited remarkable stability, allowing for 10 consecutive reaction cycles. Furthermore, the successful transfer of the reaction to a continuous flow arrangement proved to be highly advantageous, as the immobilized catalyst achieved an even higher turnover number compared with the batch system.

Notably, the flow-through system exhibited an overall longer lifetime of the catalyzing polymer, potentially attributed to its gradual deactivation along the flow direction. In contrast to a batch system where uniform deactivation occurs, the polymer in the flow column experienced greater inactivation at the entrance, while remaining partially active toward the end. Consequently, the turnover frequency (TOF) should be considered as an average value that continuously varies throughout the column.

Overall, this study not only presents a novel synthetic route and an immobilization strategy for ligand **L1** but also demonstrates the efficacy of the catalyst in both batch and continuous flow systems. The findings provide valuable insights into the development of efficient flow reactors for the continuous synthesis of enantioenriched compounds, such as benzosultams, and pave the way for further advancements in the field of asymmetric catalysis.

## Data Availability

The data underlying this study are available in the published article and its Supporting Information.

## Supporting Information Available

Experimental details, computational details, HPLC traces, NMR and FT-IR spectra of all the compounds (PDF)
