# Supplementary material for: Immobilization of Trifluoromethyl-Substituted Pyridine-Oxazoline Ligand and Its Application in Asymmetric Continuous Flow Synthesis of Benzosultams
Source: J Org Chem. 2023 Oct 12;88(21):15189–97. doi: 10.1021/acs.joc.3c01671 (PMC10629231; doi:10.1021/acs.joc.3c01671)
Supplement: Supplementary file 1 — jo3c01671_si_001.pdf [file jo3c01671_si_001.pdf]

Supporting Information

for

Immobilization of trifluoromethyl substituted pyridine-oxazoline ligand and its application in asymmetric continuous flow synthesis of benzosultams

Martin Kocúrik,[a] Jan Bartáček,*[a] Pavel Drabina,[a] Jiří Váňa,[a] Jan Svoboda,[a] Lenka Husáková,[b] Vladimír Finger,[c,d] Michaela Hympánová,[d,e] and Miloš Sedlák[a]

[a]*Institute of Organic Chemistry and Technology, Faculty of Chemical Technology, University of Pardubice, Studentská 573, Pardubice CZ 532 10, Czech Republic. e-mail* jan.bartacek@upce.cz

[b]*Department of Analytical Chemistry, Faculty of Chemical Technology, University of Pardubice, Studentská 573, Pardubice CZ 532 10, Czech Republic.*

[c] *Faculty of Pharmacy in Hradec Králové, Charles University, Akademika Heyrovského 1203, 50005 Hradec Králové CZ 500 05, Czech Republic*

[d] *Biomedical Research Center, University Hospital Hradec Králové, Sokolská 581, Hradec Králové CZ 500 05, Czech Republic*

[e] *Faculty of Military Health Sciences, University of Defence, Trebešská 1575, Hradec Králové CZ 500 01, Czech Republic*

Table of Contents

S1-Experimental details, compounds characterization 3
General 3
Instrumentation 3
Determination of Pd content in heterogeneous catalysts 3
Sample preparation for ICP-MS analysis 4
Catalyst efficiency evaluation 5
Continuous flow reaction setup 5
S2-Synthesis 6
Preparation of ligands 6
Preparation of substrates 9
General procedure for palladium-catalyzed enantioselective additions of arylboronic acids to cyclic ketimines in homogeneous conditions 11
General procedure for palladium-catalyzed enantioselective additions of arylboronic acids to cyclic ketimines in heterogeneous conditions 11
S3-DFT calculations 22
S4-Hammett plot for catalysis with L5·Pd(TFA)$_2$ 30
S5-Optimization of continuous flow synthesis of P1j 31
S6-NMR, FT-IR spectra 32
S7-HPLC traces 69
References 117

S1-Experimental details, compounds characterization

General

All chemicals were purchased from commercial resources and used without further purification unless otherwise mentioned. TentaGel™ S NH_2 (loading value of NH_2 0.2-0.3 mmol/g) was purchased from ThermoFisher Scientific. Thin layer chromatography was performed on aluminium plates coated with silica gel SiO_2 with visualization by UV light (254 or 366 nm) or by *p*-anisaldehyde-sulfuric acid visualization reagent. Preparative chromatography was performed on Reveleris® X2 on C18 columns. Determination of melting points were placed in open capillaries on Buchi B-540 and were not corrected.

Instrumentation

The FT-IR spectra were recorded on FT-IR Nicolet iS50 using the ATR technique. The region of diamond crystal absorption (1900–2400 cm^{-1}) was removed from spectra.

NMR spectra were measured at 24°C on Bruker AVANCE III or Bruker Ascend™ 500. ^{1}H NMR spectra were calibrated to tetramethylsilane. ^{13}C NMR spectra were calibrated to the middle signal of the multiplet of used solvent. ^{13}C NMR spectra were measured with proton decoupling or with APT technique. $19^F\{^1H\}$ NMR spectra were not calibrated.

HRMS were measured by the „dried droplet "method using the MALDI mass spectrometer LTQ Orbitrap XL (Thermo Fisher Scientific, Bremen, Germany) equipped with nitrogen UV laser (337 nm, 60 Hz). The spectra were taken in the positive ion mode with the resolution 100,000 at m/z=400. The resulting spectrum represents an average of all measurements. 2,5-dihydroxybenzoic acid (DHB) was used as a matrix.

HPLC analysis were performed on HPLC instrument with UV–Vis diode array (200–800 nm) SYKAM 3240 and with chiral columns Daicel Chiralcel OJ-H, OD-H or Chiralpak AD-H, OD-H, or IA.

The swelling capacities of the prepared copolymer were determined in accordance with the literature. [1]

Analysis on scanning electron microscope (SEM) were measured on JEOL JSM-LV. The accelerating voltage of the primary electron beam was set to 15kV in the low vacuum mode (11 Pa).

Determination of Pd content in heterogeneous catalysts

Pd concentration data was collected using an Agilent 7900 ICP-MS system (Agilent Technologies, Inc., Santa Clara, CA, USA). The instrument was equipped with quadrupole analyzer, the standard nickel interface cones, glass concentric nebulizer MicroMist (400 µL min-1), the Peltier-cooled quartz spray chamber, 2.5-mm internal diameter quartz torch, low-pulsation, 10-roller peristaltic pump with three separate channels for precise delivery of samples and standards, and octopole-based collision/reaction cell (ORS4), optimized for effective and reliable removal of potential multiple polyatomic interferences using kinetic energy discrimination (KED) in helium (He) collision mode. For Pd quantification, the system provides detection limits at ppt levels and accurate results in complex and variable sample matrices (see Ref. [2]). The instrument was optimized using the autotuning function of the ICP-MS

MassHunter software. The isotopes used for the quantification of Pd with the instrument operating parameters are shown in Table S1.

Table S1 Agilent 7900 ICP-MS operating conditions.

Parameter	Setting	
ICP		
Plasma mode	General purpose	
Rf power (27 MHz) (W)	1550	
Sampling depth (mm)	10	
Plasma gas flow ($L\cdot min^{-1}$)	15	
Auxiliary gas flow ($L\cdot min^{-1}$)	0.9	
Nebulizer gas flow ($L\cdot min^{-1}$)	1.05	
Nebulizer pump (rps)	0.1	
Spray chamber temperature (° C)	2	
Mass spectrometer	No gas mode	He mode
Extract 1 (V)	0	0
Extract 2 (V)	-205	-200
Omega bias (V)	-75	-85
Omega lens (V)	11.3	10.7
Cell entrance	-30	-40
Cell exit	-50	-60
Deflect (V)	13.8	1.6
Plate bias	-35	-60
Helium flow ($mL\cdot min^{-1}$)	0	5
OctP bias	-8	-18
OctP RF	200	
Energy discrimination (V)	5	5
Isotopes (integration time, s)	^{105}Pd, ^{108}Pd, ^{103}Rh (0.1)	
Acquisition		
Points per peak	1	
Replicates	3	
Sweeps/replicate	100	

Analyte concentrations were determined with external calibration ranging from blank to 100 $\mu g\cdot L^{-1}$ of Pd. Calibration standards were premixed daily from 500 $mg\cdot L^{-1}$ of Pd stock solution prepared by an appropriate dilution of the SCP Science standard (1 $g\cdot L^{-1}$) using 1% HNO_3 to ensure the stability of the analyte. Linear calibrations were obtained with coefficients of determination >0.999 to compensate for possible instrumental drift and matrix effects, a 200 $\mu g\cdot L^{-1}$ Rh internal standard element (SCP Science, Canada) was simultaneously aspired and mixed with samples.

Sample preparation for ICP-MS analysis

Samples were digested in a Speedwave XPERT closed microwave oven system (Berghof, Eningen, Germany) with a power output of dual magnetrons of 2 × 1,000 W and optical sensors for contactless real-time recording of the sample temperature and pressure in each vessel. The high-pressure resistant

(up to 100 bar) TFM™ -PTFE vessels DAK100 were used for sample digestion. The 3 to 10 mg sample aliquots were accurately weighed in PTFE vessels, then 6 mL of 65% HNO3 was added. The vessels were closed, and the following microwave oven heating program was performed: (i) 5 min at 170 °C and 60 % power (ramp 5 min), (ii) 25 min at 220 °C and 70 % power (ramp 5 min), (iii) 5 min at 100 °C and 10 % power (ramp 1 min). After digestion, the solutions were cooled to room temperature and diluted to 25 mL with deionized water. The digests were diluted 100 × with deionized water prior to analysis by ICP-MS. Each sample was prepared in three replicates. Blanks consisting of deionized water and reagents were subjected to a similar preparation procedure.

Catalyst efficiency evaluation

The turnover number (TON) was expressed for the production of the major stereoisomer and calculated according to the following equation:

$$TON = \frac{\frac{yield\ (\%) \cdot major(\%)}{100}}{mol\ \%\ cat.}$$

Turnover frequency (TOF) was then expressed according to the formula:

$$TOF = \frac{TON}{time}$$

Continuous flow reaction setup

Figure 9 in the manuscript shows the flow system's experimental arrangement. A Schlenk flask with a stock solution containing substrate and boronic acid was saturated with oxygen and maintained under an overpressure of 0.5 bar. The stock solution was dosed by positive pressure into the syringe pump through the three-way valve. The stock solution from the syringe pump was dosed into the reactor at a defined rate. The inlet overpressure depended on the degree of compression of the heterogeneous gel containing the catalyst. The inlet pressure was continuously monitored, and the system was set to shut down when the limit pressure was exceeded automatically. The output from the reactor was introduced into a storage flask under atmospheric pressure. Here, the absorbed oxygen was spontaneously desorbed. The Asia syringe pump was used as the dosing pump. The maximum overpressure was set to 10 bars. The reactor was tested for overpressure of 15 bars. The reactor consisted of a 400 mm long Teflon capillary with an inner diameter of 2 mm and was tempered in a water bath to the required temperature. The reactor packing column has been created by compressing the catalytically active swelled solid polymer. Pressing was done at first mechanically by a piston and then by dosing pump by pumping the solvent.

S2-Synthesis

Preparation of ligands

General procedure for synthesis of oxazoline cycle

$ZnCl_2$ (3.96 g, 0.029 mol, 3 eq.) was melted in Schlenk flask under vacuum by heatgun and cooled down under N_2 atmosphere. Solution of corresponding nitrile (0.00968 mol, 1 eq.) and *L-tert*-leucinol (1.7 g, 0.0145 mol, 1.5 eq.) in chlorobenzene (20 mL) was degassed for 15 min, and then transferred to the Schlenk flask with $ZnCl_2$. Resulting mixture was refluxed for 24 h under atmosphere of N_2 on the DrySyn heating block. After cooling, mixture was transferred to the separatory funnel with DCM (15 mL) and with 10 % solution of NaOH (100 mL). Organic phase was separated, and aqueous phase was extracted with DCM (4 × 15 mL), combined organic phase were washed with distilled water (20 mL), brine (20 mL), then dried over Na_2SO_4 and evaporated do dryness.

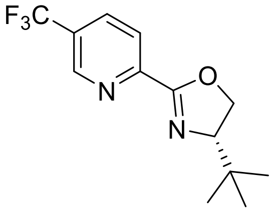

(*S*)-4-(*tert*-butyl)-2-(5-(trifluoromethyl)pyridin-2-yl)-4,5-dihydrooxazole (L1)

white solid, m.p. 106-107 °C, 2.62 g, 99 %, characterization data in accordance with ref. [3].

^{1}H NMR (400 MHz, $CDCl_3$) δ 8.93 (s, 1H), 8.19 (d, *J*=8.35 Hz, 1H), 7.99 (dd, *J*=8.52, 1.83 Hz, 1H), 4.46 (dd, *J*=10.62, 9.14 Hz, 1H), 4.32 (t, *J*=8.53 Hz, 1H), 4.13 (dd, *J*=10.52, 8.90 Hz, 1H), 0.95 (s, 9H).

^{13}C{^{1}H} NMR (101 MHz, $CDCl_3$) δ 161. 6, 150.1, 146.6 (q, *J*=4.12 Hz), 133.9 (q, *J*=3.62 Hz), 128.1 (q, 32.26 Hz), 123.3 (q, *J*=272.45 Hz), 76.8, 69.7, 34.1, 26.0.

^{19}F{^{1}H} NMR (376 MHz, $CDCl_3$) δ -62.60.

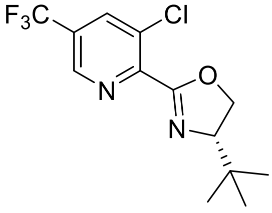

(*S*)-4-(*tert*-butyl)-2-(3-chloro-5-(trifluoromethyl)pyridin-2-yl)-4,5-dihydrooxazole (L3)

Yellow oil, 2.95g, 99 %

^{1}H NMR (400 MHz, $CDCl_3$) δ 8.77 (s, 1H), 8.00 (s, 1H), 4.41 (dd, *J*=10.15, 8.64 Hz, 1H), 4.27 (t, *J*=8.47 Hz, 1H), 4.17 (dd, *J*=10.19, 8.50 Hz, 1H), 0.95 (s, 9H).

$^{13}C\{^1H\}$ NMR (101 MHz, $CDCl_3$) δ 159.5, 148.8, 144.0 (q, *J*=3.97 Hz), 135.7 (q, *J*=3.66 Hz), 132.2. 128.4 (q, *J*=33.87 Hz), 122.2 (q, *J*=273.30 Hz), 77.3, 69.3, 33.9, 25.9.

$^{19}F\{^1H\}$ NMR (376 MHz, $CDCl_3$) δ -63.56.

HRMS calcd for $[C_{13}H_{14}ClF_3N_2O+H]^+$ 307.0825, found $[C_{13}H_{14}ClF_3N_2O+H]^+$ 307.0827; Δ = 0.94 ppm

FT-IR (ATR) cm^{-1}: 2957, 2906, 2871, 1679, 1479, 1394, 1319, 1137, 1047, 912.

Preparation of methyl (*S*)-4-(2-(4-(*tert*-butyl)-4,5-dihydrooxazol-2-yl)-5-(trifluoromethyl)pyridin-3-yl)benzoate (L4a)

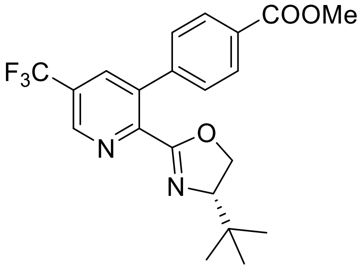

Schlenk flask was charged with **L3** (1.5 g, 4.89 mmol, 1 eq.), 4-(Methoxycarbonyl)benzeneboronic acid (1.32 g, 7.34 mmol, 1.5 eq.), K_3PO_4 (2.1 g, 9.87 mmol, 2 eq.),), $Pd(PPh_3)_4$ (0,282 g; 0.244 mmol, 5 mol %) and with Xantphos (0.169 g; 0.293 mmol, 6 mol %). Flask was evacuated and refiled with N_2 (3 times), then dry toluene (30 mL) was added and mixture was heated at 105 °C for 36 h under N_2 on a oil bath. After cooling, reaction mixture was filtered through plug of celite, eluted with EtOAc (40 mL) and evaporated. Raw mixture was purified by column chromatography with mobile phase EtOAc:Hexane (1:4+ 5 % TEA).

white solid, m.p. 123-124 °C, 1.73 g, 87 %

^1H NMR (500 MHz, $CDCl_3$) δ 8.96 (d, *J*=1.35 Hz, 1H), 8.11-8.09 (m, 2H), 7.95 (d, *J*=1.54 Hz, 1H), 7.48-7.46 (m, 2H), 4.28-4.23 (m, 1H), 4.02-3.99 (m, 2H), 3.96 (s, 3H), 0.82 (s, 9H).

$^{13}C\{^1H\}$ NMR (126 MHz, $CDCl_3$) δ 166.7, 161.5, 149.3, 145.5 (q, *J*=3.93 Hz), 141.9, 137.4, 135.4 (q, *J*=3.43 Hz), 130.3, 129.7, 128.9, 127.6 (q, *J*=33.54 Hz), 123.1 (q, *J*=272.29 Hz), 69.5, 52.5, 33.9, 26.1.

$^{19}F\{^1H\}$ NMR (376 MHz, $CDCl_3$) δ -62.47.

HRMS calcd for $[C_{21}H_{21}F_3N_2O_3+H]^+$ 407.1577, found $[C_{21}H_{21}F_3N_2O_3+H]^+$ 407.1580; Δ = 0.82 ppm.

FT-IR (ATR) cm^{-1}: 2958, 2904, 2870, 1726, 1679, 1419, 1346, 1275, 1108, 1032, 951, 775, 705.

Preparation of (S)-4-(2-(4-(tert-butyl)-4,5-dihydrooxazol-2-yl)-5-(trifluoromethyl)pyridin-3-yl)benzoic acid (L4b)

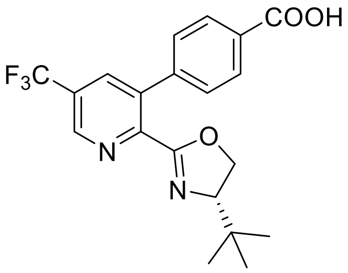

100 mL flask was charged with **L4a** (0.5 g, 1.23 mmol, 1 eq.), LiOH×H_2O (0.129 g, 3.08 mmol, 2.5 eq.) and with THF:water (4:1, 60 mL). Mixture was refluxed for 2 h on a oil bath, then it was cooled and transferred to a separatory funnel and extracted with Et_2O (5 mL). Aqueous phase was carefully acidified to pH=4 with 1 M HCl and immediately extracted with DCM (3 × 6 mL). Combined DCM phase were washed with brine, dried over Na_2SO_4 and evaporated.

white solid, m.p. 219-220 °C, 0.41 g, 85 %

^{1}H NMR (500 MHz, DMSO) δ 13.12 (s, 1H), 9.09 (d, *J*=1.39 Hz, 1H), 8.36 (d, *J*=1.78 Hz, 1H), 8.01-8.00 (m, 2H), 7.59-7.58 (m, 2H), 4.26 (dd, *J*=10.12, 8.93 Hz, 1H), 4.05 (t, *J*=8.95 Hz, 1H), 3.89 (dd, *J*=10.12, 9.26 Hz, 1H), 0.72 (s, 9H).

^{13}C{^{1}H} NMR (126 MHz, DMSO) δ 167.0, 160.5, 149.6, 145.3 (q, *J*=3.97 Hz), 140.8, 136.5, 135.7 (q, *J*=3.42 Hz), 130.7, 129.3, 129.0, 126.2 (q, *J*=32.84 Hz), 123.2 (q, *J*=273.52 Hz), 76.0, 68.7, 33.4, 25.6.

^{19}F{^{1}H} NMR (376 MHz, $CDCl_3$) δ -60.91.

HRMS calcd for $[C_{20}H_{19}F_3N_2O_3+H]^+$ 393.1420, found $[C_{20}H_{19}F_3N_2O_3+H]^+$ 393.1428; Δ = 1.95 ppm.

FT-IR (ATR) cm^{-1}: 2966, 2871, 1699, 1660, 1607, 1415, 1340, 1265, 1110, 858, 775, 560.

Preparation of PS-PEG-CF_3PyOx (L5)

25 mL flask was charged with TentaGel™ S NH_2 (1.38 g, 0.3567 mmol, 0.7 eq.), **L4b** (0.2 g, 0.5097 mmol, 1 eq.), EDC×HCl (0.29 mg, 1.529 mmol, 3 eq.), HOBt×H_2O (0.21 g, 1.529 mmol, 3 eq.), DMAP (0.018 g, 0.1529 mmol, 0.3 eq.), DMF (10 mL) and mixture was stirred at rt for 24 h. Resulting **L5** was collected by filtration, washed with DMF (2×5 mL), MeOH (2×10 mL), DCM (3×10 mL) and dried under vacuum (1.502 g, 97 %, Swelling capacity(TFE): 4.5 ml·g^{-1})

Gel phase ^{1}H NMR (500 MHz, $CDCl_3$) δ 9.00, 8.00, 7.52, 7.36, 7.12, 6.63, 4.35, 4.02, 3.67, 2.80 (s, 2H), 0.87 (s, 9H).

Gel phase ^{13}C{^{1}H} NMR (126 MHz, $CDCl_3$) δ 166.7, 161.6, 149.3, 145.3, 140.1, 137.3, 135.3, 134.6, 128.7, 127.5, 127.4, 123.0 (q, *J*=273.42 Hz), 76.9, 70.6, 69.8, 69.5, 40.4, 39.9, 33.8, 26.0.

FT-IR (ATR) cm^{-1}: 2864, 1660, 1452, 1347, 1097, 846, 699, 540, 415.

Preparation of Pd·PS-PEG-CF_3PyOx (L5·Pd(TFA)$_2$)

25 mL flask was charged with **L5** (0.503 g, 0.1284 mmol, 1.5 eq.), Pd(TFA)$_2$ (0.0285 g, 0.0856 mmol, 1 eq.), EtOAc (10 mL) and mixture was heated to 60 °C for 1 h on a sand bath. Resulting **L5·Pd(TFA)$_2$** was collected by filtration, washed with EtOAc (2×10 mL), MeOH (2×10 mL), DCM (3×10 mL) and dried under vacuum (Pd content 0.1437 mmol/g, 88 %)

FT-IR (ATR) cm^{-1}: 2864, 1723, 1452, 1348, 1096, 846, 699, 539.

Preparation of substrates

Preparation of 3-butylbenzo[*d*]isothiazole 1,1-dioxide (S1)

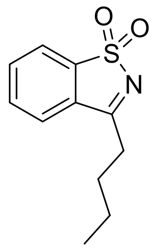

Saccharin (6 g, 32.75 mmol, 1 eq.) was added to Schlenk flask, followed by addition of dry THF (250 mL) and cooled to -78 °C. Solution of *n*-BuLi in hexane (26 mL, 2.5 M, 2 eq.) was added over period of 1 h. Reaction mixture was cooled to -78 for 4 h and the slowly heated to rt overnight. Then solid NH_4Cl was added followed by addition of water (80 mL). Mixture was transferred to the separation funnel, aqueous phase was separated and extracted with DCM (3×50 mL). Combined organic phase was washed with 10% HCl (2×100 mL), saturated $NaHCO_3$ (2×100 mL), brine then dried over Na_2SO_4 and evaporated. Crude product was recrystalized from EtOH.

m.p. 94-95 °C , white solid, 5.56 g, 76 %, characterization data in accordance with ref. [4].

^{1}H NMR (500 MHz, $CDCl_3$) δ 7.91-7.87 (m, 1H), 7.76-7.69 (m, 3H), 2.96 (t, *J*=7.40 Hz, 2H), 1.86 (p, *J*=7.69 Hz, 2H), 1.49 (sextet, *J*=7.54 Hz, 2H), 0.97 (t, *J*=7.34 Hz, 3H).

^{13}C{^{1}H} NMR (126 MHz, $CDCl_3$) δ 176.5, 139.7, 134.0, 133.6, 131.3, 124.0, 122.4, 30.9, 27.5, 22.4, 13.8.

Preparation of 3-methylbenzo[*d*]isothiazole 1,1-dioxide (S2)

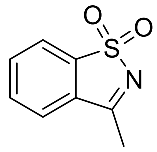

Saccharin (2 g, 10.92 mmol, 1 eq.) was added to Schlenk flask, followed by addition of dry THF (120 mL) and cooled to –78 °C. Solution of MeLi in Et_2O (13.6 mL, 1.6 M, 2 eq.) was added over period of 30 min. Reaction mixture was cooled to -78 for 4 h and the slowly heated to rt overnight. Then water

(50 mL) was added, mixture was transferred to the separation funnel and extracted with Et_2O (100 mL). Organic phase was separated and washed with 10% HCl (2×50 mL), saturated $NaHCO_3$ (2×50 mL), brine then dried over Na_2SO_4 and evaporated. Crude product was recrystalized from acetone.

Mp: 211-213 °C, colourless solid, 1.21 g, 61 %, characterization data in accordance with ref. [5].

^{1}H NMR (500 MHz, $CDCl_3$) δ 7.94-7.91 (m, 1H), 7.78-7.73 (m, 2H), 7.71-7.67 (m, 1H), 2.67 (s, 3H).
^{13}C{^{1}H} NMR (126 MHz, $CDCl_3$) δ 173.3, 139.7, 134.1, 133.7, 131.6, 124.2, 122.5, 17.7.

Preparation of 3-phenylbenzo[*d*]isothiazole 1,1-dioxide (S3)

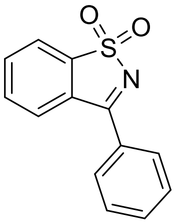

Schlenk flask was charged with magnesium turnings (1.25 g; 42.94 mol; 3.1 eq.), dry THF (15 mL) and few drops of CH_3I. Brombenzene (5.2 mL; 49.1 mol; 3 eq.) was slowly added at 0 °C, after addition was complete, reaction mixture was refluxed for 2h.

Saccharin (3 g, 16.37 mmol, 1 eq.) was added to Schlenk flask, followed by addition of dry THF (15 mL). Phenylmagnesium bromide (49.1 mmol, 3 eq.) was slowly added at 0 °C and gradually heated to rt overnight. Reaction mixture was quenched with 2 M HCl (15 mL), diluted with water, transferred to the separatory funnel, extracted with EtOAc (3×50 mL). Combined organic phase was washed with brine, dried over Na_2SO_4 and evaporated. Crude product was recrystalized from EtOAc .

Mp: 164-165 °C , pale yellow solid, 2.07 g, 52 %, characterization data in accordance with ref. [6].

^{1}H NMR (500 MHz, $CDCl_3$) δ 7.99 (d, *J*=7.38 Hz, 1H), 7.95 (d, *J*=7.74 Hz, 2H), 7.90 (d, *J*=7.51 Hz, 1H), 7.79 (t, *J*=7.38 Hz, 1H), 7.75 (t, *J*=7.38 Hz, 1H), 7.69 (t, *J*=7.38 Hz, 1H), 7.60 (t, *J*=7.61 Hz, 2H).

^{13}C{^{1}H} NMR (126 MHz, $CDCl_3$) δ 171.2, 141.1, 133.8, 133.5, 130.6, 130.4, 129.6, 129.3, 126.7, 123.1.

Preparation of 3-isopropylbenzo[*d*]isothiazole 1,1-dioxide (S4)

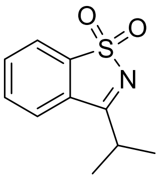

Schlenk flask was charged with magnesium turnings (1.31 g, 54 mmol, 3.1 eq.), dry THF (15 mL) and few drops of CH_3I. Isopropyl chloride (4.5 mL, 49.1 mmol, 3 eq.) was slowly added at 0 °C, after addition was complete, reaction mixture was refluxed for 2h.

Saccharin (3.1 g, 16.92 mmol, 1 eq.) was added to Schlenk flask, followed by addition of dry THF (40 mL). Isopropylmagnesium chloride (49.1 mmol, 3 eq.) was slowly added at 0 °C and gradually heated to rt overnight. Reaction mixture was quenched with 2 M HCl (15 mL), transferred to the separatory funnel, extracted with Et_2O (4×20 mL). Combined organic phase was washed with brine, dried over Na_2SO_4 and evaporated. Crude product was recrystalized from Et_2O.

Mp: 87-88 °C, white solid, 1.06 g, 31 %, characterization data in accordance with ref. [7].

^{1}H NMR (500 MHz, $CDCl_3$) δ 7.94-7.90 (m, 1H), 7.76-7.70 (m, 3H), 3.37 (sep, *J*=7.01 Hz, 1H), 1.43 (d, *J*=6.98 Hz, 6H).

^{13}C{^{1}H} NMR (126 MHz, $CDCl_3$) δ 180.5, 140.3, 133.9, 133.5, 130.7, 124.1, 122.7, 30.6, 19.9.

General procedure for palladium-catalyzed enantioselective additions of arylboronic acids to cyclic ketimines in homogeneous conditions

L1 (4.6 mg, 0.0168 mmol, 7.5 mol %) and $Pd(TFA)_2$ (3.7 mg, 0.0112 mmol, 5 mol %) were stirred in TFE (1.1 mL) in test tube (50 mL) opened to air for 2 h at 24 °C. Then substrate (0.224 mmol, 1.0 equiv) and arylboronic acid (0.336 mmol, 1.5 equiv) were added and the wall of the test tube was rinsed with TFE (1.1 mL). The mixture was stirred at the certain temperature (sand bath) in test tube open to air, after certain time the solvent was removed by rotary evaporation. The residue was purified by column chromatography with mobile phase EtOAc:Hexane (1:4) or in the case of **P1f**, **P1g**, **P1h**, **P1i**, **P1l**, **P2l**, **P3l**, **P4l** by flash chromatography with C18 stationary phase with mobile phase Water:Acetonitrile (10→30 % MeCN).

Racemic products were synthesized in a manner analogous to the general procedure using $Pd(TFA)_2$ (10 mol %) and bipyridine (15 mol %) at 60 °C (sand bath) for 24 h.

General procedure for palladium-catalyzed enantioselective additions of arylboronic acids to cyclic ketimines in heterogeneous conditions

L5·Pd(TFA)$_2$ (240 mg, 0.04 mmol Pd, 15 mol % Pd) was placed to the test tube (50 mL) and TFE (2.5 mL) was added. Then substrate (0.224 mmol, 1.0 equiv) and arylboronic acid (0.336 mmol, 1.5 equiv) were added and the wall of the test tube was rinsed with TFE (1.5 mL). The mixture was stirred open to air at the 40 °C (sand bath), after certain time the **L5·Pd(TFA)$_2$** was filtered off, washed with DCM (10 mL), MeOH (10 mL), DCM (10 mL) and filtrate was removed by rotary evaporation. The residue was purified by column chromatography with mobile phase EtOAc:Hexane (1:4) or in the case of **P1f**, **P1g**, **P1h**, **P1i**, **P1l**, **P2l**, **P3l**, **P4l** by flash chromatography with C18 stationary phase with mobile phase Water:Acetonitrile (10→30 % MeCN). **L5·Pd(TFA)$_2$** after filtration and washing was vacuum dried and used in next reaction cycle.

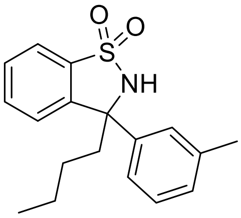

3-butyl-3-(*m*-tolyl)-2,3-dihydrobenzo[*d*]isothiazole 1,1-dioxide (P1a)

White solid, m.p. 70-71 °C, 70 mg, 99 %, characterization data in accordance with ref. [4].

^{1}H NMR (500 MHz, $CDCl_3$) δ 7.78 (d, *J*=7.83 Hz, 1H), 7.58 (t, *J*=7.59 Hz, 1H), 7.51 (t, *J*=7.55 Hz, 1H), 7.33-7.32 (m, 2H), 7.27-7.23 (m, 2H), 7.10 (d, *J*=7.53 Hz, 1H), 4.78 (s, 1H), 2.40 (td, *J*=14.13, 4.32 Hz, 1H), 2.34 (s, 3H), 2.29 (td, *J*=12.14, 4.61 Hz, 1H), 1.51-1.42 (m, 1H), 1.39-1.31 (m, 2H), 1.07-0.98 (m, 1H), 0.88 (t. *J*=7.71 Hz, 3H).

^{13}C{^{1}H} NMR (126 MHz, $CDCl_3$) δ 143.7, 142.4, 138.9, 134.5, 133.5, 129.3, 129.0, 129.0, 126.8, 124.5, 123.2, 121.5, 68.9, 40.3, 26.2, 22.8, 21.8, 14.0.

Chiral HPLC: Chiralpak AD-H, hexane/iPrOH (70/30), 230 nm, 0.5 mL/min, t_{R1}=13.86 min (minor), t_{R2}=18.23 min (major), er= 2.6:97.4 (homogeneous cond.), 96.5:3.5 (heterogeneous cond.).

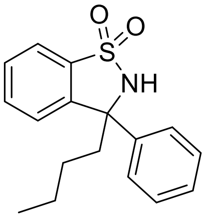

3-butyl-3-phenyl-2,3-dihydrobenzo[*d*]isothiazole 1,1-dioxide (P1b)

White solid, m.p. 143-144 °C, 69 mg, 98 %, characterization data in accordance with ref. [4].

^{1}H NMR (500 MHz, CDCl3) δ 7.77 (d, *J*=7.77 Hz, 1H), 7.59-7.53 (m, 3H), 7.50 (td, *J*=7.81 Hz, 0.92 Hz, 1H), 7.38-7.34 (m, 2H), 7.30-7.27 (m, 2H), 4.90 (s, 1H), 2.42 (ddd, *J*=14.22, 12.21, 4.23 Hz, 1H), 2.30 (ddd, *J*=14.09, 11.89, 4.46 Hz, 1H), 1.50-1.40 (m, 1H), 1.39-1.31 (m, 2H), 1.08-1.00 (m, 1H), 0.87 (t, *J*=7.35 Hz, 3H).

^{13}C{^{1}H} NMR (126 MHz, CDCl3) δ 143.7, 142.5, 134.5, 133.5, 129.4, 129.1, 128.2, 126.1, 124.4, 121.4, 68.9, 40.2, 26.2, 22.8, 14.0.

Chiral HPLC: Chiralpak AD-H, hexane/iPrOH (70/30), 230 nm, 0.8 mL/min, t_{R1}=15.28 min (minor), t_{R2}=17.44 min (major), er= 1.5:98.5 (homogeneous cond.).

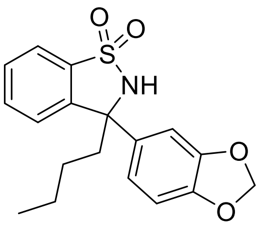

3-(benzo[*d*][1,3]dioxol-5-yl)-3-butyl-2,3-dihydrobenzo[*d*]isothiazole 1,1-dioxide (P1c)

Gummy oil, 76 mg, 98 %, characterization data in accordance with ref. [4].

^{1}H NMR (500 MHz, $CDCl_3$) δ 7.77 (d, *J*=7.96 Hz, 1H), 7.58 (td, *J*=7.58, 1.10 Hz, 1H), 7.51 (td, *J*=7.71, 1.03 Hz, 1H), 7.27 (d, *J*=7.81 Hz, 1H), 7.00-6.99 (m, 2H), 6.76 (d, *J*=8.04 Hz, 1H), 5.95-5.94 (m, 2H), 4.77 (s, 1H), 2.34 (ddd, *J*=14.15, 12.62, 4.30 Hz, 1H), 2.24 (ddd, *J*=14.15, 12.20, 4.72 Hz, 1H), 1.49-1.40 (m, 1H), 1.39-1.28 (m, 2H), 1.05-0.97 (m, 1H), 0.87 (t, *J*=7.29 Hz, 3H).

^{13}C{^{1}H} NMR (126 MHz, $CDCl_3$) δ 148.4, 147.4, 143.7, 136.4, 134.5, 133.5, 129.4, 124.4, 121.5, 119.6, 108.4, 107.1, 101.5, 68.8, 40.4, 26.2, 22.8, 14.0.

Chiral HPLC: Chiralpak AD-H, hexane/iPrOH (70/30), 230 nm, 1 mL/min, t_{R1}=17.5 min (minor), t_{R2}=20.9 min (major), er= 3.5:96.5 (homogeneous cond.).

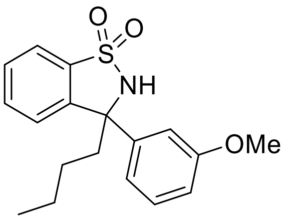

3-butyl-3-(3-methoxyphenyl)-2,3-dihydrobenzo[*d*]isothiazole 1,1-dioxide (P1d)

Gummy oil, 45 mg, 60 %, characterization data in accordance with ref. [4].

^{1}H NMR (500 MHz, $CDCl_3$) δ 7.75 (d, *J*=7.89 Hz, 1H), 7.57 (t, *J*=7.44 Hz, 1H), 7.49 (t, *J*=7.72 Hz, 1H), 7.31 (d, *J*=7.89 Hz, 1H), 7.28-7.25 (m, 1H), 7.13-7.11 (m, 2H), 6.82-6.80 (m, 1H), 4.97 (s, 1H), 3.77 (s, 3H), 2.39 (ddd, *J*=14.48, 12.46, 4.15 Hz, 1H), 2.27 (ddd, *J*=14.20, 11.95, 4.39 Hz, 1H), 1.49-1.40 (m, 1H), 1.38-1.28 (m, 2H), 1.08-1.01 (m, 1H), 0.86 (t, *J*=7.57 Hz, 3H).

^{13}C{^{1}H} NMR (126 MHz, $CDCl_3$) δ 160.0, 144.2, 143.5, 134.4, 133.5, 130.1, 129.4, 124.4, 121.4, 118.3, 113.0, 112.5, 68.8, 55.3, 40.2, 26.1, 22.7, 13.9.

Chiral HPLC: Chiralpak AD-H, hexane/iPrOH (70/30), 230 nm, 0.5 mL/min, t_{R1}=24.8 min (minor), t_{R2}=28.0 min (major), er= 2.3:97.7 (homogeneous cond.).

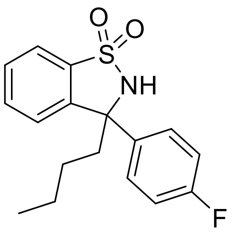

3-butyl-3-(4-fluorophenyl)-2,3-dihydrobenzo[*d*]isothiazole 1,1-dioxide (P1e)

White solid, m.p. 151-152 °C, 46 mg, 64 %, characterization data in accordance with ref. [4].

^{1}H NMR (500 MHz, $CDCl_3$) δ 7.75 (d, *J*=7.85 Hz, 1H), 7.59 (t, *J*=7.90 Hz, 1H), 7.54-7.49 (m, 3H), 7.29 (d, *J*=7.90 Hz, 1H), 7.01 (t, *J*=8.69 Hz, 2H), 5.13 (s, 1H), 2.38 (ddd, *J*=14.28, 12.44, 3.98 Hz, 1H), 2.28 (ddd, *J*=14.28, 11.94, 4.33 Hz, 1H), 1.46-1.29 (m, 3H), 1.08-0.99 (m, 1H), 0.86 (t, *J*=7.26 Hz, 3H).

^{13}C{^{1}H} NMR (126 MHz, $CDCl_3$) δ 162.3 (d, *J*=247.32 Hz), 143.5, 138.4 (d, *J*=3.22 Hz), 134.5, 133.6, 129.4, 128.1 (d, *J*=8.22 Hz), 124.3, 121.4, 115.8 (d, *J*=21.38 Hz), 68.4, 40.4, 26.1, 22.7, 13.9.

Chiral HPLC: Chiralpak AD-H, hexane/iPrOH (70/30), 230 nm, 0.5 mL/min, t_{R1}=21.9 min (minor), t_{R2}=27.2 min (major), er=2.6:97.4 (homogeneous cond.).

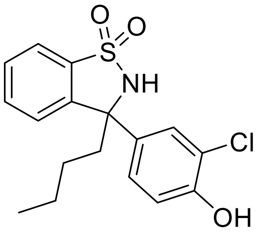

3-butyl-3-(3-chloro-4-hydroxyphenyl)-2,3-dihydrobenzo[*d*]isothiazole 1,1-dioxide (P1f)

White solid, m.p. 150-152 °C, 66 mg, 84 %

^{1}H NMR (500 MHz, MeOD) δ 7.72 (d, *J*=7.75 Hz, 1H), 7.62 (td, *J*=7.67, 1.21 Hz, 1H), 7.55-7.53 (m, 2H), 7.45 (d, *J*=7.97 Hz, 1H), 7.36 (dd, *J*=8.66, 2.49 Hz, 1H), 6.87 (d, *J*=8.57 Hz, 2H), 2.35-2.28 (m, 2H), 1.45-1.37 (m, 1H), 1.36-1.28 (m, 2H), 1.01-0.91 (m, 1H), 0.86 (t, *J*=7.30 Hz, 3H).

^{13}C{^{1}H} NMR (126 MHz, MeOD) δ 153.7, 144.9, 137.0, 135.6, 134.5, 130.4, 128.9, 126.8, 125.4, 121.7, 121.6, 117.4, 69.1, 40.9, 27.2, 23.6, 14.3.

HRMS calcd for $[C_{17}H_{18}ClNO_3S+Na]^+$ 374.0588; found $[C_{17}H_{18}ClNO_3S+Na]^+$ 374.0594; Δ = 1.76 ppm.

FT-IR (ATR) cm^{-1}: 3374, 3347, 2962, 2930, 1604, 1510, 1268, 1158, 1120, 1055, 962, 767, 569.

Chiral HPLC: Chiralpak IA, hexane/iPrOH (70/30), 230 nm, 0.5 mL/min, t_{R1}=11.6 min (minor), t_{R2}=19.4 min (major), er= 8.2:94.8 (homogeneous cond.).

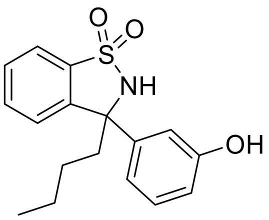

3-butyl-3-(3-hydroxyphenyl)-2,3-dihydrobenzo[*d*]isothiazole 1,1-dioxide (P1g)

White solid, m.p. 166-167 °C, 54 mg, 76 %

^{1}H NMR (500 MHz, MeOD) δ 7.72 (d, *J*=7.71 Hz, 1H), 7.63 (t, *J*=7.36 Hz, 1H), 7.55 (t, *J*=7.46 Hz, 1H), 7.46 (d, 7.80 Hz, 1H), 7.16 (t, *J*=7.94 Hz, 1H), 7.08 (d, *J*=8.12 Hz, 1H), 7.05 (t, *J*=2.03 Hz, 1H), 6.67 (dd, *J*=8.08, 2.01 Hz, 1H), 2.39-2.27 (m, 2H), 1.48-1.39 (m, 1H), 1.38-1.29 (m, 2H), 1.03-0.94 (m, 1H), 0.87 (t, *J*=7.26 Hz, 3H).

^{13}C{^{1}H} NMR (126 MHz, MeOD) δ 158.8, 146.2, 145.0, 135.7, 134.4, 130.6, 130.3, 125.6, 121.7, 118.2, 115.5, 114.3, 69.7, 41.1, 27.3, 23.7, 14.3.

HRMS calcd for $[C_{17}H_{19}NO_3S+Na]^+$ 340.0977; found $[C_{17}H_{19}NO_3S+Na]^+$ 340.0982; Δ = 1.50 ppm.

FT-IR (ATR) cm^{-1}: 3409, 3261, 2942, 1588, 1449, 1263, 1156, 1156, 1057, 871, 754, 569.

Chiral HPLC: Chiralpak IA, hexane/iPrOH (70/30), 230 nm, 1 mL/min, t_{R1}=9.61 min (minor), t_{R2}=13.74 min (major), er= 2.2:97.8 (homogeneous cond.).

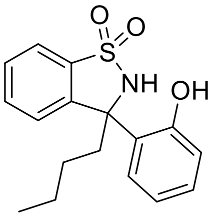

3-butyl-3-(2-hydroxyphenyl)-2,3-dihydrobenzo[*d*]isothiazole 1,1-dioxide (P1h)

White solid, m.p. 136-138 °C, 63 mg, 89 %

^{1}H NMR (500 MHz, MeOD) δ 7.82 (d, *J*=8.04 Hz, 1H), 7.74 (d, *J*=7.87 Hz, 1H), 7.70 (t, *J*=7.56 Hz, 1H), 7.59 (t, *J*=7.56 Hz, 1H), 7.25 (d, *J*=7.89 Hz, 1H), 7.12 (t, *J*=7.51 Hz, 1H), 6.83 (d, *J*=8.01 Hz, 1H), 6.75 (t, *J*=7.58 Hz, 1H), 2.74 (ddd, *J*=13.82, 12.62, 4.09, 1H), 2.19 (ddd, *J*=14.13, 11.67, 4.28 Hz, 1H), 1.44-1.36 (m, 1H), 1.33-1.25 (m, 2H), 1.05-0.96 (m, 1H), 0.86 (t, *J*=7.32 Hz, 3H).

^{13}C{^{1}H} NMR (126 MHz, MeOD) δ 156.0, 144.3, 136.7, 133.8, 130.3, 130.2, 129.2, 128.8, 126.8, 121.8, 120.4, 117.6, 70.2, 40.1, 27.7, 23.7, 14.3.

HRMS calcd for $[C_{17}H_{19}NO_3S+Na]^+$ 340.0977; found $[C_{17}H_{19}NO_3S\ Na]^+$ 340.0922; Δ = 1.30 ppm.

FT-IR (ATR) cm^{-1}: 3419, 3333, 3262, 2935, 2868, 1604, 1452, 1338, 1256, 1157, 933, 759, 572.

Chiral HPLC: Chiralpak IA, hexane/iPrOH (70/30), 230 nm, 0.5 mL/min, t_{R1}=9.2 min (minor), t_{R2}=12.7 min (major), er= 96.3:3.7 (homogeneous cond.).

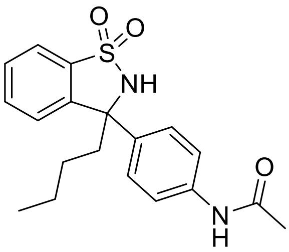

***N*-(4-(3-butyl-1,1-dioxido-2,3-dihydrobenzo[*d*]isothiazol-3-yl)phenyl)acetamide (P1i)**

White solid, m.p. 258-259 °C, 79 mg, 99 %

^{1}H NMR (500 MHz, DMSO) δ 9.97 (s, 1H), 8.50 (s, 1H), 7.75 (d, *J*=7.72 Hz, 1H), 7.67-7.62 (m, 2H), 7.56-7.50 (m, 5H), 2.30-2.24 (m, 2H), 2.01 (s, 3H), 1.29-1.22 (m, 3H), 0.93-0.85 (m, 1H), 0.81 (t, *J*=7.12 Hz, 3H).

$^{13}C\{^{1}H\}$ NMR (126 MHz, DMSO) δ 168.3, 143.3, 138.4, 138.1, 134.4, 133.2, 129.2, 126.3, 124.4, 120.5, 118.9, 67.9, 25.8, 23.9, 22.1, 14.0.

HRMS calcd for $[C_{19}H_{22}N_2O_3S+H]^+$ 359.1423; found $[C_{19}H_{22}N_2O_3S+H]^+$ 359.1431; Δ = 2.23 ppm.

FT-IR (ATR) cm^{-1}: 3350, 3110, 2953, 2772, 1668, 1517, 1406, 1255, 1127, 968, 831, 523.

Chiral HPLC: Chiralpak IA, hexane/iPrOH (70/30), 230 nm, 1 mL/min, t_{R1}=9.52 min (minor), t_{R2}=11.89 min (major), er= 3.3:96.7 (homogeneous cond.), 5.2:94.8 (heterogeneous cond.).

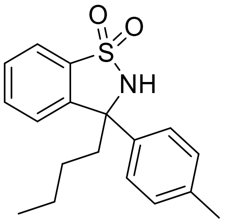

3-butyl-3-(*p*-tolyl)-2,3-dihydrobenzo[*d*]isothiazole 1,1-dioxide (P1j)

White solid, m.p. 170-171 °C, 70 mg, 99 %, characterization data in accordance with ref. [4].

^{1}H NMR (500 MHz, $CDCl_3$) δ 7.77 (d, *J*=7.71 Hz, 1H), 7.56 (td, *J*=7.66, 1.19 Hz, 1H), 7.49 (td, *J*=7.80, 0.94 Hz, 1H), 7.40 (m, 2H), 7.26 (d, *J*=7.72 Hz, 1H), 7.16 (m, 2H), 4.80 (s, 1H), 2.39 (ddd, *J*=14.23, 12.37, 4.18 Hz, 1H), 2.32 (s, 3H), 2.31-2.26 (m, 1H), 1.50-1.41 (m, 1H), 1.39-1.31 (m, 2H), 1.07-0.99 (m, 1H), 0.87 (t, *J*=7.37 Hz, 3H).

$^{13}C\{^1H\}$ NMR (126 MHz, $CDCl_3$) δ 143.9, 139.5, 138.1, 134.5, 133.5, 129.7, 129.3, 126.1, 124.4, 121.4, 68.8, 40.2, 26.2, 22.8, 21.0, 14.0.

Chiral HPLC: Chiralpak AD-H, hexane/iPrOH (70/30), 230 nm, 0.5 mL/min, t_{R1}=26.4 min (major), t_{R2}=32.0 min (minor), er=97.2:2.8 (homogeneous cond.), 96:4 (heterogeneous cond.).

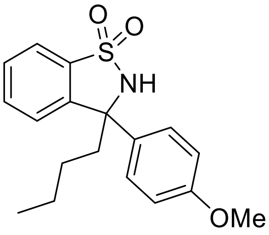

3-butyl-3-(4-methoxyphenyl)-2,3-dihydrobenzo[*d*]isothiazole 1,1-dioxide (P1k)

White solid, m.p. 110-112 °C, 73 mg, 99 %, characterization data in accordance with ref. [4].

^1H NMR (500 MHz, $CDCl_3$) δ 7.76 (d, *J*=7.86 Hz, 1H), 7.57 (t, *J*=7.50 Hz, 1H), 7.50 (t, *J*=7.71 Hz, 1H), 7.43 (m, 2H), 7.25 (d, *J*=7.76 Hz, 1H), 6.86 (m, 2H), 4.85 (s, 1H), 3.77 (s, 3H), 2.38 (td, *J*=14.19, 4.37 Hz, 1H), 2.28 (td, *J*=14.55, 4.37 Hz, 1H), 1.49-1.40 (m, 1H), 1.38-1.30 (m, 2H), 1.06-0.97 (m, 1H), 0.86 (t, *J*=7.33 Hz, 3H).

$^{13}C\{^1H\}$ NMR (126 MHz, $CDCl_3$) δ 159.3 144.0, 134.5, 134.5, 133.5, 129.3, 127.5, 124.4, 121.4, 114.3, 68.6, 55.4, 40.2, 26.2, 22.8, 14.0.

Chiral HPLC: Chiralpak AD-H, hexane/iPrOH (70/30), 230 nm, 1 mL/min, t_{R1}=13.6 min (minor), t_{R2}=18.44 min (major), er= 95.5:4.5 (homogeneous cond.), 99.3:0.7 (homogeneous cond.), 94.8:5.2 (heterogeneous cond.).

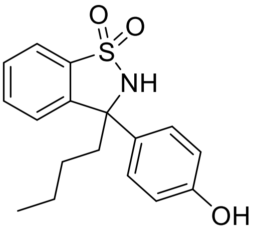

3-butyl-3-(4-hydroxyphenyl)-2,3-dihydrobenzo[*d*]isothiazole 1,1-dioxide (P1l)

White solid, m.p. 191-192 °C, 70 mg, 99 %

^1H NMR (500 MHz, MeOD) δ 7.71 (d, *J*=7.82, 1H), 7.61 (t, *J*=7.64 Hz, 1H), 7.52 (t, *J*=7.67 Hz, 1H), 7.42-7.40 (m, 3H), 6.74 (d, *J*=8.65 Hz, 2H), 2.32 (td, J=10.15 Hz, 4.55 Hz, 2H), 1.48-1.38 (m, 1H), 1.37-1.29 (m, 2H), 1.01-0.92 (m,1H), 0.87 (t, *J*=7.35 Hz, 3H).

$^{13}C\{^1H\}$ NMR (126 MHz, MeOD) δ 158.0, 145.6, 135.6, 135.4, 134.4, 130.1, 128.4, 125.5, 121.6, 116.2, 69.6, 41.1, 27.3, 23.7, 14.3.

HRMS calcd for $[C_{17}H_{19}NO_3S+Na]^+$ 298.0508; found $[C_{17}H_{19}NO_3S+Na]^+$ 298.0512; Δ = 1.31 ppm.

FT-IR (ATR) cm^{-1}: 3328, 2956, 2868, 1613, 1513, 1452, 1362, 1272, 1158, 1054, 939, 834, 755, 525.

Chiral HPLC: Chiralpak IA, hexane/iPrOH (70/30), 230 nm, 1 mL/min, t_{R1}=7.22 min (minor), t_{R2}=13.30 min (major), er= 5.6:94.4 (homogeneous cond.), 2.1:97.9 (homogeneous cond.), 6:94 (heterogeneous cond.).

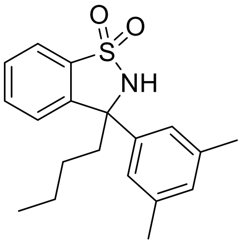

3-butyl-3-(3,5-dimethylphenyl)-2,3-dihydrobenzo[*d*]isothiazole 1,1-dioxide (P1m)

Gummy oil, 73 mg, 99 %, characterization data in accordance with ref. [4].

^{1}H NMR (500 MHz, $CDCl_3$) δ 7.77 (d, *J*=7.78 Hz, 1H), 7.58 (t, *J*=7.56 Hz, 1H), 7.50 (t, *J*=7.72 Hz, 1H), 7.27 (d, *J*=7.94 Hz, 1H), 7.12 (s, 2H), 6.93 (s, 1H), 4.81 (s, 1H), 2.38 (td, *J*=14.40, 4.08 Hz, 1H), 2.30 (s, 6H), 2.28-2.25 (m, 1H), 1.51-1.42 (m, 1H), 1.39-1.31 (m, 2H), 1.05-0.96 (m, 1H), 0.87 (t, *J*=7.35 Hz, 3H).

$^{13}C\{^1H\}$ NMR (126 MHz, $CDCl_3$) δ 143.8, 142.5, 138.7, 134.5, 133.5, 129.9, 129.3, 124.5, 123.9, 121.4, 68.9, 40.3, 26.2, 22.8, 21.6, 14.0.

Chiral HPLC: Chiralpak AD-H, hexane/iPrOH (70/30), 230 nm, 0.5 mL/min, t_{R1}=10.9 min (minor), t_{R2}=12.6 min (major), er= 2:98 (homogeneous cond.), 96.7:3.3 (heterogeneous cond.)

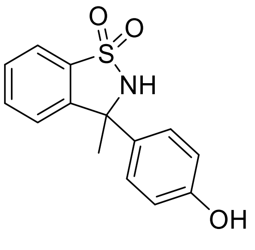

3-(4-hydroxyphenyl)-3-methyl-2,3-dihydrobenzo[*d*]isothiazole 1,1-dioxide (P2l)

White solid, m.p. 200-202 °C, 60 mg, 98 %

^{1}H NMR (500 MHz, MeOD) δ 7.73 (d, *J*=7.61 Hz, 1H), 7.59 (t, *J*=7.53 Hz, 1H), 7.53 (t, *J*=7.53 Hz, 1H), 7.35-7.32 (m, 3H), 6.74 (d, *J*=8.73 Hz, 2H), 1.96 (s, 3H).

$^{13}C\{^1H\}$ NMR (126 MHz, MeOD) δ 158.1, 147.2, 135.6, 135.5, 134.4, 130.2, 128.6, 125.7, 121.5, 116.1, 66.0, 28.9.

HRMS calcd for $[C_{14}H_{13}NO_3S+Na]^+$ 298.0508; found $[C_{14}H_{13}NO_3S +Na]^+$ 298.0512; Δ = 1.31 ppm.

FT-IR (ATR) cm^{-1}: 3323, 3237, 2973, 2927, 1611, 1513, 1439, 1290, 1223, 1157, 1055, 945, 836, 748, 550.

Chiral HPLC: Chiralpak IA, hexane/iPrOH (70/30), 230 nm, 1 mL/min, t_{R1}=7.1 min (minor), t_{R2}=9.3 min (major), er=3.5:96.5 (homogeneous cond.).

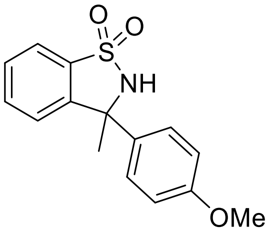

3-(4-methoxyphenyl)-3-methyl-2,3-dihydrobenzo[*d*]isothiazole 1,1-dioxide (P2k)

White solid, m.p. 136-137 °C, 64 mg, 99 %, characterization data in accordance with ref. [8].

^{1}H NMR (500 MHz, $CDCl_3$) δ 7.79 (d, *J*=7,8 Hz, 1H), 7.57 (dt, *J*=7.45 Hz, *J*=1.26 Hz, 1H), 7.52 (dt, *J*=7.45 Hz, *J*=1.26 Hz, 1H), 7.38-7.35 (m, 2H), 7.18 (d, *J*=7,86 Hz, 1H), 6.89-6.85 (m, 2H), 4.76 (s, 1H), 3.79 (s, 3H), 2.04 (s, 3H).

$^{13}C\{^1H\}$ NMR (126 MHz, $CDCl_3$) δ 159.6, 145.7, 134.8, 134.6, 133.6, 129.3, 127.7, 124.6, 121.3, 114.3, 65.2, 55.4, 28.8.

Chiral HPLC: Chiralpak AD-H, hexane/iPrOH (70/30), 230 nm, 0,8 mL/min, t_{R1}=12.1 min (minor), t_{R2}=20.1 min (major), er=3.4:96.6 (homogeneous cond., rt), 3.6:96.4 (homogeneous cond., 40 °C). 94.5:5.5 (heterogeneous cond.).

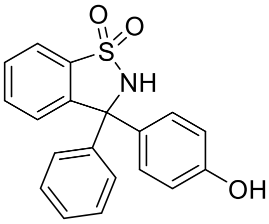

3-(4-hydroxyphenyl)-3-phenyl-2,3-dihydrobenzo[*d*]isothiazole 1,1-dioxide (P3l)

White solid, m.p. 100-101 °C, 73 mg, 97 %

^{1}H NMR (500 MHz, MeOD) δ 7.79 (d, *J*=7.82 Hz, 1H), 7.66 (t, *J*=7.76 Hz, 1H), 7.60 (t, *J*=7.82 Hz, 1H), 7.46 (d, *J*=7.97 Hz, 1H), 7.39-7.37 (m, 2H), 7.34-7.28 (m, 3H), 7.09-7.06 (m, 2H), 6.75-6.72 (m, 2H).

$^{13}C\{^1H\}$ NMR (126 MHz, MeOD) δ 158.5, 145.2, 144.7, 136.2, 135.6, 134.1, 130.6, 130.3, 129.3, 128.9, 128.8, 127.9, 121.8, 116.0, 72.9.

HRMS calcd for $[C_{19}H_{15}NO_3S+H]^+$ 338.0845; found $[C_{19}H_{15}NO_3S+H]^+$ 338.0849; Δ = 1.29 ppm.

FT-IR (ATR) cm^{-1}: 3412, 3257, 3061, 1612, 1512, 1272, 1154, 1066, 969, 832, 699, 570, 405.

Chiral HPLC: Chiralpak IA, hexane/iPrOH (70/30), 230 nm, 1 mL/min, t_{R1}=10.2 min (minor), t_{R2}=13.8 min (major), er=4:96 (homogeneous cond.).

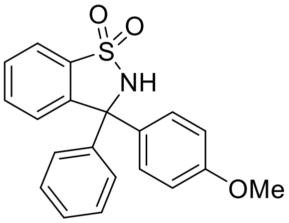

3-(4-methoxyphenyl)-3-phenyl-2,3-dihydrobenzo[*d*]isothiazole 1,1-dioxide (P3k)

White solid, m.p. 67-68 °C, 69 mg, 88 %, characterization data in accordance with ref. [8].

^{1}H NMR (500 MHz, $CDCl_3$) δ 7.81 (dt, *J*=7.73, 0.91 Hz, 1H), 7.62 (td, *J*=7.67, 1.21 Hz, 1H), 7.56 (td, *J*=7.71, 1.07 Hz, 1H), 7,42-7.38 (m, 3H), 7.36-7.30 (m, 3H), 7.25-7.22 (m, 2H), 6.87-6.84 (m, 2H), 4.89 (s, 1H), 3.79 (s, 3H).

^{13}C{^{1}H} NMR (126 MHz, $CDCl_3$) δ 159.6, 143.5, 143.2, 135.2, 135.2, 133.3, 129.6, 129.2, 128.8, 128.4, 127.8, 126.7, 121.6, 114.1, 72.3, 55.4.

Chiral HPLC: Chiralpak OD-H, hexane/iPrOH (80/20), 230 nm, 0.6 mL/min, t_{R1}=18.3 min (minor), t_{R2}=21.2 min (major), er=3.8:96.2 (homogeneous cond.).

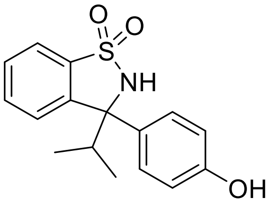

3-(4-hydroxyphenyl)-3-isopropyl-2,3-dihydrobenzo[*d*]isothiazole 1,1-dioxide (P4l)

White solid, m.p. 214-216 °C, 66 mg, 98 %

^{1}H NMR (500 MHz, MeOD) δ 7.65 (d, *J*=7.71 Hz, 1H), 7.57-7.53 (m, 3H), 7.49 (d, *J*=7.85 Hz, 1H), 7.45 (t, *J*=7.68 Hz, 1H), 6.77 (d, *J*=8.80 Hz, 2H), 3,00 (sep, *J*=6.74 Hz, 1H), 0.92 (d, *J*=6.73 Hz, 3H), 0.72 (d, *J*=6,68 Hz, 3H).

^{13}C{^{1}H} NMR (126 MHz, MeOD) δ 157.8, 145.3, 135.0, 134.7, 134.4, 130.0, 128.3, 125.2, 121.6, 116.3, 73.8, 36.6, 18.0, 17.1.

HRMS calcd for $[C_{16}H_{17}NO_3S+Na]^+$ 326.0821; found $[C_{16}H_{17}NO_3S+Na]^+$ 326.0824; Δ = 1.07 ppm.

FT-IR (ATR) cm^{-1}: 3471, 3427, 3257, 2973, 2877, 1613, 1515, 1450, 1363, 1262, 1154, 1126, 1013, 835, 752, 579.

Chiral HPLC: Chiralpak IA, hexane/iPrOH (70/30), 230 nm, 1 mL/min, t_{R1}=7.6 min (minor), t_{R2}=18.3 min (major), er=12.1:87.9 (homogeneous cond.).

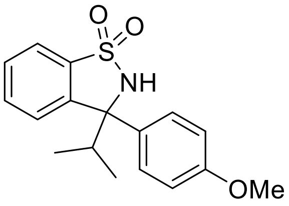

3-isopropyl-3-(4-methoxyphenyl)-2,3-dihydrobenzo[*d*]isothiazole 1,1-dioxide (P4k)

White solid, m.p. 153-154 °C, 70 mg, 99 %, characterization data in accordance with ref. [9].

^{1}H NMR (500 MHz, $CDCl_3$) δ 7.72 (d, *J*=7,84 Hz, 1H), 7.59-7.55 (m, 2H), 7.53 (d, *J*=7,52 Hz, 1H), 7.45 (t, *J*=7,73 Hz, 1H), 7.32 (d, *J*=7,86 Hz, 1H), 6.90-6.86 (m, 2H), 4.83 (s, 1H), 3.77 (s, 3H), 2.94 (sept, *J*=6,68 Hz, 1H), 0.97 (d, *J*=6,71 Hz, 3H), 0.81 (d, *J*=6,71 Hz, 3H).

^{13}C{^{1}H} NMR (126 MHz, $CDCl_3$) δ 159.1, 143.7, 134.4, 133.7, 133.6, 129.1, 127.2, 123.9, 121.5, 114.4, 72.5, 55.4, 35.7, 17.8, 16.9.
Chiral HPLC: Chiralpak AD-H, hexane/iPrOH (90/10), 230 nm, 1 mL/min, t_{R1}=18.3 min (minor), t_{R2}=25.5 min (major), er=15.8:84.2 (homogeneous cond.).

S3-DFT calculations

The DFT calculation were performed to elucidate the reaction rate enhancement in the case od 2-hydroxy derivative. Therefore, we calculated energies of precomplexes and key transition states using B3LYP functional with combination of 6-31+G* basis set for all atoms and SDD pseudopotential for Pd with empirical dispersion GD3bj and solvent included (CPCM, ethanol) as implemented in Gaussian 16 software [10] for 2-hydroxy and 2-methyl derivatives. The starting geometries were inspired by the work of Quan et al [11] and *n*-butyl substituent was substituted by methyl. The comparison of energies and geometries shows figures below. The results shows that in contradictory to the experimental observations, the reaction barrier for 2-methyl derivative is a little bit lower in energy in comparison to 2-hydroxyderivative (Figure S1). Our first idea, that the possible formation of hydrogen bond in transition state is responsible for rate enhancement thus does not seems probable. This is clear from the structures, where in precomplex the distance between H and acceptor oxygen is 2.0 A, while the geometry of TS does not enable this weak interaction.

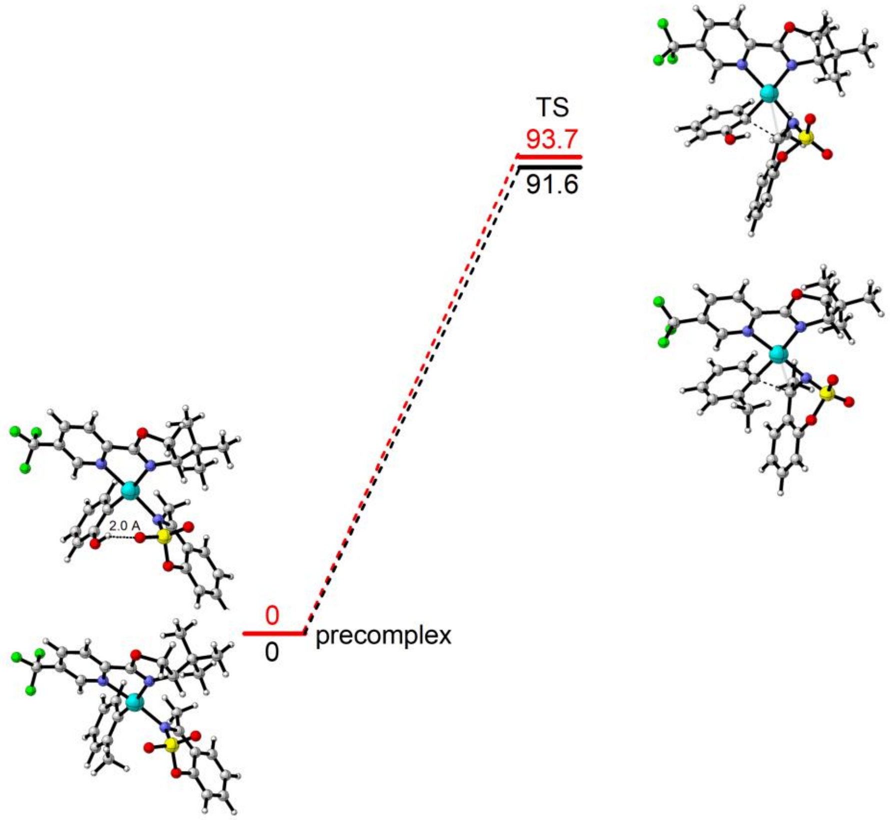

Figure S1 Energy profile of the migratory insertion step

Coordinates

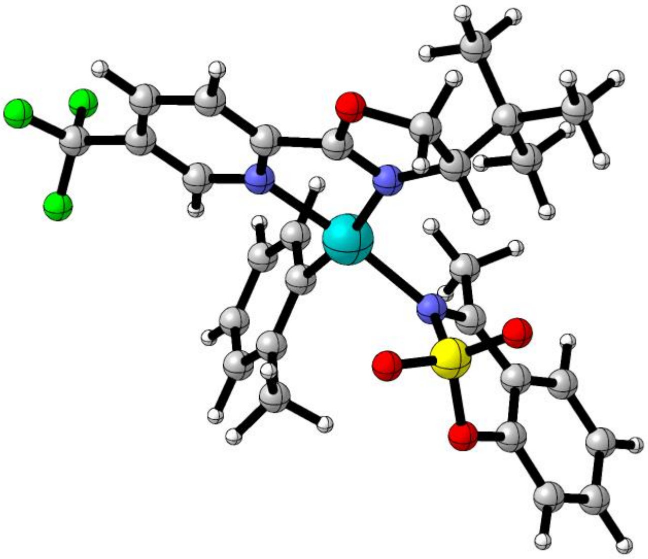

69
Precomplex 2-Me 1 1 scf done: -2375.299730 Sum of electronic and thermal Free Energies - -2374.835981 imag. freq.: 0

C	2.6590500	1.6175110	-0.4310610
C	3.9685840	1.9943700	-0.6957290
C	4.9761590	1.0319840	-0.6036770
C	3.2794010	-0.5793770	-0.0129270
N	2.3188980	0.3444540	-0.0913320
C	1.5318200	2.5566340	-0.4805280
O	1.7480070	3.8343130	-0.7962560
C	0.4107510	4.4445520	-0.8761900
C	-0.5180480	3.4102410	-0.1927890
N	0.3291290	2.1934630	-0.2041960
C	-0.9801870	3.7876430	1.2459960
C	-1.7941420	2.6313990	1.8513880
C	0.2205700	4.0868150	2.1594890
C	-1.8889280	5.0261650	1.1360800
Pd	0.2535640	0.0308910	0.0945500
C	0.3030910	-2.9323890	-0.4988770
C	0.4981610	-1.9228310	0.4603340
C	0.5649280	-4.2636620	-0.1260950
C	0.9492310	-2.2548910	1.7463980
C	1.0055720	-4.5955170	1.1565730
C	1.2018820	-3.5844700	2.1007600
C	-2.6671180	-0.7835450	0.8254130
C	-4.0293530	-1.1507070	0.4616380

C	-4.4194670	-1.2098480	-0.8918850
C	-4.9769260	-1.5555390	1.4272660
C	-5.6648960	-1.6653860	-1.2943990
C	-6.5761420	-2.0626740	-0.3145460
C	-6.2353020	-2.0005400	1.0438120
O	-3.5160080	-0.8616830	-1.8930380
S	-2.4703630	0.3437510	-1.5050450
O	-1.4554290	0.3238500	-2.5356600
O	-3.2242120	1.5566860	-1.2213910
N	-1.8247480	-0.2474260	-0.0354650
H	6.0083850	1.2951910	-0.8023690
H	4.1909380	3.0192110	-0.9667620
H	2.9655930	-1.5808240	0.2508470
H	0.4726650	5.4100580	-0.3790480
H	0.1886440	4.5802560	-1.9362040
H	-1.4054600	3.2259280	-0.8014160
H	-1.1674870	1.7520780	2.0210790
H	-2.2080240	2.9377630	2.8187140
H	-2.6258620	2.3451110	1.1999420
H	0.8795560	3.2157900	2.2480060
H	0.8158590	4.9347190	1.8027570
H	-0.1343900	4.3369450	3.1654410
H	-2.7521400	4.8238220	0.4905210
H	-2.2655230	5.2995690	2.1280240
H	-1.3604810	5.8979350	0.7342740
H	0.4255010	-5.0499860	-0.8653410
H	1.1172590	-1.4730210	2.4835460
H	1.2019970	-5.6332890	1.4123320
H	1.5526080	-3.8213760	3.1019970
H	-4.7236620	-1.5184790	2.4794880
H	-5.9083430	-1.6970520	-2.3505050
H	-7.5591510	-2.4124090	-0.6132170
H	-6.9526970	-2.3024170	1.7991470
C	-2.1761760	-1.0416800	2.2123520
H	-2.8300990	-0.5310580	2.9277050
H	-2.1985330	-2.1126240	2.4365870
H	-1.1633600	-0.6683440	2.3379780
C	5.6487560	-1.3602910	-0.1123550
C	4.6204470	-0.2675550	-0.2582780
F	6.8429170	-1.0109090	-0.6351160
F	5.2530060	-2.5020810	-0.7255900
F	5.8575500	-1.6717270	1.1929550
C	-0.1711390	-2.6390460	-1.9025050
H	-1.2611980	-2.5235830	-1.9377620
H	0.2621760	-1.7134590	-2.2900990
H	0.0905210	-3.4579980	-2.5807100

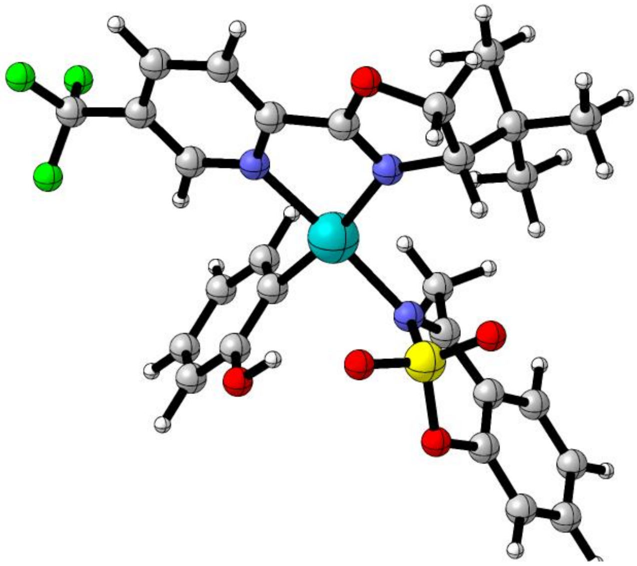

67

Precomplex 2-OH 1 1 scf done: -2411.209364 Sum of electronic and thermal Free Energies -2410.769054 imag. freq.: 0

C	2.6176120	1.6875090	-0.3859560
C	3.9229690	2.1103410	-0.5949970
C	4.9586500	1.1843710	-0.4553460
C	3.2965170	-0.4866090	0.0643540
N	2.3089500	0.4032050	-0.0614360
C	1.4592130	2.5836620	-0.4828540
O	1.6340190	3.8553730	-0.8382790
C	0.2772930	4.4097260	-0.9789680
C	-0.6343660	3.3608440	-0.2938700
N	0.2645930	2.1823480	-0.2210090
C	-1.1778570	3.7765310	1.1040850
C	-1.9675030	2.6092370	1.7181140
C	-0.0349000	4.1645380	2.0574480
C	-2.1326800	4.9675600	0.9006670
Pd	0.2588560	0.0235800	0.0701350
C	0.4805700	-2.8801600	-0.6888120
C	0.5588720	-1.9394970	0.3497860
C	0.7800770	-4.2293920	-0.4486520
C	0.9439250	-2.3802530	1.6235380
C	1.1598230	-4.6493040	0.8258750
C	1.2448750	-3.7245330	1.8717110
C	-2.6329900	-0.7797270	0.8825380
C	-4.0141390	-1.1319420	0.5858260
C	-4.4416730	-1.3052620	-0.7455170
C	-4.9541050	-1.3900190	1.6074780
C	-5.7181860	-1.7293950	-1.0757950
C	-6.6225940	-1.9782310	-0.0416370
C	-6.2431270	-1.8025610	1.2962610
O	-3.5415690	-1.1184120	-1.7945100
S	-2.4321690	0.0621680	-1.5740230
O	-1.4107730	-0.1822640	-2.5782320
O	-3.0967830	1.3521590	-1.4909650
N	-1.8009360	-0.3423160	-0.0414330

H	5.9886190	1.4839550	-0.6097380
H	4.1203180	3.1421130	-0.8590290
H	3.0067240	-1.4984320	0.3147440
H	0.2858410	5.3906390	-0.5090760
H	0.0865850	4.5064300	-2.0491580
H	-1.4832520	3.1096010	-0.9325140
H	-1.3072220	1.7713550	1.9533660
H	-2.4429570	2.9313310	2.6512560
H	-2.7524180	2.2582580	1.0400090
H	0.6581150	3.3291600	2.2080810
H	0.5373480	5.0256230	1.6946620
H	-0.4460180	4.4347080	3.0364670
H	-2.9572330	4.6995760	0.2290420
H	-2.5636010	5.2662410	1.8626940
H	-1.6248000	5.8439380	0.4827640
H	0.7142850	-4.9325770	-1.2746790
H	1.0291220	-1.6639060	2.4371170
H	1.3916020	-5.6966660	0.9985790
H	1.5438750	-4.0391760	2.8675960
H	-4.6715150	-1.2615220	2.6449290
H	-5.9897450	-1.8538790	-2.1180230
H	-7.6298110	-2.3020800	-0.2831590
H	-6.9549460	-1.9903170	2.0926600
C	-2.1094550	-0.9392480	2.2722550
H	-2.7083170	-0.3345110	2.9615760
H	-2.1848320	-1.9848480	2.5869180
H	-1.0733480	-0.6166870	2.3368830
O	0.1370720	-2.5570380	-1.9763850
H	-0.1216070	-1.6187710	-2.0405420
C	5.6984420	-1.1756440	0.0839210
C	4.6347150	-0.1266110	-0.1207690
F	6.8696690	-0.8312210	-0.4921570
F	5.3260800	-2.3719710	-0.4293300
F	5.9456580	-1.3763350	1.4042340¨

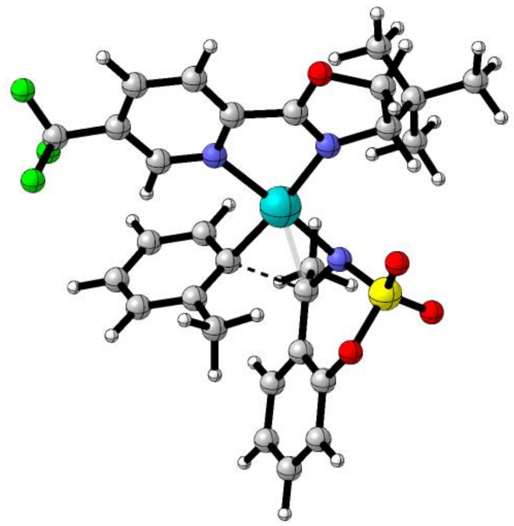

69
TS 2-Me 1 1 scf done: -2375.267057 Sum of electronic and thermal Free Energies -2374.801088 imag. freq.: 1 (-328.7)

C	2.6254000	1.3868590	-0.6370600
C	3.9618680	1.4575890	-1.0051020
C	4.7562430	0.3194820	-0.8527010
C	2.8176050	-0.8326650	0.0071680
N	2.0608130	0.2562860	-0.1379800
C	1.6952620	2.5162940	-0.7256050
O	2.1061480	3.6951970	-1.1820530
C	0.8929510	4.5305530	-1.2459150
C	-0.1522140	3.7536520	-0.4065400
N	0.4690830	2.4070490	-0.3444220
C	-0.4280070	4.3440140	1.0075540
C	-1.4380710	3.4567710	1.7532730
C	0.8652230	4.4437520	1.8340840
C	-1.0499790	5.7413270	0.8210780
Pd	-0.0186810	0.4070430	0.1636910
C	-0.1600170	-2.5394250	-0.3407870
C	-0.3160860	-1.6066000	0.7221530
C	0.5187860	-3.7380750	-0.0774930
C	0.2444440	-1.8982200	1.9900340
C	1.0449340	-4.0186190	1.1838280
C	0.9249420	-3.0857360	2.2242510
C	-2.2272700	-0.8527000	0.9877750
C	-3.0851990	-1.8349220	0.2805400
C	-3.5990460	-1.5873380	-1.0037590
C	-3.3955350	-3.0758480	0.8704130
C	-4.3986560	-2.5068220	-1.6760610
C	-4.6804670	-3.7292810	-1.0707880
C	-4.1748220	-4.0143910	0.2054890
O	-3.2879100	-0.4187820	-1.6961440
S	-3.0544800	0.9293240	-0.7440620
O	-2.4108740	1.8888470	-1.6273290
O	-4.3506090	1.2452350	-0.1445700
N	-2.0324460	0.4280830	0.4356620
H	5.8046210	0.3403110	-1.1269250
H	4.3676320	2.3811930	-1.3996720
H	2.3395450	-1.7142450	0.4092900
H	1.1642430	5.5080080	-0.8542920
H	0.6208820	4.6076610	-2.3000300
H	-1.0972190	3.6684930	-0.9430910
H	-1.0407520	2.4563910	1.9371090
H	-1.6822330	3.9099980	2.7208950
H	-2.3695750	3.3496300	1.1866000
H	1.3107320	3.4558400	1.9959580
H	1.6151050	5.0863180	1.3586460
H	0.6433640	4.8726420	2.8175490
H	-1.9612790	5.6885170	0.2131010
H	-1.3200770	6.1577840	1.7978510
H	-0.3625260	6.4484310	0.3439490
H	0.6482640	-4.4547240	-0.8844680
H	0.1502410	-1.1797510	2.7958600
H	1.5635120	-4.9576200	1.3535000

H	1.3561420	-3.2865070	3.1999520
H	-2.9975210	-3.3152420	1.8492850
H	-4.7750580	-2.2560980	-2.6620060
H	-5.2949370	-4.4570200	-1.5913070
H	-4.3902850	-4.9670930	0.6780460
C	-2.3674250	-0.7799140	2.4945690
H	-3.3912940	-0.4338410	2.6856800
H	-2.2214190	-1.7343540	2.9980410
H	-1.6822460	-0.0368350	2.9033410
C	4.9608780	-2.1100590	-0.1812070
C	4.1727010	-0.8342410	-0.3368550
F	6.2857880	-1.8759740	-0.0641490
F	4.7940300	-2.9277930	-1.2526410
F	4.5712310	-2.8103640	0.9097030
C	-0.6419740	-2.2999050	-1.7474490
H	0.1904090	-2.4383440	-2.4469280
H	-1.4162390	-3.0263650	-2.0193160
H	-1.0354710	-1.2962470	-1.8990180

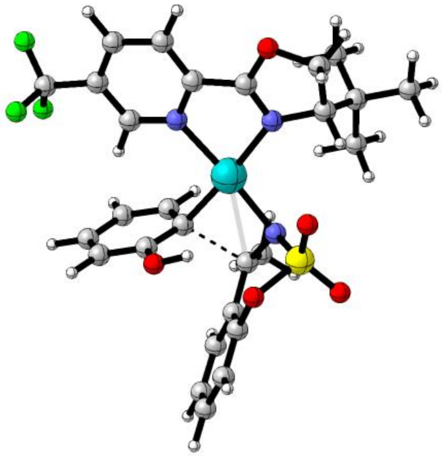

67

TS 2-OH 1 1 scf done:-2411.176156 Sum of electronic and thermal Free Energies -2410.733384 imag. freq.: 1 (-342.5)

C	2.7344740	1.1796580	-0.6207490
C	4.0715120	1.1350990	-0.9901540
C	4.7654530	-0.0667670	-0.8363380
C	2.7366370	-1.0487490	0.0258550
N	2.0761990	0.1010390	-0.1210800
C	1.9030210	2.3845570	-0.7068510
O	2.4052470	3.5235790	-1.1719360
C	1.2634520	4.4557020	-1.2313960
C	0.1620000	3.7676630	-0.3856290
N	0.6752340	2.3783100	-0.3161650
C	-0.0650490	4.3845270	1.0254060
C	-1.1246120	3.5685190	1.7839750
C	1.2374110	4.4053970	1.8433250
C	-0.5950990	5.8180140	0.8316120

Pd	0.0225290	0.4295550	0.1885110
C	-0.4406780	-2.4742550	-0.3759900
C	-0.4730300	-1.5334720	0.6968000
C	0.0668710	-3.7675050	-0.1797080
C	0.0675800	-1.9520860	1.9457590
C	0.5683530	-4.1351580	1.0616490
C	0.5852320	-3.2207260	2.1341410
C	-2.3023670	-0.6424270	1.0062010
C	-3.2524300	-1.5574660	0.3281460
C	-3.6011540	-1.3799320	-1.0219240
C	-3.8097750	-2.6648010	0.9954130
C	-4.4635880	-2.2345970	-1.6949200
C	-4.9918640	-3.3288960	-1.0106760
C	-4.6646410	-3.5411360	0.3352950
O	-3.0360650	-0.3290510	-1.7564850
S	-2.8921760	1.0900930	-0.8641930
O	-2.1378050	1.9857370	-1.7249860
O	-4.2376410	1.4519850	-0.4255760
N	-1.9960150	0.6121150	0.4275040
H	5.8117440	-0.1357540	-1.1108860
H	4.5549100	2.0201940	-1.3855330
H	2.1848150	-1.8843700	0.4327630
H	1.6153870	5.4081490	-0.8422280
H	0.9941940	4.5536350	-2.2843840
H	-0.7878160	3.7532000	-0.9213120
H	-0.7794580	2.5534930	1.9934860
H	-1.3491050	4.0550650	2.7400460
H	-2.0573850	3.4952160	1.2141270
H	1.6237230	3.3923230	2.0019200
H	2.0215880	5.0021470	1.3636650
H	1.0480410	4.8458730	2.8284560
H	-1.5184510	5.8198060	0.2397900
H	-0.8188020	6.2638590	1.8071080
H	0.1302220	6.4705020	0.3330310
H	0.0708450	-4.4576200	-1.0173320
H	0.0788070	-1.2498050	2.7714560
H	0.9625210	-5.1373730	1.2001020
H	0.9986430	-3.5085180	3.0950600
H	-3.5618830	-2.8484740	2.0335070
H	-4.7016440	-2.0384170	-2.7347660
H	-5.6625820	-4.0102890	-1.5243510
H	-5.0791900	-4.3901170	0.8689250
C	-2.3964030	-0.5314150	2.5119810
H	-3.3796410	-0.0954600	2.7287520
H	-2.3140480	-1.4874340	3.0268620
H	-1.6362210	0.1540470	2.8878020
O	-0.8743300	-2.2061950	-1.6237730
H	-1.1629380	-1.2802120	-1.7248790
C	4.7673990	-2.5024040	-0.1611350
C	4.0864650	-1.1661680	-0.3181430
F	6.1009890	-2.3717590	0.0091130
F	4.5805230	-3.2839460	-1.2557280
F	4.2849340	-3.1910100	0.8991680

S4-Hammett analysis

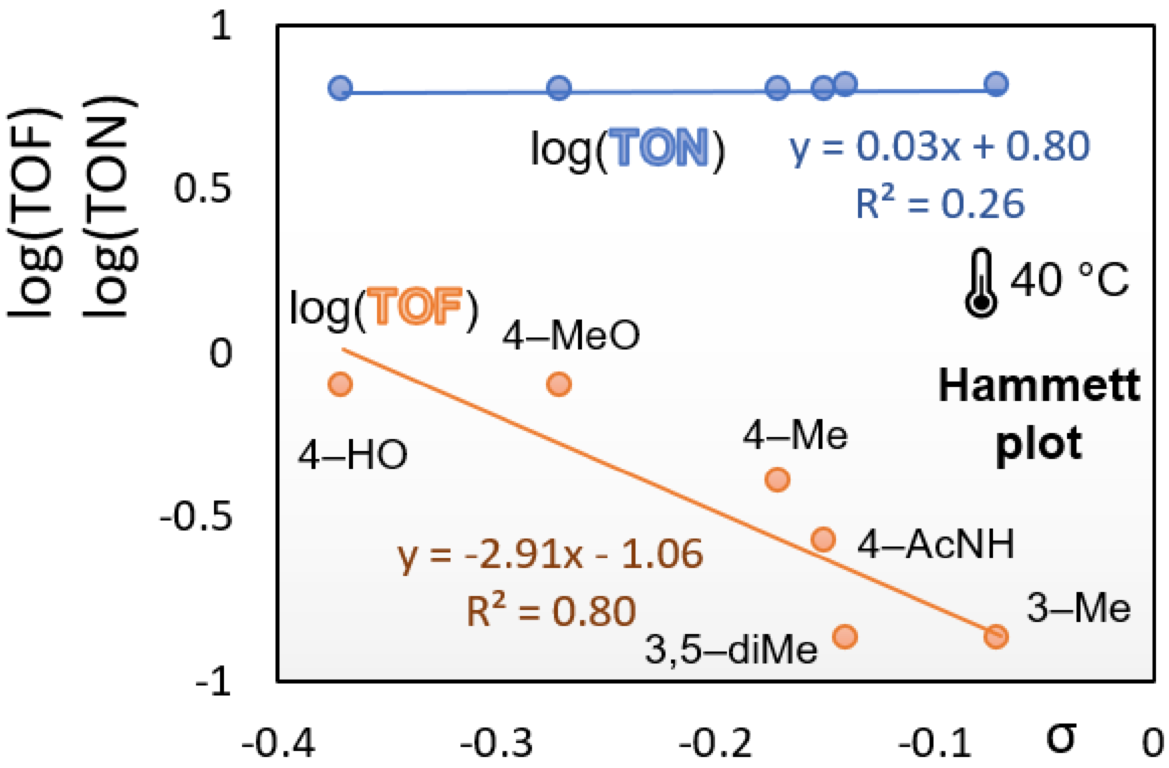

Figure S2 Hammett plot for catalysis with ***L5·Pd(TFA)$_2$***

S5-Continuous flow reactor optimization

*Table S2 Optimization of continuous flow synthesis of **P1j***

Flow (µl/min)	Pump pressure (bar)	**S1** concentration (mM)	Conversion (%)[a]
150	10	25	23
100	10	25	46
50	5	25	83
150	0.5	25	19
100	0.5	25	27
50	0.5	25	45
100	0.5	50	24
100	1.3	50	32
100	10	50	47
50	0.5	50	67
**10**	**0.5**	**50**	**97**
[a]Determined by ^{1}H NMR after equilibration (2 column volumes)			

S6-NMR, FT-IR spectra

^{1}H NMR (500 MHz, $CDCl_3$) of **L1**

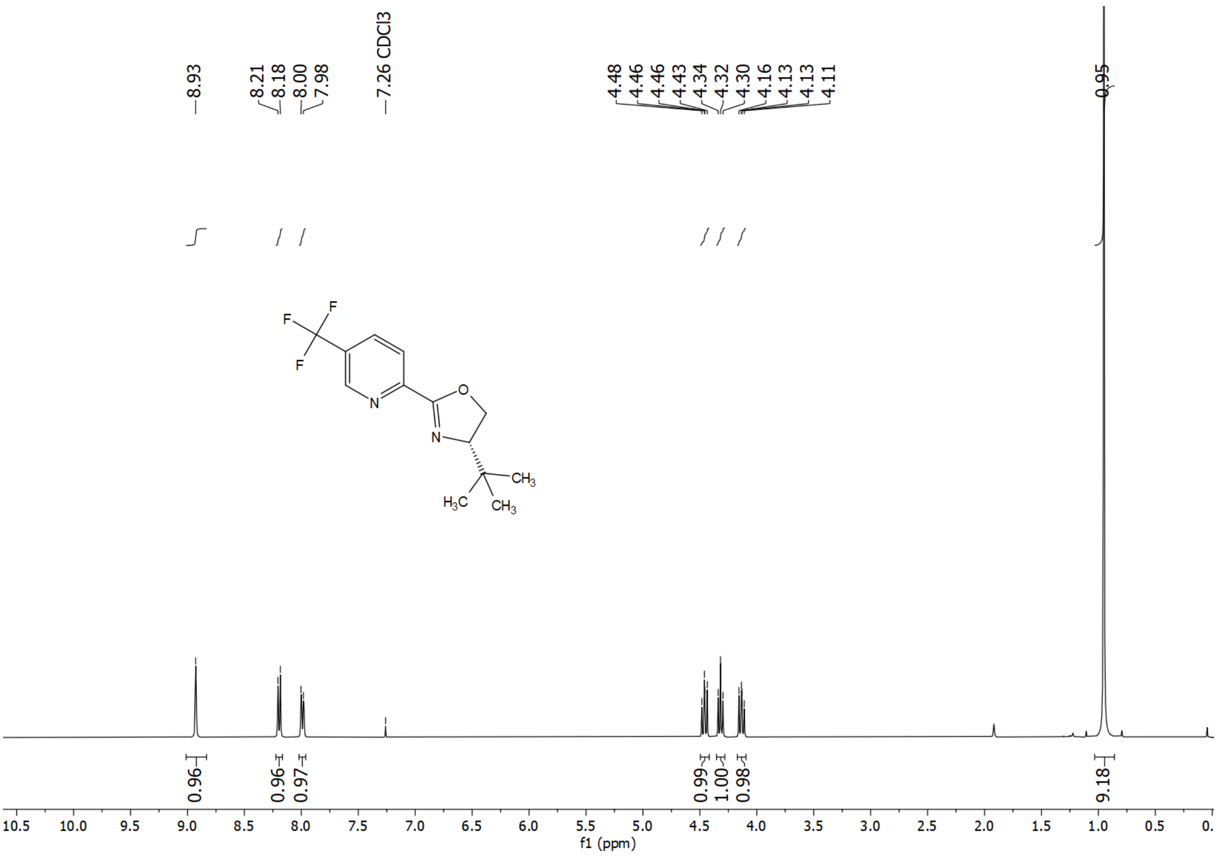

$^{13}C\{^{1}H\}$ NMR (126 MHz, $CDCl_3$) of **L1**

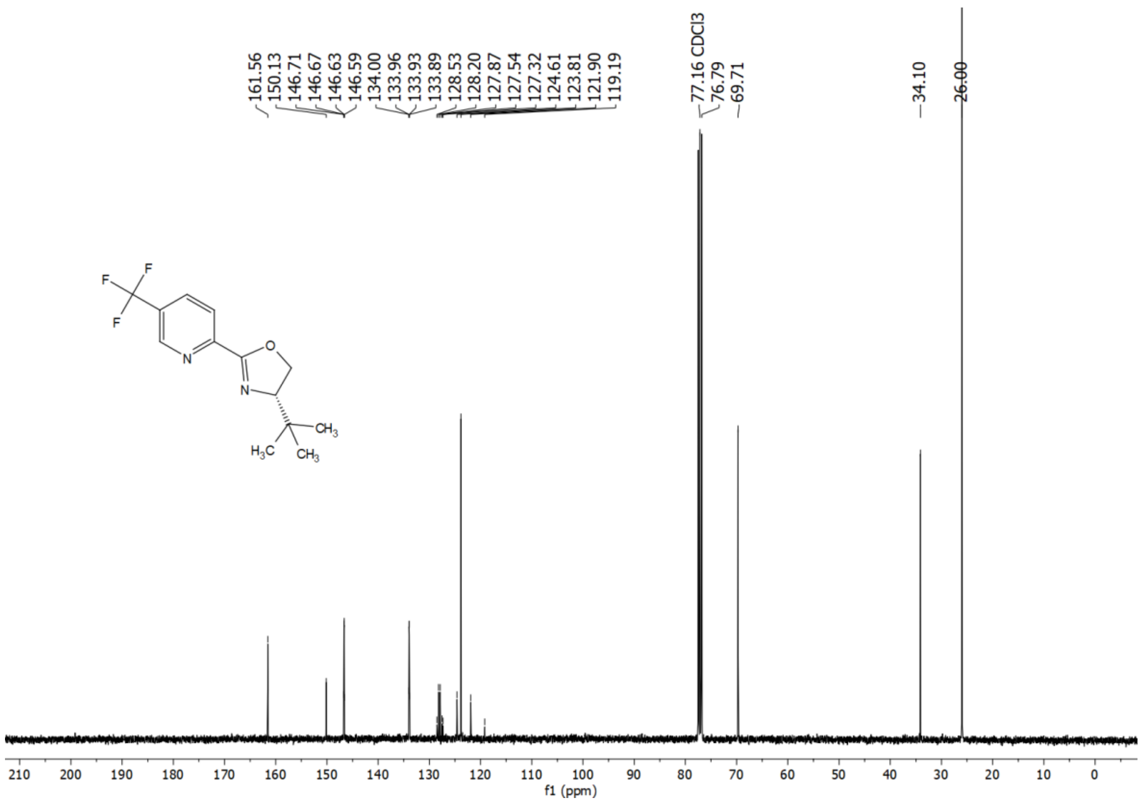

$^{19}F\{^{1}H\}$ NMR (376 MHz, CDCl3) of **L1**

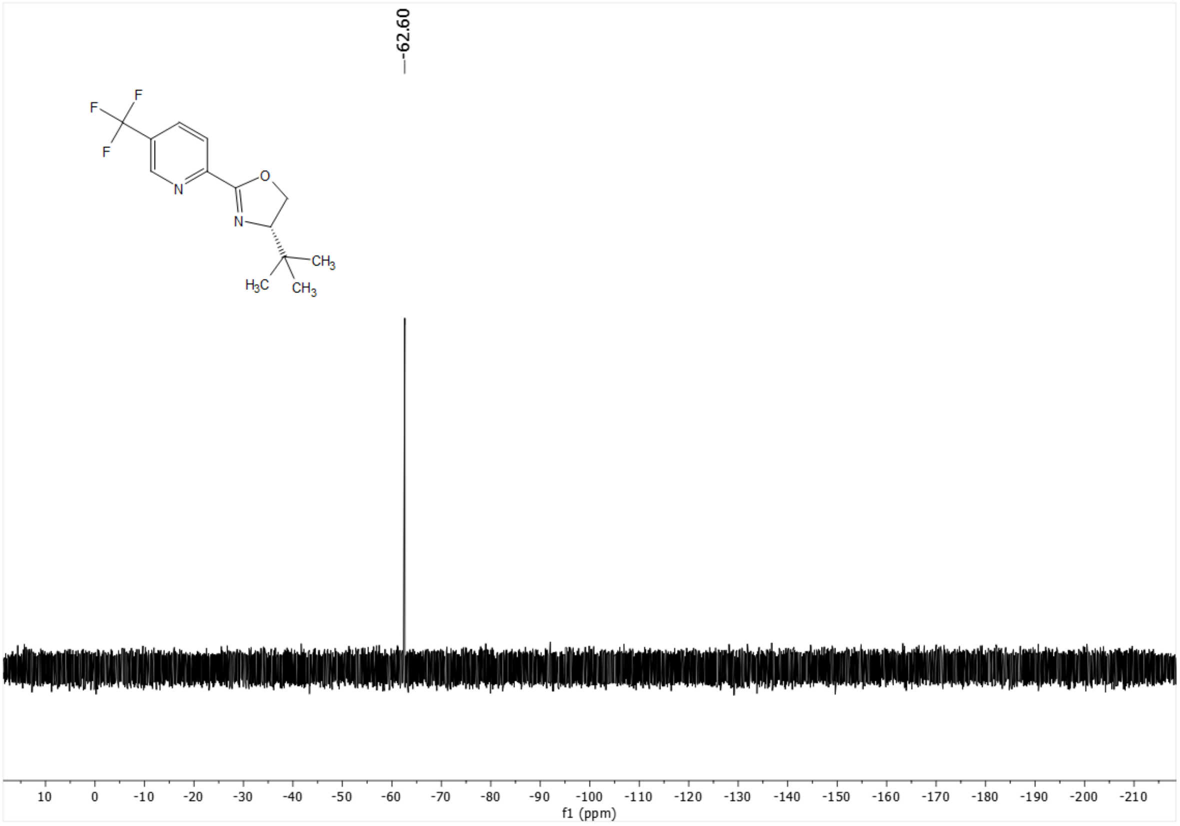

^{1}H NMR (500 MHz, $CDCl_3$) of **L3**

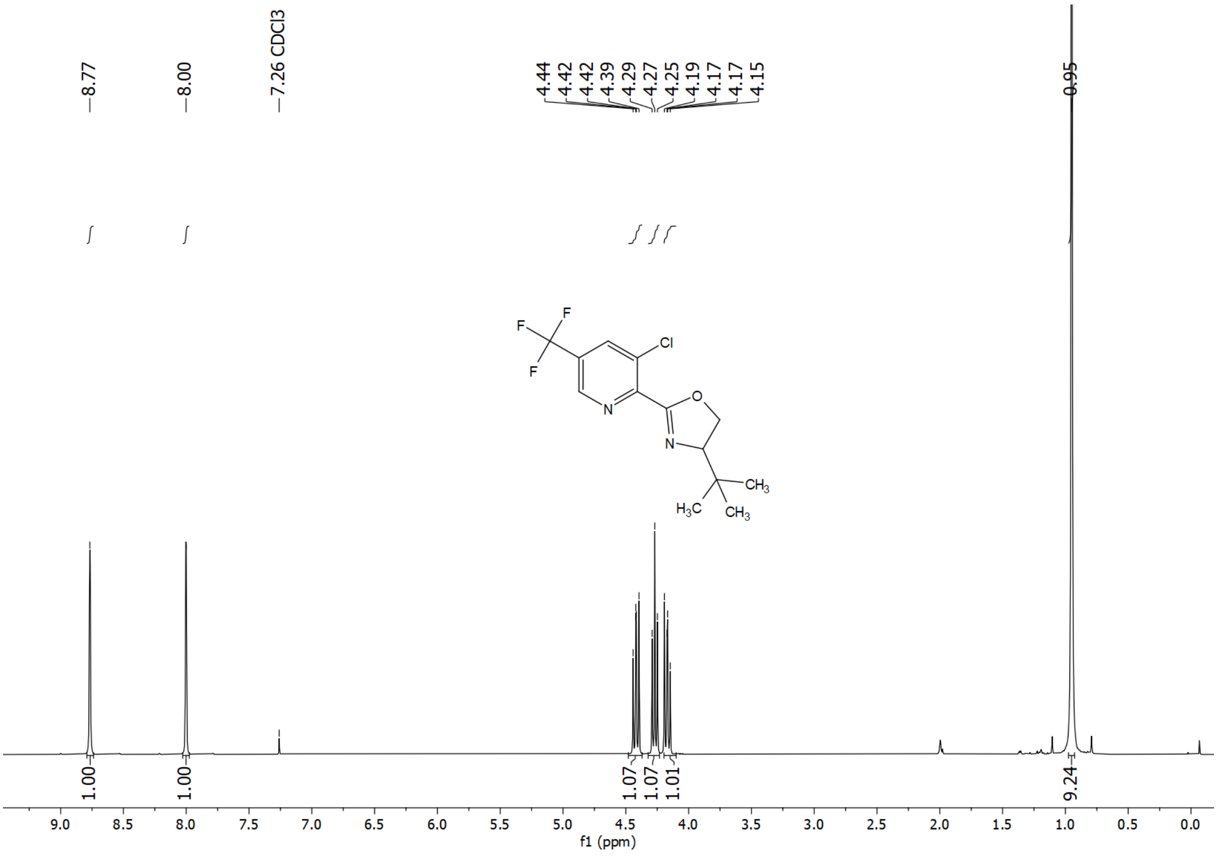

$^{13}C\{^1H\}$ NMR (126 MHz, $CDCl_3$) of **L3**

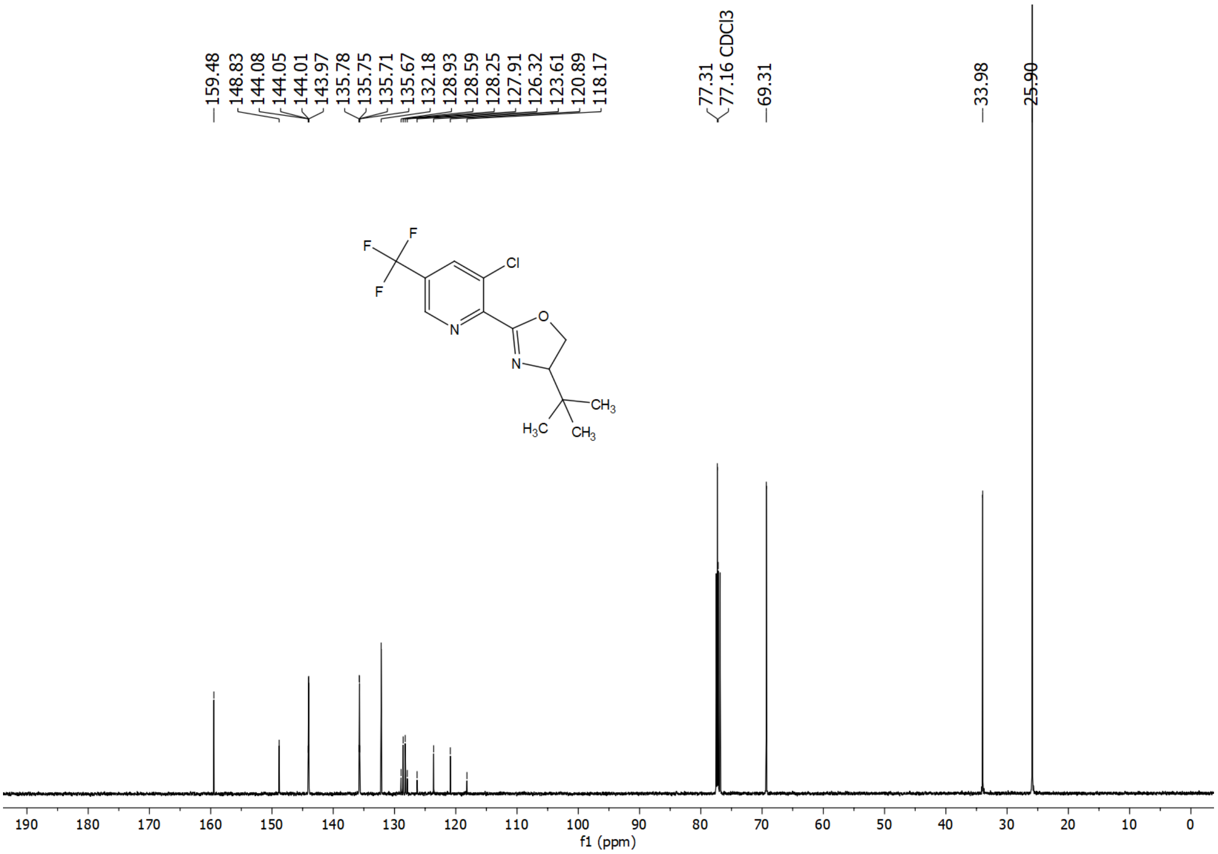

$^{19}F\{^1H\}$ NMR (376 MHz, CDCl3) of **L3**

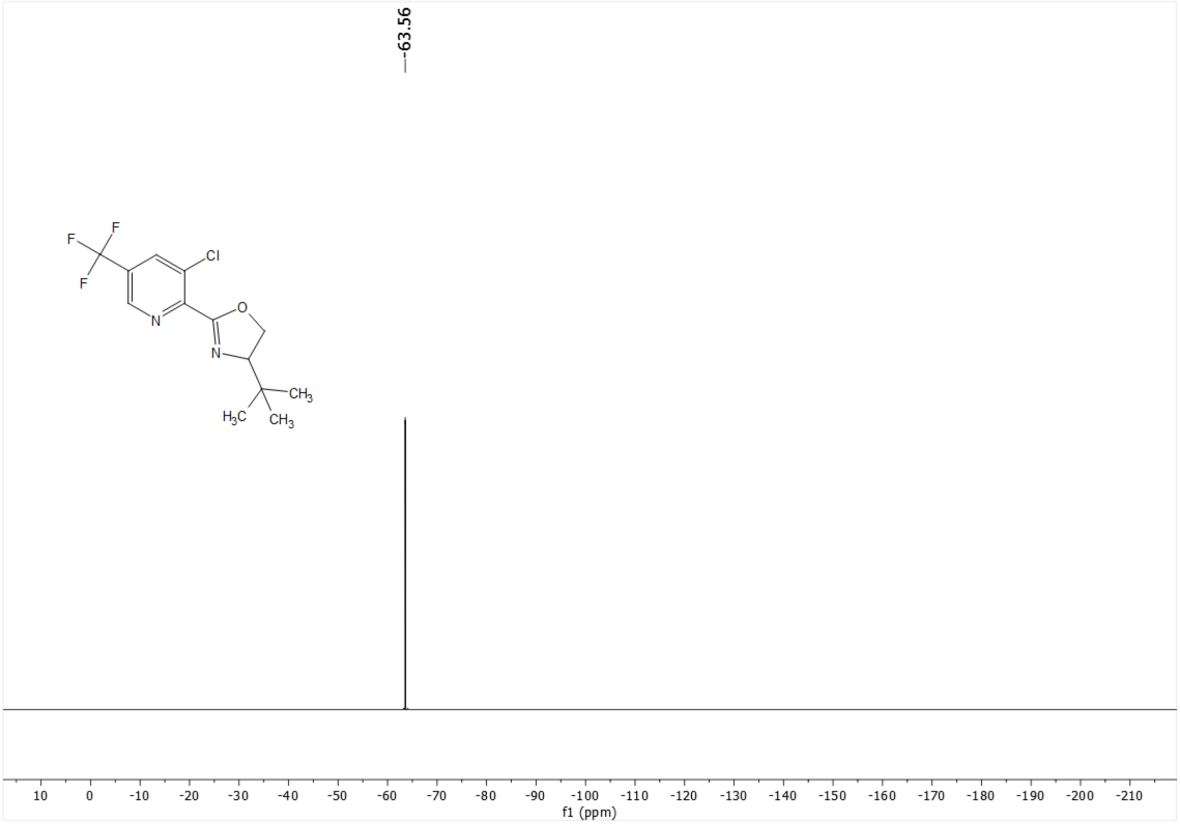

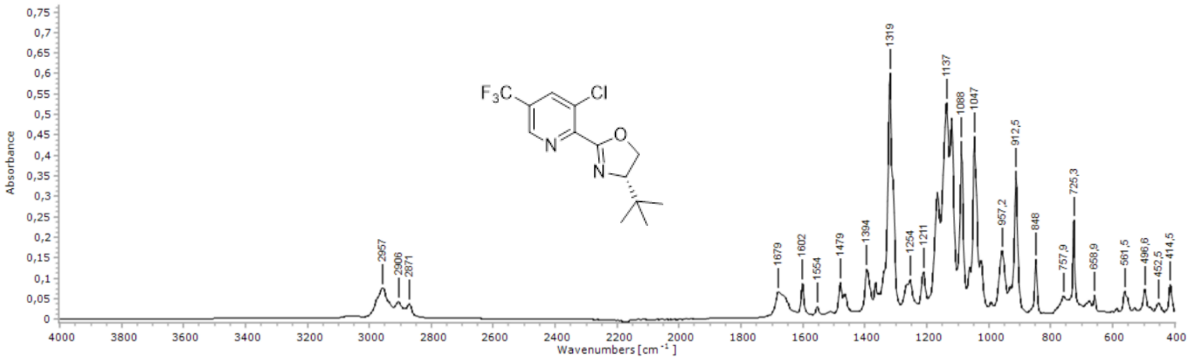

^{1}H NMR (500 MHz, $CDCl_3$) of **L4a**

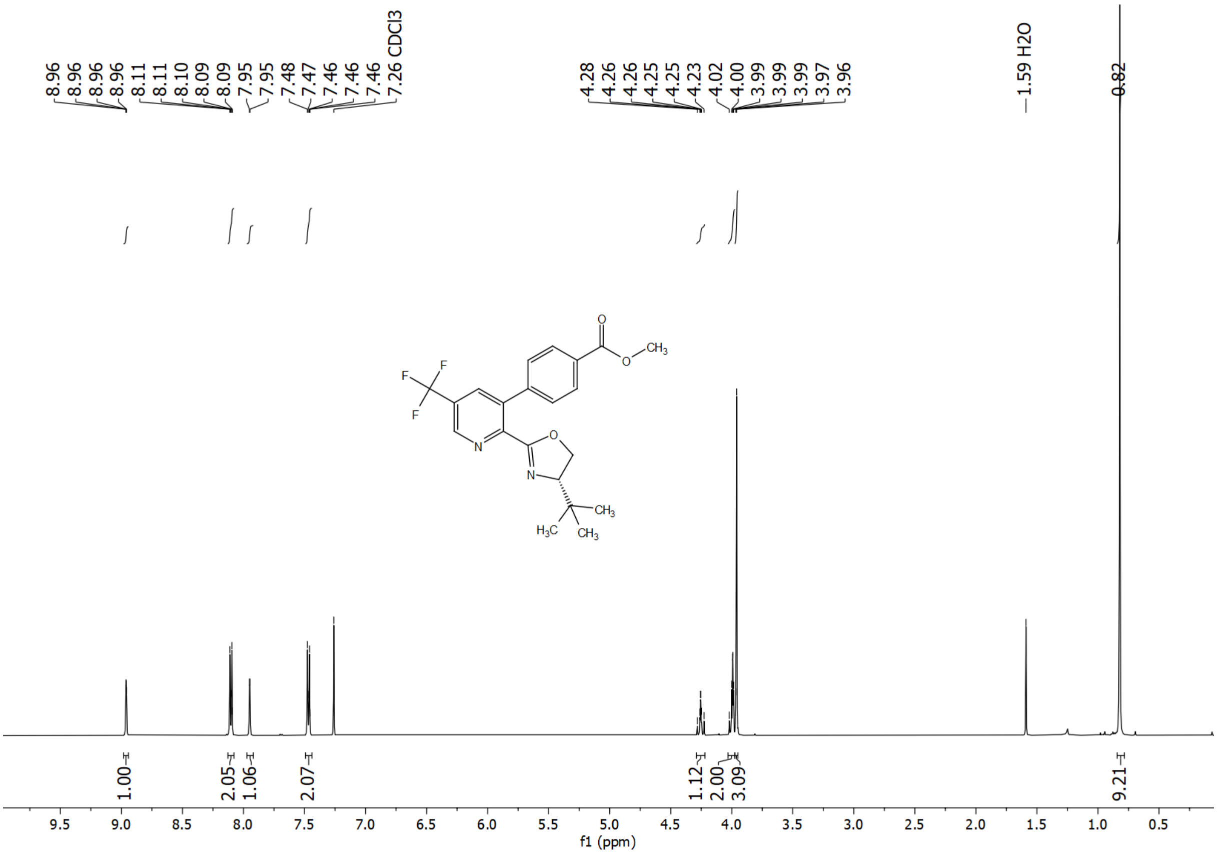

$^{13}C\{^{1}H\}$ NMR (126 MHz, $CDCl_3$) of **L4a**

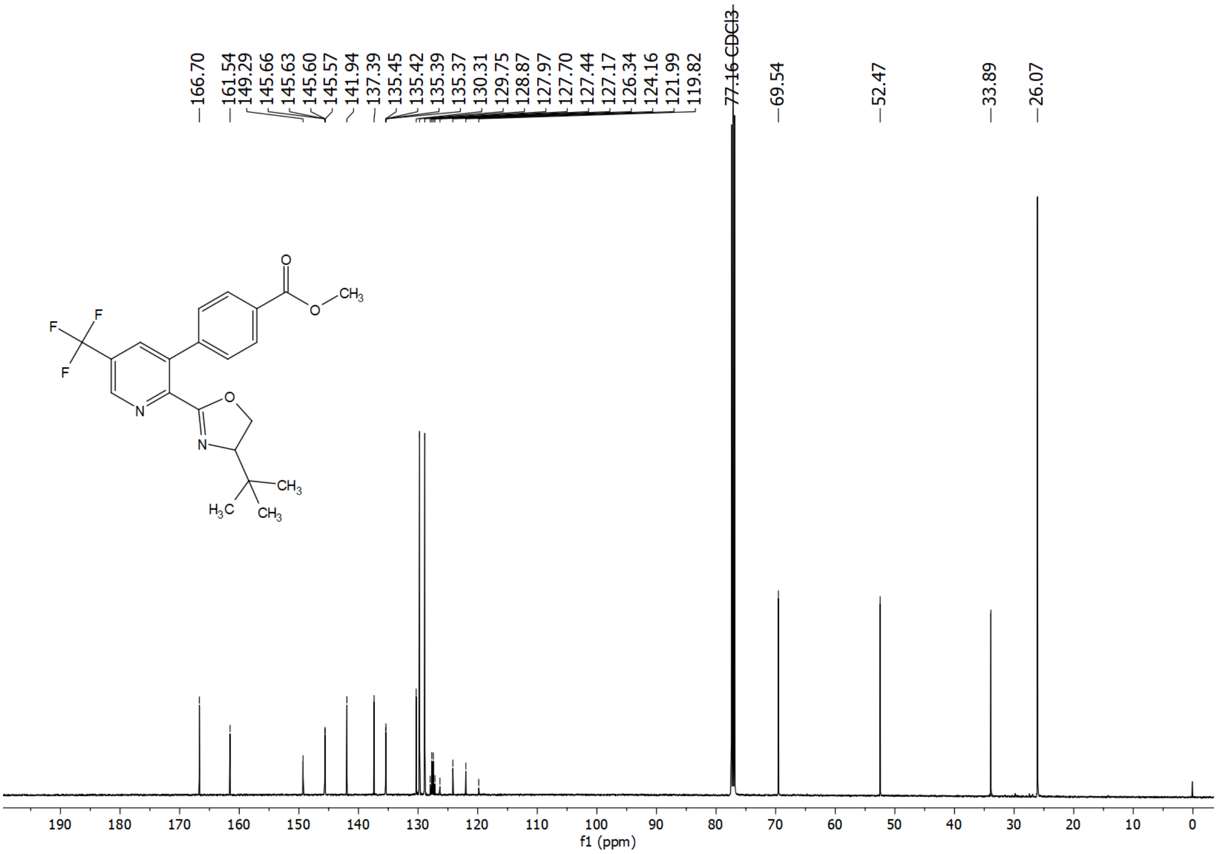

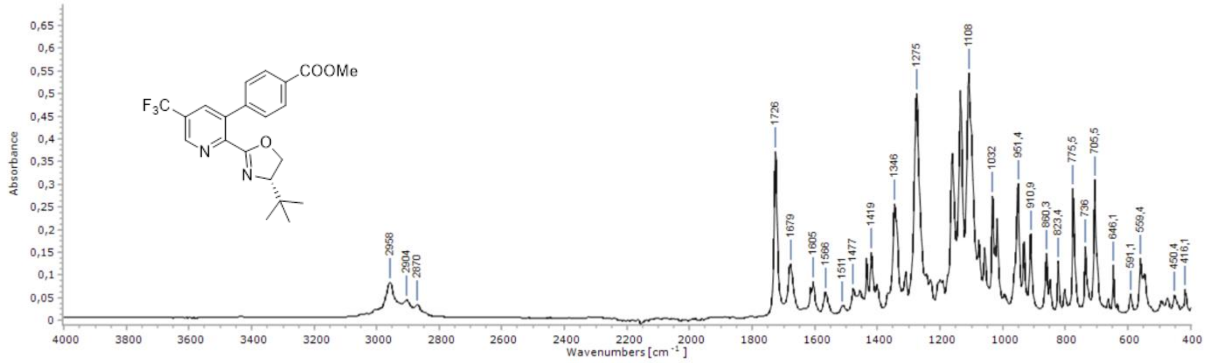

^{1}H NMR (500 MHz, DMSO-D6) of **L4b**

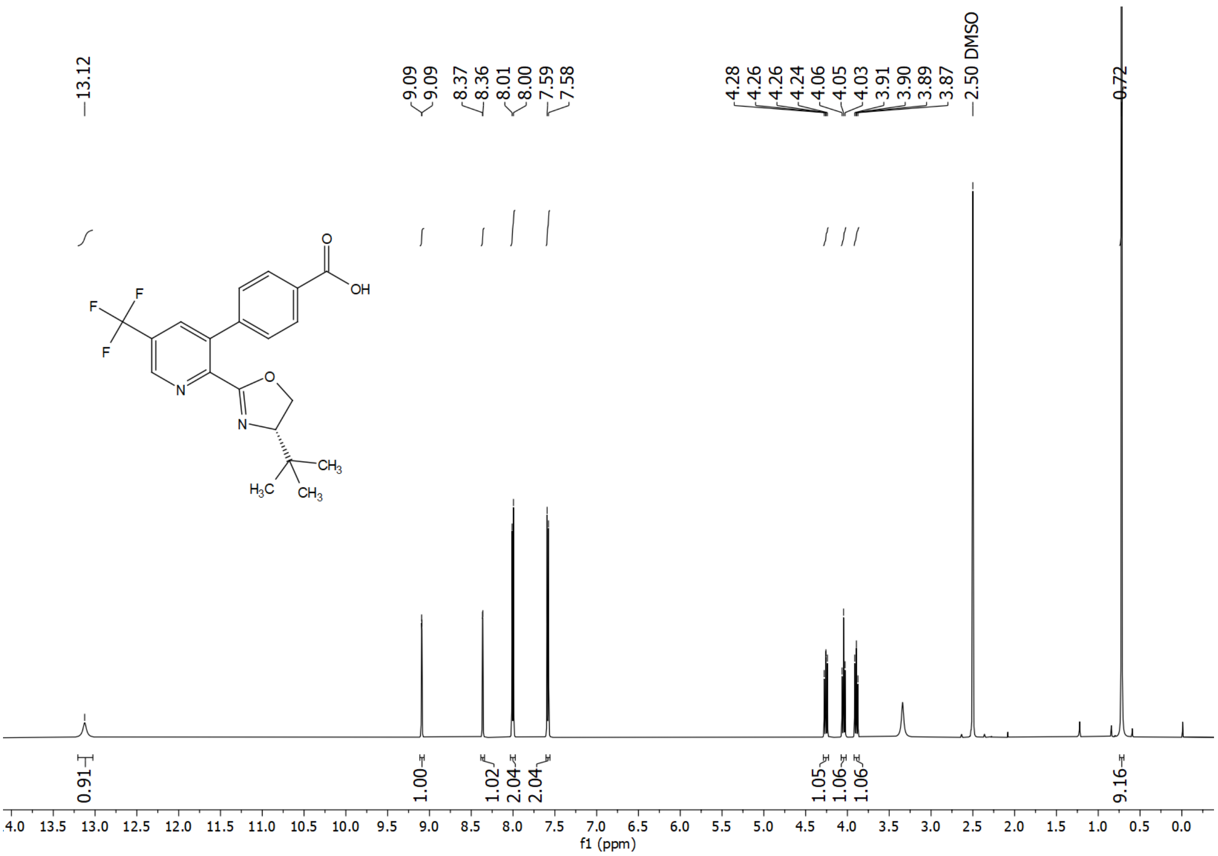

$^{13}C\{^{1}H\}$ NMR (126 MHz, DMSO-D6) of **L4b**

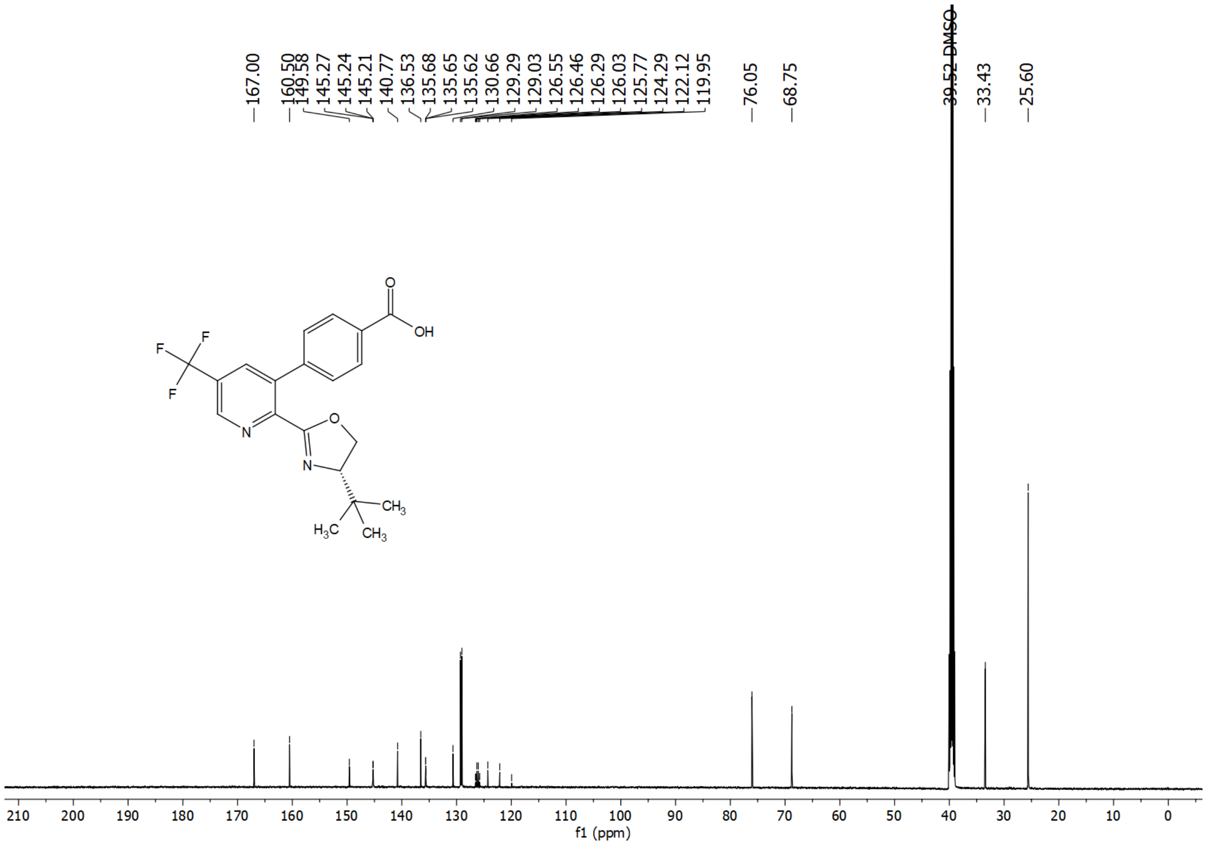

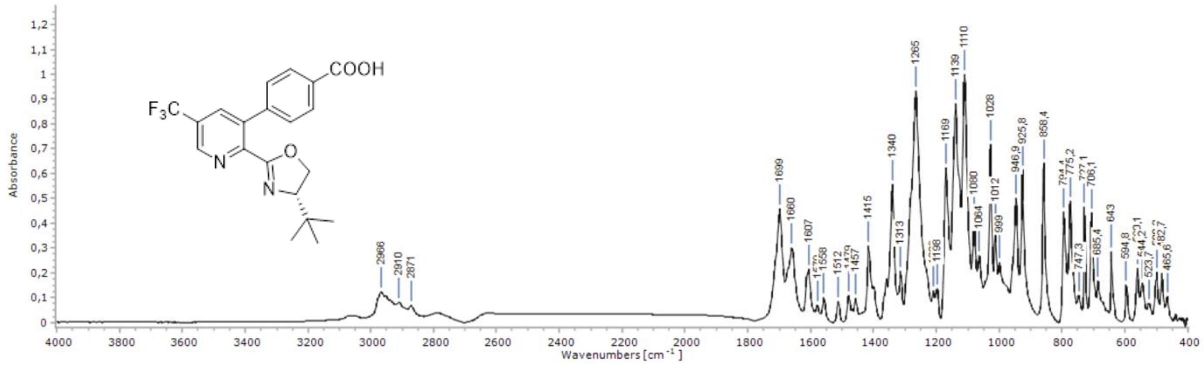

$^{19}F\{^{1}H\}$ NMR (376 MHz, $CDCl_3$) of **L4a**

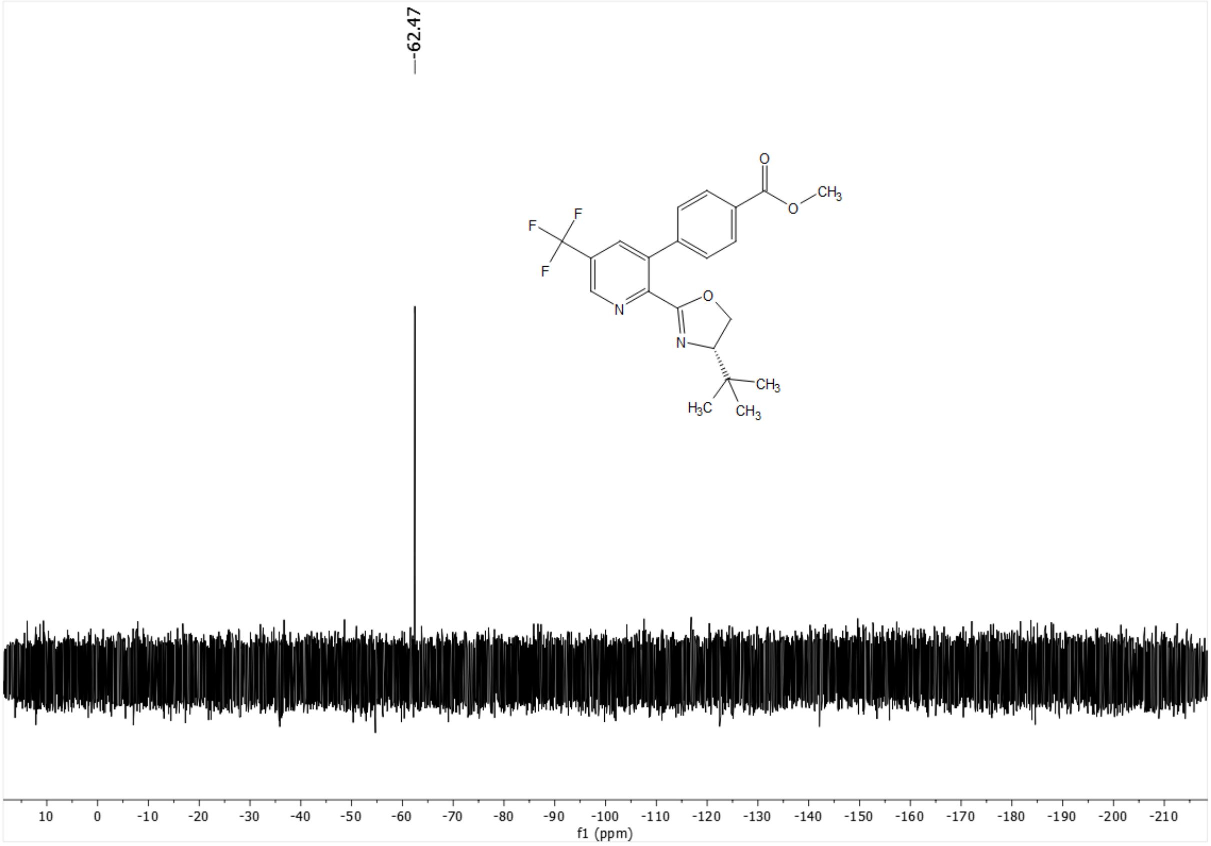

$^{19}F\{^{1}H\}$ NMR (376 MHz, DMSO) of **L4b**

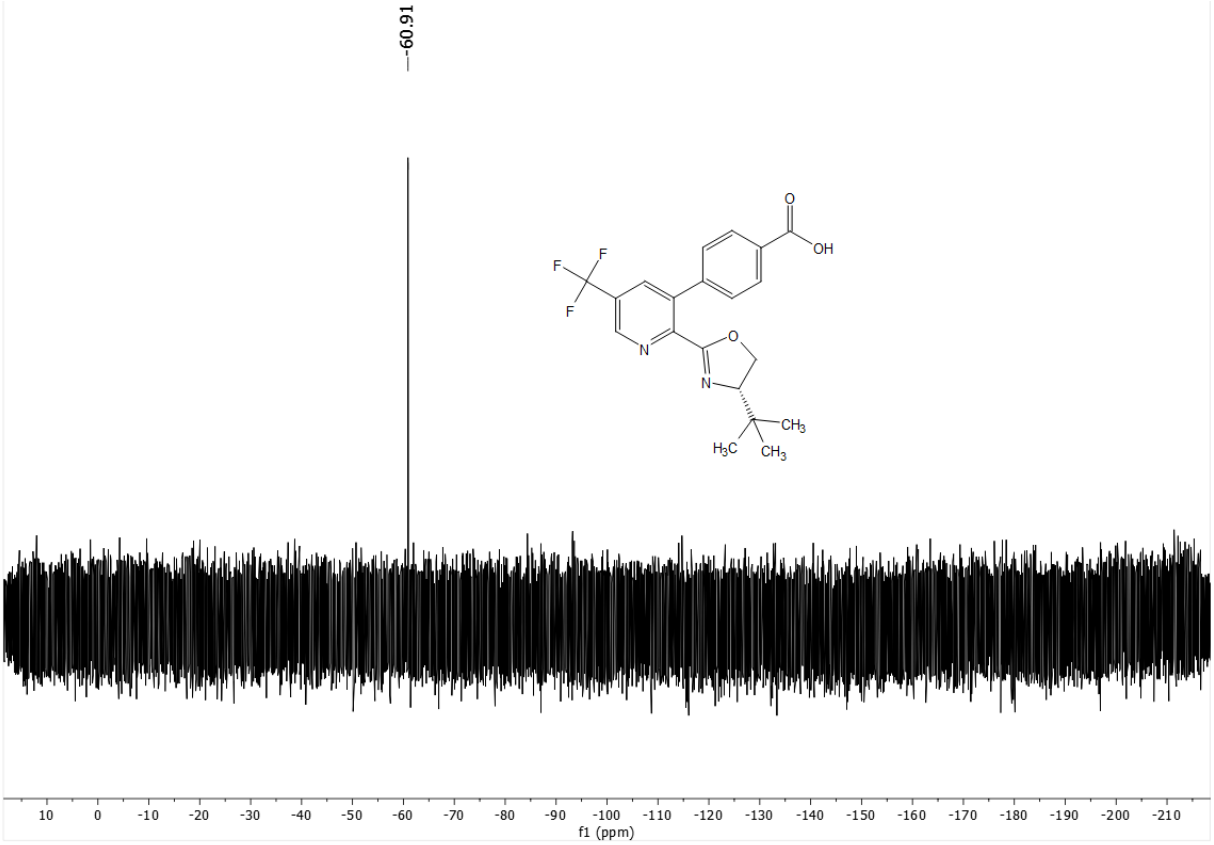

Gel-phase ^{1}H NMR (500 MHz, $CDCl_3$) of **L5**

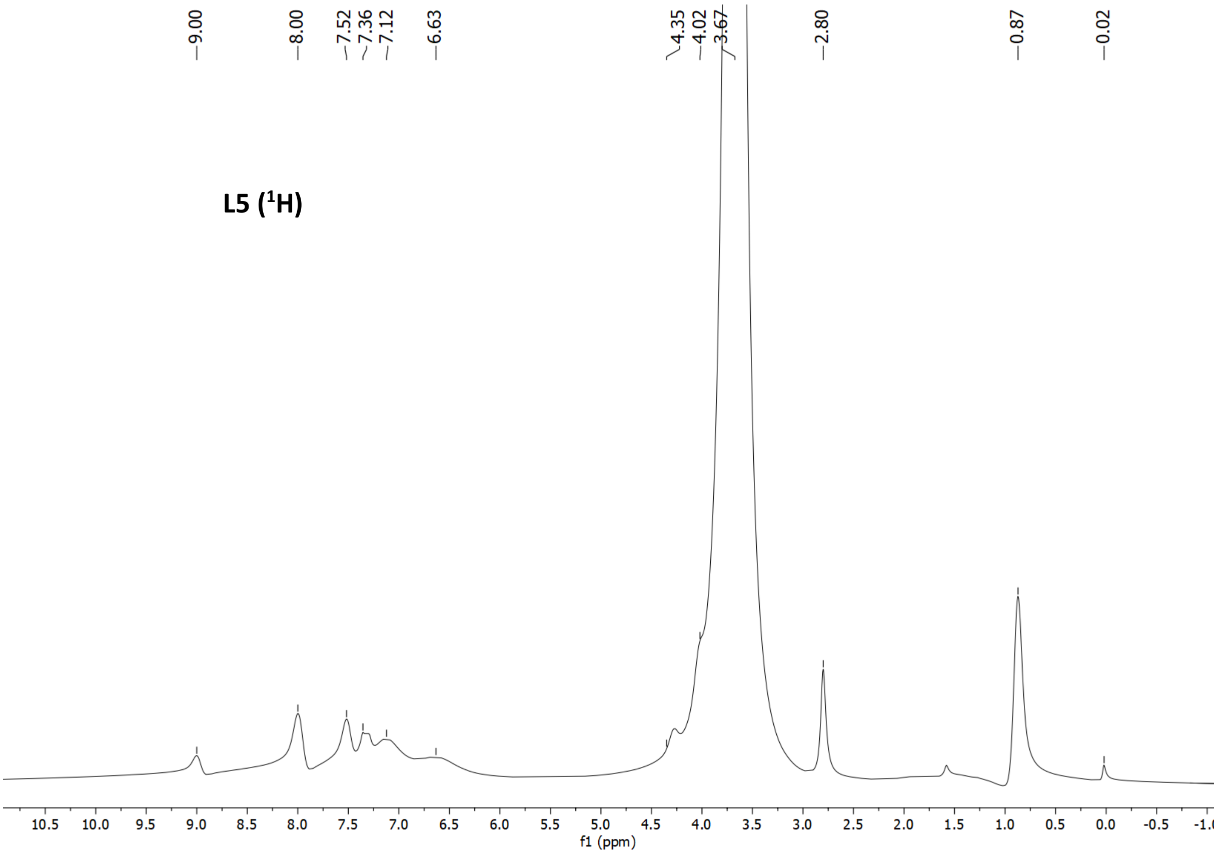

Gel phase $^{13}C\{^{1}H\}$ NMR (126 MHz, $CDCl_3$) of **L5**

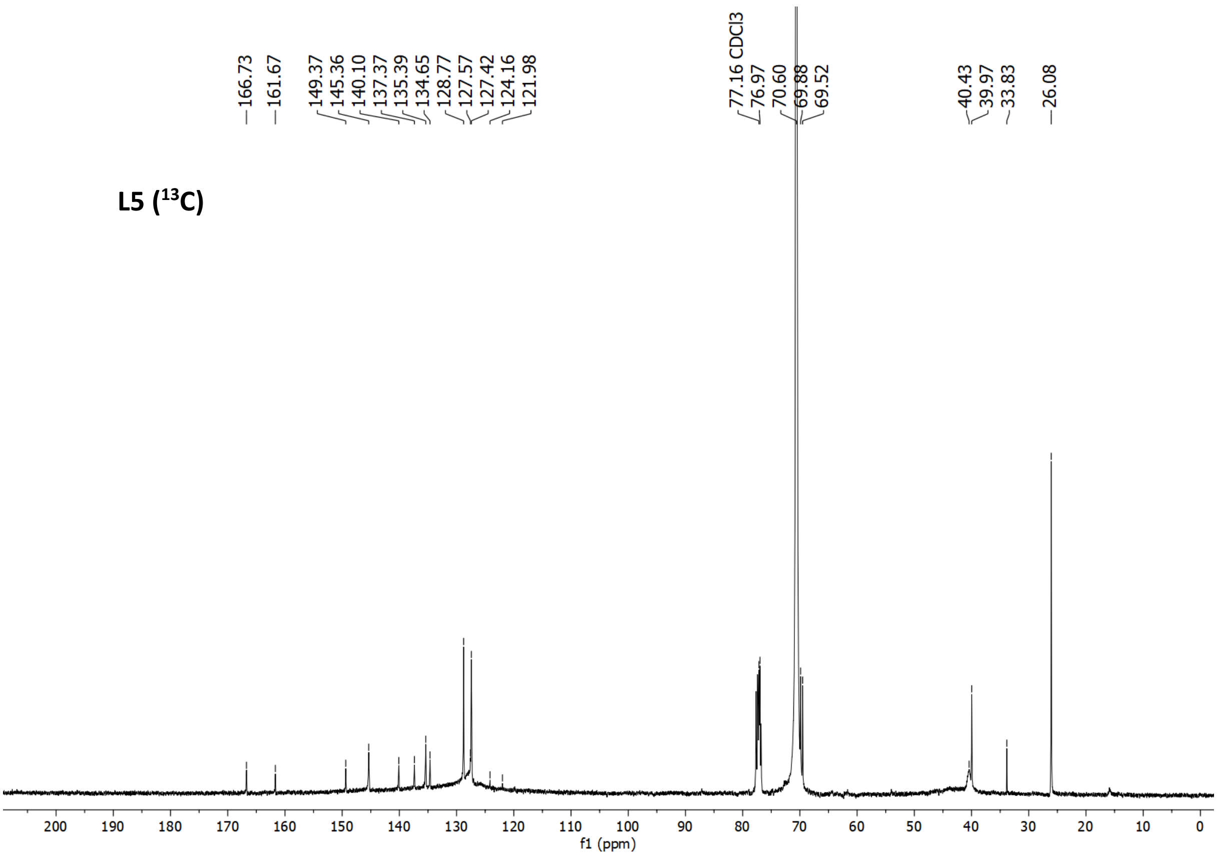

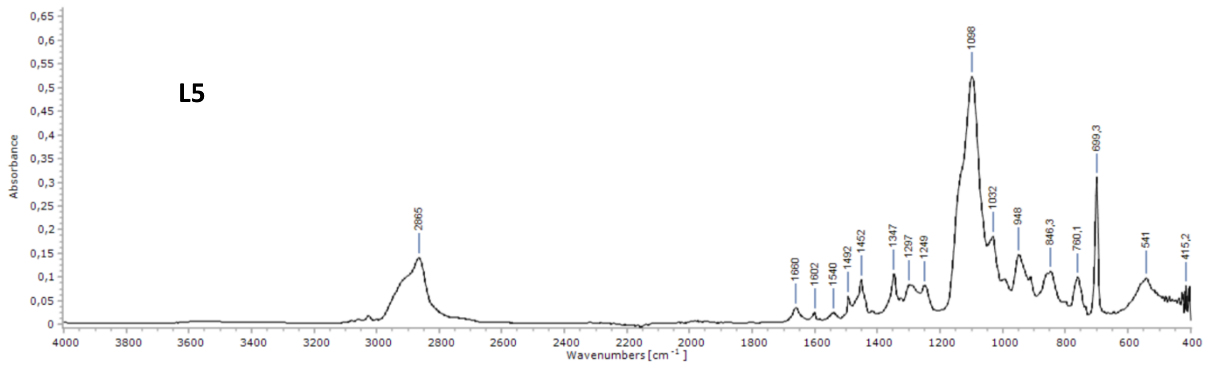

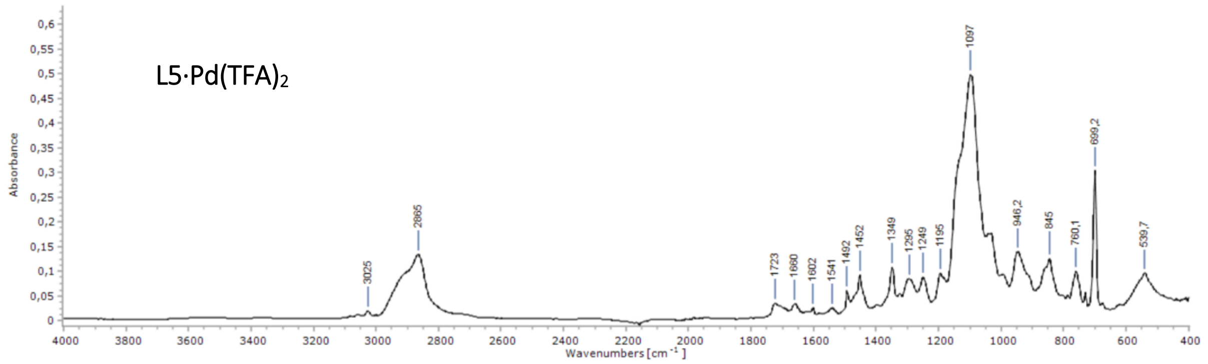

^{1}H NMR (500 MHz, $CDCl_3$) of **S1**

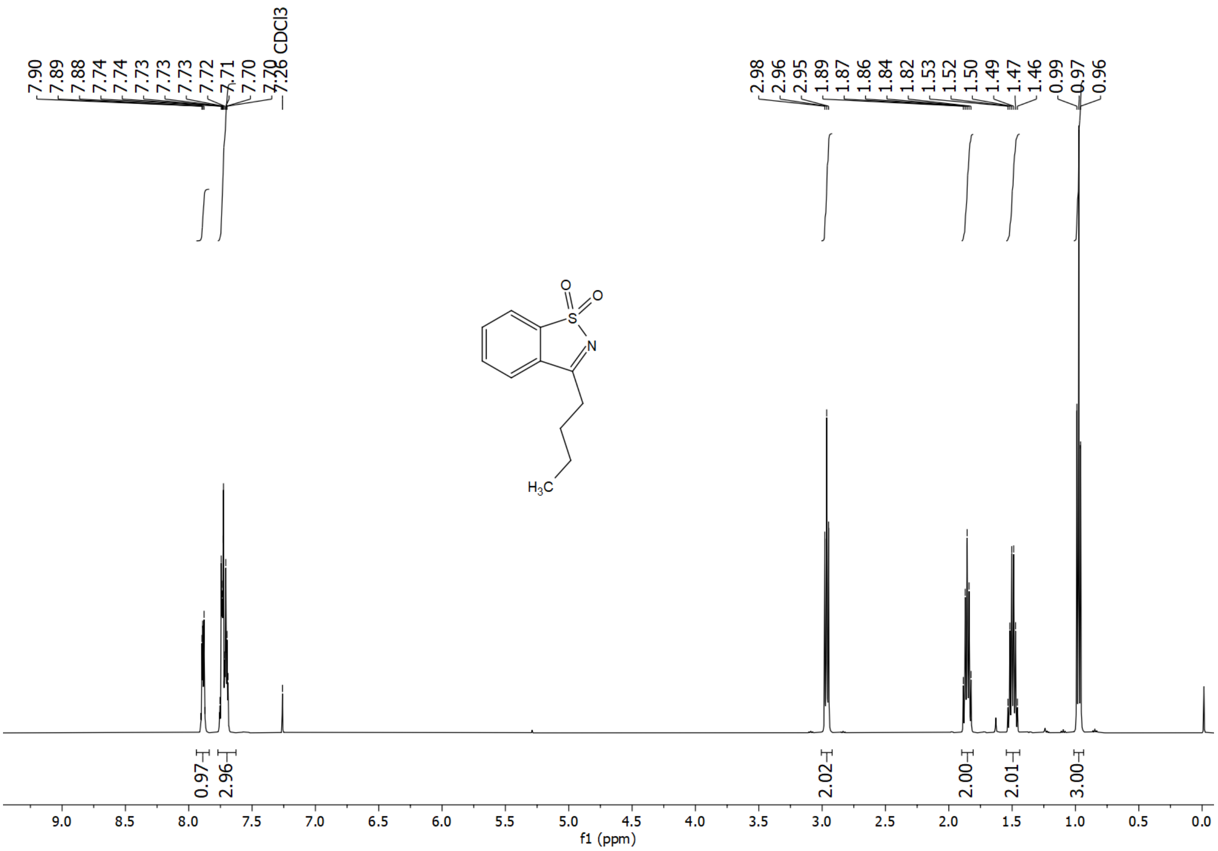

^{13}C{^{1}H} NMR (126 MHz, $CDCl_3$) of **S1**

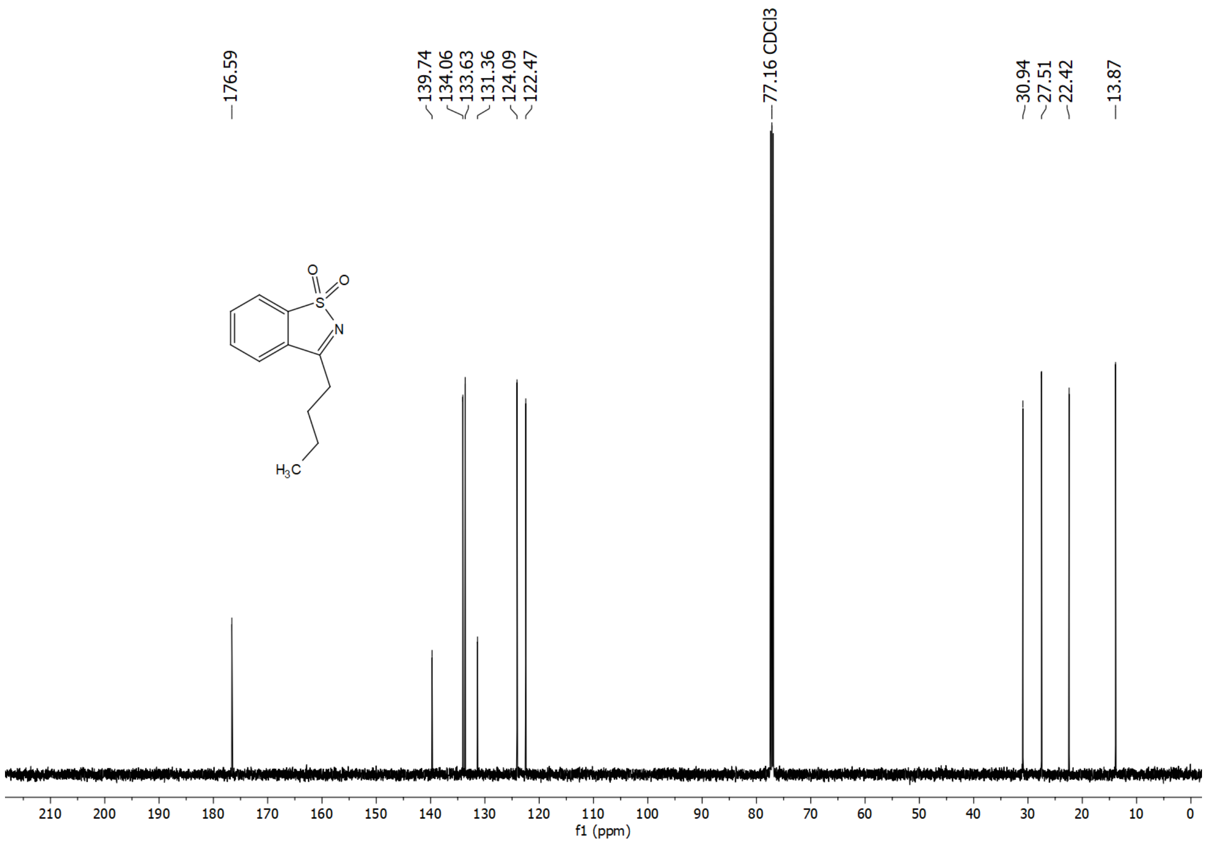

^{1}H NMR (500 MHz, $CDCl_3$) of **S2**

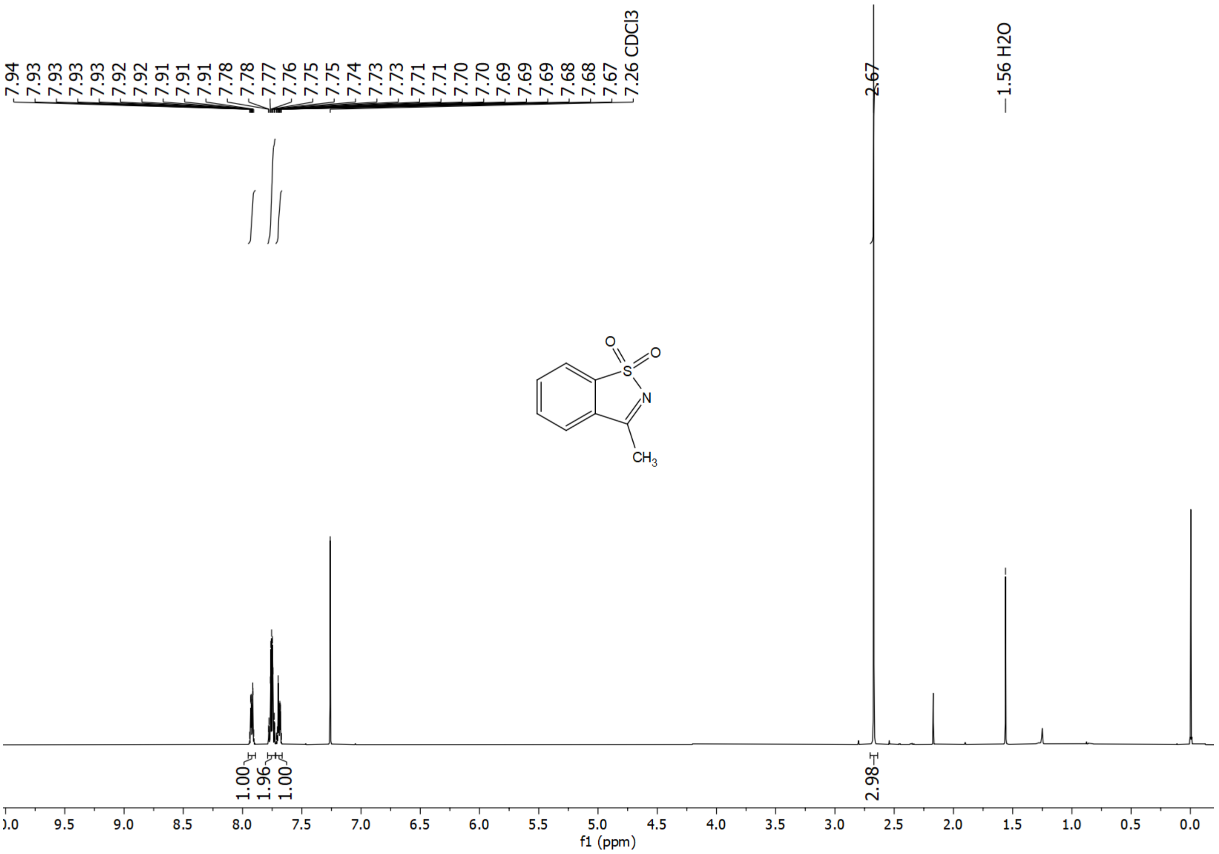

^{13}C{^{1}H} NMR (126 MHz, $CDCl_3$) of **S2**

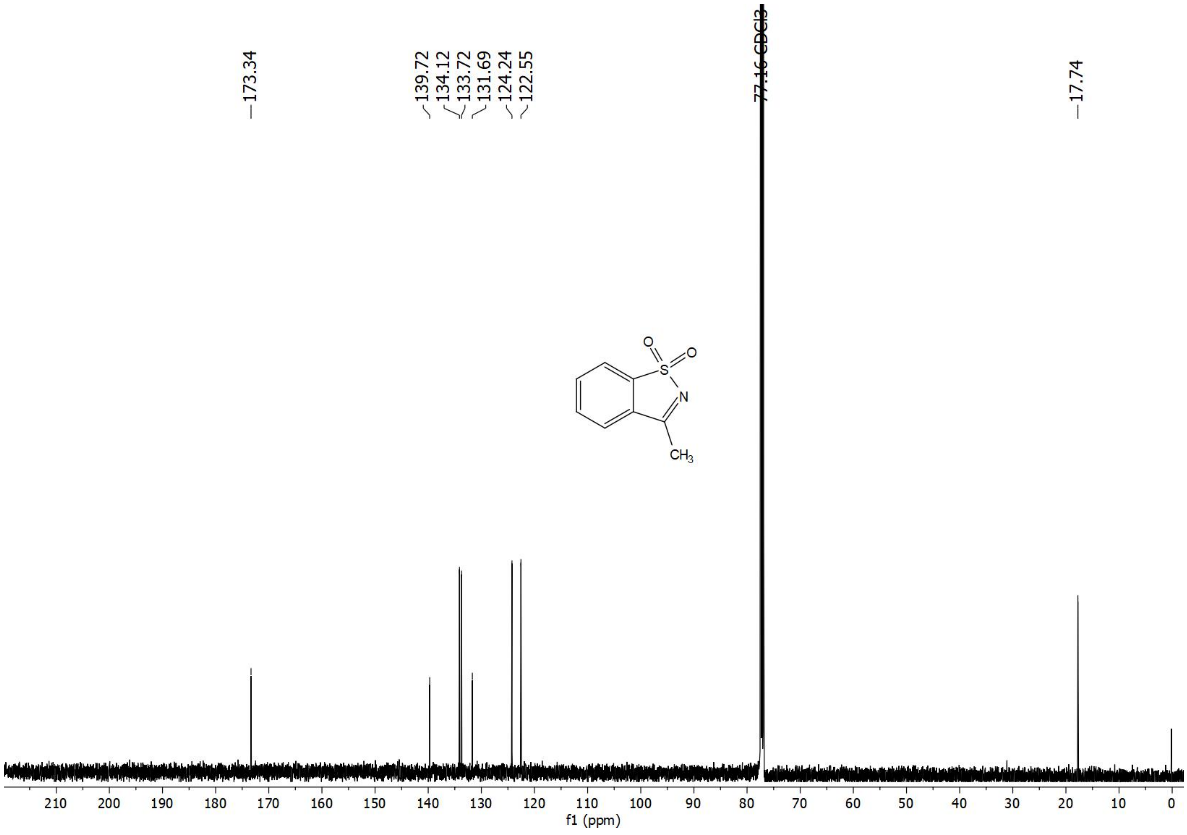

^{1}H NMR (500 MHz, $CDCl_3$) of **S3**

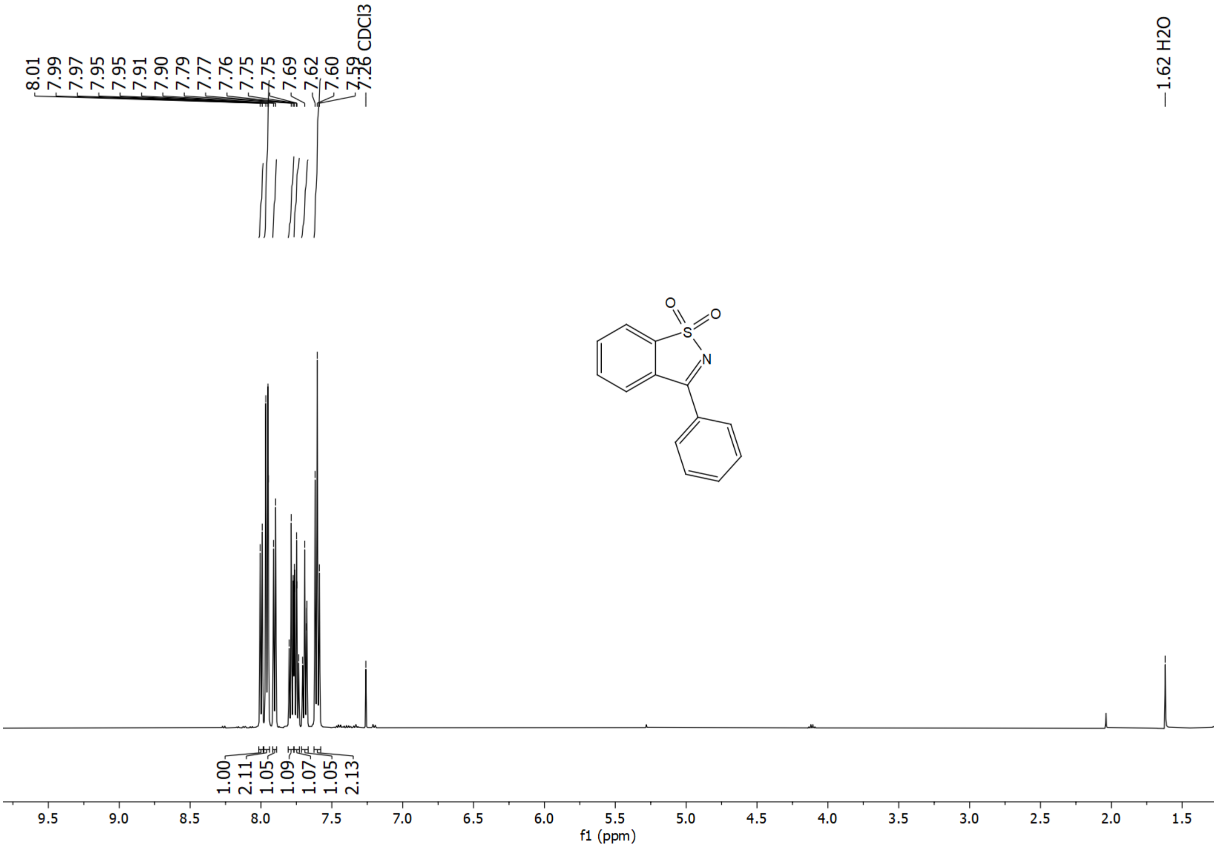

^{13}C{^{1}H} NMR (126 MHz, $CDCl_3$) of **S3**

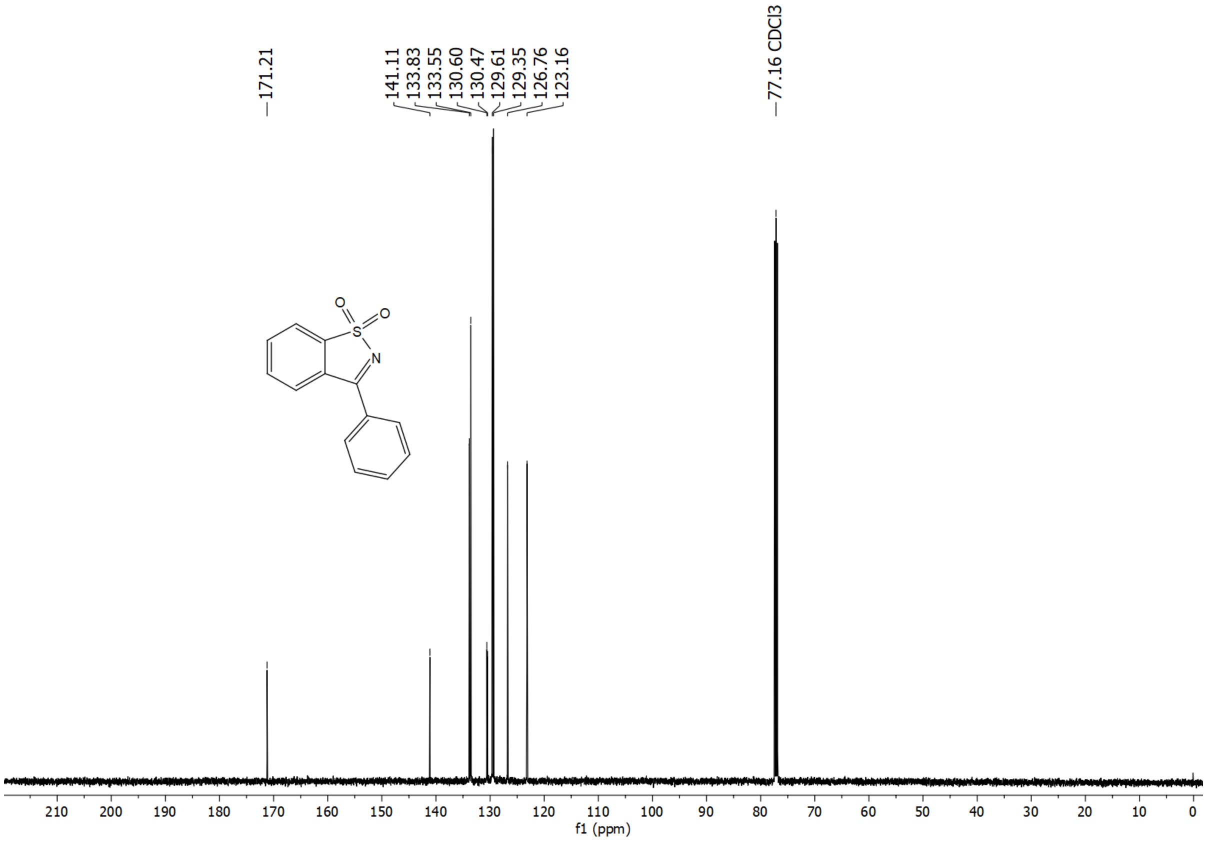

^{1}H NMR (500 MHz, $CDCl_3$) of **S4**

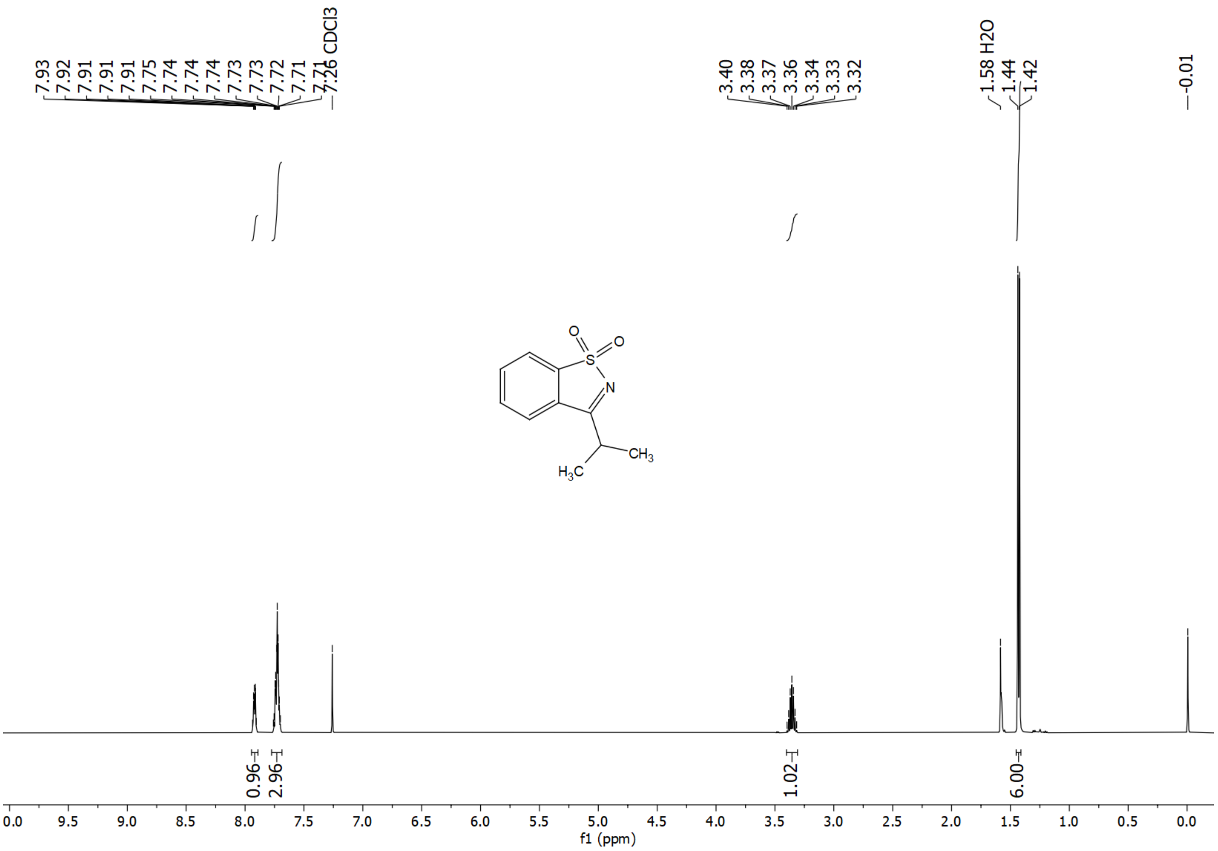

$^{13}C\{^{1}H\}$ NMR (126 MHz, $CDCl_3$) of **S4**

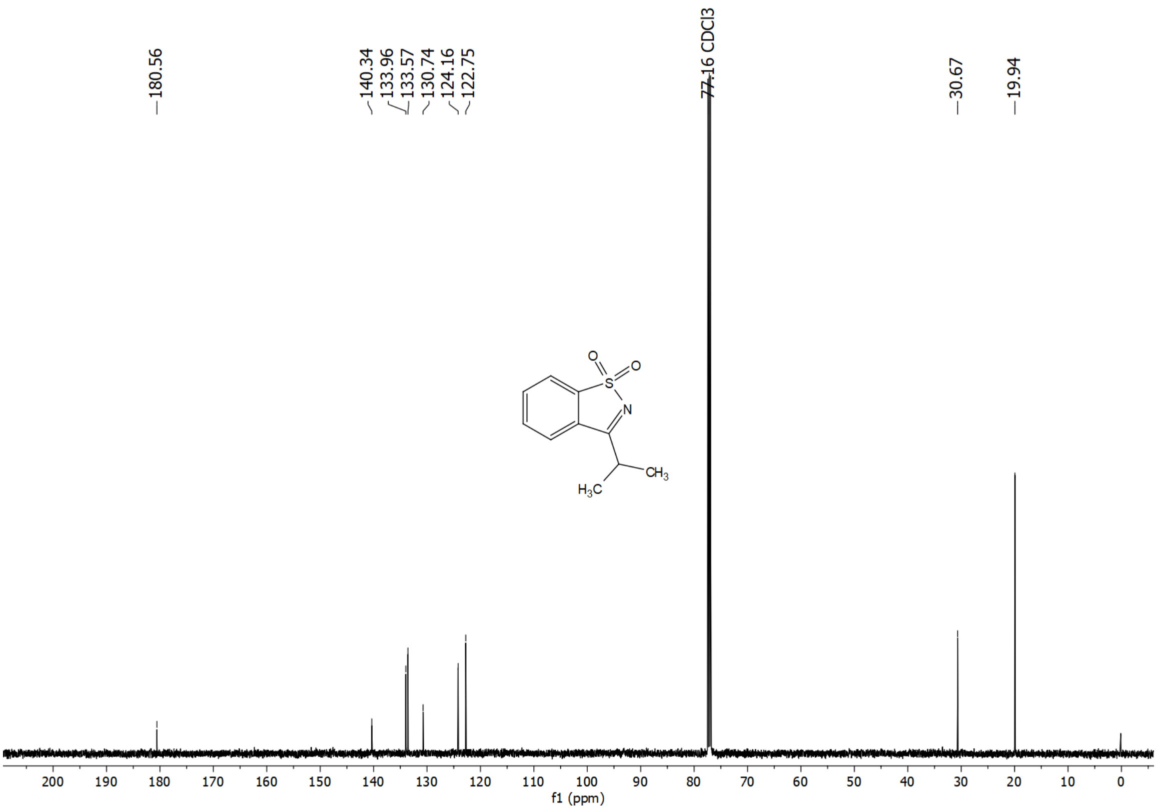

^{1}H NMR (500 MHz, $CDCl_3$) of **P1a**

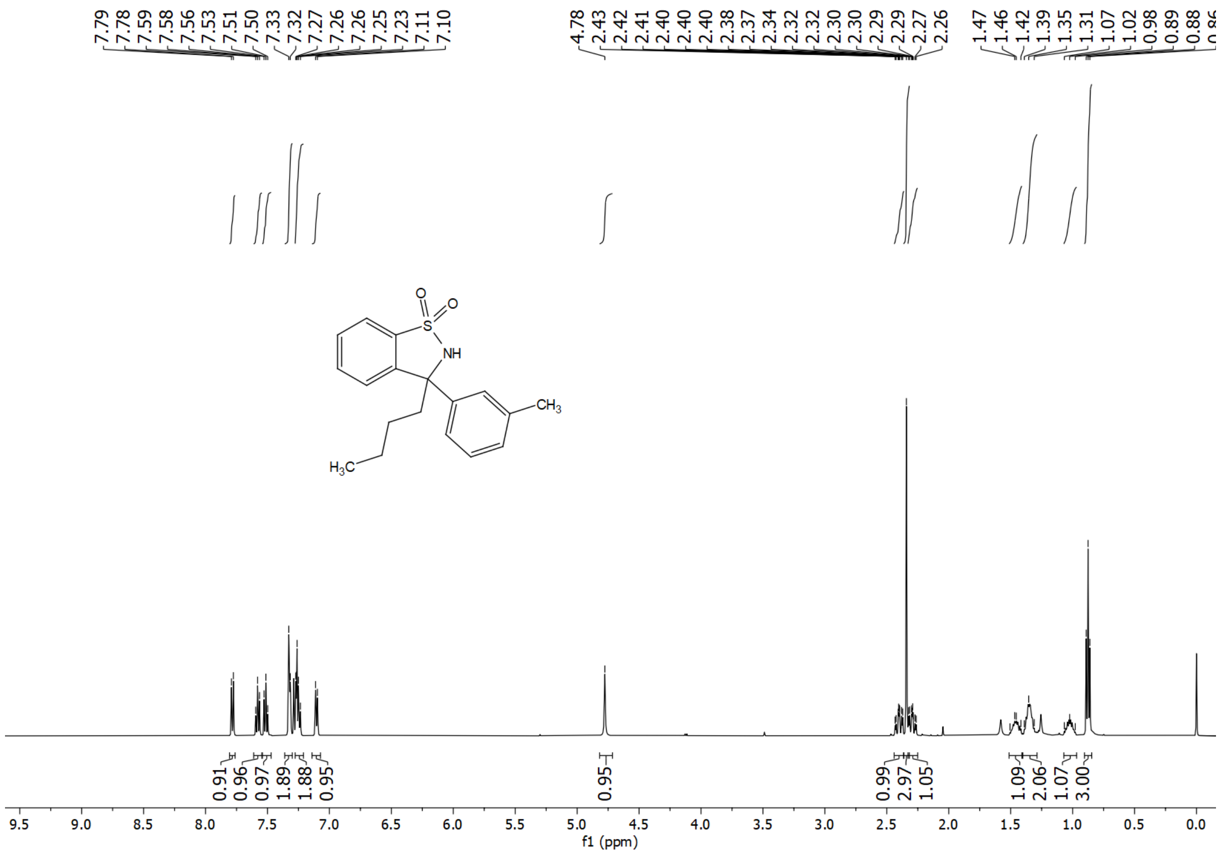

$^{13}C\{^{1}H\}$ NMR (126 MHz, $CDCl_3$) of **P1a**

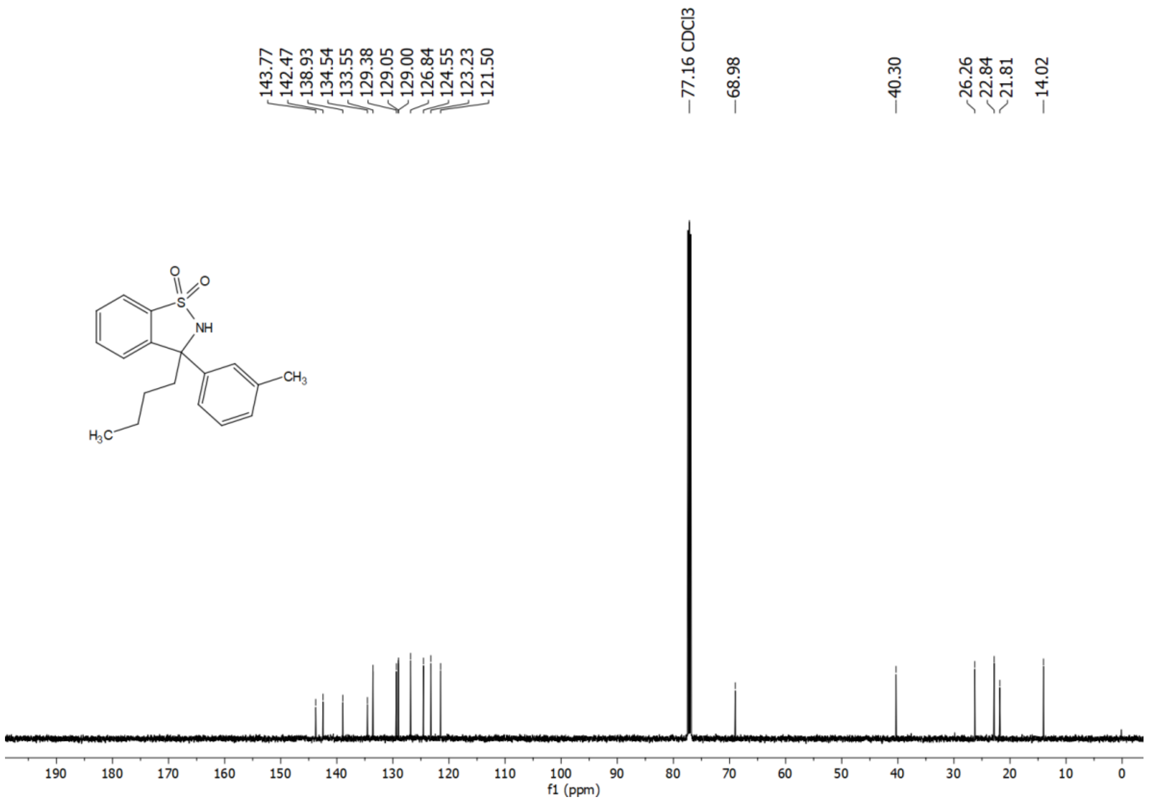

^{1}H NMR (500 MHz, $CDCl_3$) of **P1b**

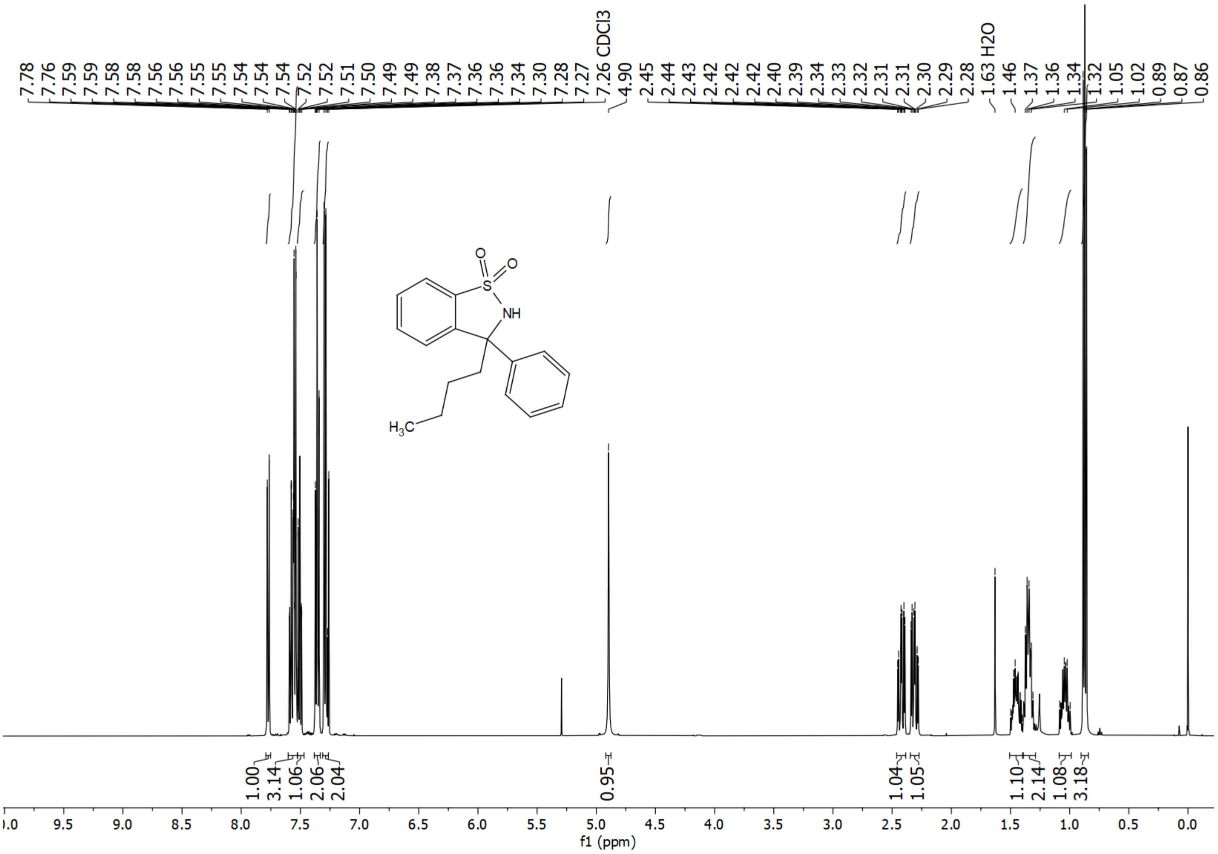

^{13}C{^{1}H} NMR (126 MHz, $CDCl_3$) of **P1b**

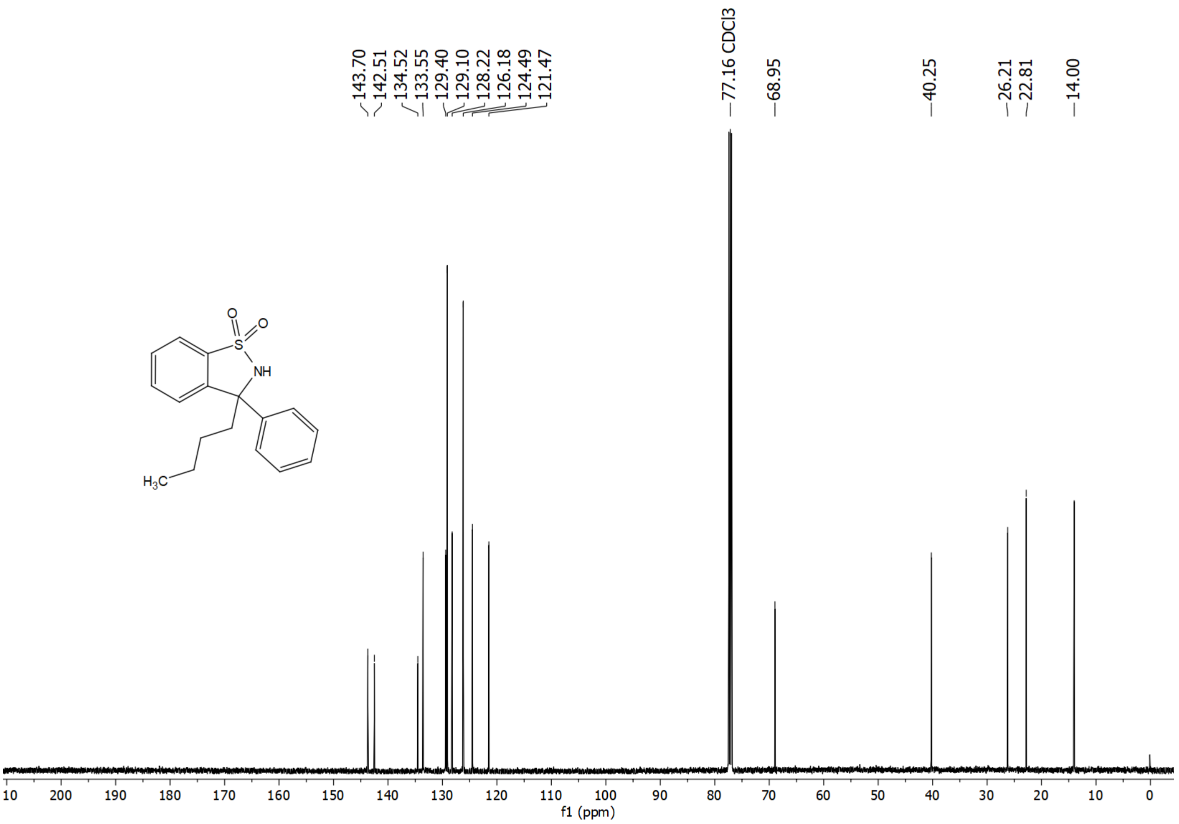

^{1}H NMR (500 MHz, $CDCl_3$) of **P1c**

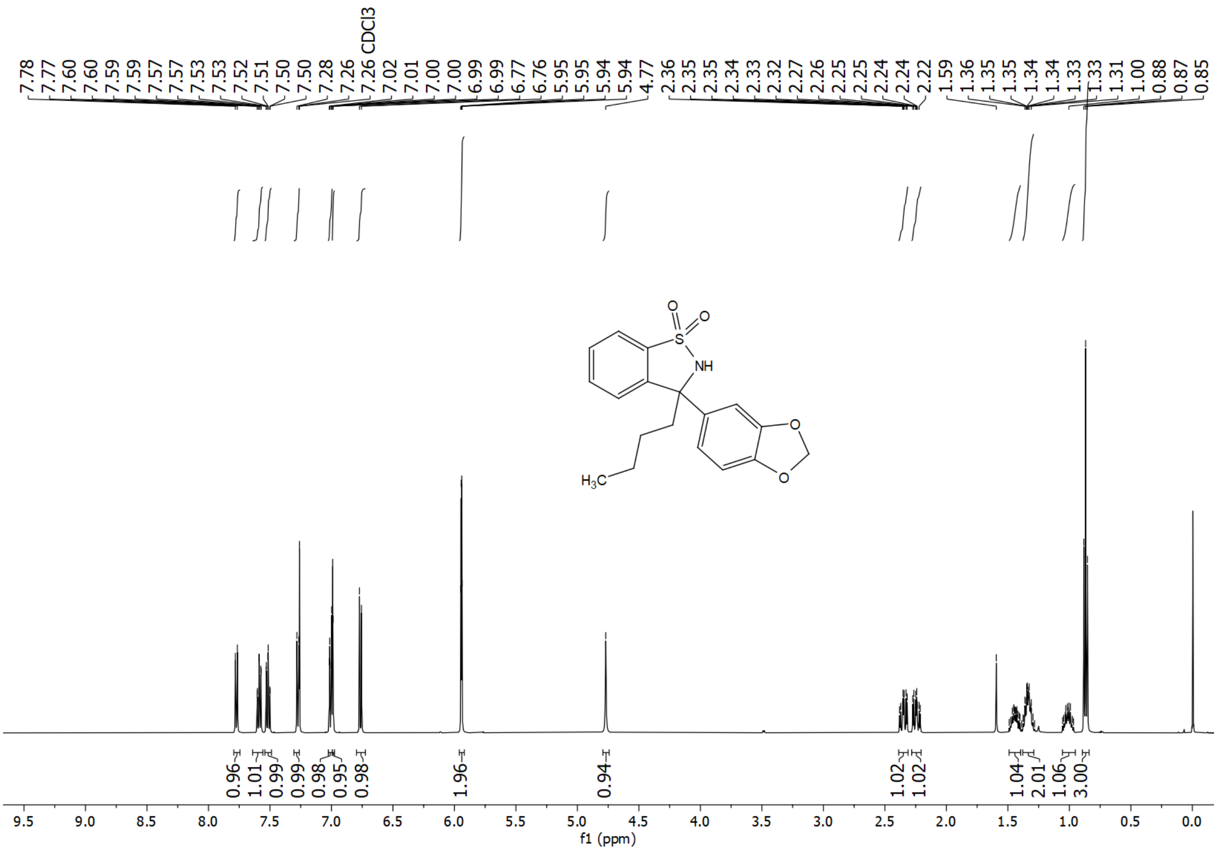

^{13}C{^{1}H} NMR (126 MHz, $CDCl_3$) of **P1c**

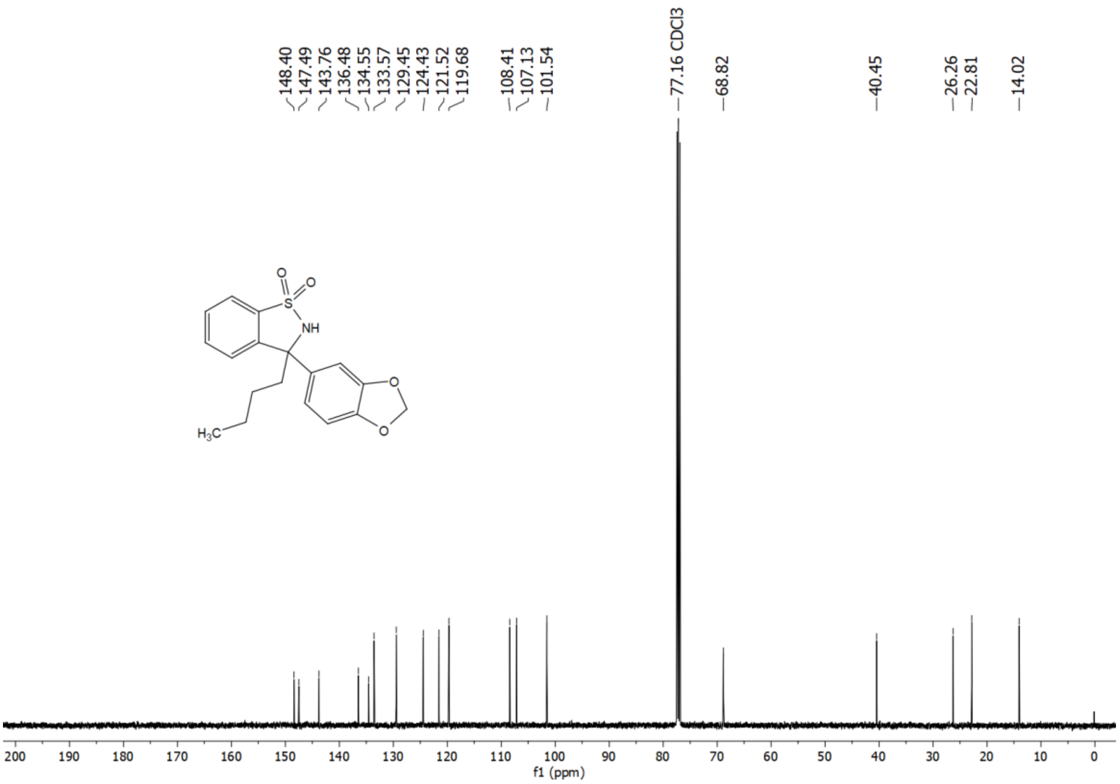

^{1}H NMR (500 MHz, $CDCl_3$) of **P1d**

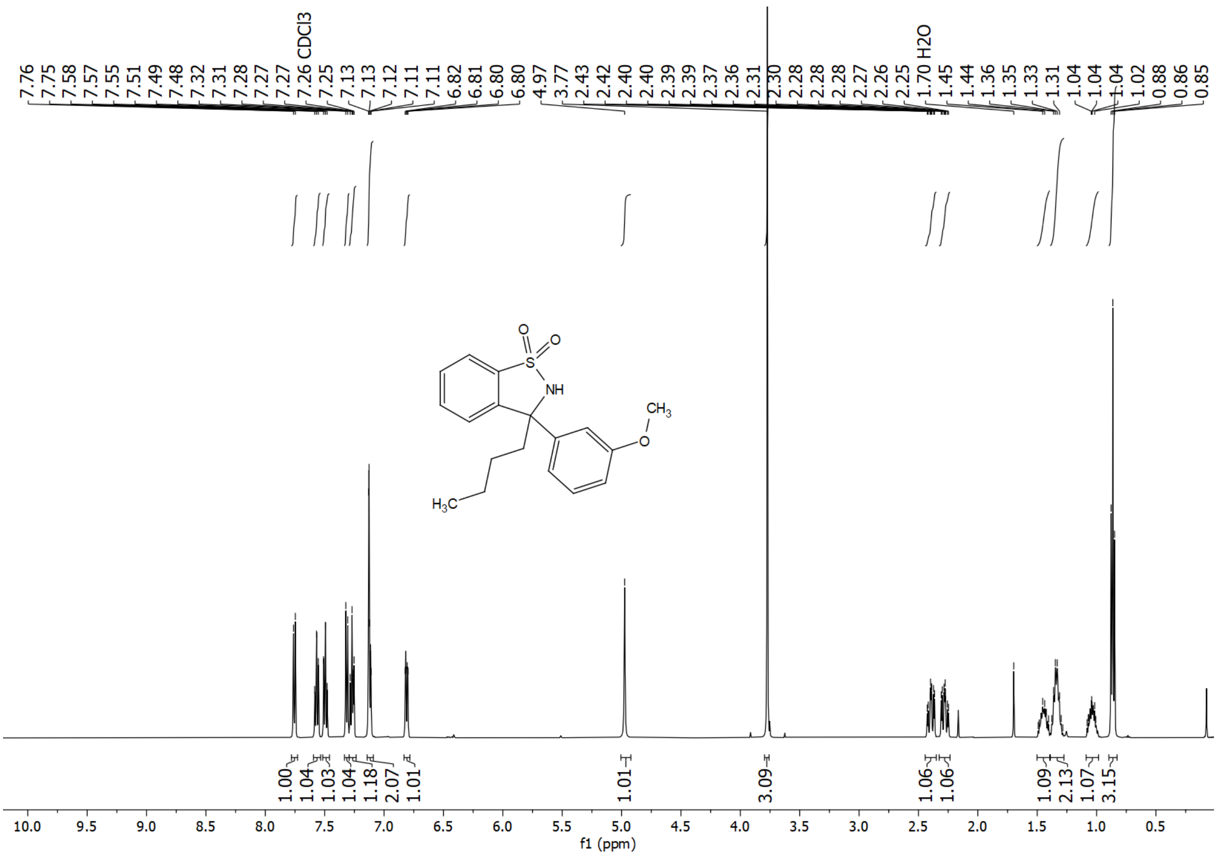

$^{13}C\{^{1}H\}$ NMR (126 MHz, $CDCl_3$) of **P1d**

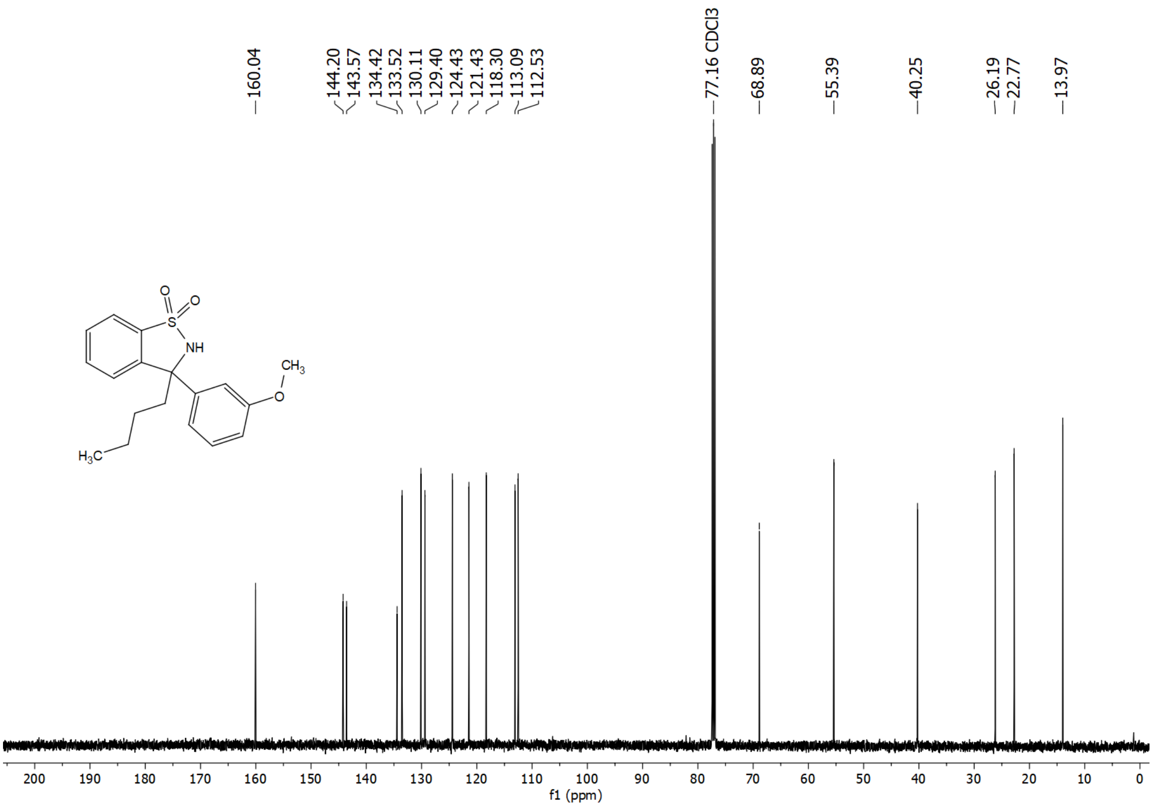

^{1}H NMR (500 MHz, $CDCl_3$) of **P1e**

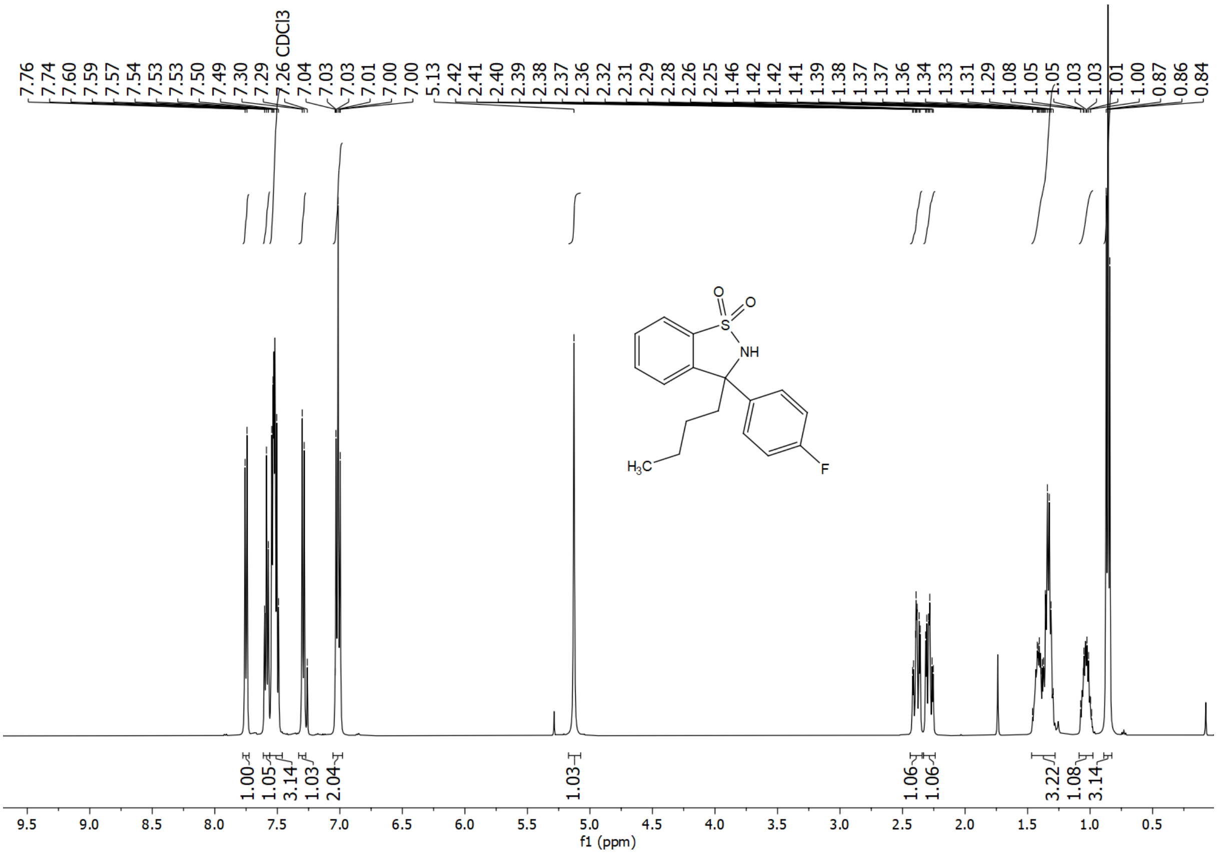

$^{13}C\{^{1}H\}$ NMR (126 MHz, $CDCl_3$) of **P1d**

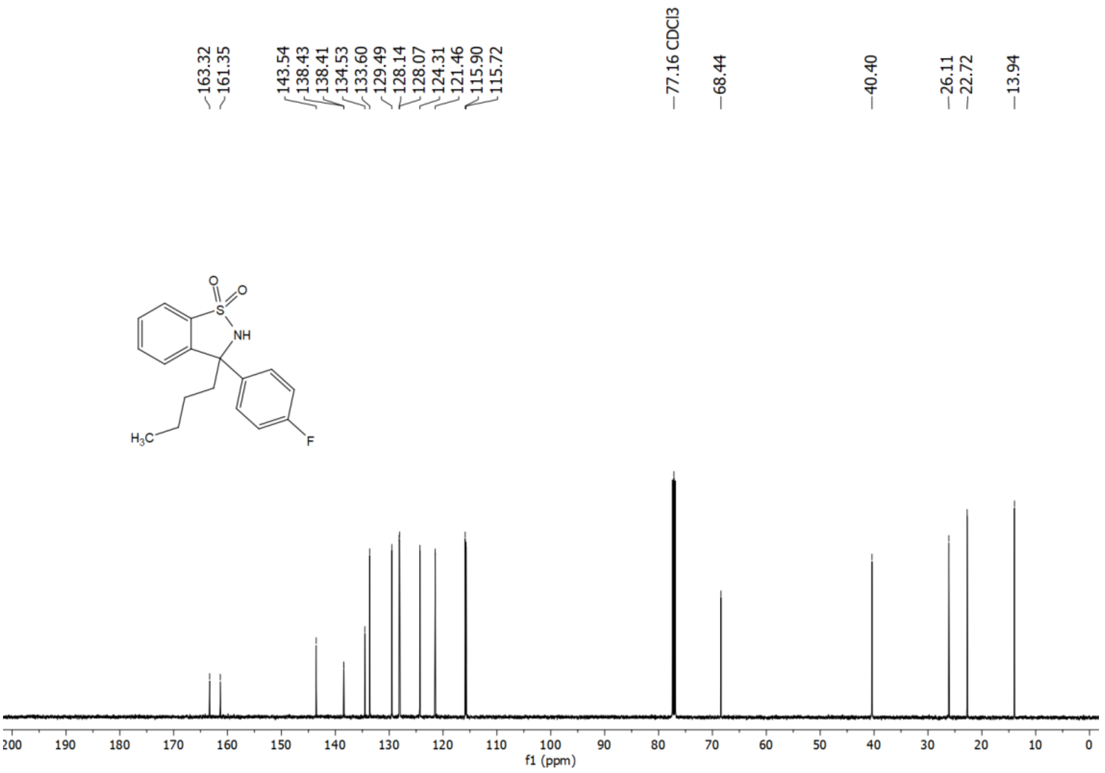

^{1}H NMR (500 MHz, CD_3OD) of **P1f**

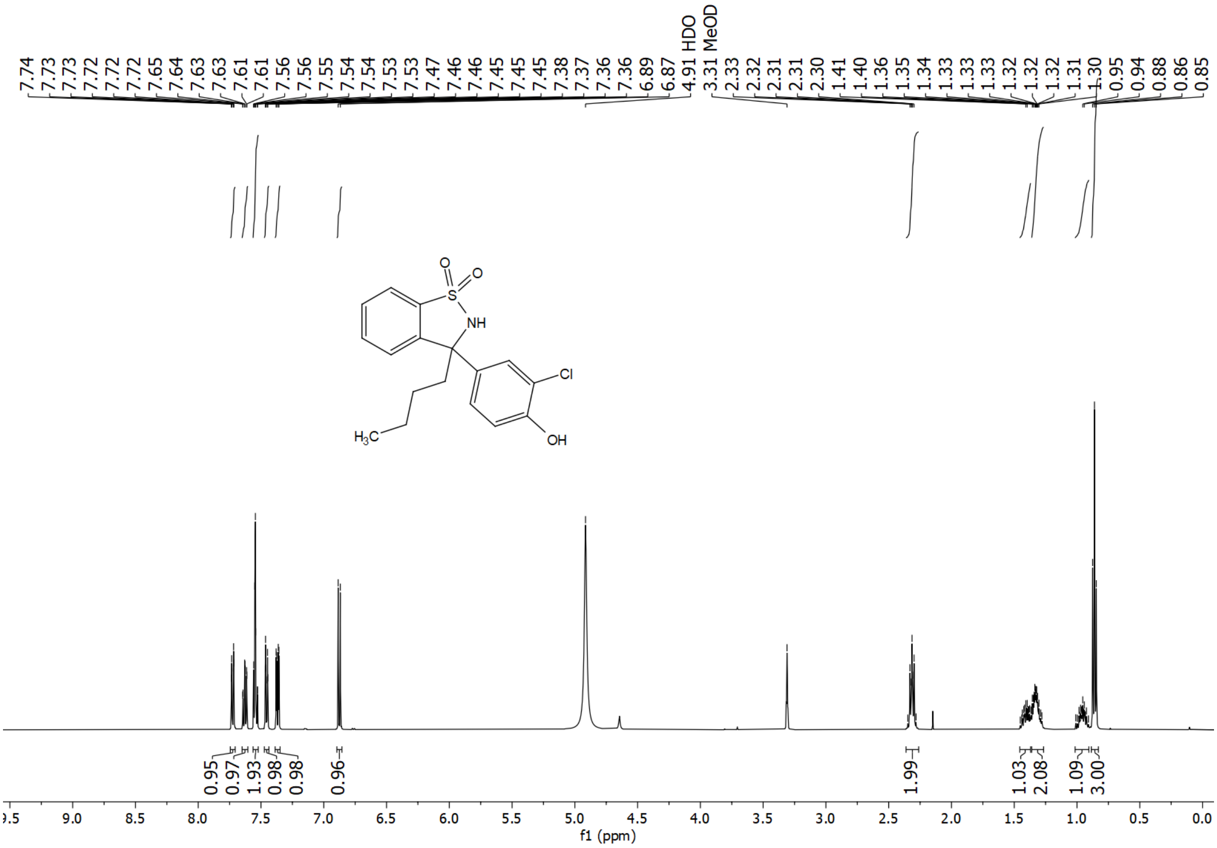

^{13}C{^{1}H} NMR (126 MHz, CD_3OD) of **P1f**

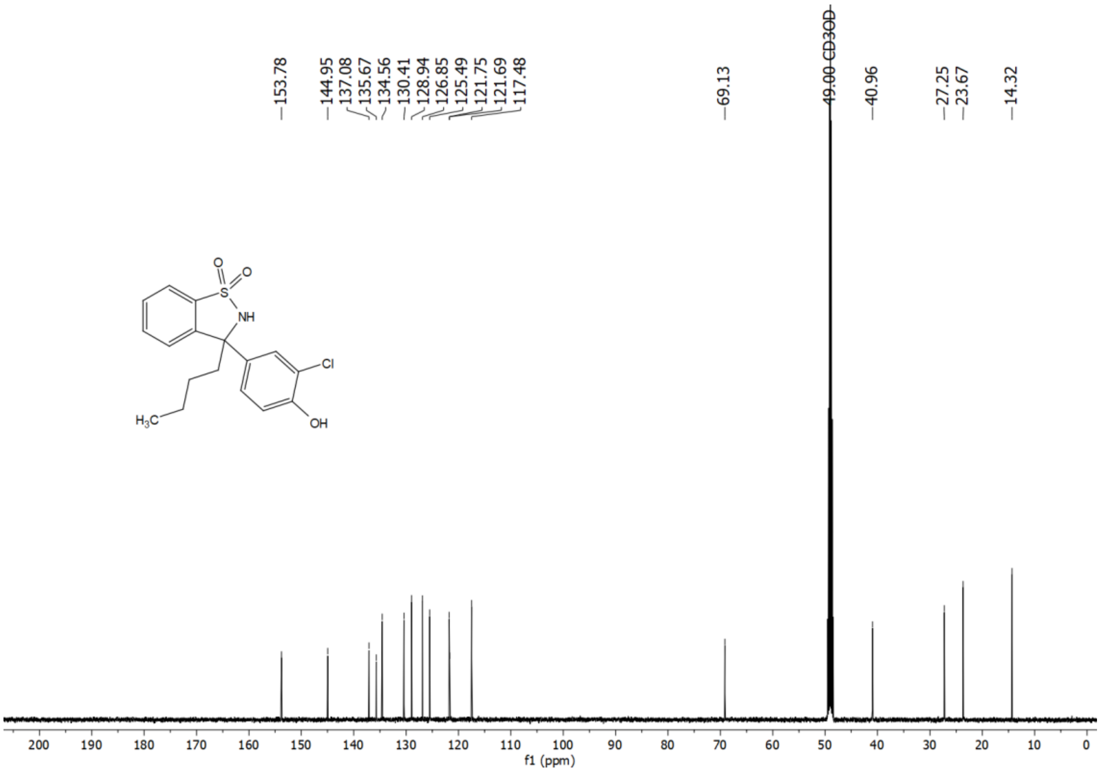

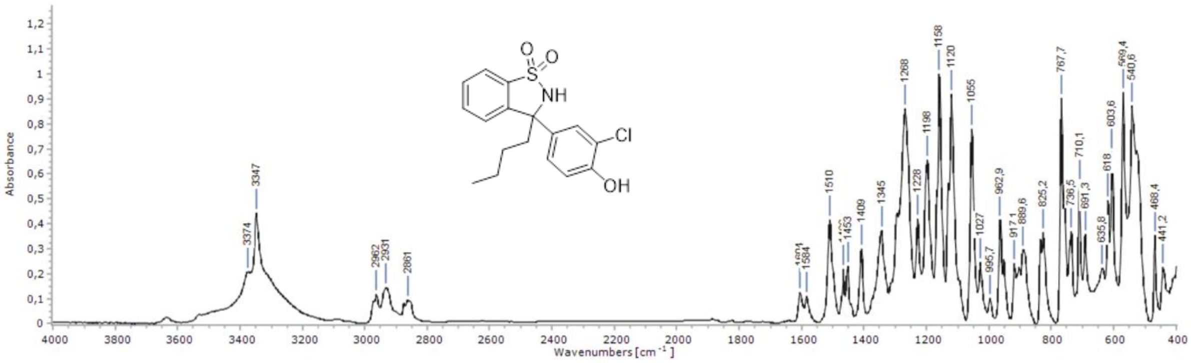

^{1}H NMR (500 MHz, CD_3OD) of **P1g**

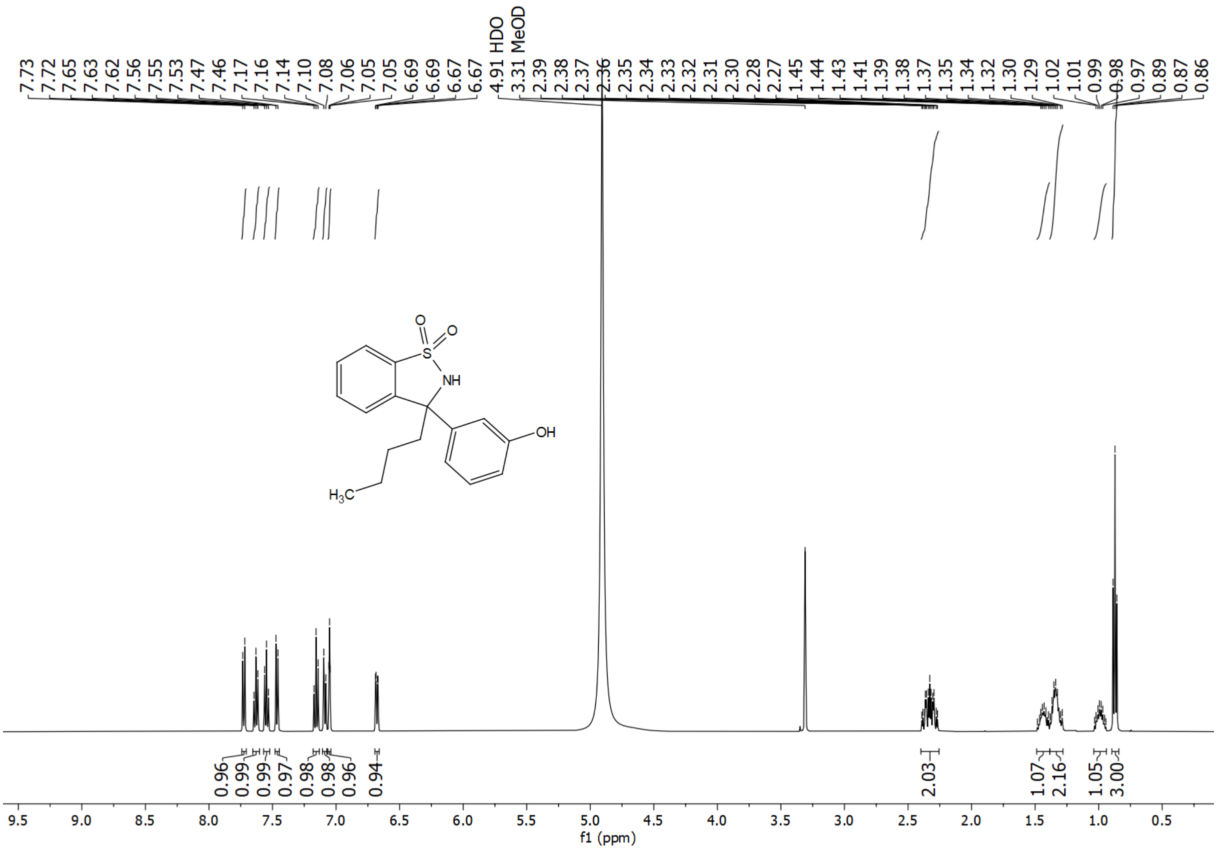

$^{13}C\{^1H\}$ NMR (126 MHz, CD_3OD) of **P1g**

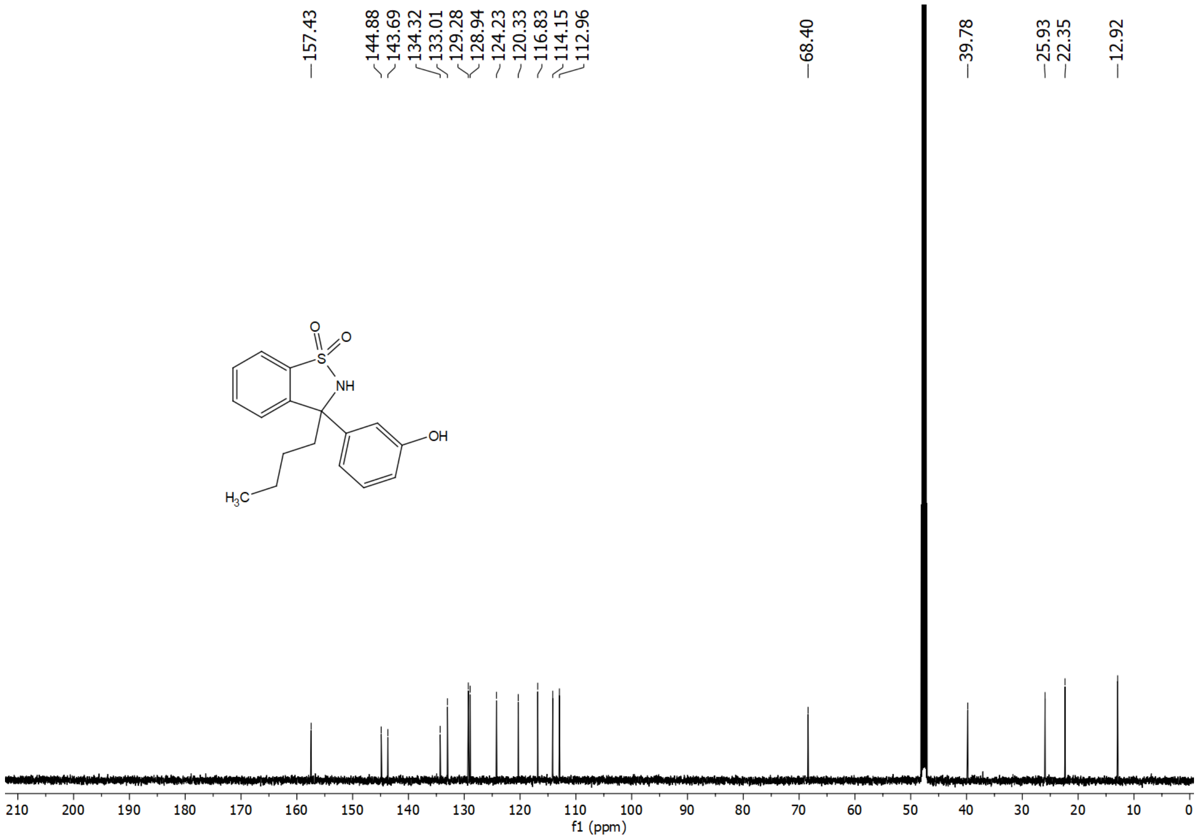

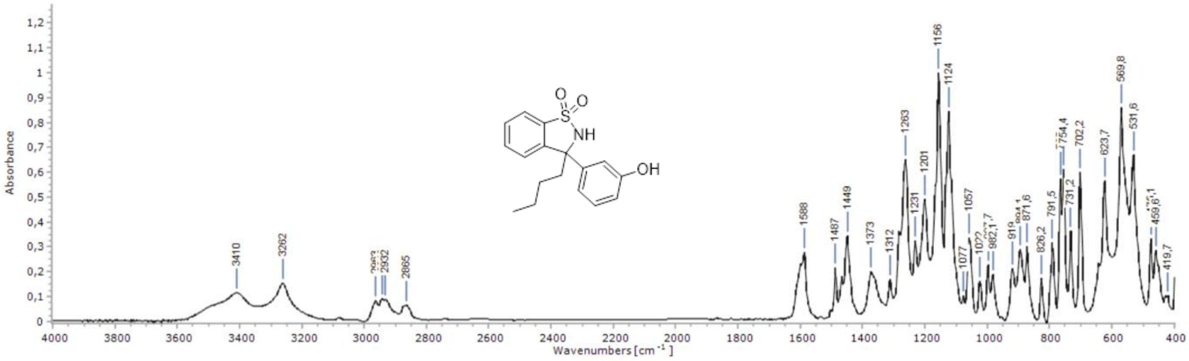

^{1}H NMR (500 MHz, CD_3OD) of **P1h**

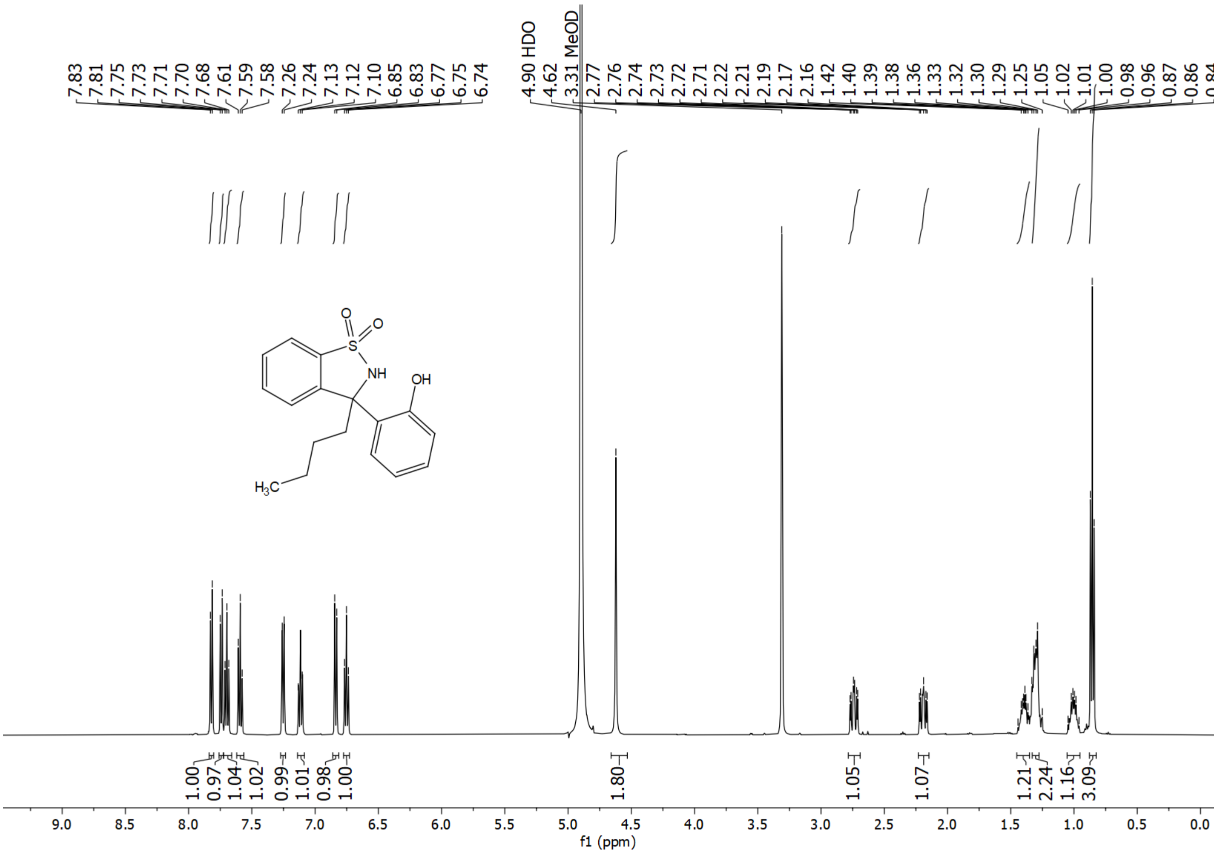

$^{13}C\{^{1}H\}$ NMR (126 MHz, CD_3OD) of **P1h**

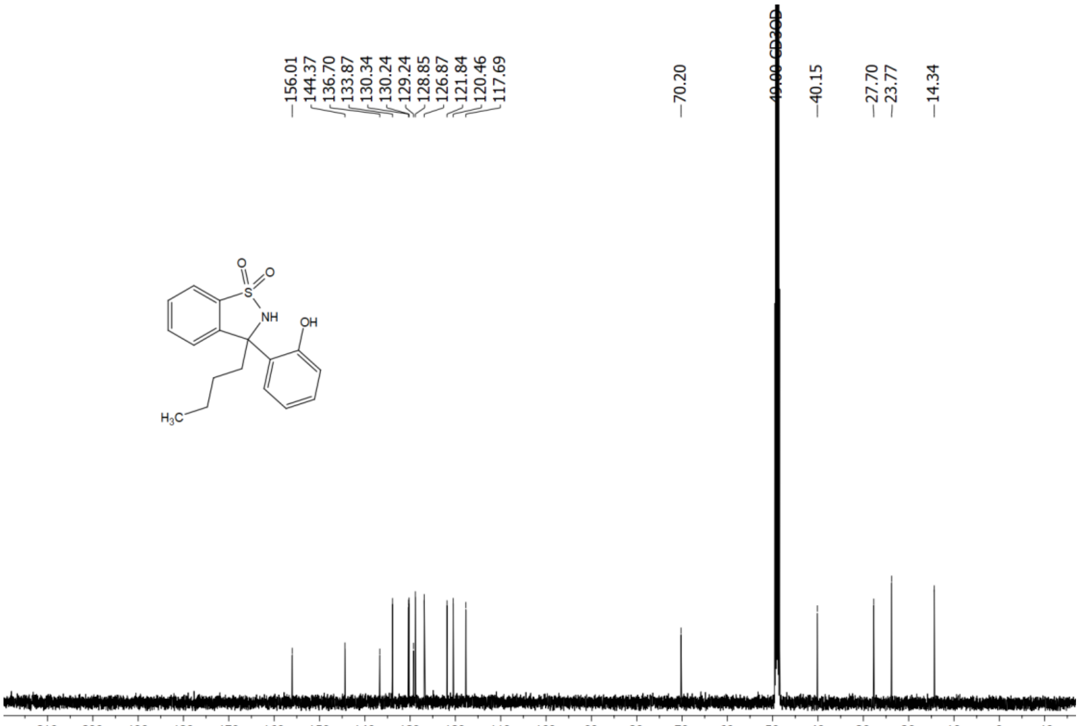

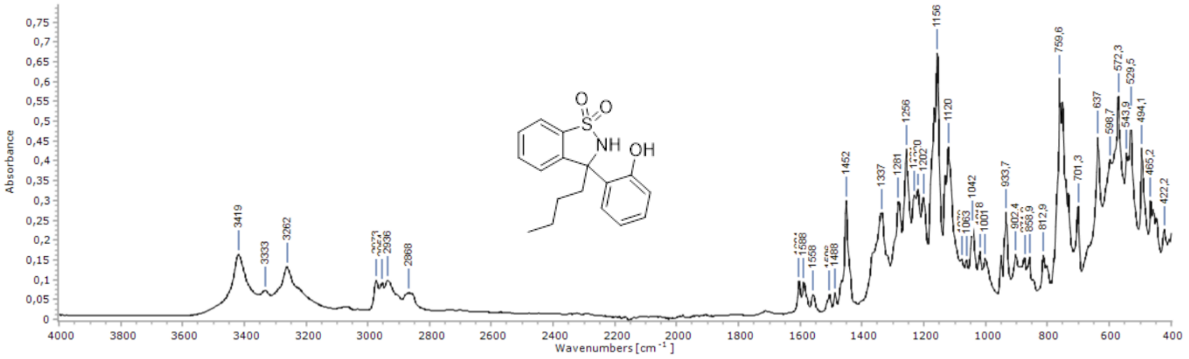

^{1}H NMR (500 MHz, DMSO-D6) of **P1i**

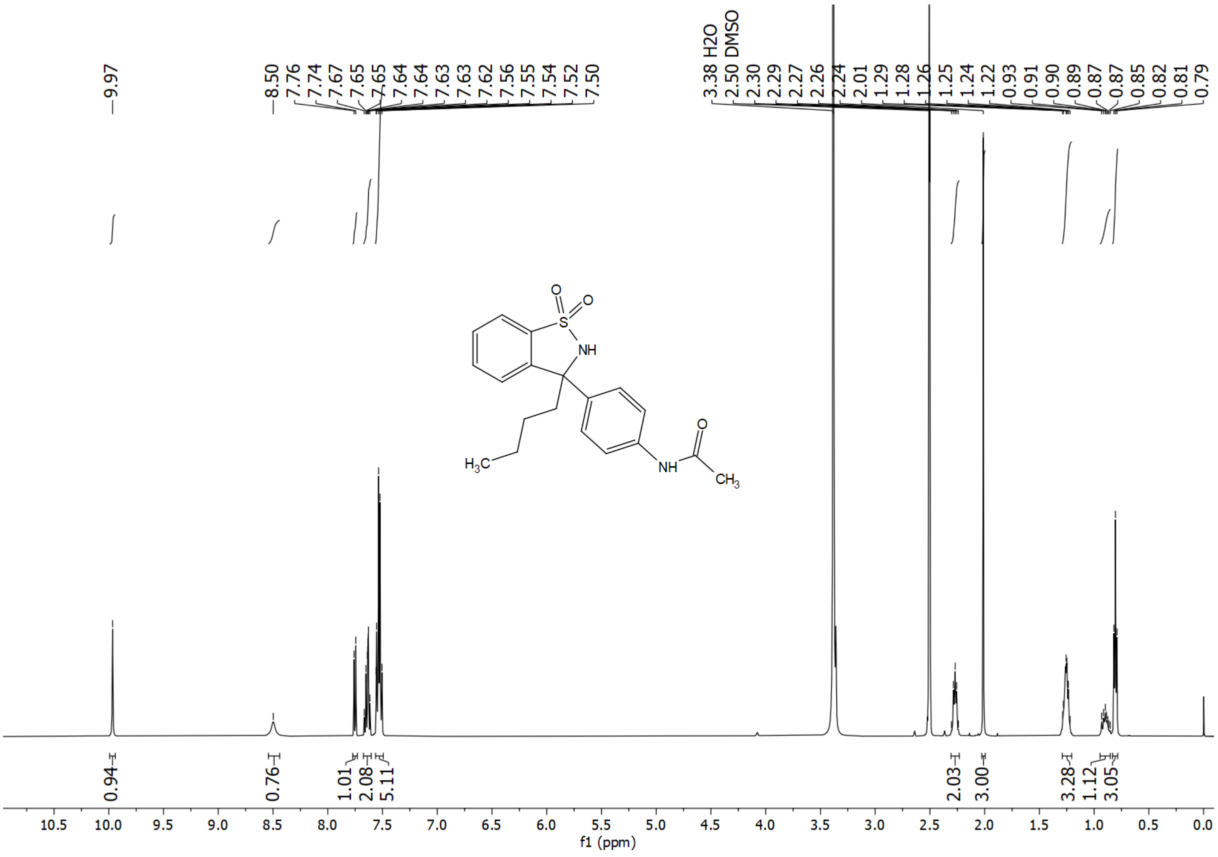

$^{13}C\{^{1}H\}$ NMR (126 MHz, DMSO-D6) of **P1i**

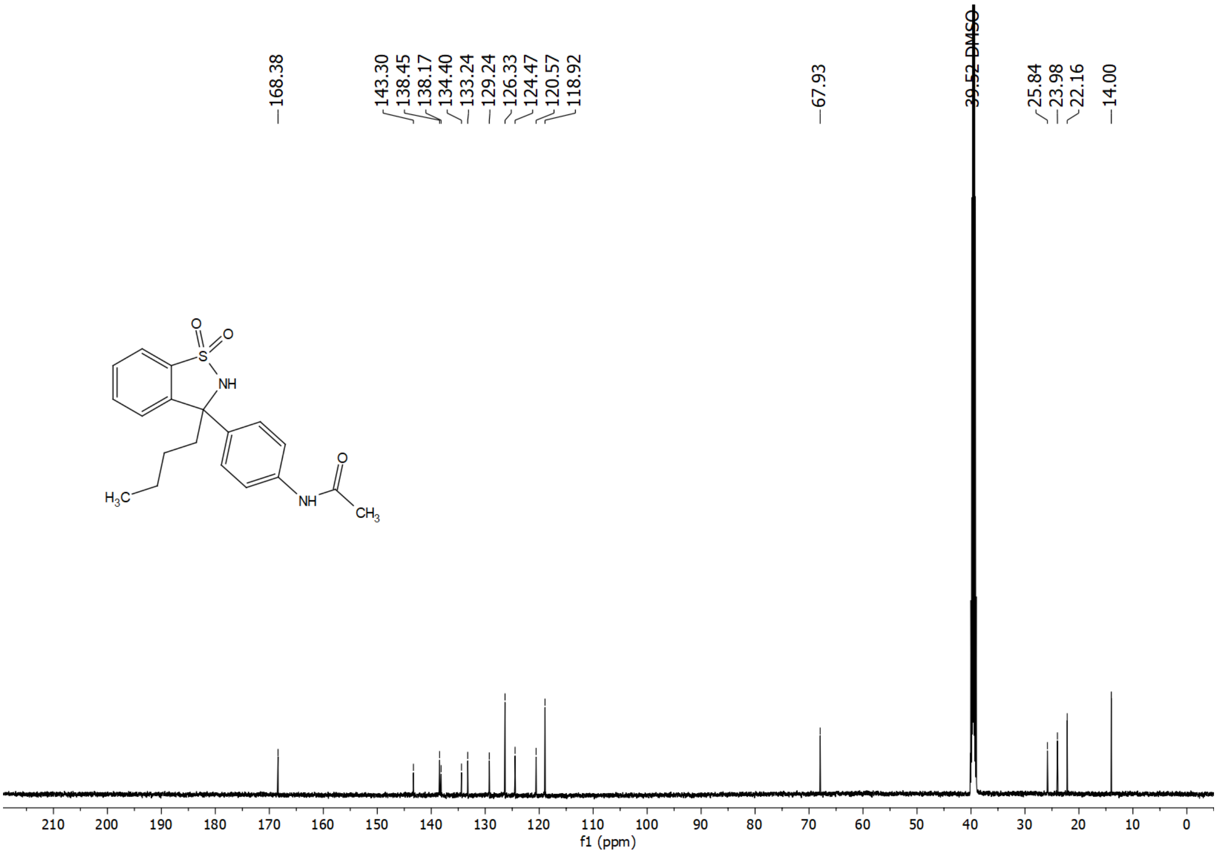

^{13}C APT NMR (126 MHz, DMSO-D6) of **P1i**

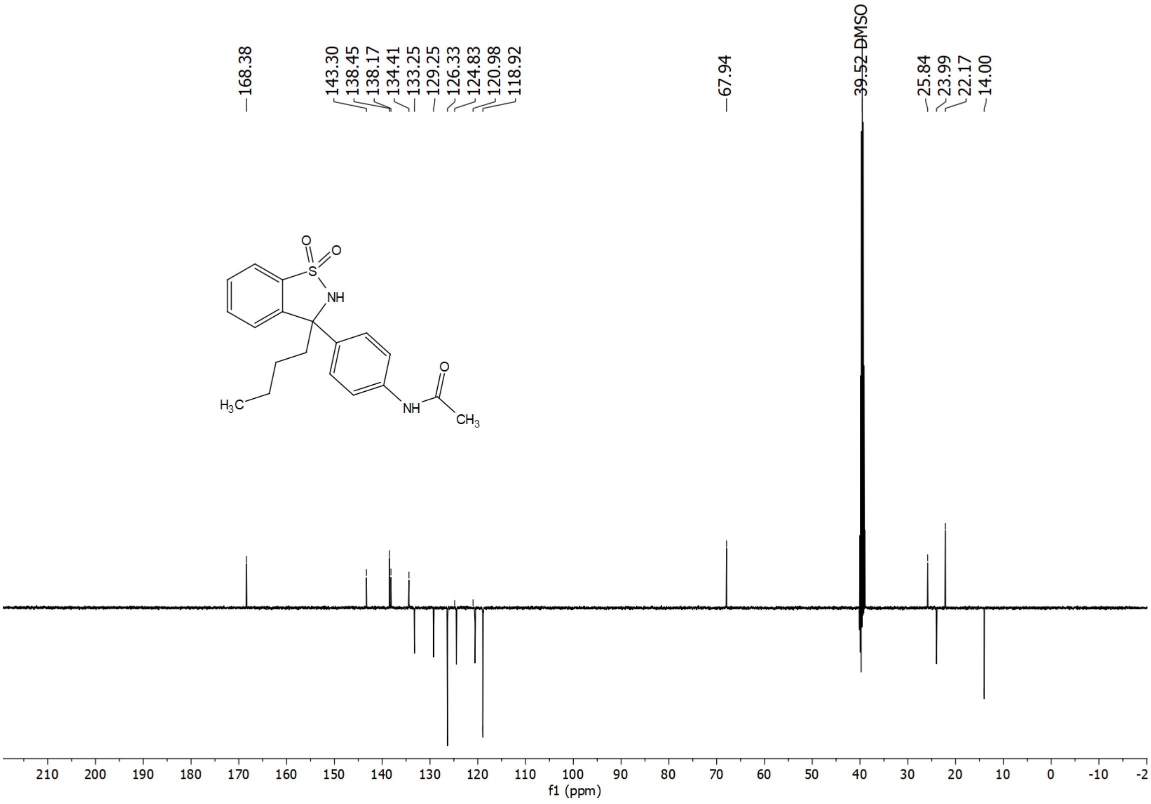

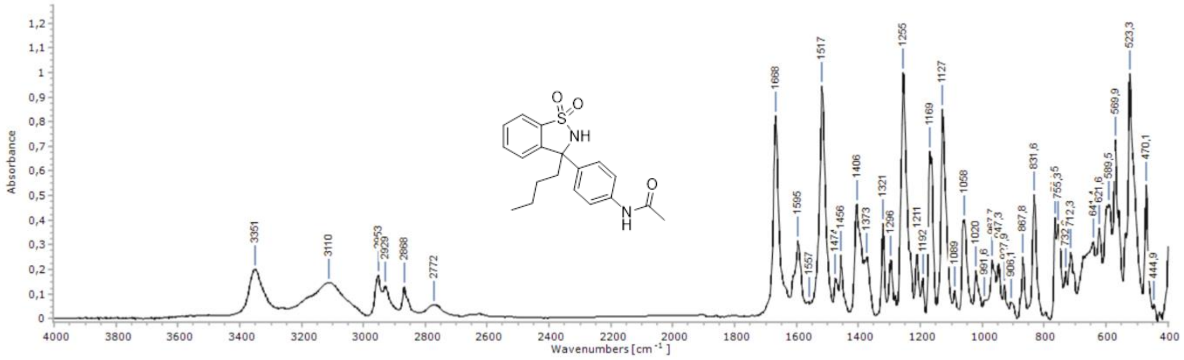

^{1}H NMR (500 MHz, $CDCl_3$) of **P1j**

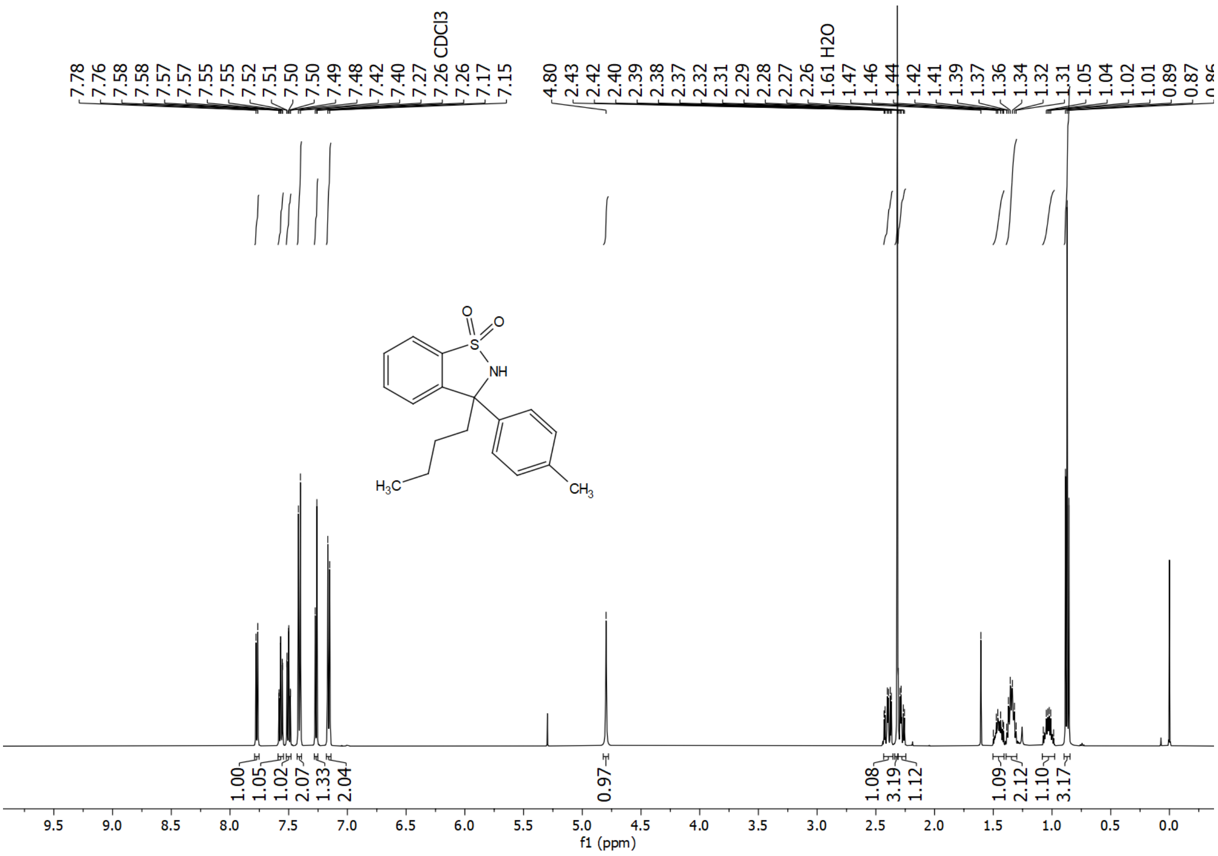

$^{13}C\{^{1}H\}$ NMR (126 MHz, $CDCl_3$) of **P1j**

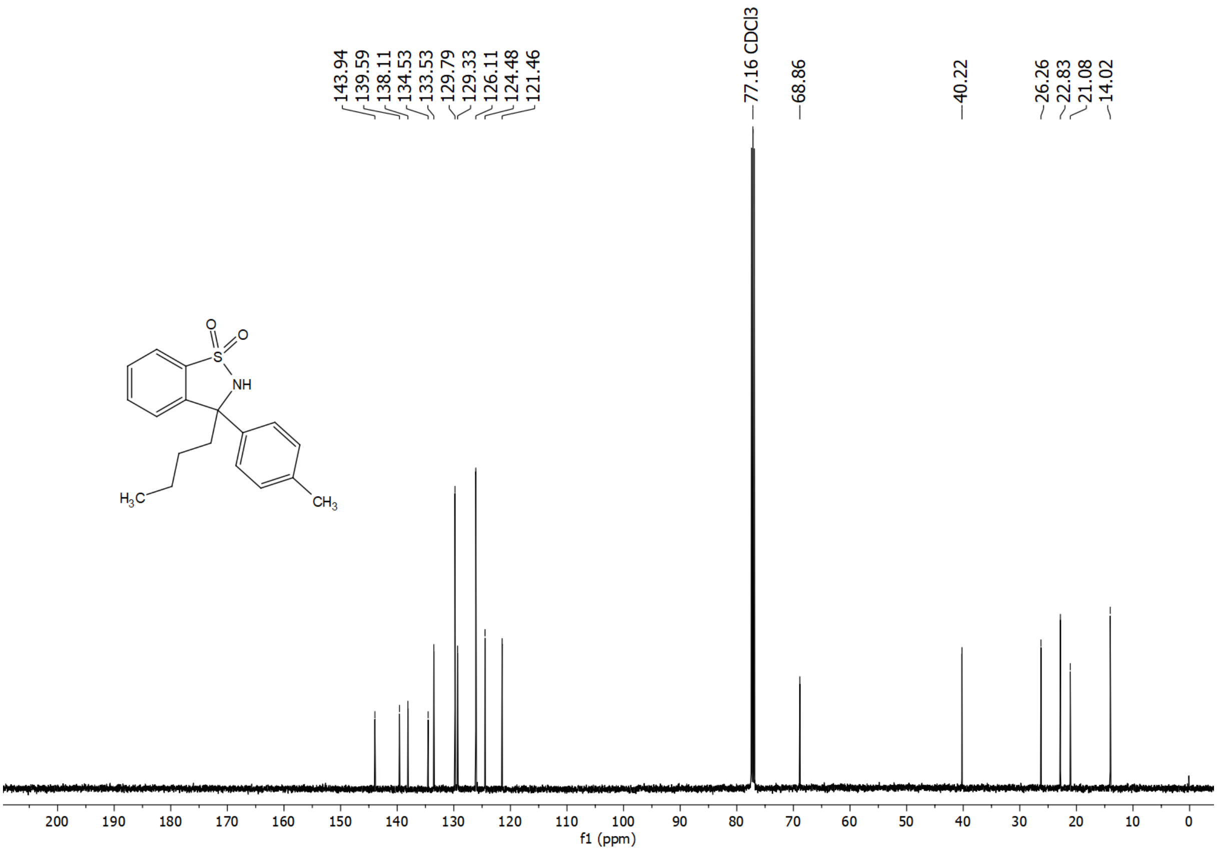

^{1}H NMR (500 MHz, $CDCl_3$) of **P1k**

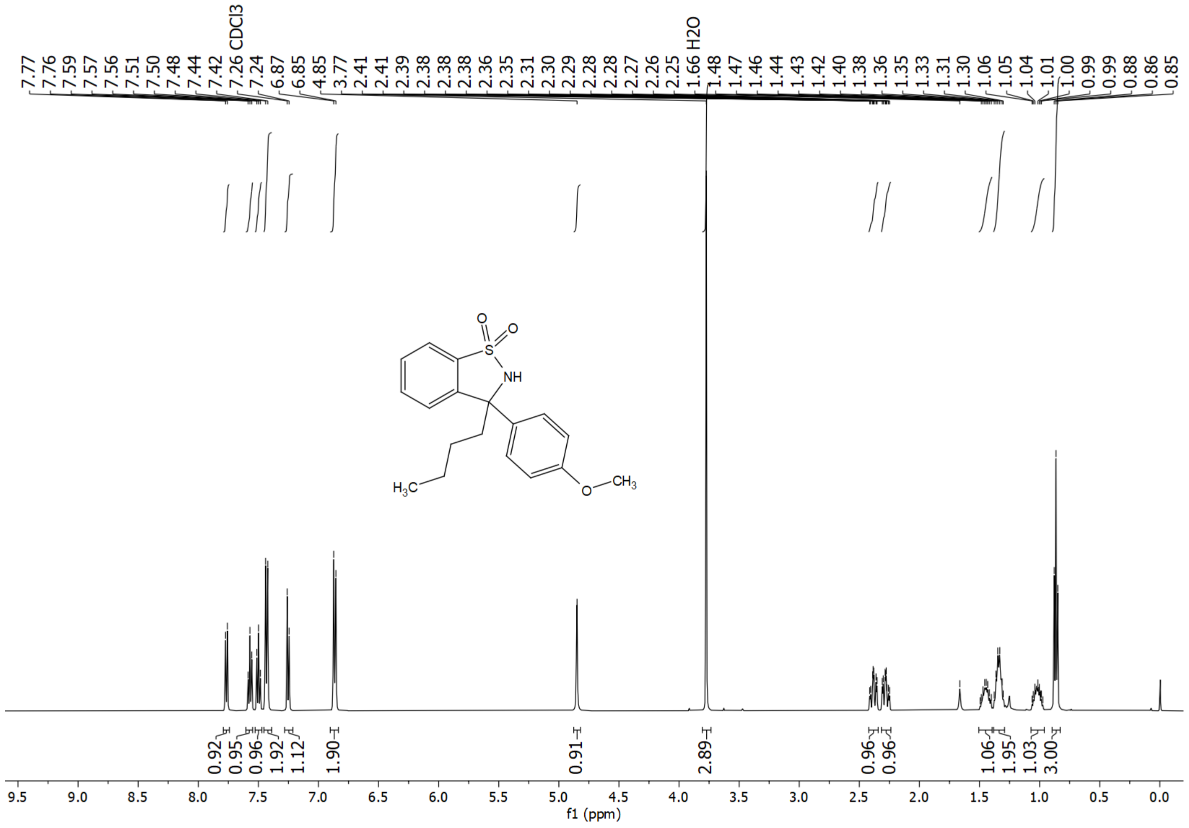

$^{13}C\{^{1}H\}$ NMR (126 MHz, $CDCl_3$) of **P1k**

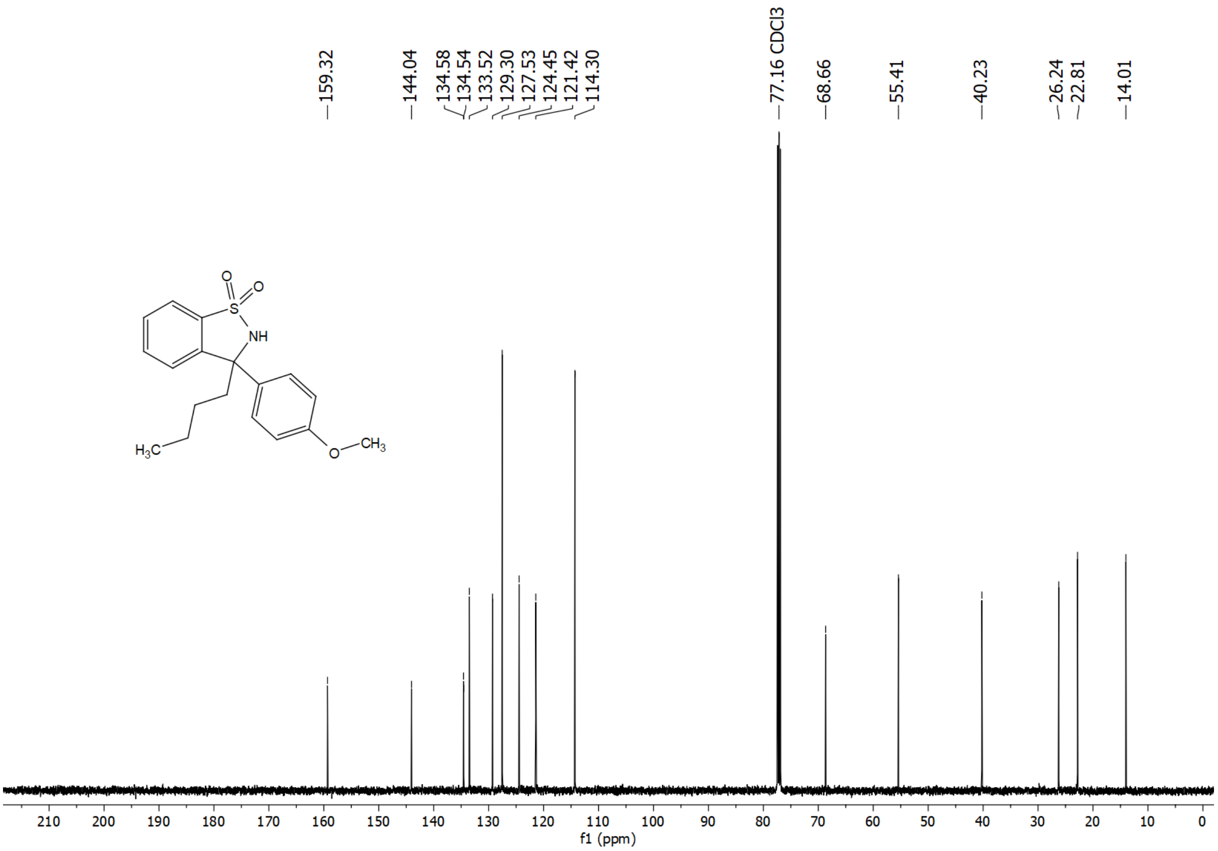

^{1}H NMR (500 MHz, CD_3OD) of **P1l**

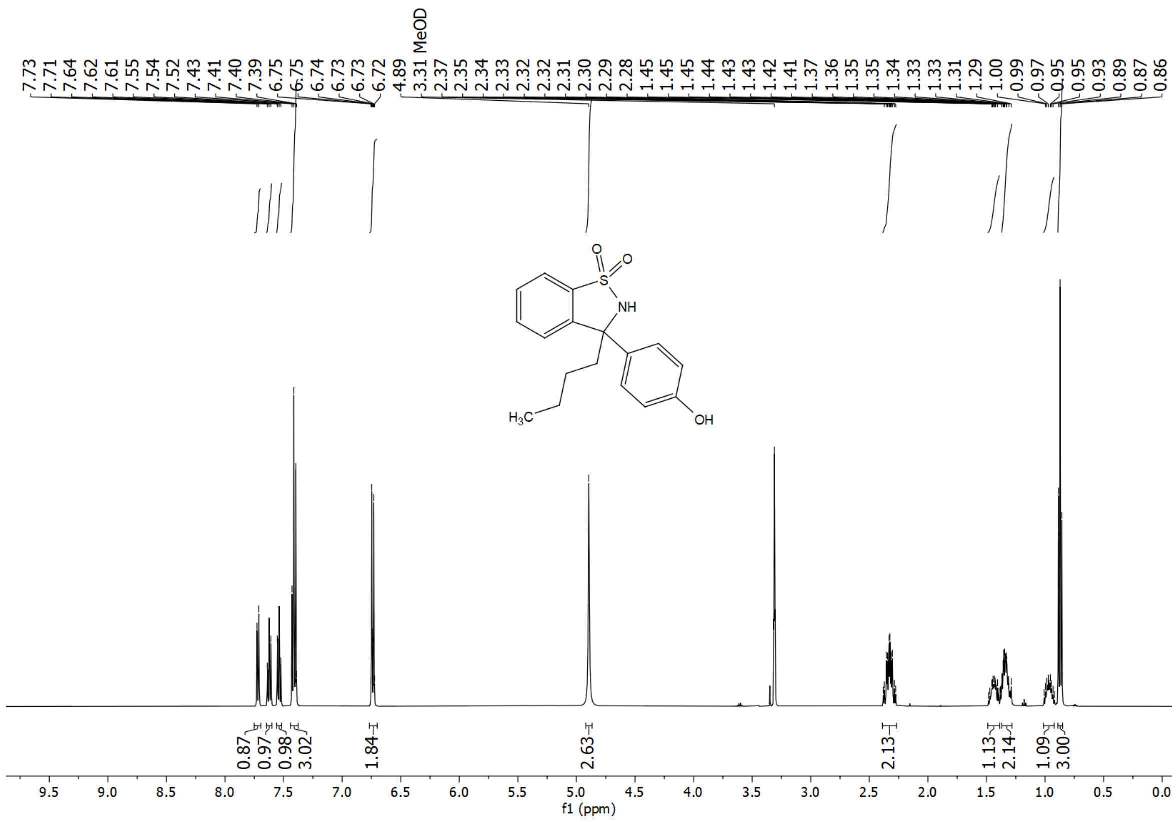

$^{13}C\{^{1}H\}$ NMR (126 MHz, CD_3OD) of **P1l**

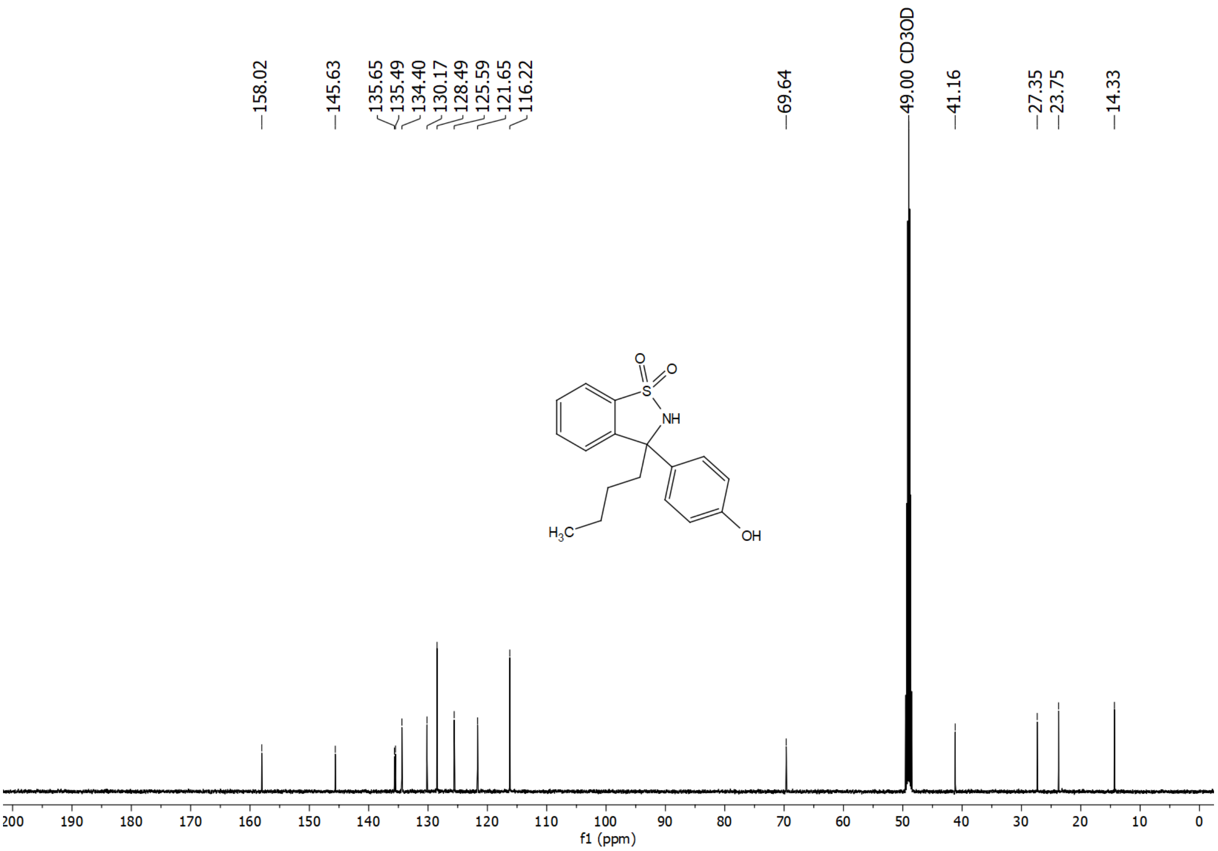

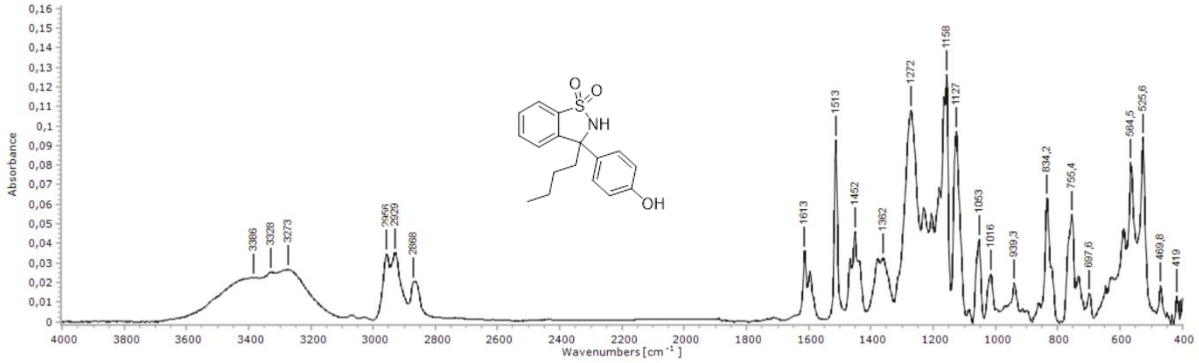

^{1}H NMR (500 MHz, $CDCl_3$) of **P1m**

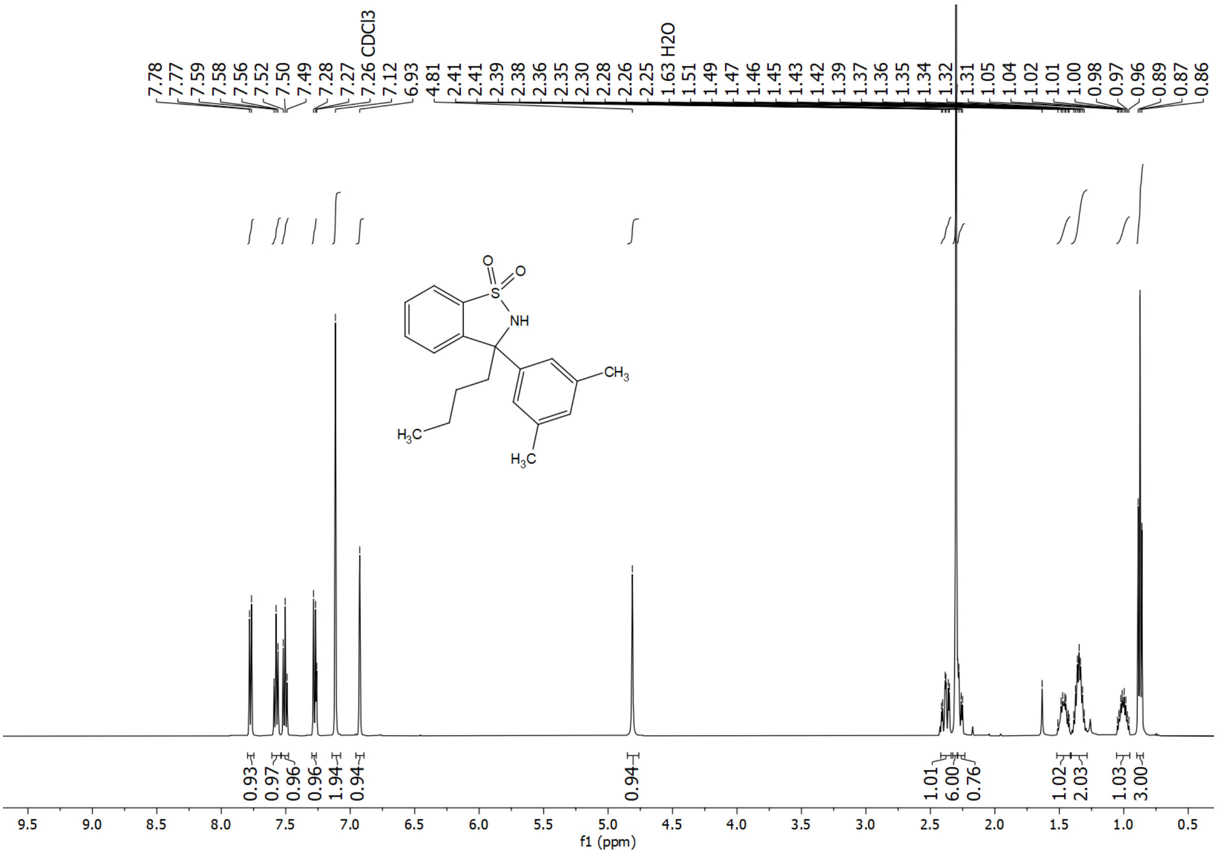

^{13}C{^{1}H} NMR (126 MHz, $CDCl_3$) of **P1m**

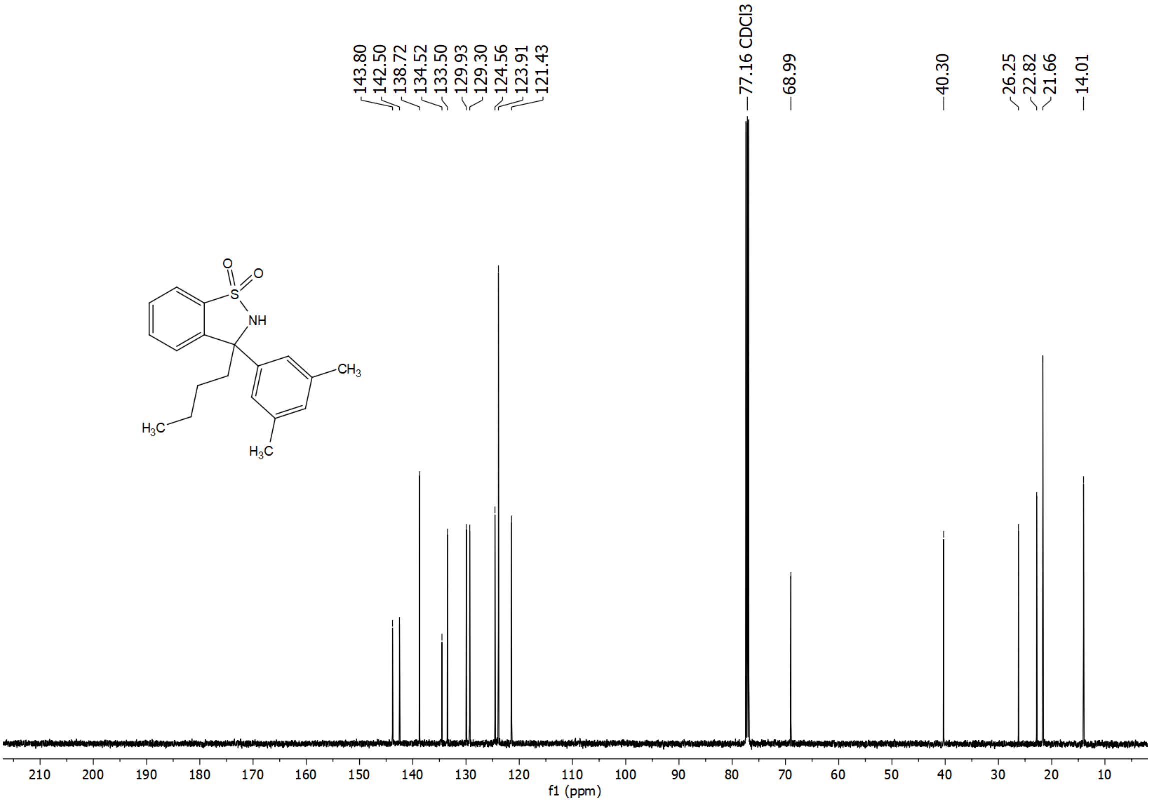

^{1}H NMR (500 MHz, CD_3OD) of **P2l**

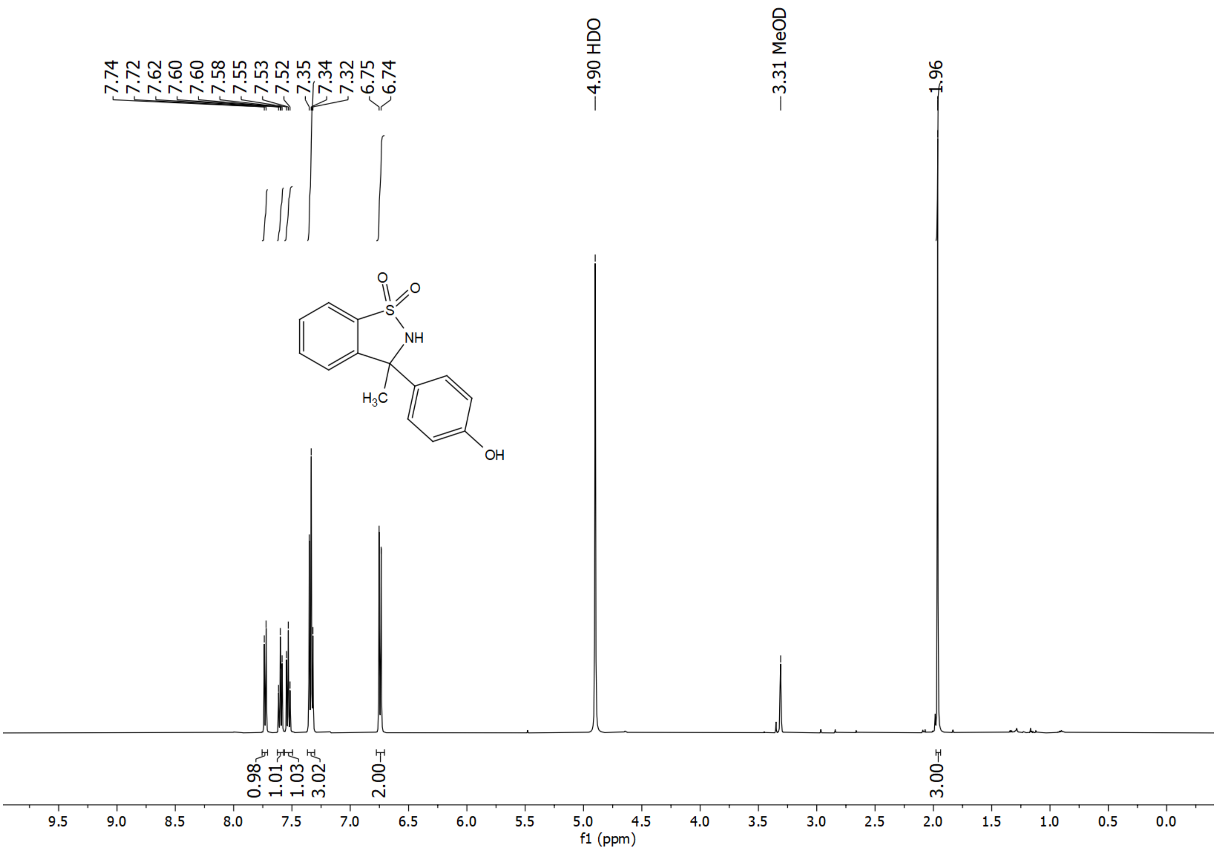

$^{13}C\{^{1}H\}$ NMR (126 MHz, $CDCl_3$) of **P2l**

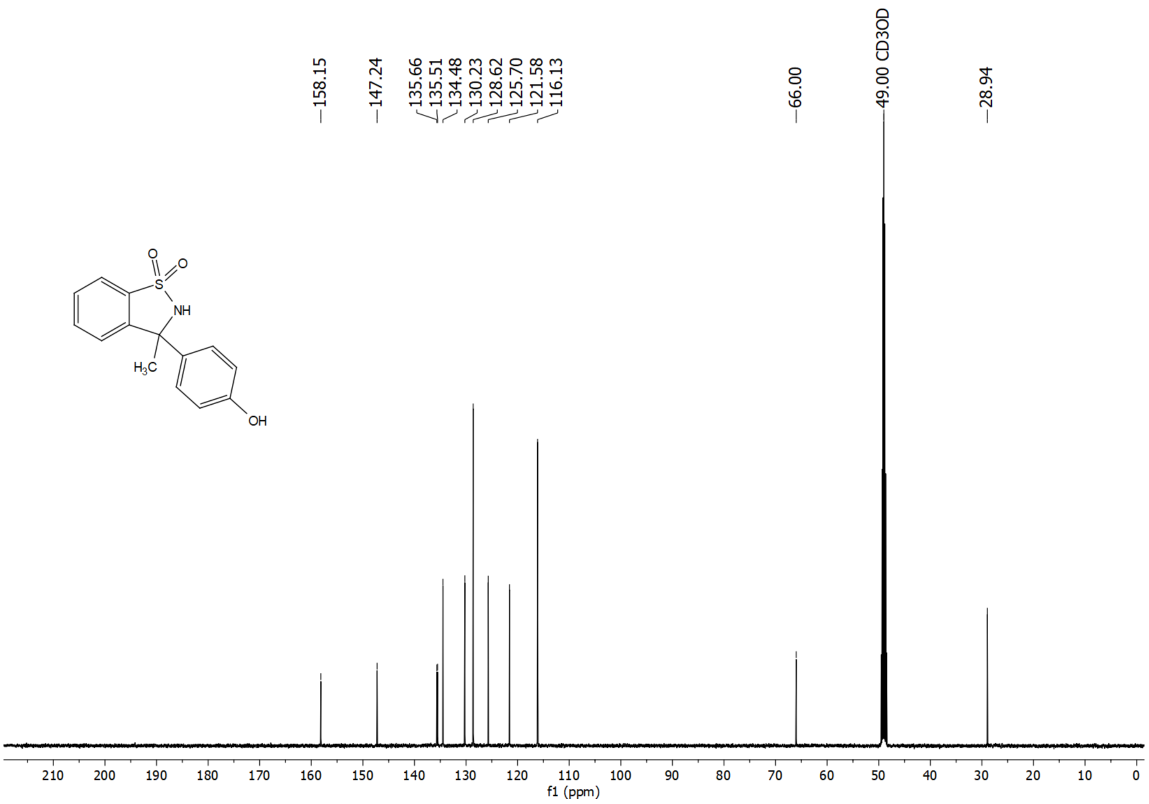

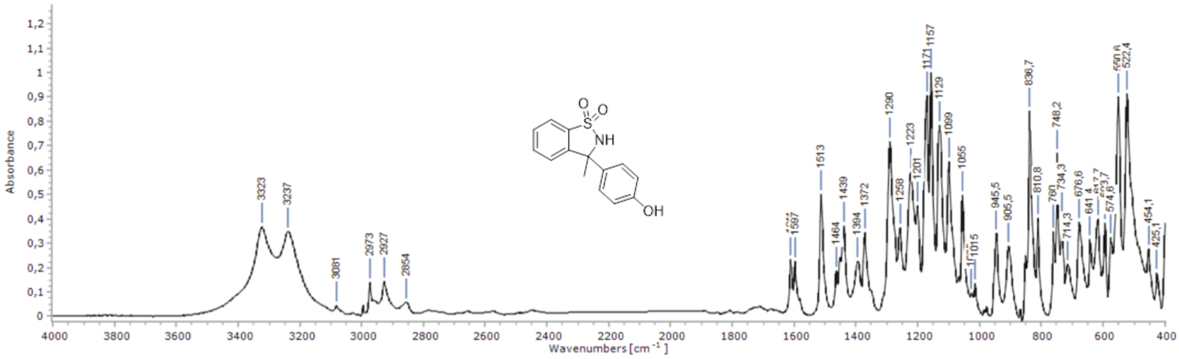

^{1}H NMR (500 MHz, $CDCl_3$) of **P2k**

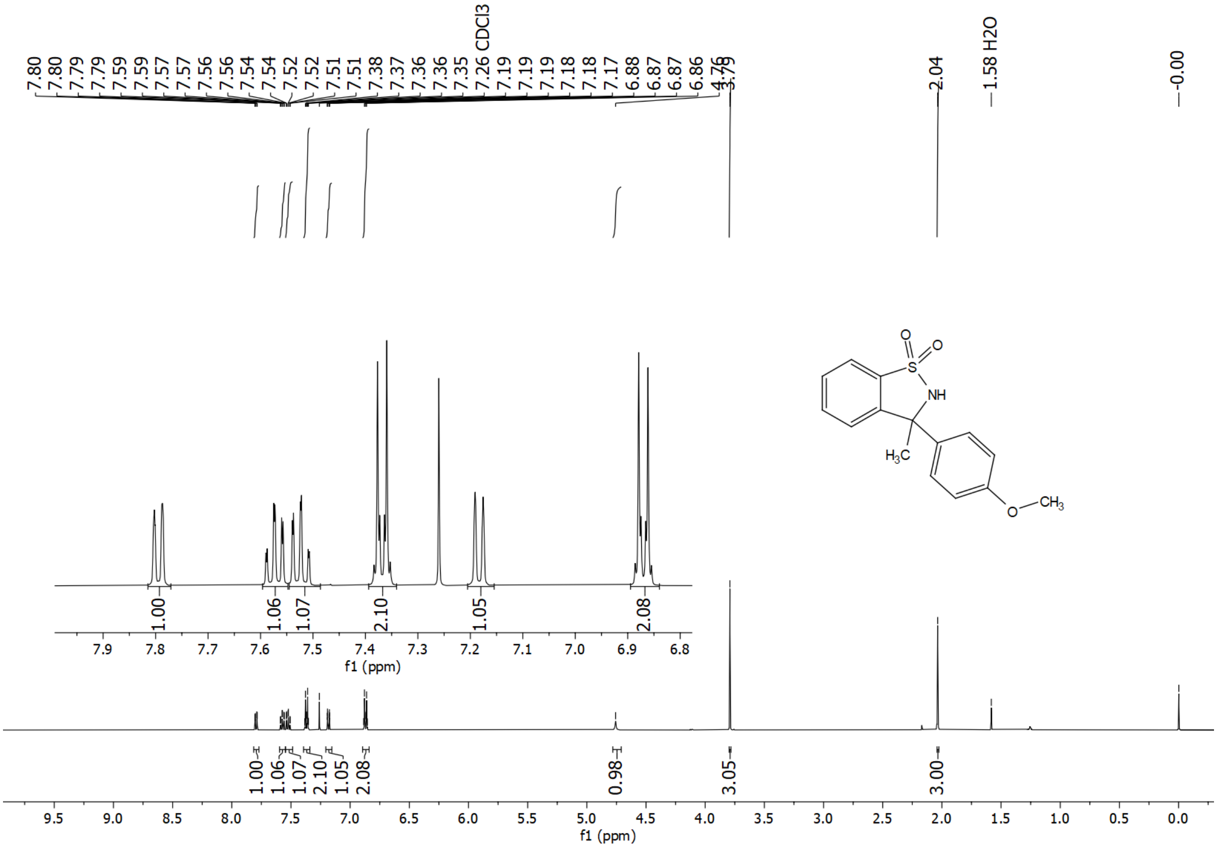

$^{13}C\{^1H\}$ NMR (126 MHz, $CDCl_3$) of **P2k**

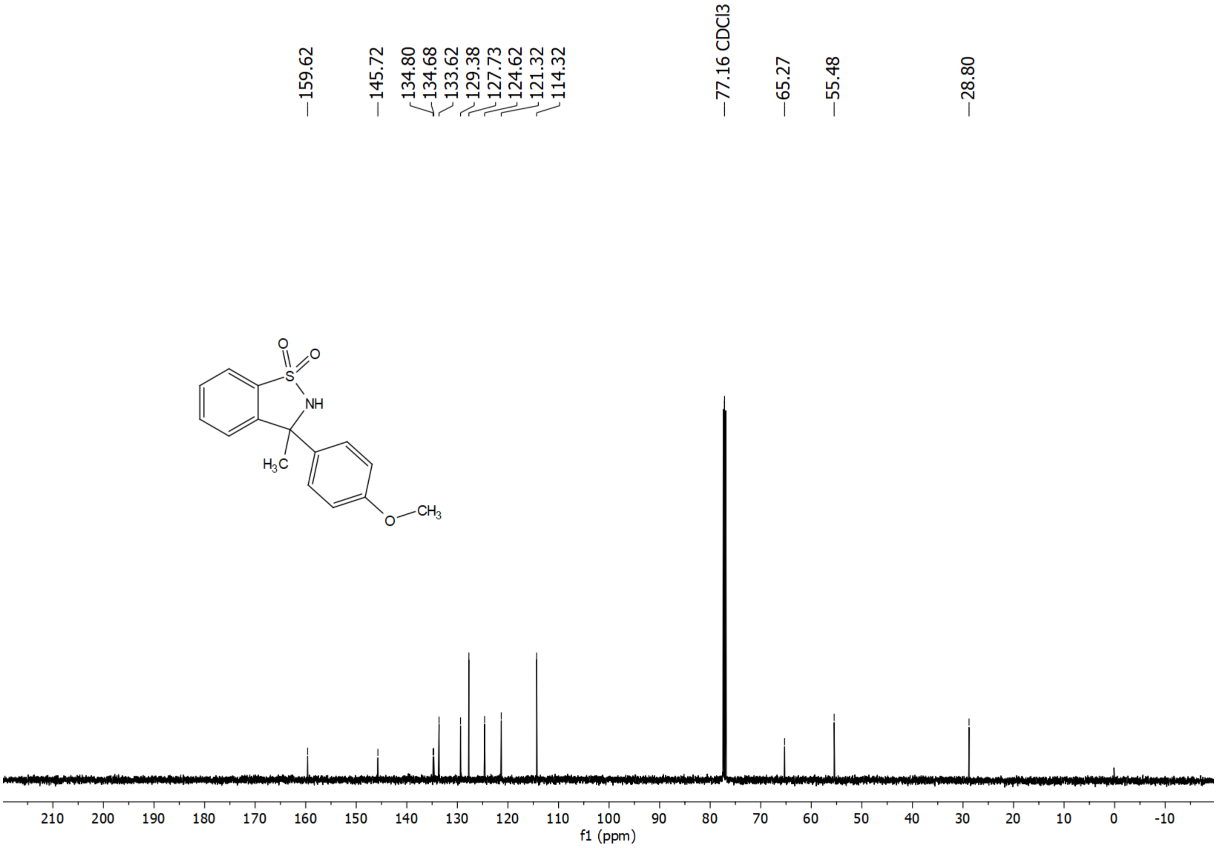

^1H NMR (500 MHz, CD_3OD) of **P3l**

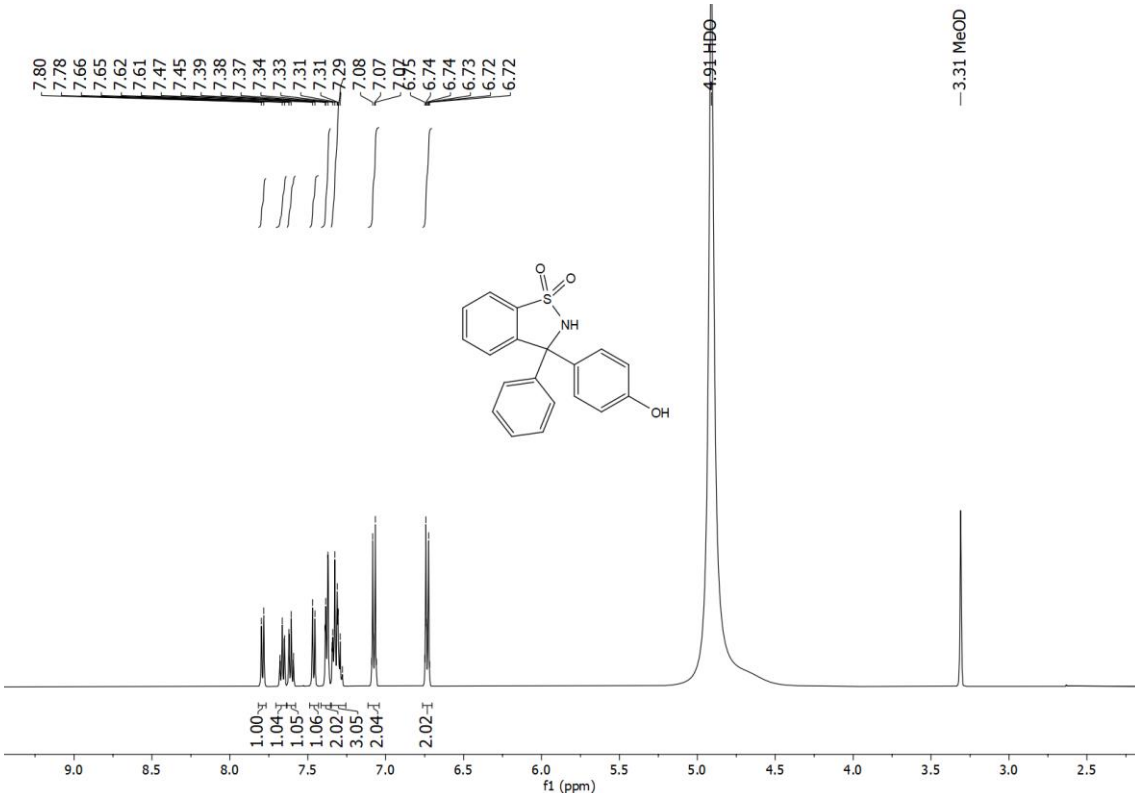

$^{13}C\{^{1}H\}$ NMR (126 MHz, CD_3OD) of **P3l**

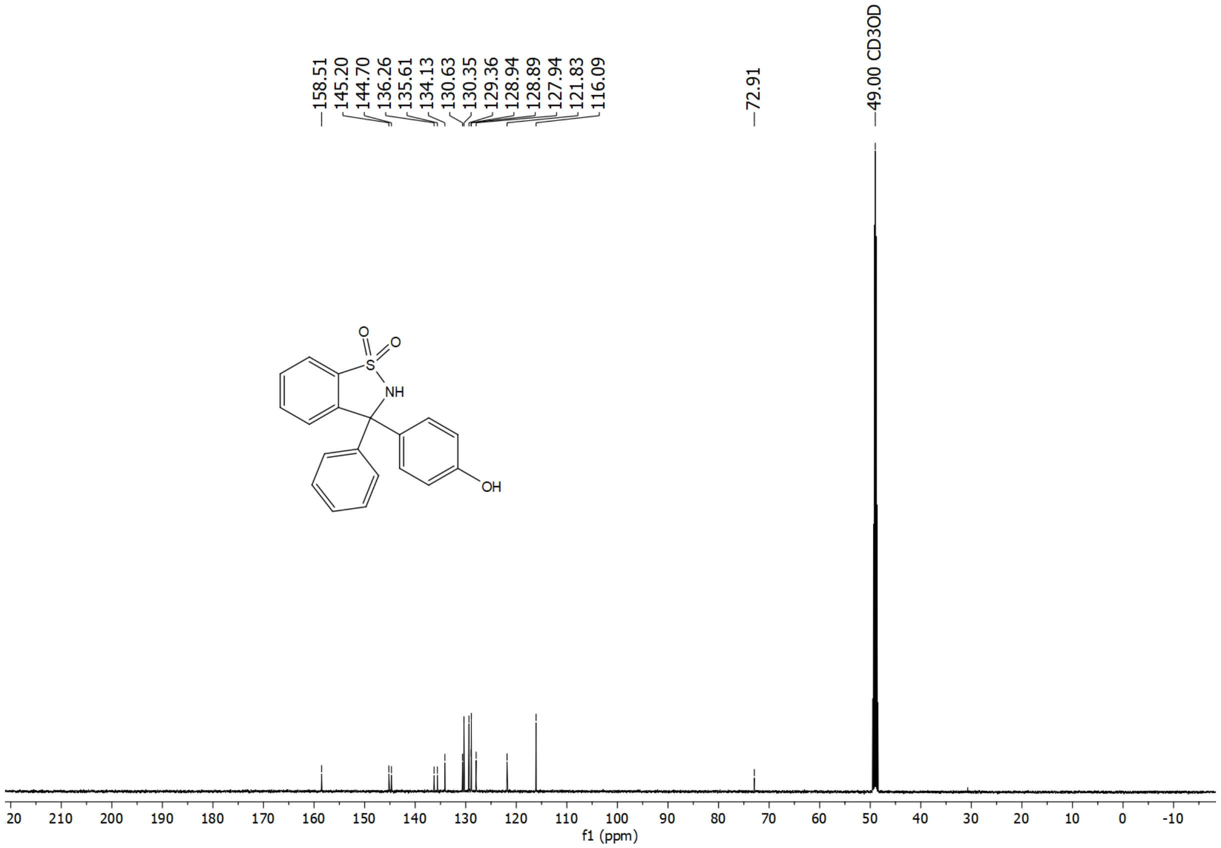

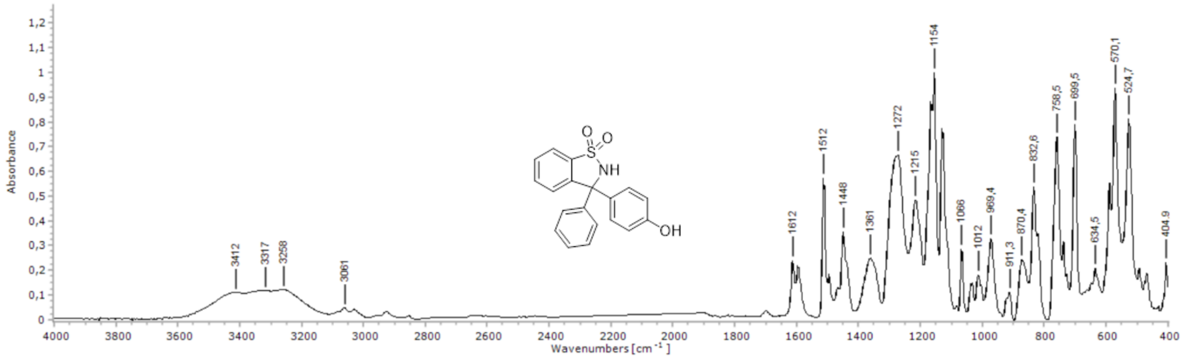

^{1}H NMR (500 MHz, $CDCl_3$) of **P3k**

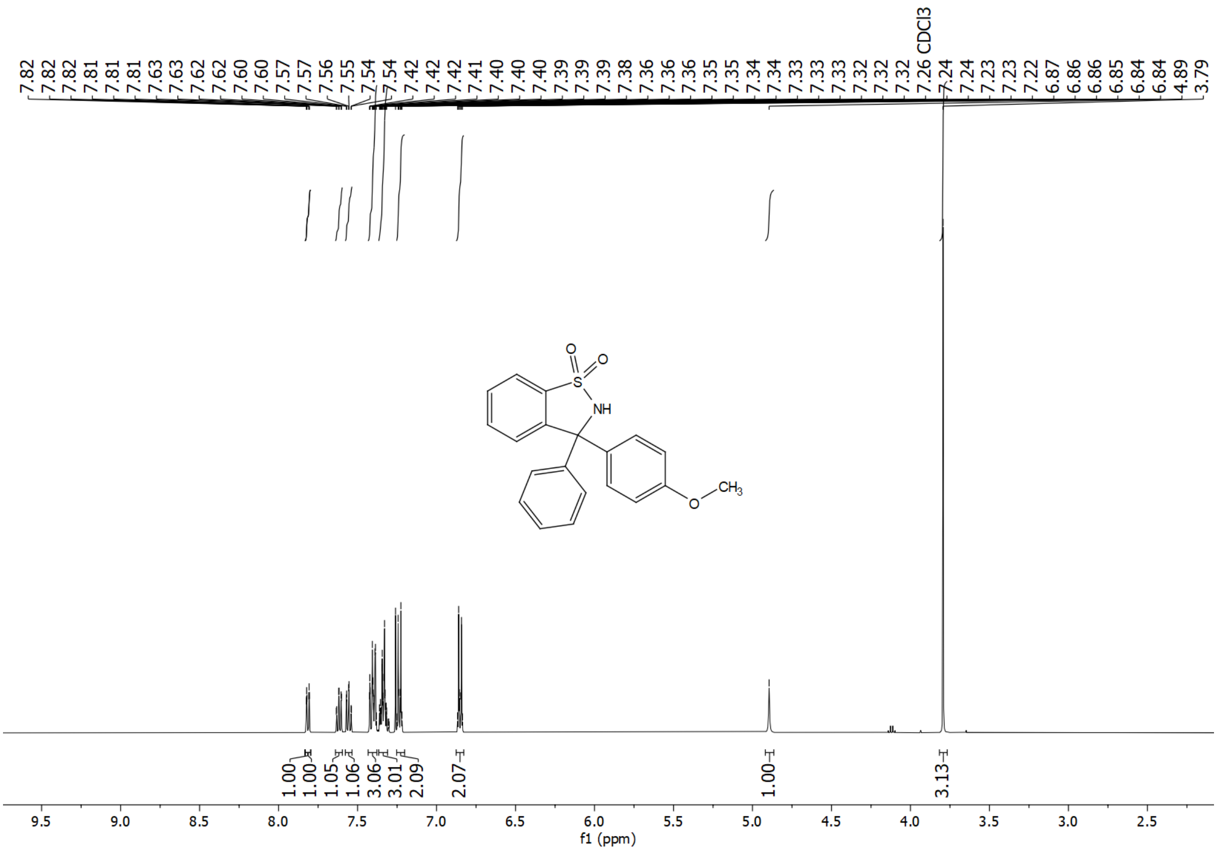

^{13}C{^{1}H} NMR (126 MHz, $CDCl_3$) of **P3k**

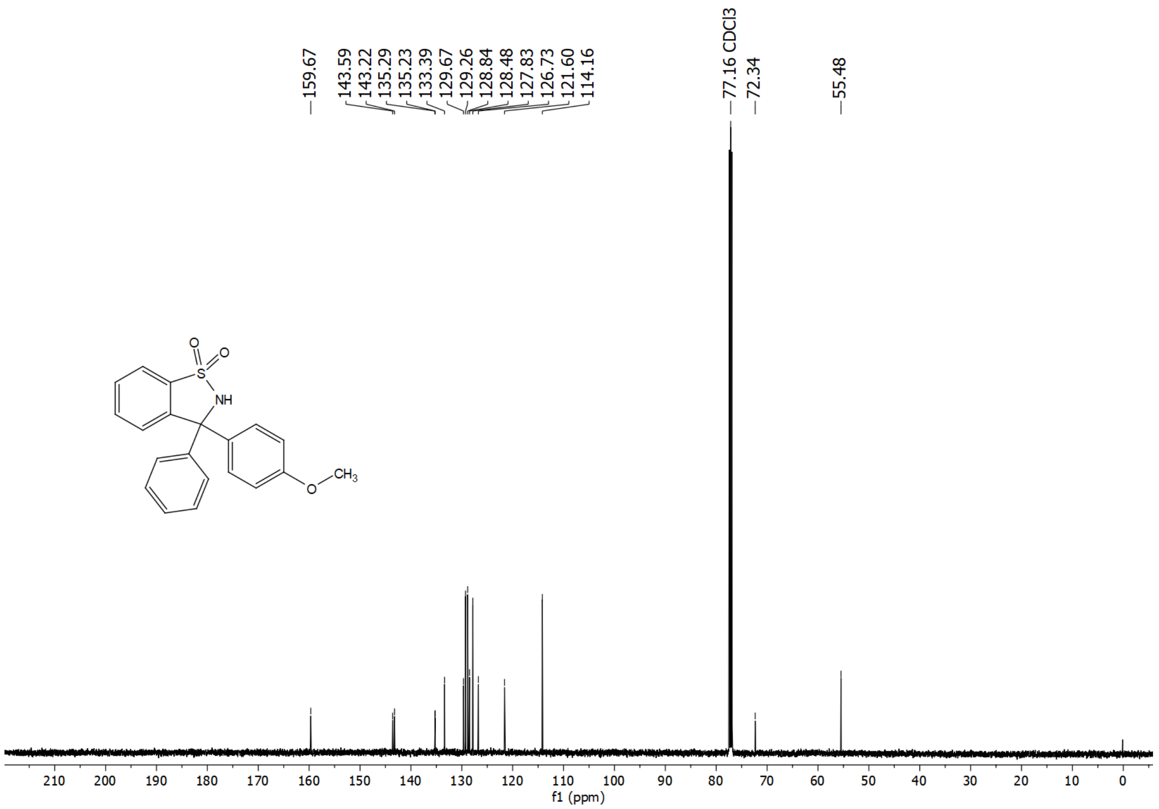

^{1}H NMR (500 MHz, CD_3OD) of **P4l**

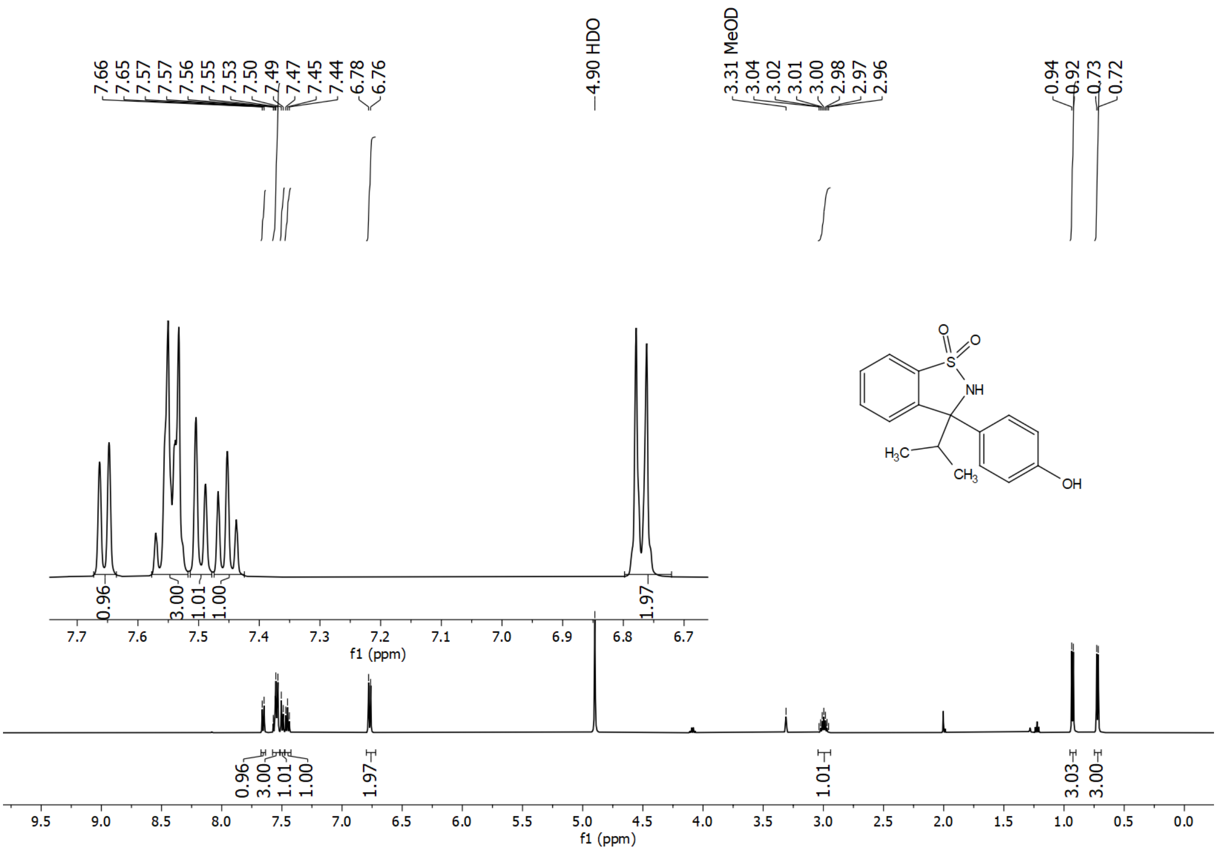

$^{13}C\{^{1}H\}$ NMR (126 MHz, CD_3OD) of **P4l**

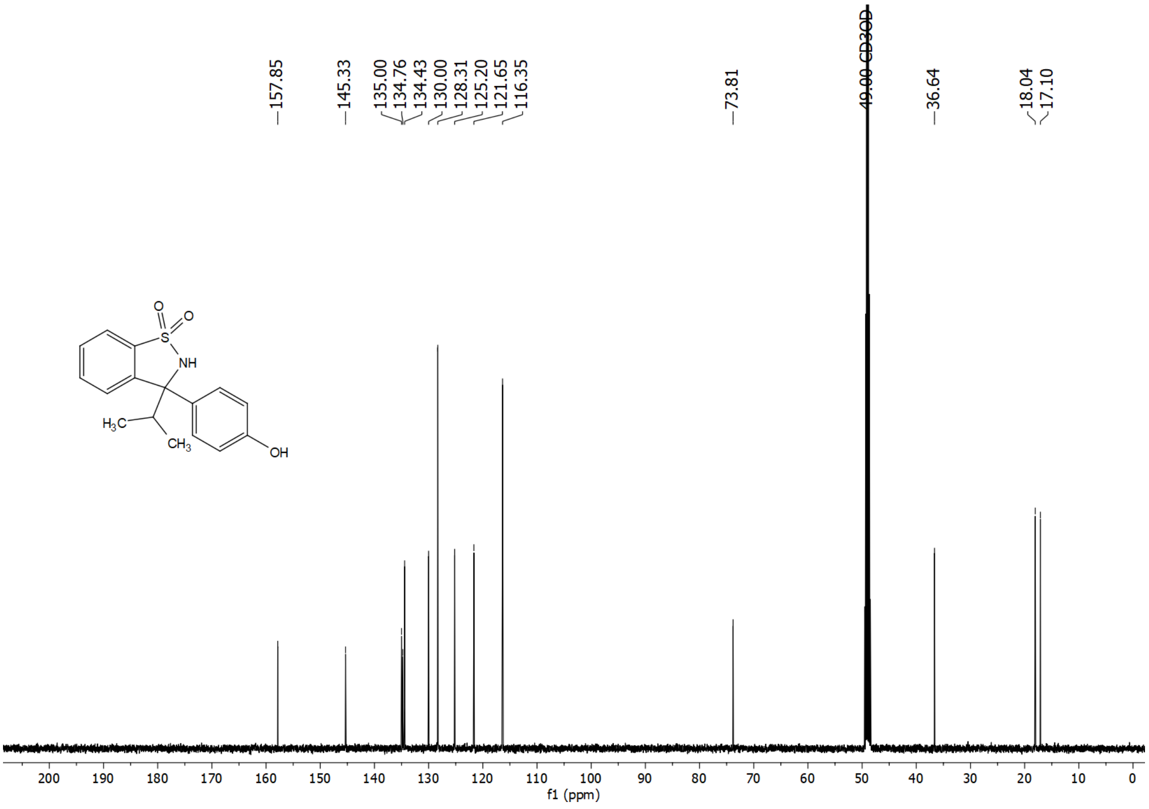

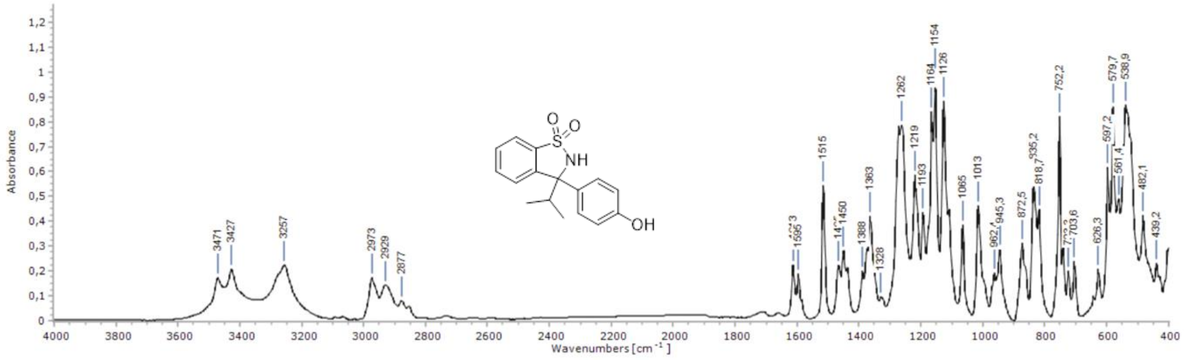

^{1}H NMR (500 MHz, $CDCl_3$) of **P4k**

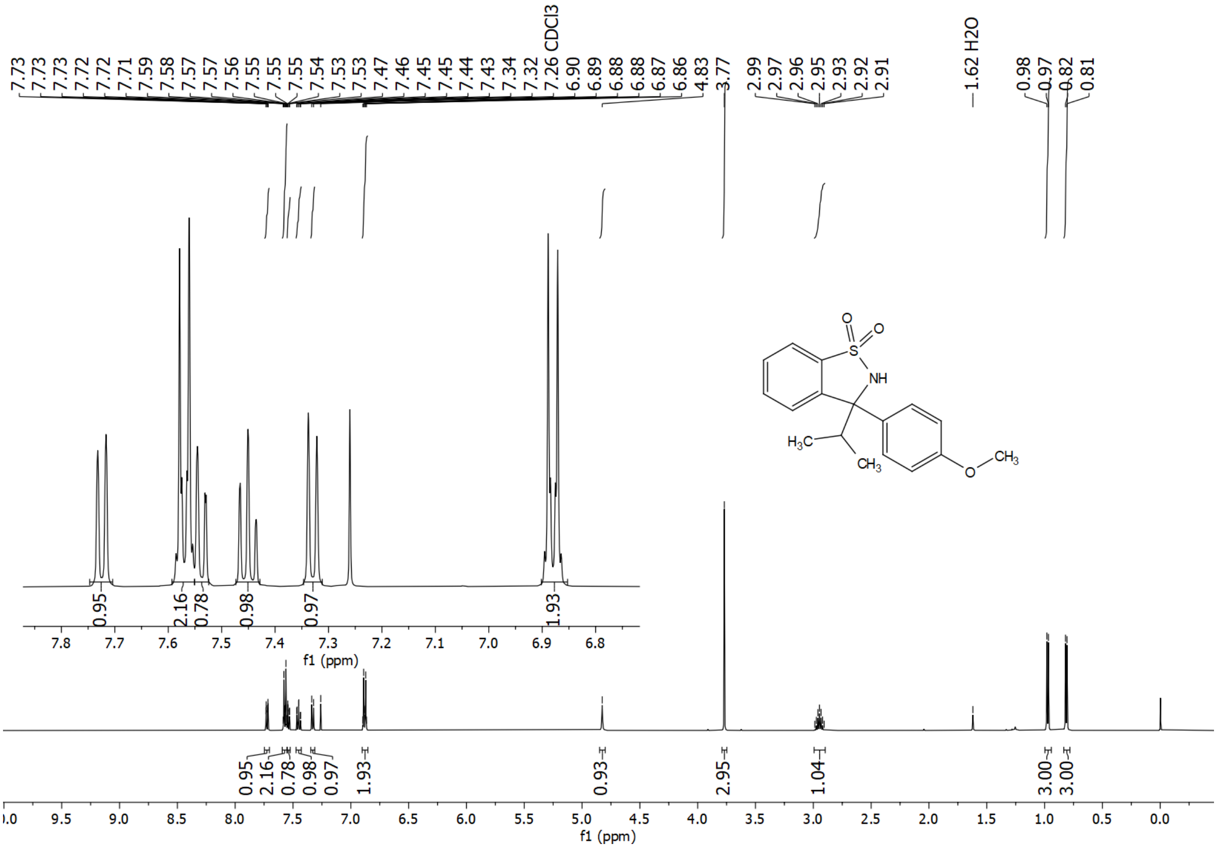

$^{13}C\{^{1}H\}$ NMR (126 MHz, $CDCl_3$) of **P4k**

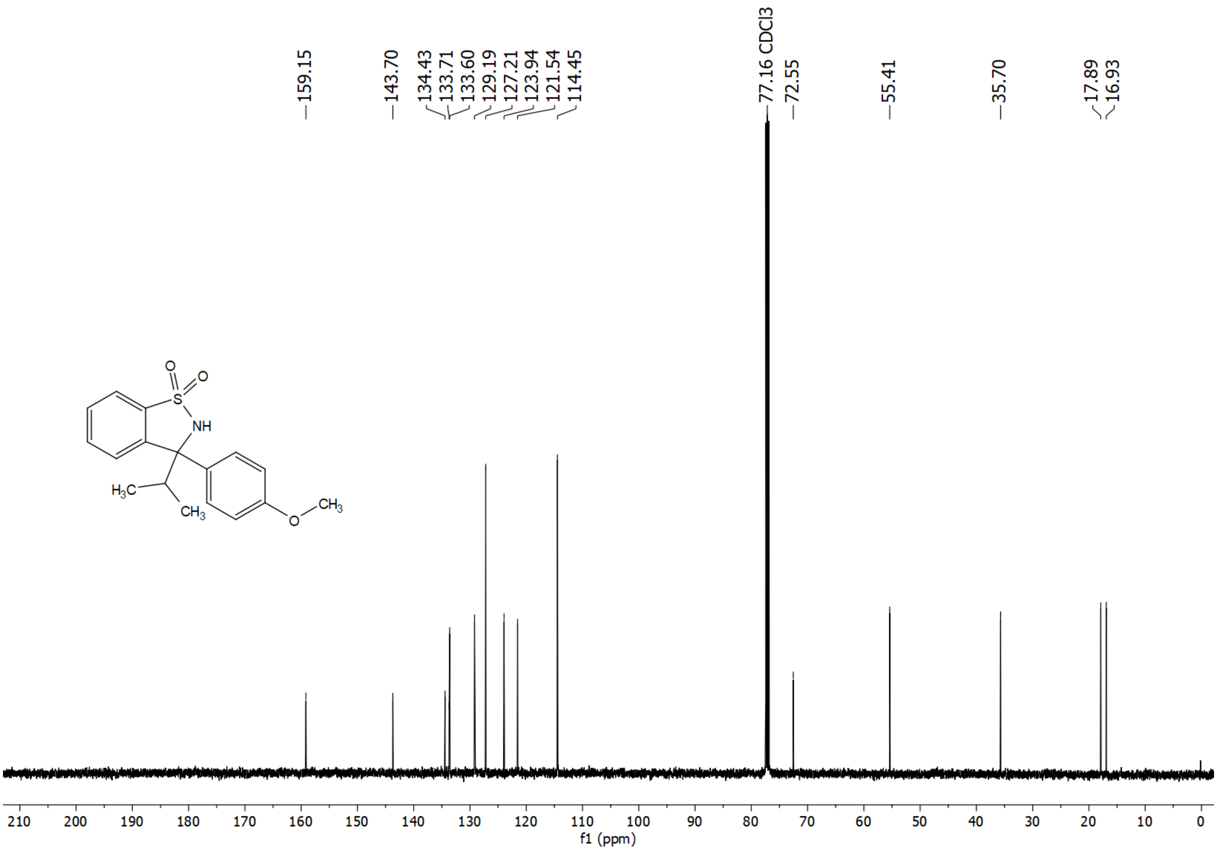

S7-HPLC traces

Column : Chiralpak AD-H
Mobile Phase : hexan/IPA 70/30
Flow Rate : 0,5 ml/min
Note :

Detection :
Temperature :
Pressure : 50 bar

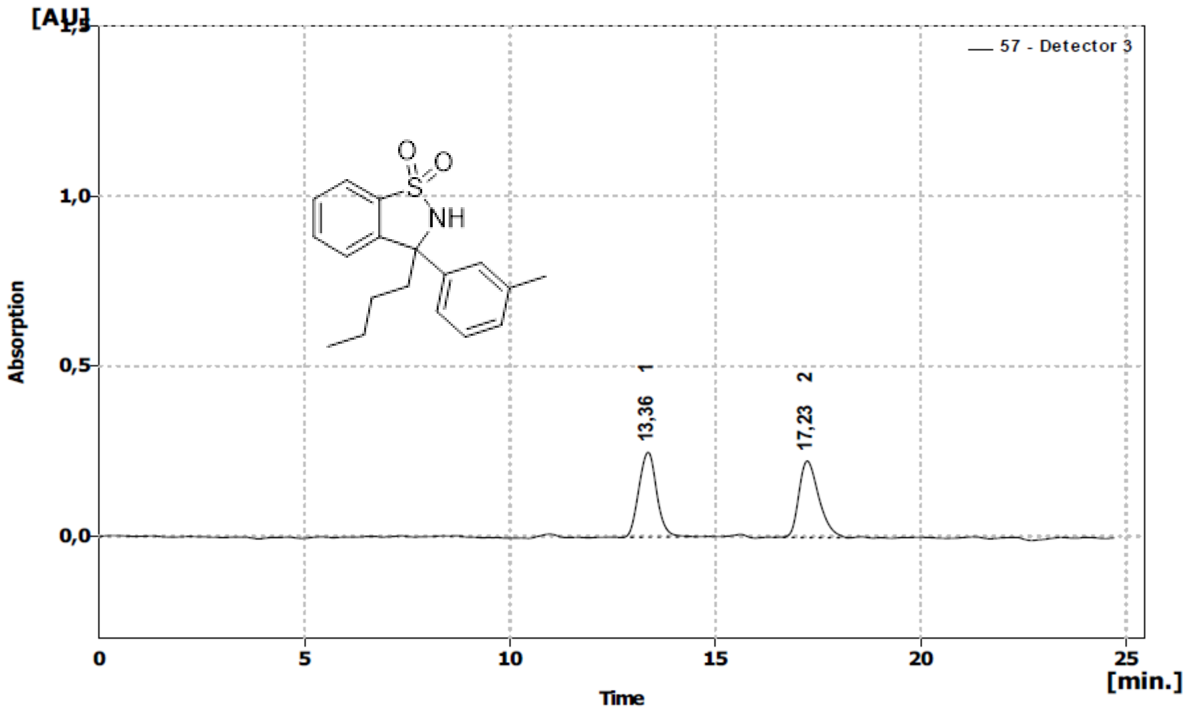

Result Table (Uncal - 57 - Detector 3)

	Reten. Time [min]	Area [mAU.s]	Height [mAU]	Area [%]	Height [%]	W05 [min]	Peak Purity [-]	Compound Name
1	13,358	7572,903	249,270	49,8	52,6	0,47	850	
2	17,233	7638,106	224,791	50,2	47,4	0,53	795	
	Total	15211,009	474,061	100,0	100,0			

Column	: Chiralpak AD-H	Detection	:
Mobile Phase	: hexan/IPA 70/30	Temperature	:
Flow Rate	: 0,8 ml/min	Pressure	: 50 bar
Note	:		

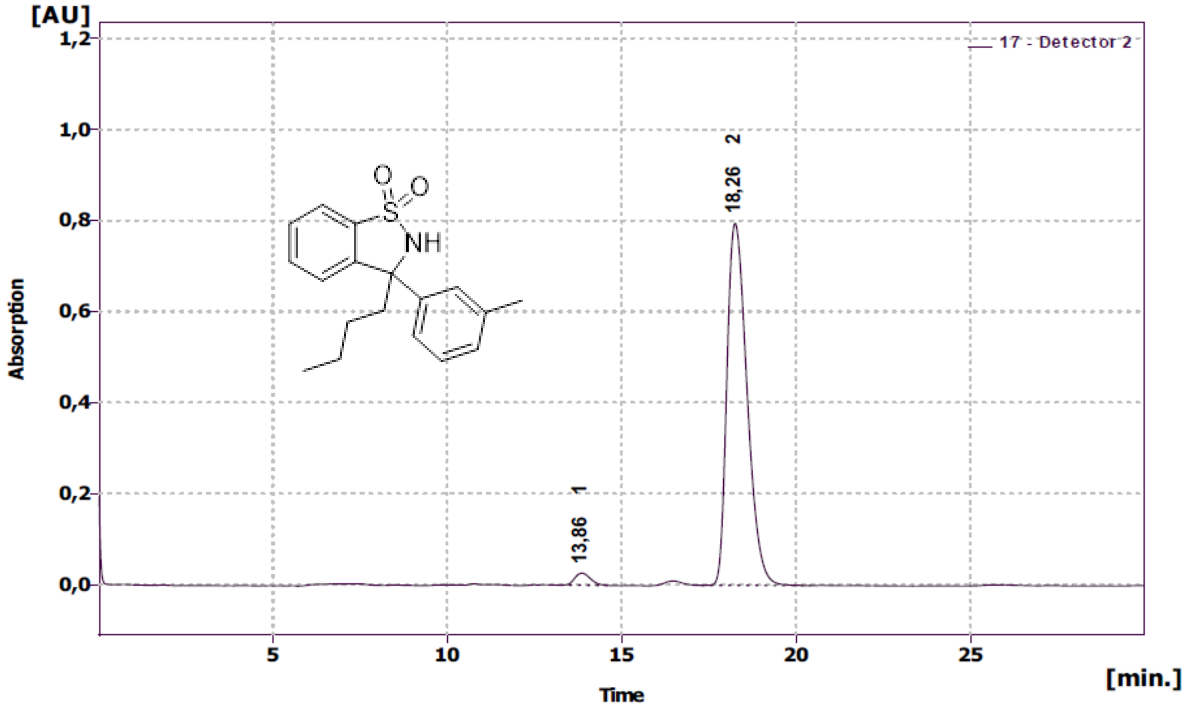

Result Table (Uncal - 17 - Detector 2)

	Reten. Time [min]	Area [mAU.s]	Height [mAU]	Area [%]	Height [%]	W05 [min]	Peak Purity [-]	Compound Name
1	13,858	846,779	26,650	2,6	3,2	0,51	820	
2	18,258	31895,338	795,920	97,4	96,8	0,63	704	
	Total	32742,117	822,570	100,0	100,0			

Column	: Chiralpak AD-H	Detection	:
Mobile Phase	: hexan/IPA 70/30	Temperature	:
Flow Rate	: 0,5 ml/min	Pressure	: 50 bar
Note	:		

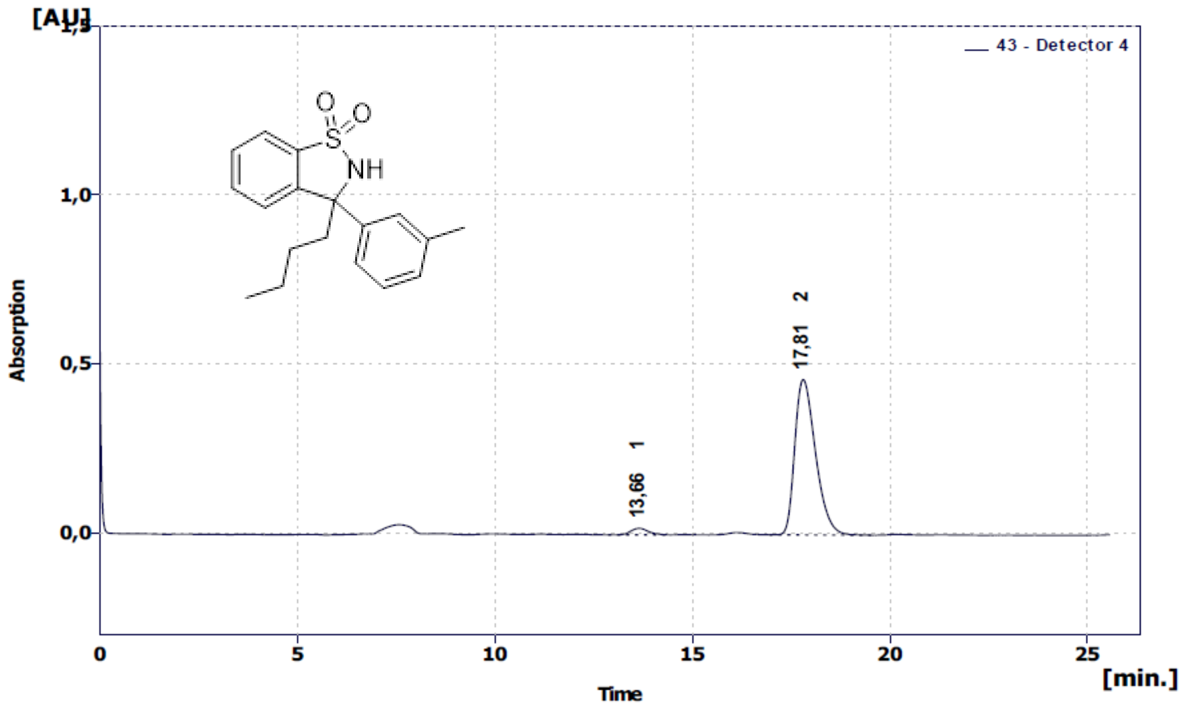

Result Table (Uncal - 43 - Detector 4)

	Reten. Time [min]	Area [mAU.s]	Height [mAU]	Area [%]	Height [%]	W05 [min]	Peak Purity [-]	Compound Name
1	13,658	613,705	19,427	3,5	4,1	0,49	855	
2	17,808	16799,572	459,184	96,5	95,9	0,57	638	
	Total	17413,277	478,611	100,0	100,0			

Column	: Chiralpak AD-H		Detection	:
Mobile Phase	: hexan/IPA 70/30		Temperature	:
Flow Rate	: 0,8 ml/min		Pressure	: 50 bar
Note	:			

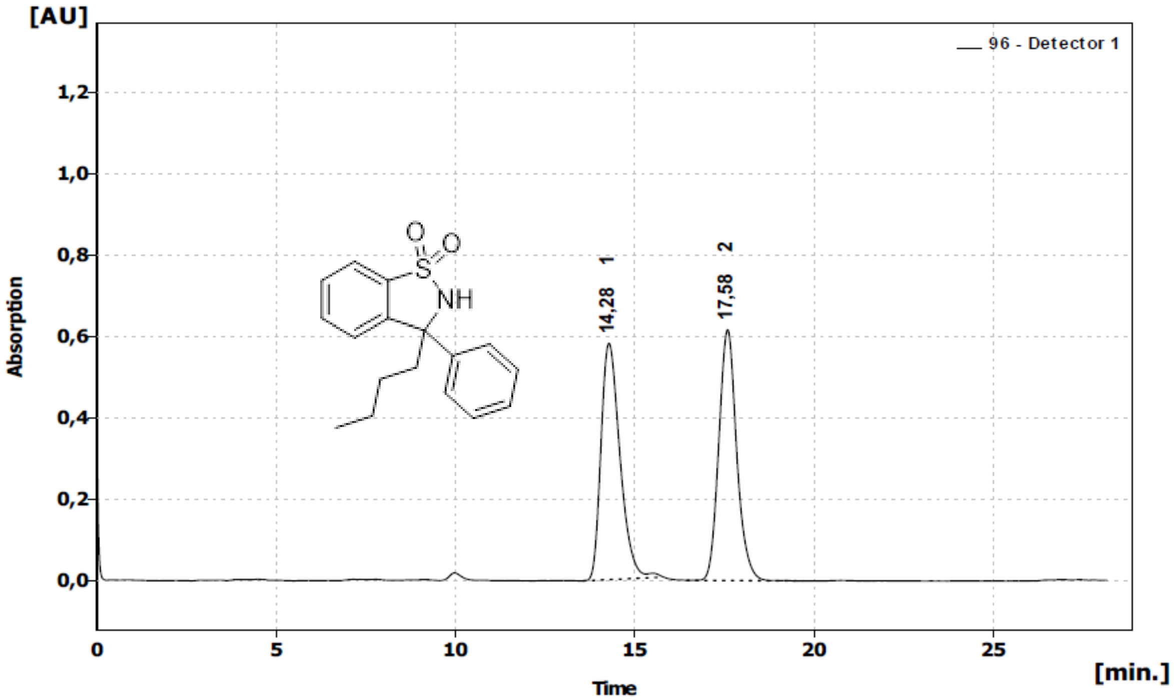

Result Table (Uncal - 96 - Detector 1)

	Reten. Time [min]	Area [mAU.s]	Height [mAU]	Area [%]	Height [%]	W05 [min]	Peak Purity [-]	Compound Name
1	14,283	21465,673	581,399	50,0	48,5	0,57	917	
2	17,583	21423,982	617,293	50,0	51,5	0,53	629	
	Total	42889,655	1198,692	100,0	100,0			

Column	: Chiralpak AD-H	Detection	:
Mobile Phase	: hexan/IPA 70/30	Temperature	:
Flow Rate	: 0,8 ml/min	Pressure	: 50 bar
Note	:		

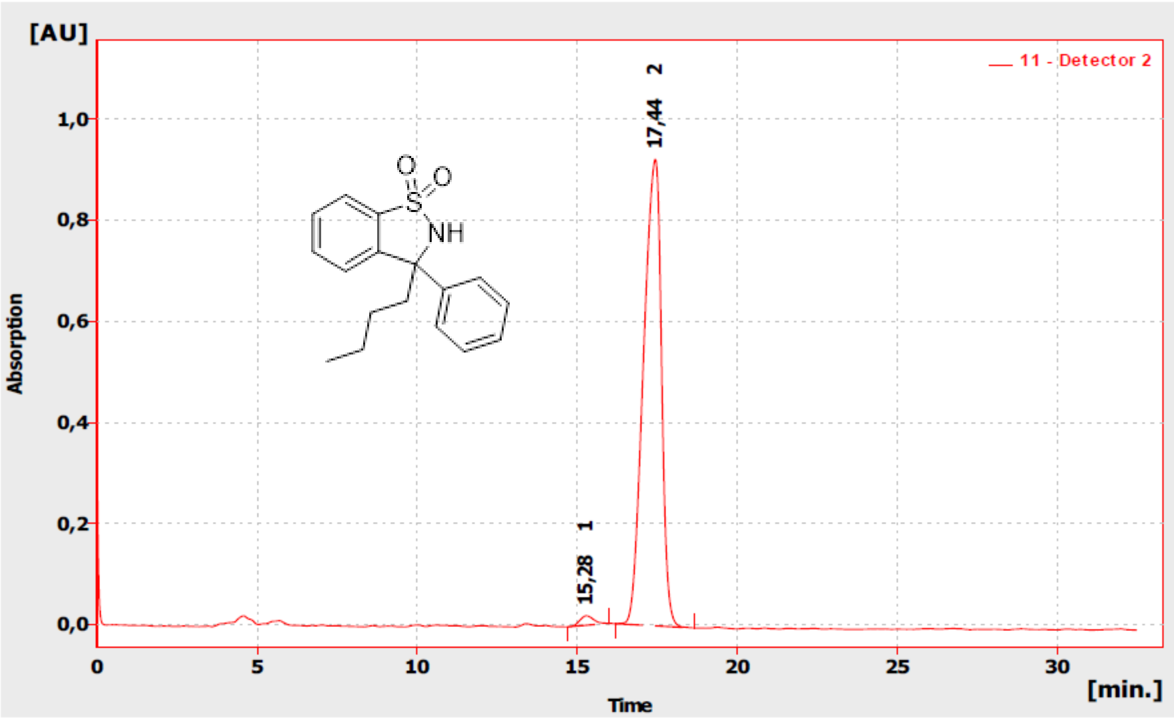

Result Table (Uncal - 11 - Detector 2)

	Reten. Time [min]	Area [mAU.s]	Height [mAU]	Area [%]	Height [%]	W05 [min]	Peak Purity [-]	Compound Name
1	15,275	561,317	18,812	1,5	2,0	0,49	824	
2	17,442	35881,306	922,864	98,5	98,0	0,63	759	
	Total	36442,624	941,676	100,0	100,0			

Column	: Chiralpak AD-H	Detection	:
Mobile Phase	: hexan/IPA 70/30	Temperature	:
Flow Rate	: 1,0 ml/min	Pressure	: 61 bar
Note	:		

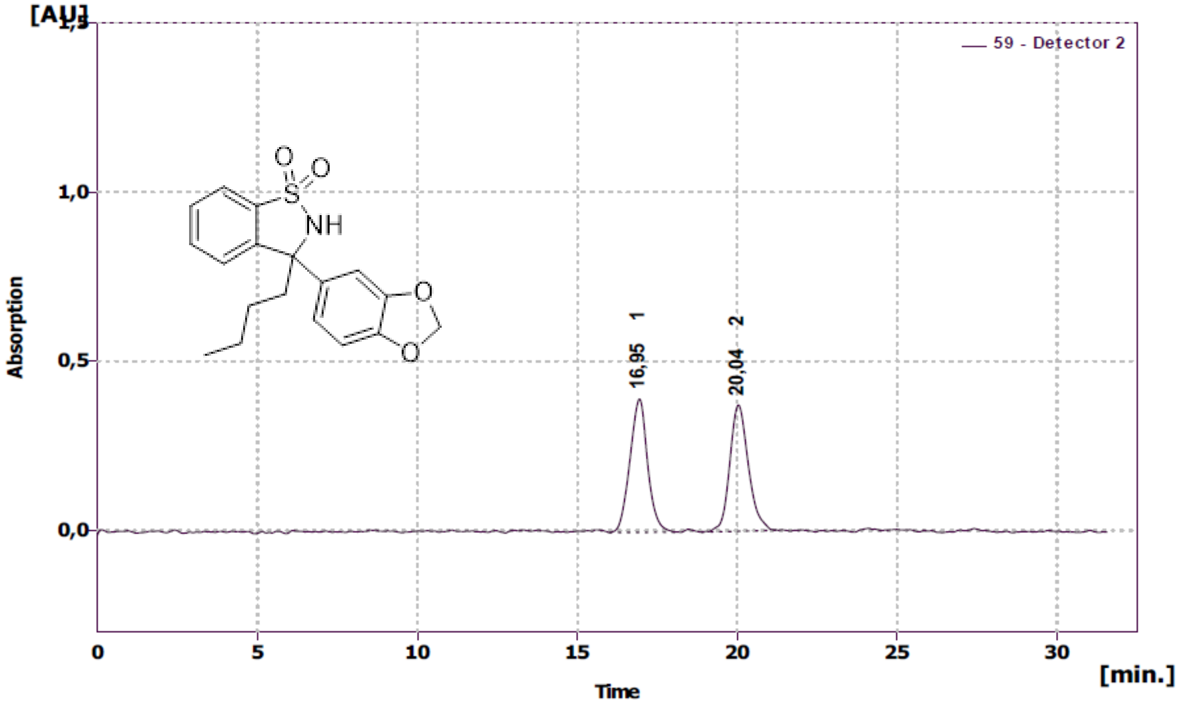

Result Table (Uncal - 59 - Detector 2)

	Reten. Time [min]	Area [mAU.s]	Height [mAU]	Area [%]	Height [%]	W05 [min]	Peak Purity [-]	Compound Name
1	16,950	15248,031	393,901	50,2	51,4	0,59	724	
2	20,042	15153,531	372,903	49,8	48,6	0,62	797	
	Total	30401,562	766,804	100,0	100,0			

Column	: Chiralpak AD-H		Detection	:	
Mobile Phase	: hexan/IPA 70/30		Temperature	:	
Flow Rate	: 1,0 ml/min		Pressure	: 61 bar	
Note	:				

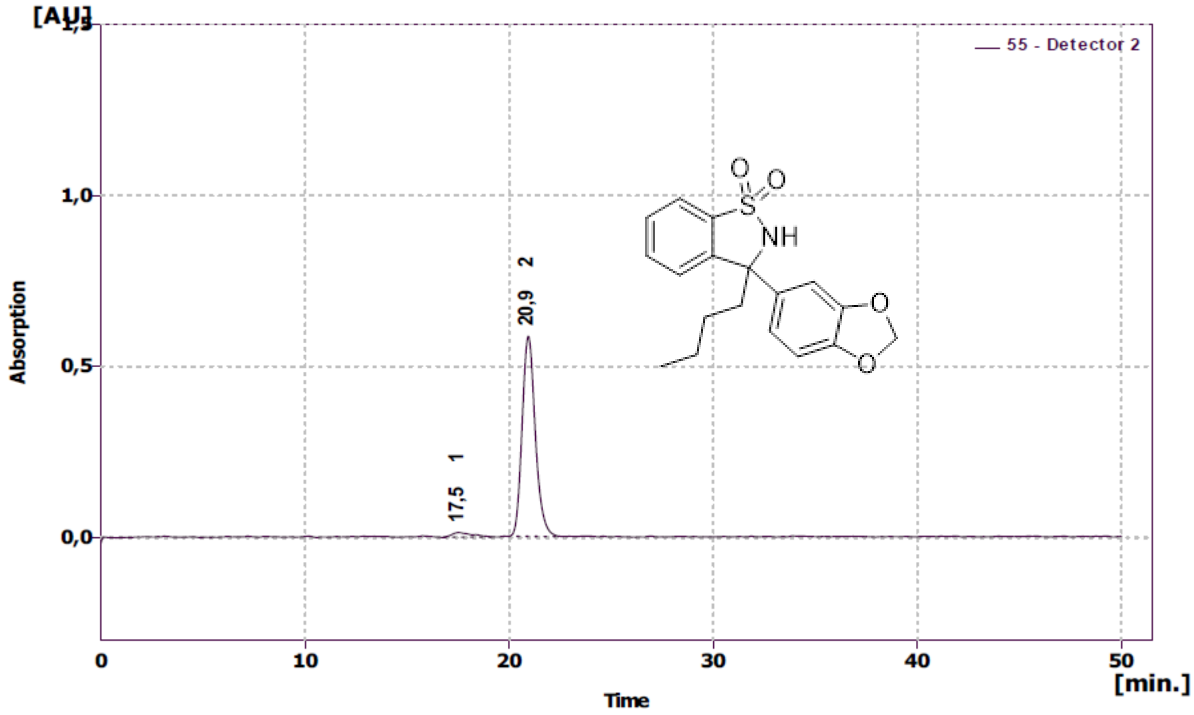

Result Table (Uncal - 55 - Detector 2)

	Reten. Time [min]	Area [mAU.s]	Height [mAU]	Area [%]	Height [%]	W05 [min]	Peak Purity [-]	Compound Name
1	17,517	962,561	13,653	3,5	2,3	1,11	984	
2	20,933	26327,244	584,860	96,5	97,7	0,69	856	
	Total	27289,804	598,512	100,0	100,0			

Column	: Chiralpak AD-H	Detection	:
Mobile Phase	: hexan/IPA 70/30	Temperature	:
Flow Rate	: 0,5 ml/min	Pressure	: 31 bar
Note	:		

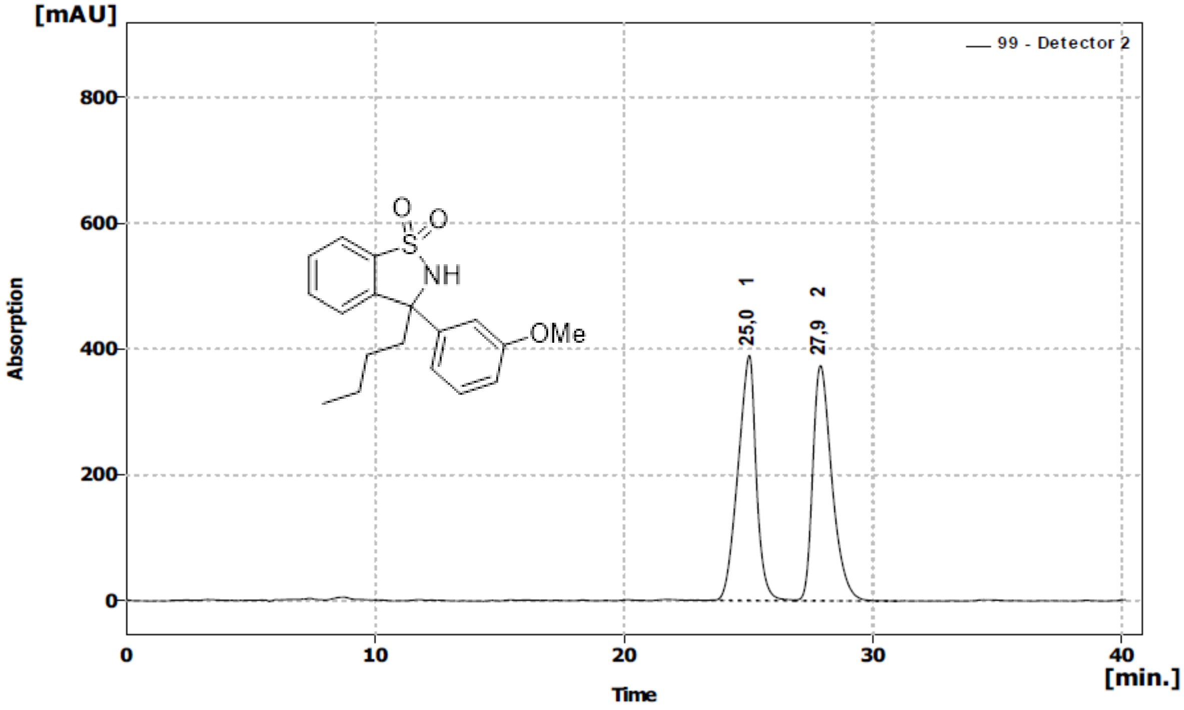

Result Table (Uncal - 99 - Detector 2)

	Reten. Time [min]	Area [mAU.s]	Height [mAU]	Area [%]	Height [%]	W05 [min]	Peak Purity [-]	Compound Name
1	25,025	19907,755	389,471	49,9	51,0	0,78	868	
2	27,892	20024,634	373,506	50,1	49,0	0,82	839	
	Total	39932,389	762,977	100,0	100,0			

Column : Chiralpak AD-H
Mobile Phase : hexan/IPA 70/30
Flow Rate : 0,5 ml/min
Note :

Detection :
Temperature :
Pressure : 31 bar

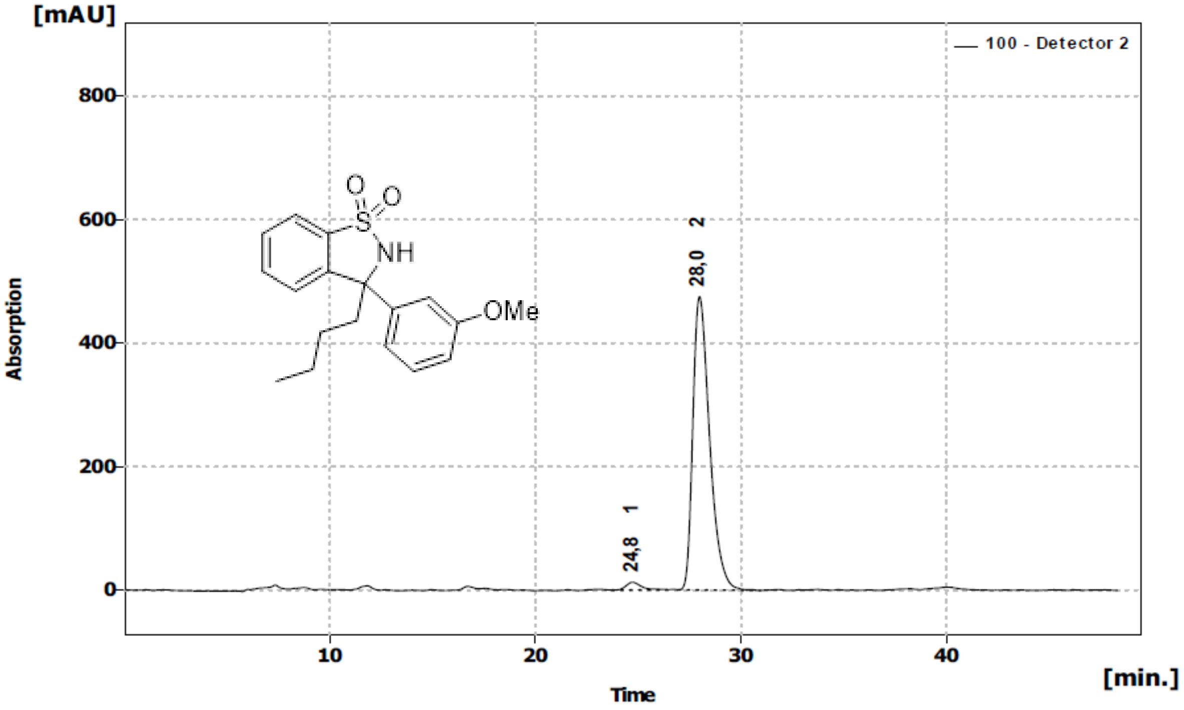

Result Table (Uncal - 100 - Detector 2)

	Reten. Time [min]	Area [mAU.s]	Height [mAU]	Area [%]	Height [%]	W05 [min]	Peak Purity [-]	Compound Name
1	24,758	633,275	12,547	2,3	2,6	0,74	987	
2	27,983	26966,143	475,131	97,7	97,4	0,88	825	
	Total	27599,418	487,678	100,0	100,0			

Column	: Chiralpak AD-H		Detection	:
Mobile Phase	: hexan/IPA 70/30		Temperature	:
Flow Rate	: 0,5 ml/min		Pressure	: 31 bar
Note	:			

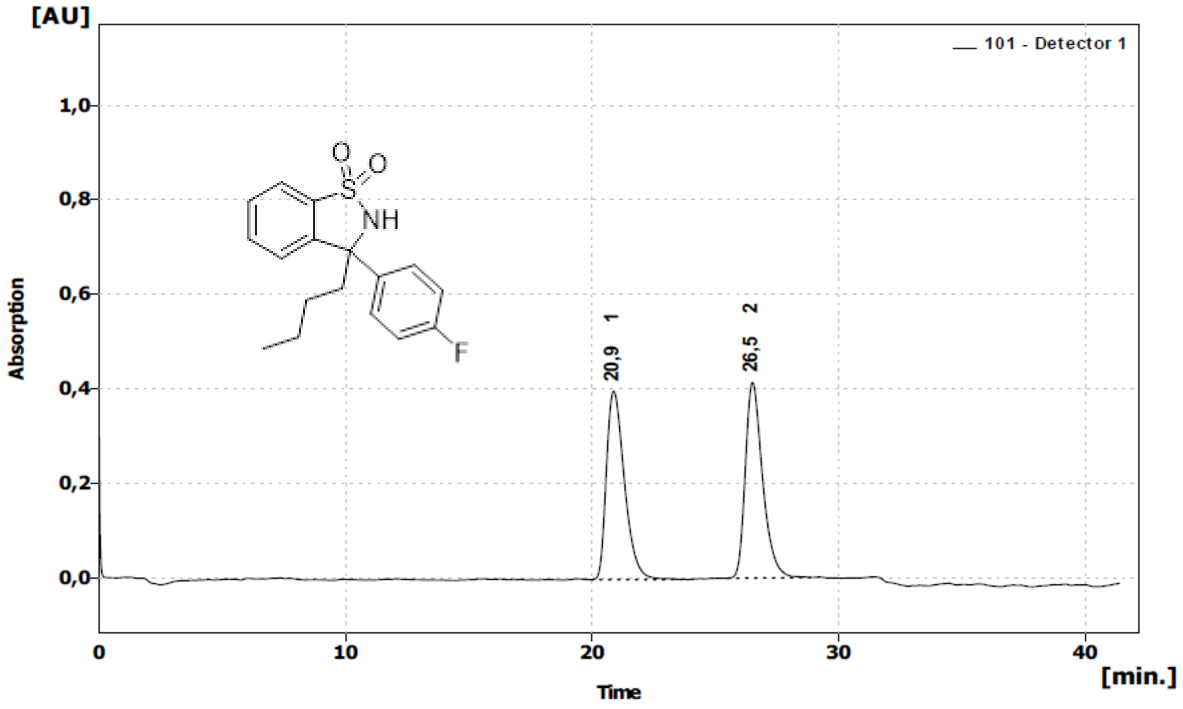

Result Table (Uncal - 101 - Detector 1)

	Reten. Time [min]	Area [mAU.s]	Height [mAU]	Area [%]	Height [%]	W05 [min]	Peak Purity [-]	Compound Name
1	20,875	20365,023	398,829	50,2	49,1	0,79	852	
2	26,508	20176,921	413,803	49,8	50,9	0,74	902	
	Total	40541,943	812,632	100,0	100,0			

Column	: Chiralpak AD-H	Detection	:
Mobile Phase	: hexan/IPA 70/30	Temperature	:
Flow Rate	: 0,5 ml/min	Pressure	: 31 bar
Note	:		

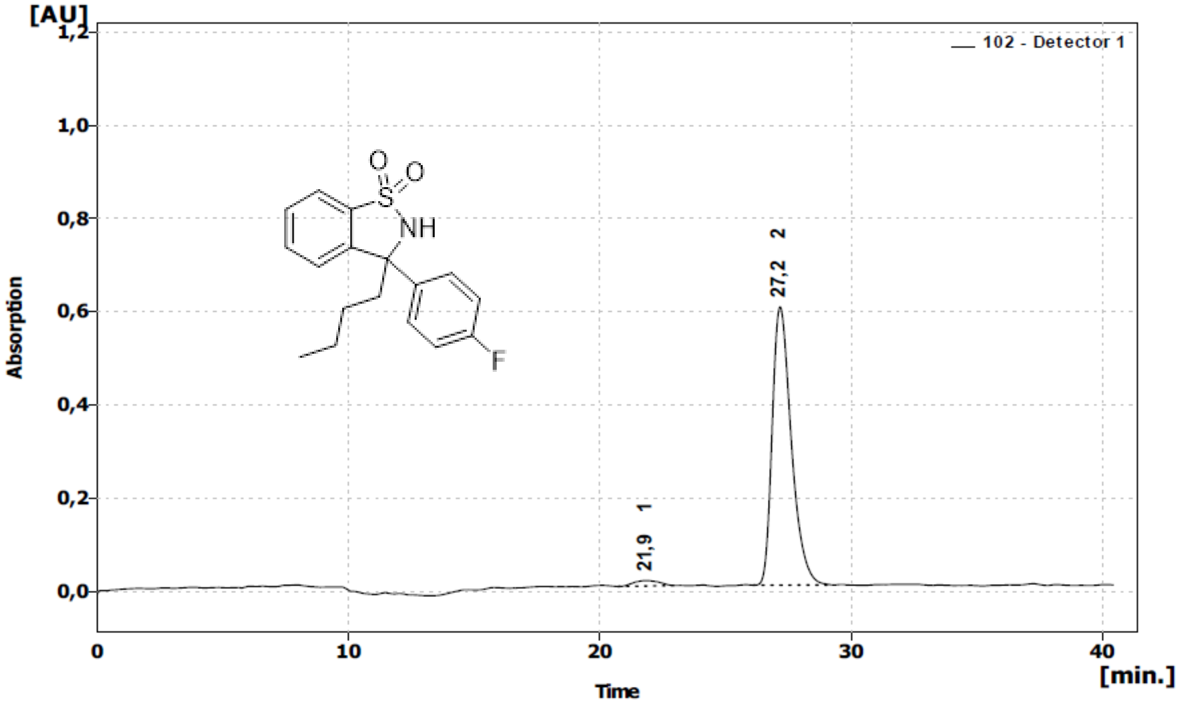

Result Table (Uncal - 102 - Detector 1)

	Reten. Time [min]	Area [mAU.s]	Height [mAU]	Area [%]	Height [%]	W05 [min]	Peak Purity [-]	Compound Name
1	21,875	833,872	11,724	2,6	1,9	1,24	994	
2	27,175	31635,844	596,952	97,4	98,1	0,81	920	
	Total	32469,717	608,676	100,0	100,0			

Column	: Chiralpak IA	Detection	:
Mobile Phase	: hexan/IPA 70/30	Temperature	:
Flow Rate	: 0,5 ml/min	Pressure	: 28 bar
Note	:		

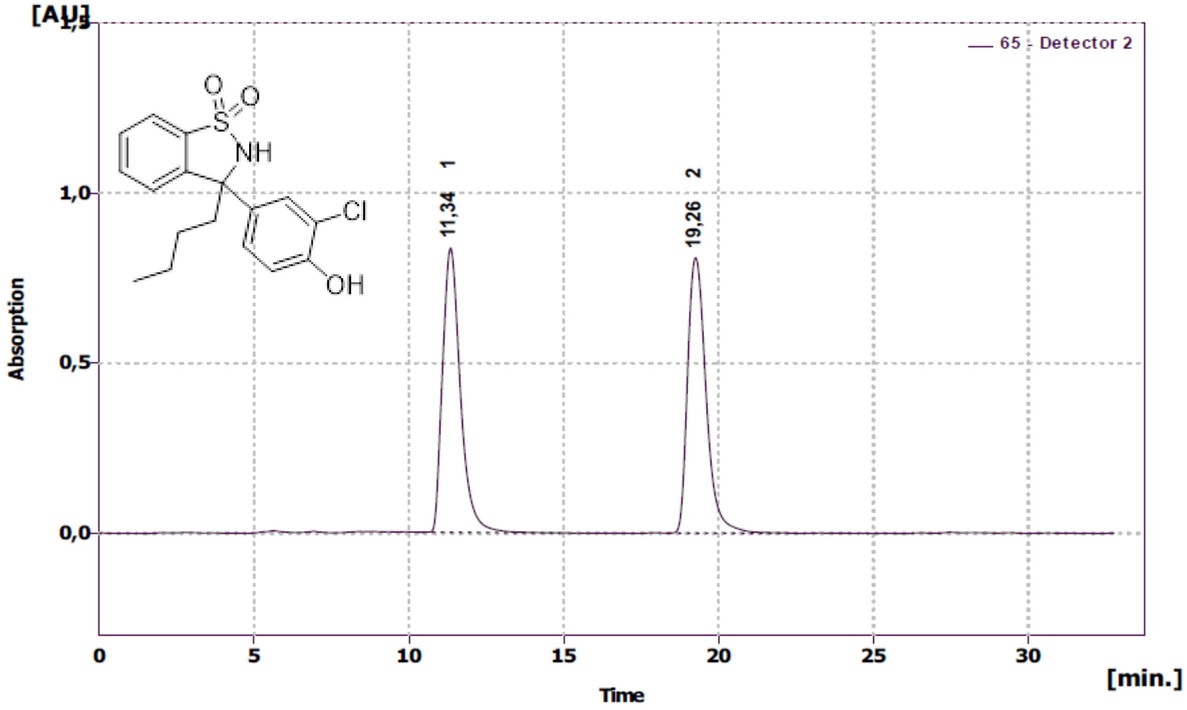

Result Table (Uncal - 65 - Detector 2)

	Reten. Time [min]	Area [mAU.s]	Height [mAU]	Area [%]	Height [%]	W05 [min]	Peak Purity [-]	Compound Name
1	11,342	33508,729	835,170	49,9	50,8	0,61	783	
2	19,258	33682,079	809,055	50,1	49,2	0,63	733	
	Total	67190,808	1644,225	100,0	100,0			

Column	: Chiralpak IA	Detection	:
Mobile Phase	: hexan/IPA 70/30	Temperature	:
Flow Rate	: 0,5 ml/min	Pressure	: 28 bar
Note	:		

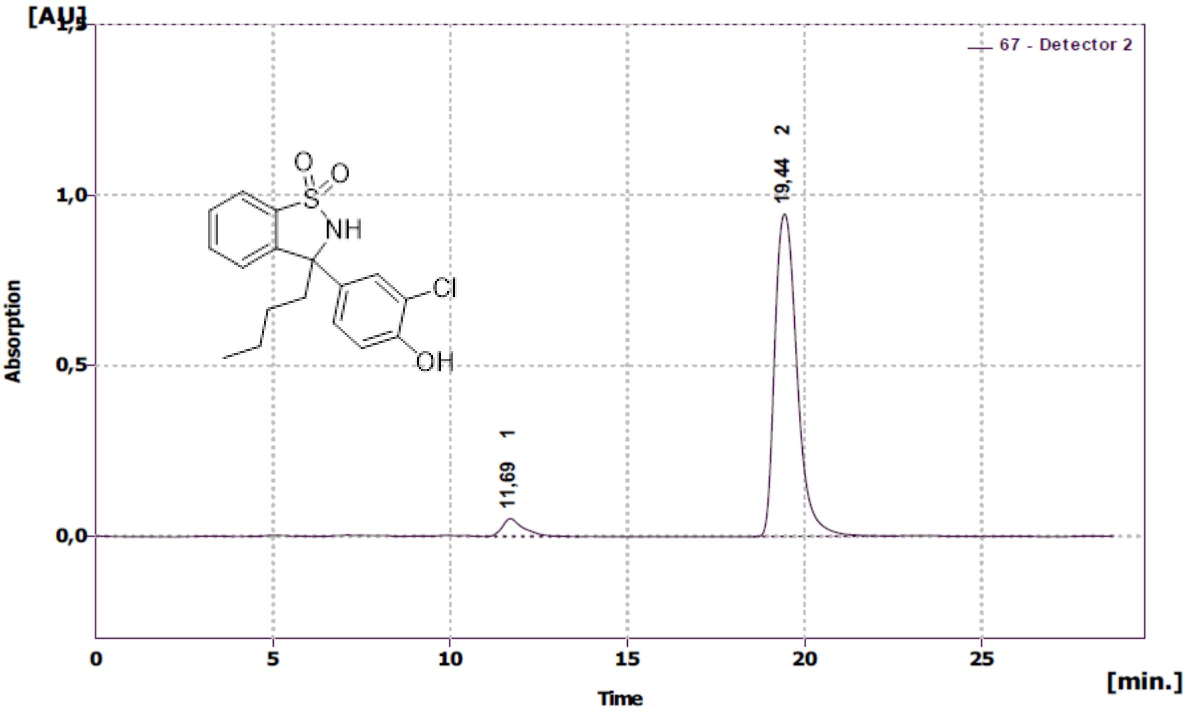

Result Table (Uncal - 67 - Detector 2)

	Reten. Time [min]	Area [mAU.s]	Height [mAU]	Area [%]	Height [%]	W05 [min]	Peak Purity [-]	Compound Name
1	11,692	2268,072	52,781	5,2	5,3	0,60	744	
2	19,442	41311,253	945,166	94,8	94,7	0,68	738	
	Total	43579,325	997,947	100,0	100,0			

Column	: Chiralpak IA	Detection	:
Mobile Phase	: 70/30 hexan/IPA	Temperature	:
Flow Rate	: 1,0 ml/min	Pressure	: 53 bar
Note	:		

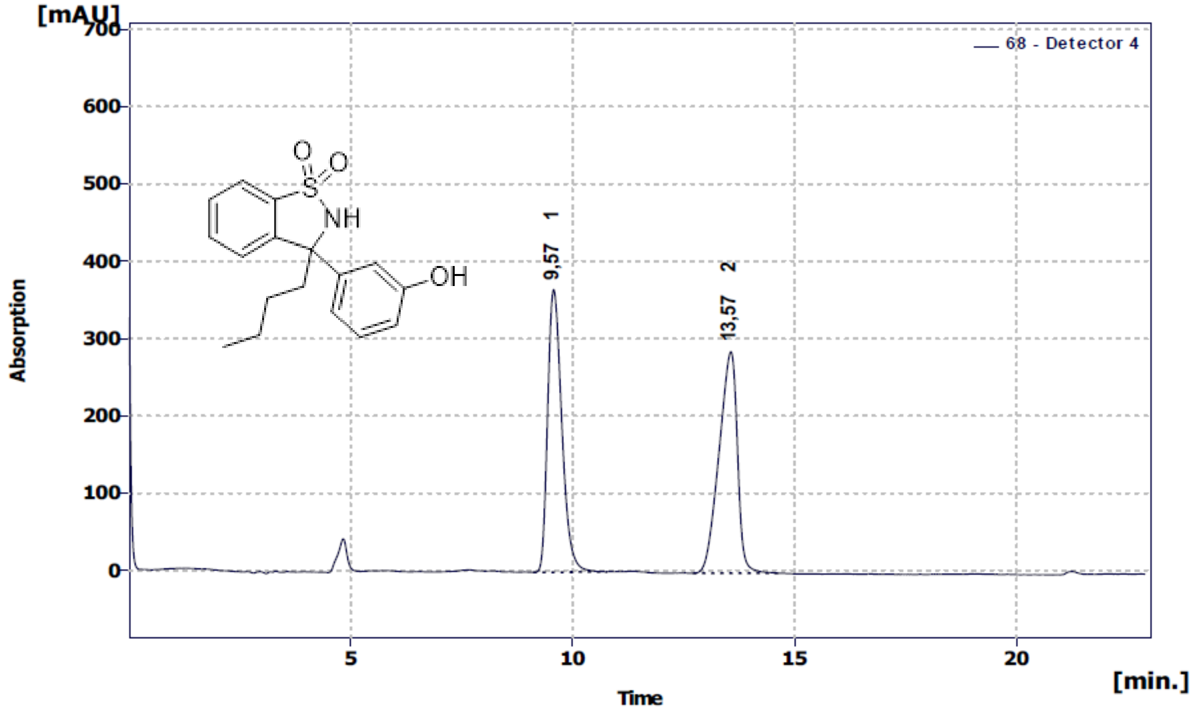

Result Table (Uncal - 68 - Detector 4)

	Reten. Time [min]	Area [mAU.s]	Height [mAU]	Area [%]	Height [%]	W05 [min]	Peak Purity [-]	Compound Name
1	9,575	8123,474	365,663	49,5	56,1	0,35	692	
2	13,567	8289,326	286,594	50,5	43,9	0,46	707	
	Total	16412,800	652,257	100,0	100,0			

Column : Chiralpak IA
Mobile Phase : 70/30 hexan/IPA
Flow Rate : 1,0 ml/min
Note :

Detection :
Temperature :
Pressure : 53 bar

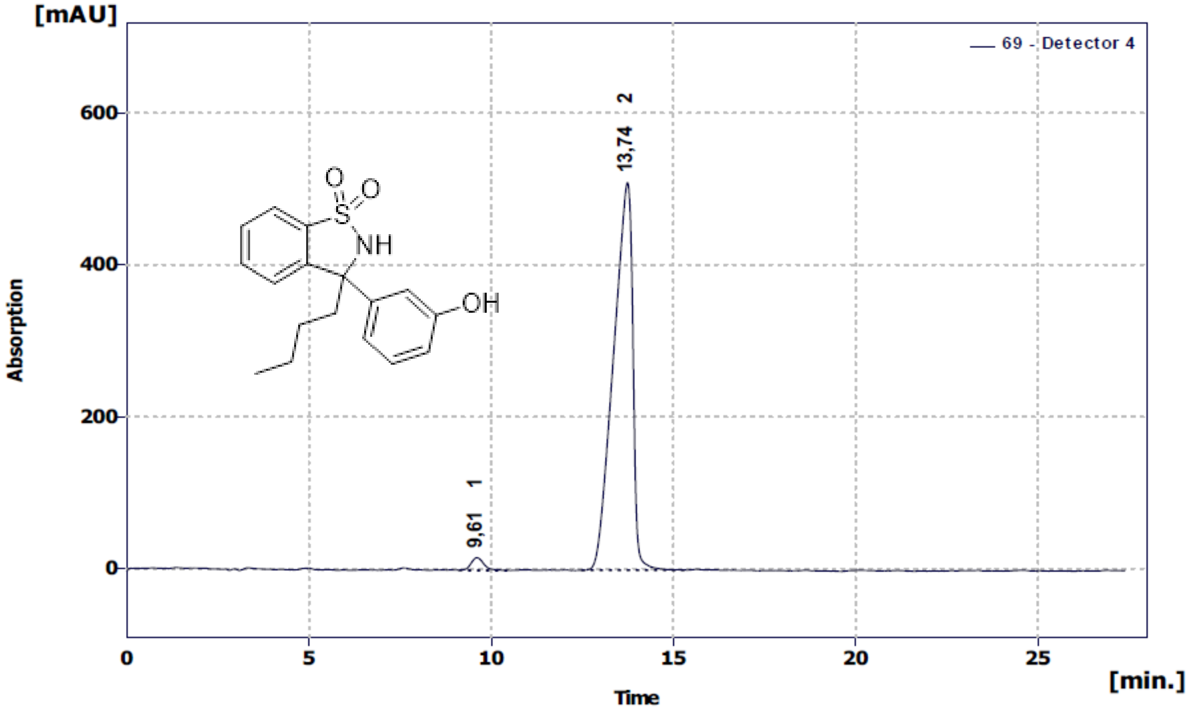

Result Table (Uncal - 69 - Detector 4)

	Reten. Time [min]	Area [mAU.s]	Height [mAU]	Area [%]	Height [%]	W05 [min]	Peak Purity [-]	Compound Name
1	9,608	414,583	16,733	2,2	3,2	0,38	602	
2	13,742	18821,564	510,479	97,8	96,8	0,60	643	
	Total	19236,146	527,212	100,0	100,0			

Column	: Chiralpak IA		Detection	:	
Mobile Phase	: Hexan/IPA 70/30		Temperature	:	
Flow Rate	: 0,5 ml/min		Pressure	: 28 bar	
Note	:				

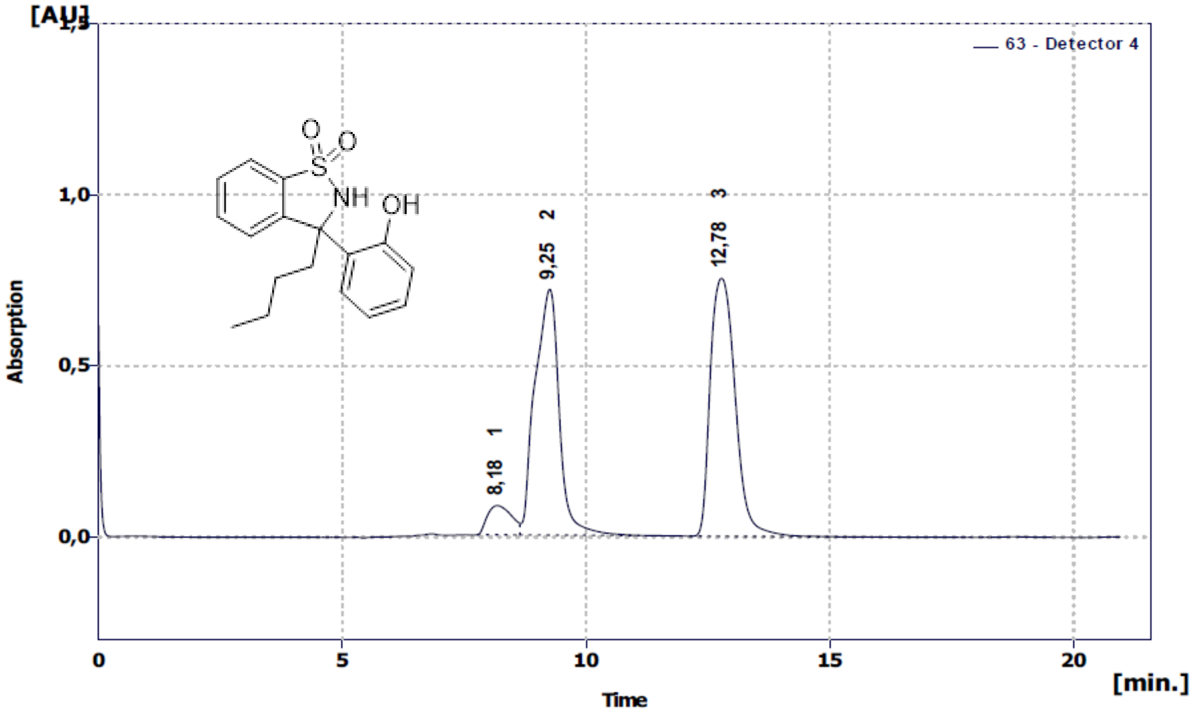

Result Table (Uncal - 63 - Detector 4)

	Reten. Time [min]	Area [mAU.s]	Height [mAU]	Area [%]	Height [%]	W05 [min]	Peak Purity [-]	Compound Name
1	8,175	2947,190	85,487	5,4	5,5	0,63	969	
2	9,250	24894,473	717,697	45,6	46,1	0,57	885	
3	12,775	26809,529	753,623	49,1	48,4	0,57	725	
	Total	54651,192	1556,807	100,0	100,0			

Column	: Chiralpak IA		Detection	:	
Mobile Phase	: Hexan/IPA 70/30		Temperature	:	
Flow Rate	: 0,5 ml/min		Pressure	: 28 bar	
Note	:				

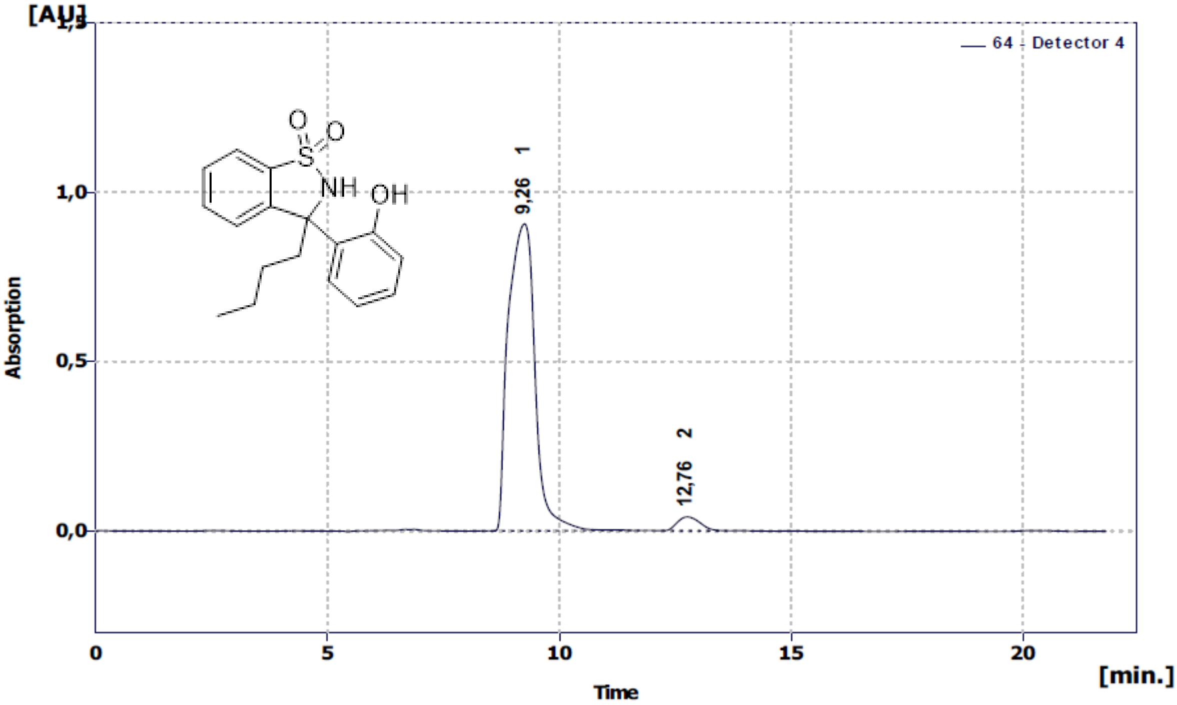

Result Table (Uncal - 64 - Detector 4)

	Reten. Time [min]	Area [mAU.s]	Height [mAU]	Area [%]	Height [%]	W05 [min]	Peak Purity [-]	Compound Name
1	9,258	36270,394	905,731	96,3	95,7	0,67	732	
2	12,758	1393,393	40,911	3,7	4,3	0,57	971	
	Total	37663,787	946,643	100,0	100,0			

Column	: Chiralpak IA	Detection	:
Mobile Phase	: hexan/IPA 70/30	Temperature	:
Flow Rate	: 1,0 ml/min	Pressure	: 54 bar
Note	:		

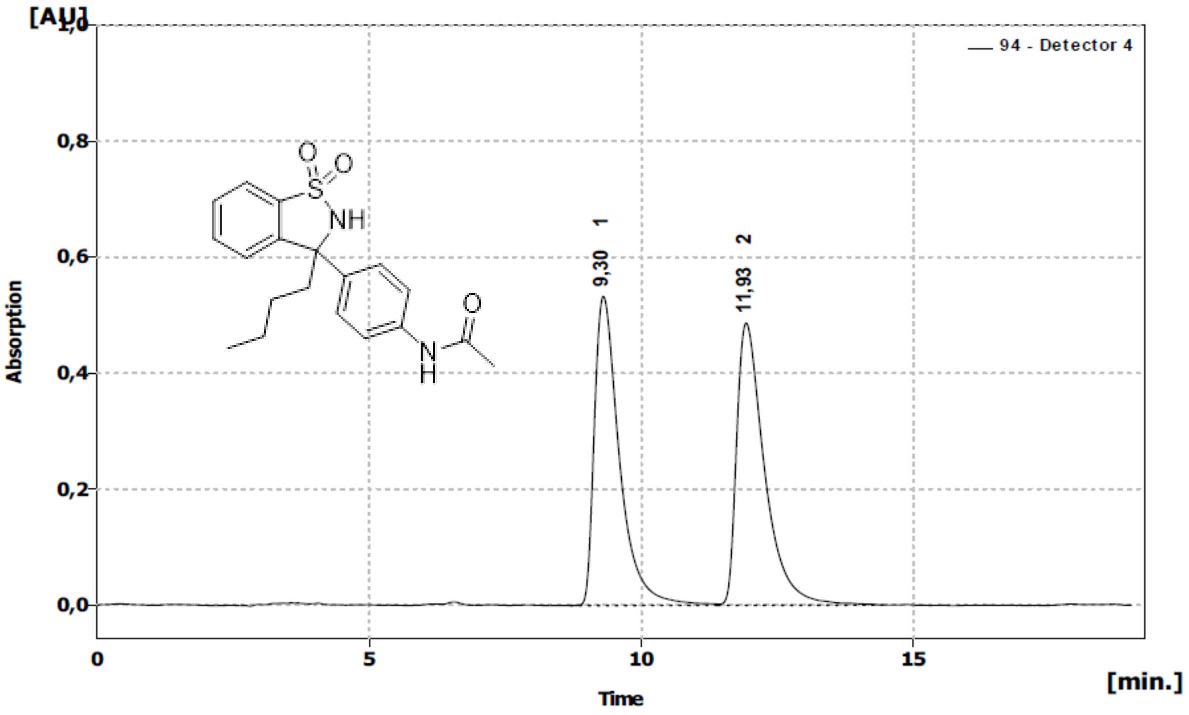

Result Table (Uncal - 94 - Detector 4)

	Reten. Time [min]	Area [mAU.s]	Height [mAU]	Area [%]	Height [%]	W05 [min]	Peak Purity [-]	Compound Name
1	9,300	17160,209	533,010	49,7	52,3	0,47	887	
2	11,925	17379,331	486,734	50,3	47,7	0,53	926	
	Total	34539,540	1019,744	100,0	100,0			

Column	: Chiralpak IA		Detection	:
Mobile Phase	: hexan/IPA 70/30		Temperature	:
Flow Rate	: 1,0 ml/min		Pressure	: 54 bar
Note	:			

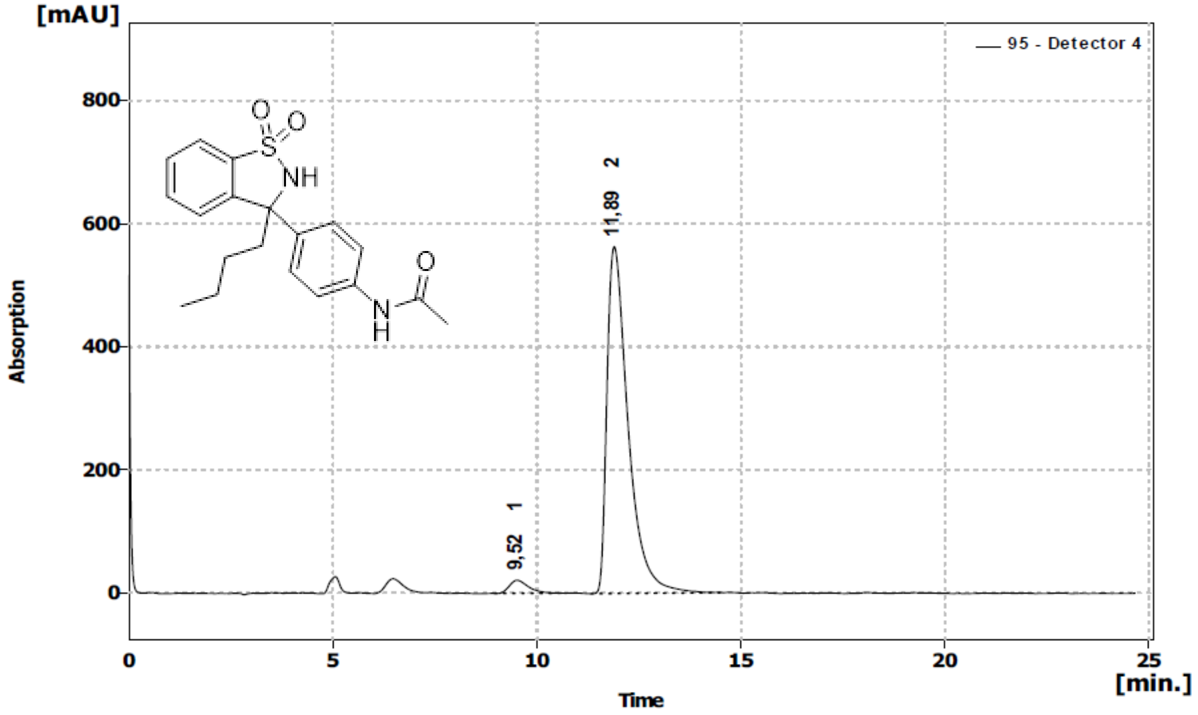

Result Table (Uncal - 95 - Detector 4)

	Reten. Time [min]	Area [mAU.s]	Height [mAU]	Area [%]	Height [%]	W05 [min]	Peak Purity [-]	Compound Name
1	9,517	703,958	21,475	3,3	3,7	0,50	897	
2	11,892	20314,758	563,727	96,7	96,3	0,53	896	
	Total	21018,717	585,202	100,0	100,0			

Column	: Chiralpak IA		Detection	:	
Mobile Phase	: hexan/IPA 70/30		Temperature	:	
Flow Rate	: 1,0 ml/min		Pressure	: 54 bar	
Note	:				

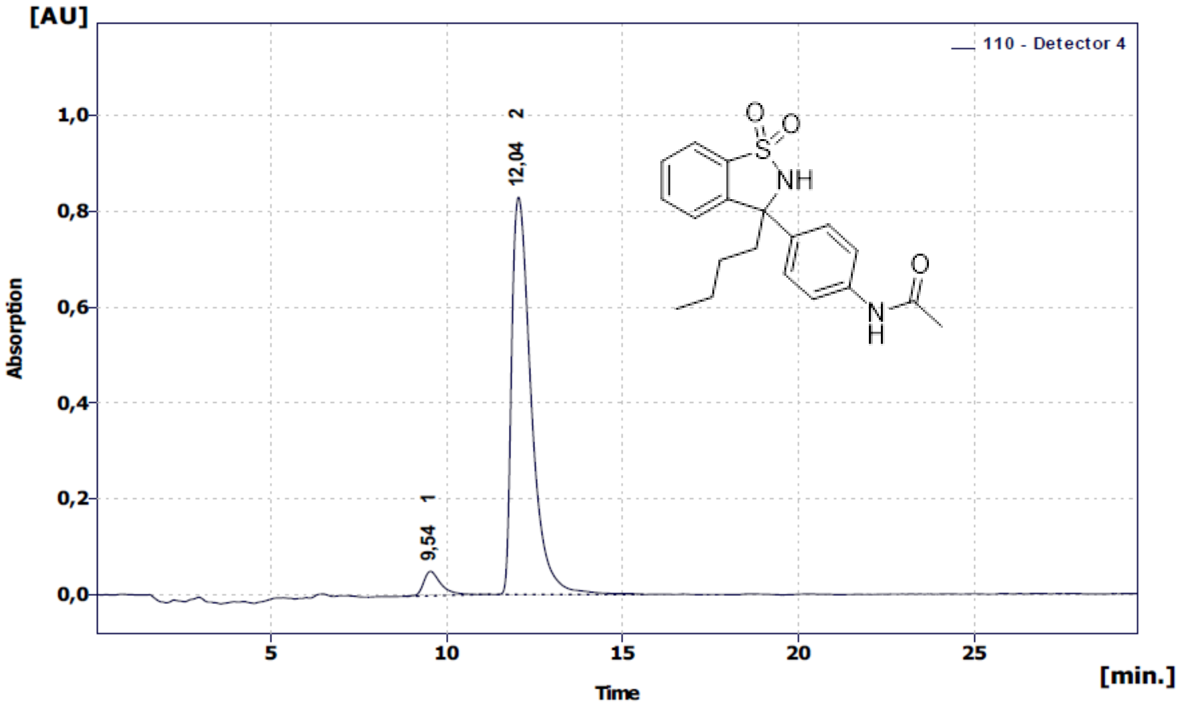

Result Table (Uncal - 110 - Detector 4)

	Reten. Time [min]	Area [mAU.s]	Height [mAU]	Area [%]	Height [%]	W05 [min]	Peak Purity [-]	Compound Name
1	9,542	1766,791	50,851	5,2	5,8	0,51	859	
2	12,042	32156,849	828,193	94,8	94,2	0,58	859	
	Total	33923,640	879,044	100,0	100,0			

Column : Chiralpak AD-H
Mobile Phase : hexan/IPA 70/30
Flow Rate : 0,5 ml/min
Note :

Detection :
Temperature :
Pressure : 31 bar

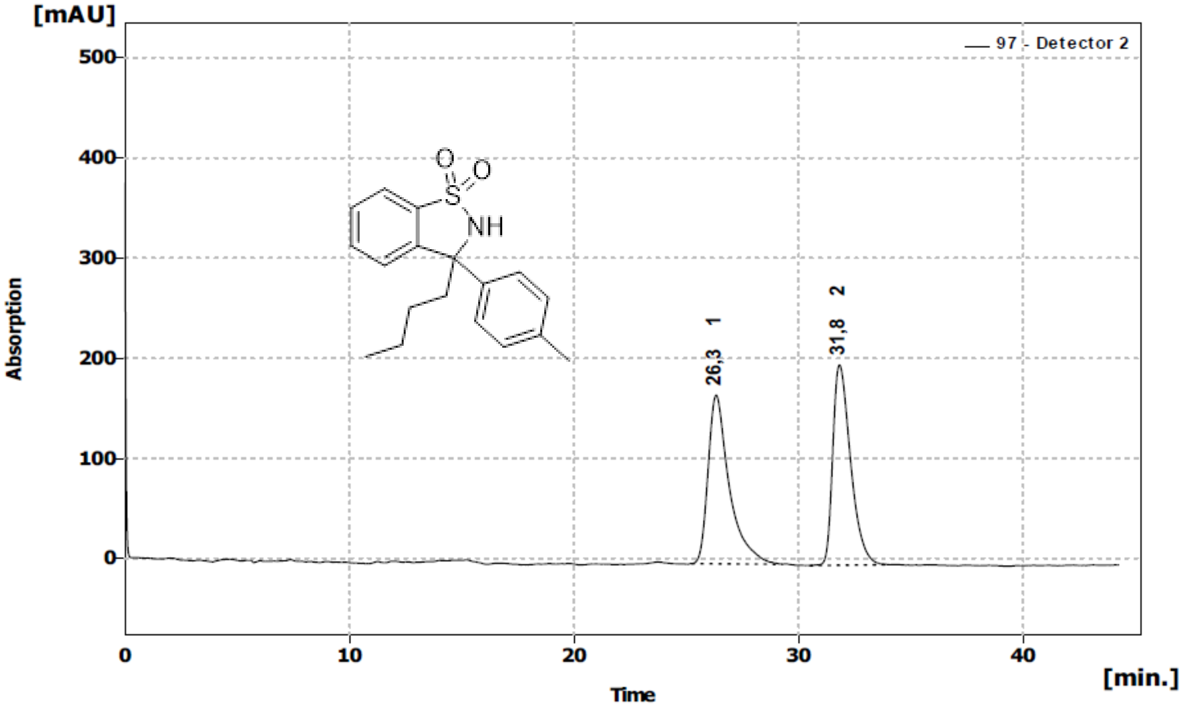

Result Table (Uncal - 97 - Detector 2)

	Reten. Time [min]	Area [mAU.s]	Height [mAU]	Area [%]	Height [%]	W05 [min]	Peak Purity [-]	Compound Name
1	26,325	11110,683	168,454	50,1	45,7	0,93	916	
2	31,825	11068,873	200,154	49,9	54,3	0,85	777	
	Total	22179,556	368,608	100,0	100,0			

Column	: Chiralpak AD-H	Detection	:
Mobile Phase	: hexan/IPA 70/30	Temperature	:
Flow Rate	: 0,5 ml/min	Pressure	: 31 bar
Note	:		

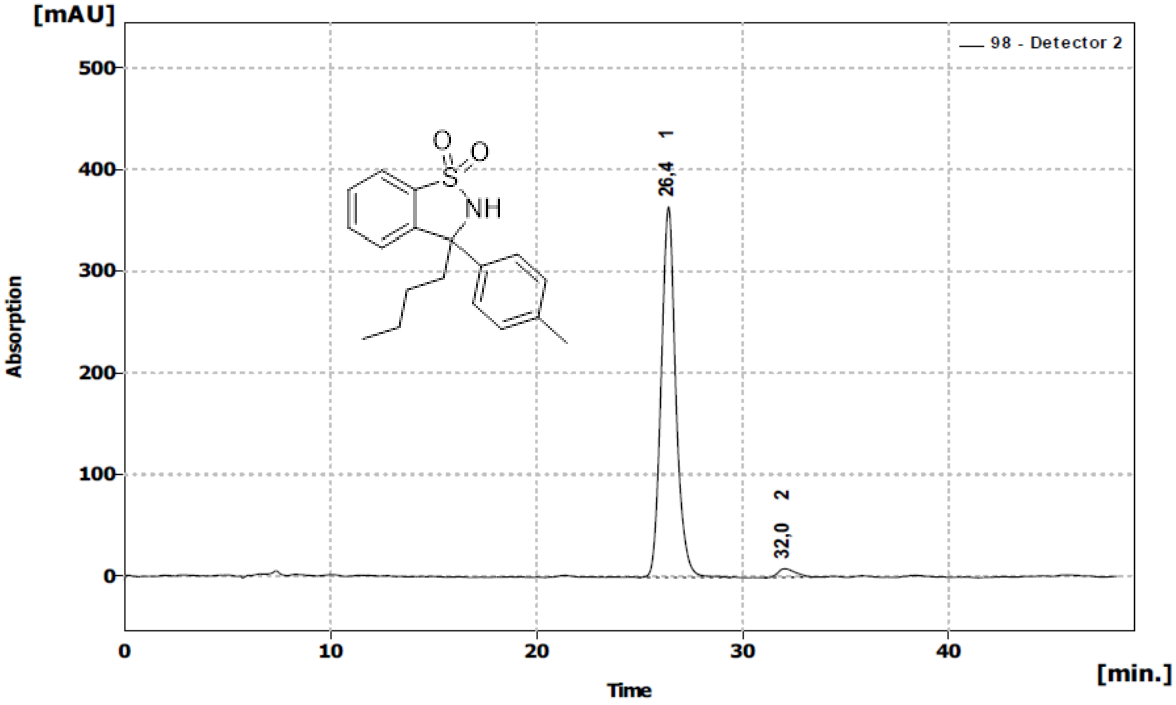

Result Table (Uncal - 98 - Detector 2)

	Reten. Time [min]	Area [mAU.s]	Height [mAU]	Area [%]	Height [%]	W05 [min]	Peak Purity [-]	Compound Name
1	26,408	18090,467	364,718	97,2	97,7	0,74	848	
2	32,025	520,109	8,654	2,8	2,3	0,97	986	
	Total	18610,577	373,372	100,0	100,0			

Column	: Chiralpak AD-H	Detection	:
Mobile Phase	: hexan/IPA 70/30	Temperature	:
Flow Rate	: 0,5 ml/min	Pressure	: 31 bar
Note	:		

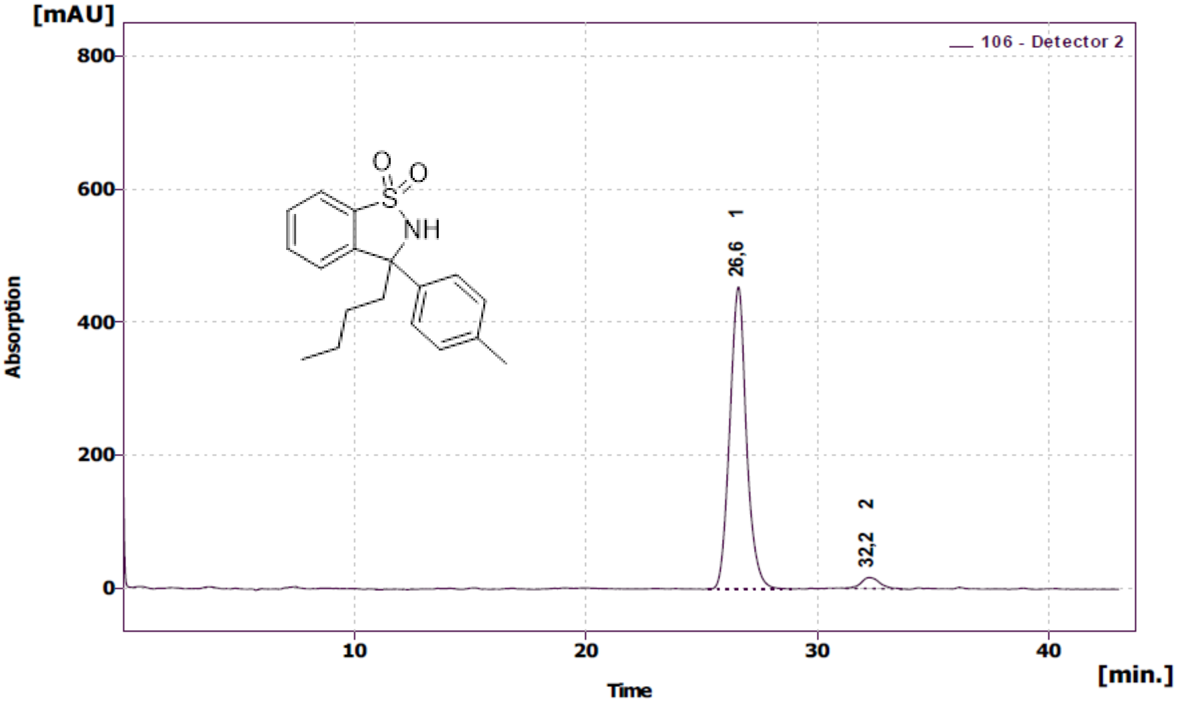

Result Table (Uncal - 106 - Detector 2)

	Reten. Time [min]	Area [mAU.s]	Height [mAU]	Area [%]	Height [%]	W05 [min]	Peak Purity [-]	Compound Name
1	26,592	22872,686	454,694	96,0	96,5	0,76	837	
2	32,217	942,412	16,658	4,0	3,5	0,84	968	
	Total	23815,098	471,352	100,0	100,0			

Column	: Chiralpak AD-H		Detection	:
Mobile Phase	: hexan/IPA 70/30		Temperature	:
Flow Rate	: 1,0 ml/min		Pressure	: 61 bar
Note	:			

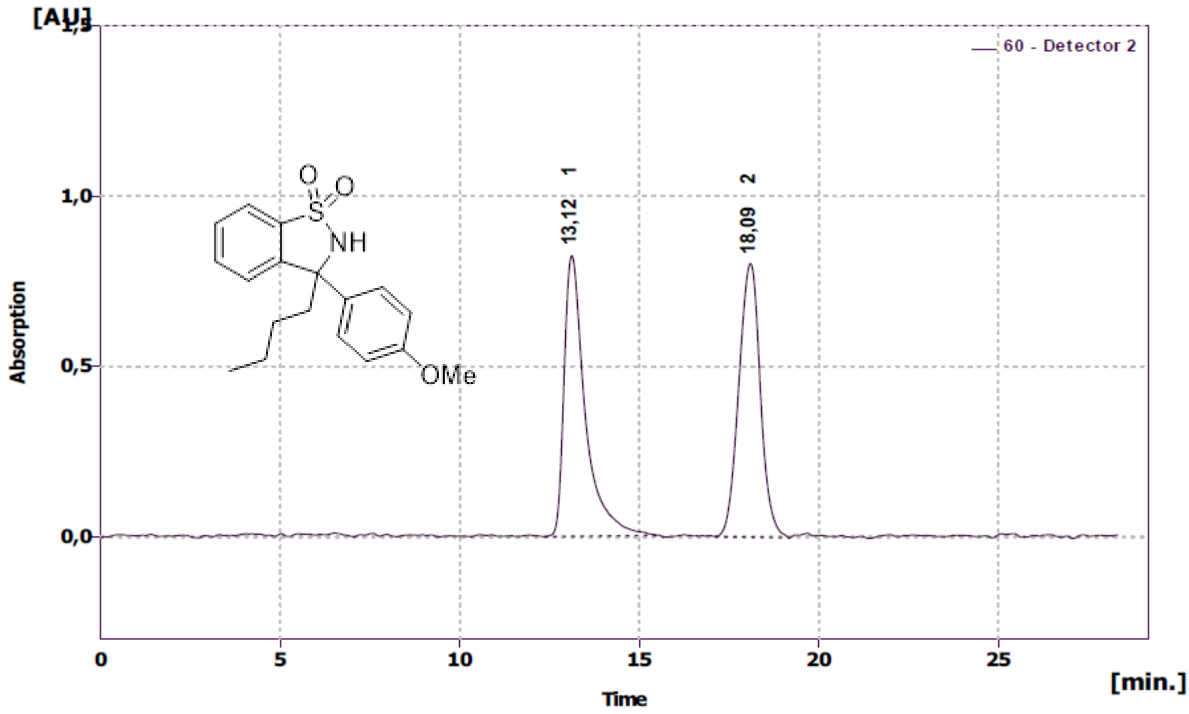

Result Table (Uncal - 60 - Detector 2)

	Reten. Time [min]	Area [mAU.s]	Height [mAU]	Area [%]	Height [%]	W05 [min]	Peak Purity [-]	Compound Name
1	13,117	33742,232	823,759	50,2	50,7	0,57	878	
2	18,092	33525,320	802,610	49,8	49,3	0,65	894	
	Total	67267,552	1626,369	100,0	100,0			

Column	: Chiralpak AD-H		Detection	:
Mobile Phase	: hexan/IPA 70/30		Temperature	:
Flow Rate	: 1,0 ml/min		Pressure	: 61 bar
Note	:			

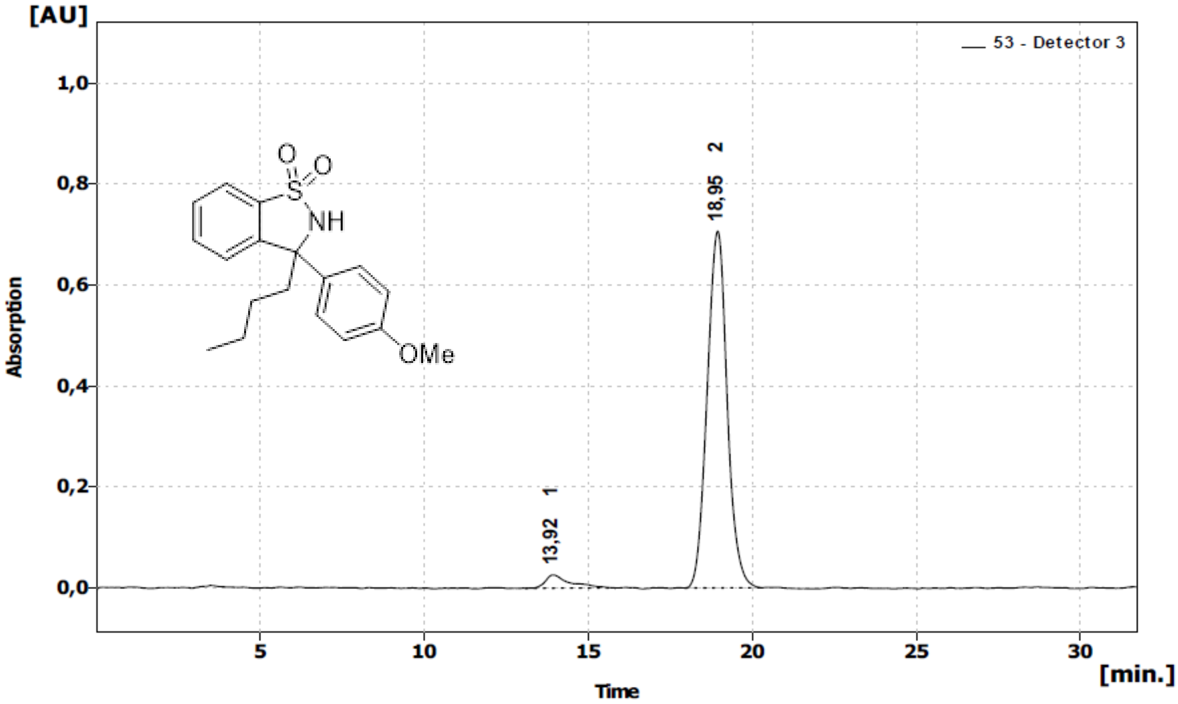

Result Table (Uncal - 53 - Detector 3)

	Reten. Time [min]	Area [mAU.s]	Height [mAU]	Area [%]	Height [%]	W05 [min]	Peak Purity [-]	Compound Name
1	13,917	1406,701	26,389	4,5	3,6	0,65	890	
2	18,950	30008,963	707,176	95,5	96,4	0,66	854	
	Total	31415,663	733,565	100,0	100,0			

Column : Chiralpak AD-H
Mobile Phase : hexan/IPA 70/30
Flow Rate : 1,0 ml/min
Note :

Detection :
Temperature :
Pressure : 61 bar

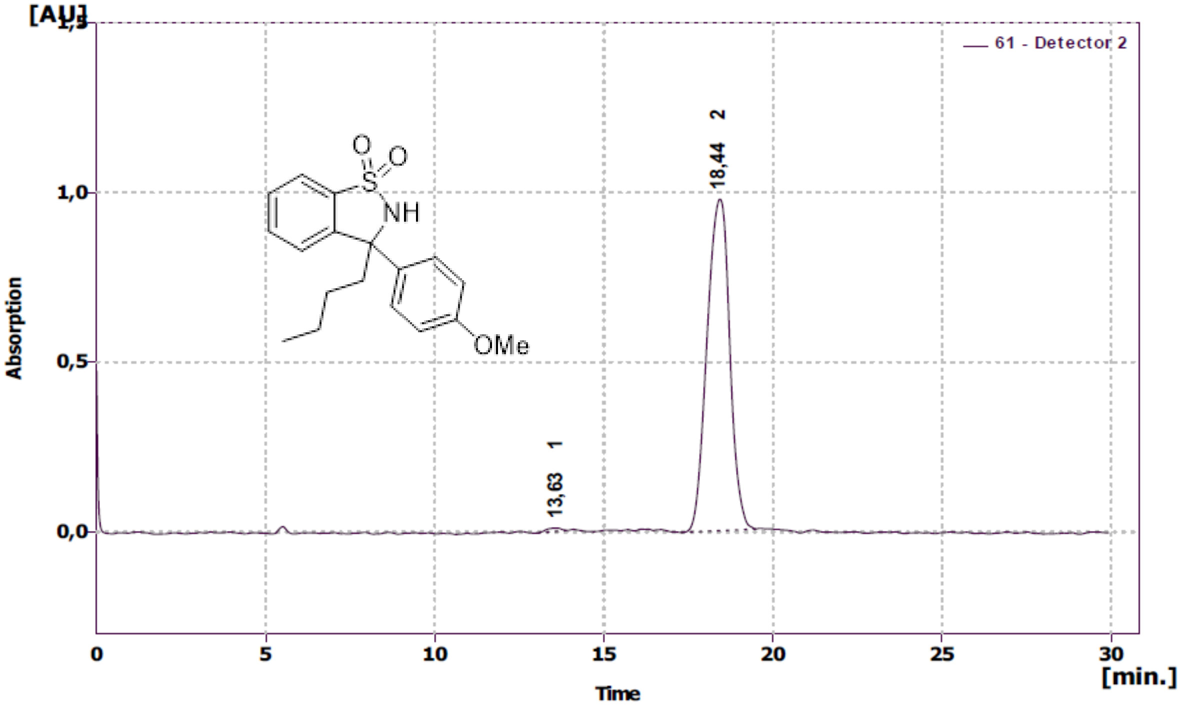

Result Table (Uncal - 61 - Detector 2)

	Reten. Time [min]	Area [mAU.s]	Height [mAU]	Area [%]	Height [%]	W05 [min]	Peak Purity [-]	Compound Name
1	13,633	327,917	9,467	0,7	1,0	0,51	791	
2	18,442	45604,352	976,372	99,3	99,0	0,75	867	
	Total	45932,269	985,839	100,0	100,0			

Column	: Chiralpak AD-H	Detection	:
Mobile Phase	: hexan/IPA 70/30	Temperature	:
Flow Rate	: 1,0 ml/min	Pressure	: 61 bar
Note	:		

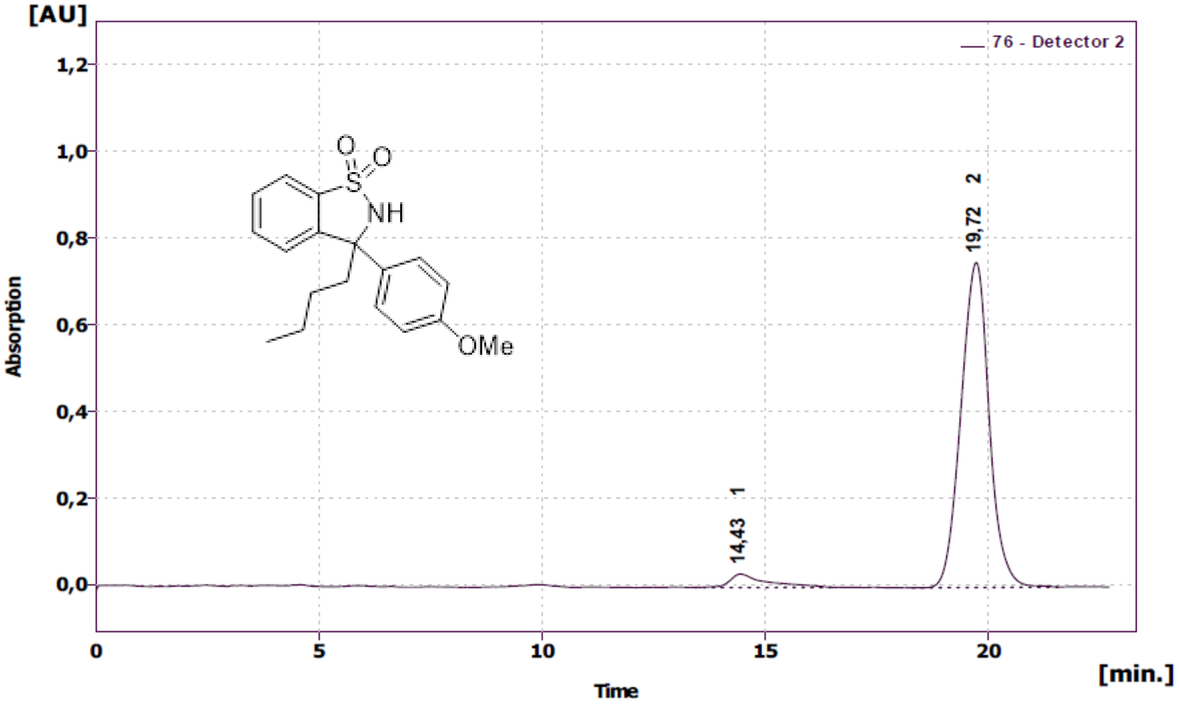

Result Table (Uncal - 76 - Detector 2)

	Reten. Time [min]	Area [mAU.s]	Height [mAU]	Area [%]	Height [%]	W05 [min]	Peak Purity [-]	Compound Name
1	14,425	1812,961	31,050	5,2	4,0	0,68	764	
2	19,717	32973,821	749,222	94,8	96,0	0,68	707	
	Total	34786,782	780,272	100,0	100,0			

Column	: Chiralpak IA	Detection	:
Mobile Phase	: hexan/IPA 70/30	Temperature	:
Flow Rate	: 1,0 ml/min	Pressure	: 54 bar
Note	:		

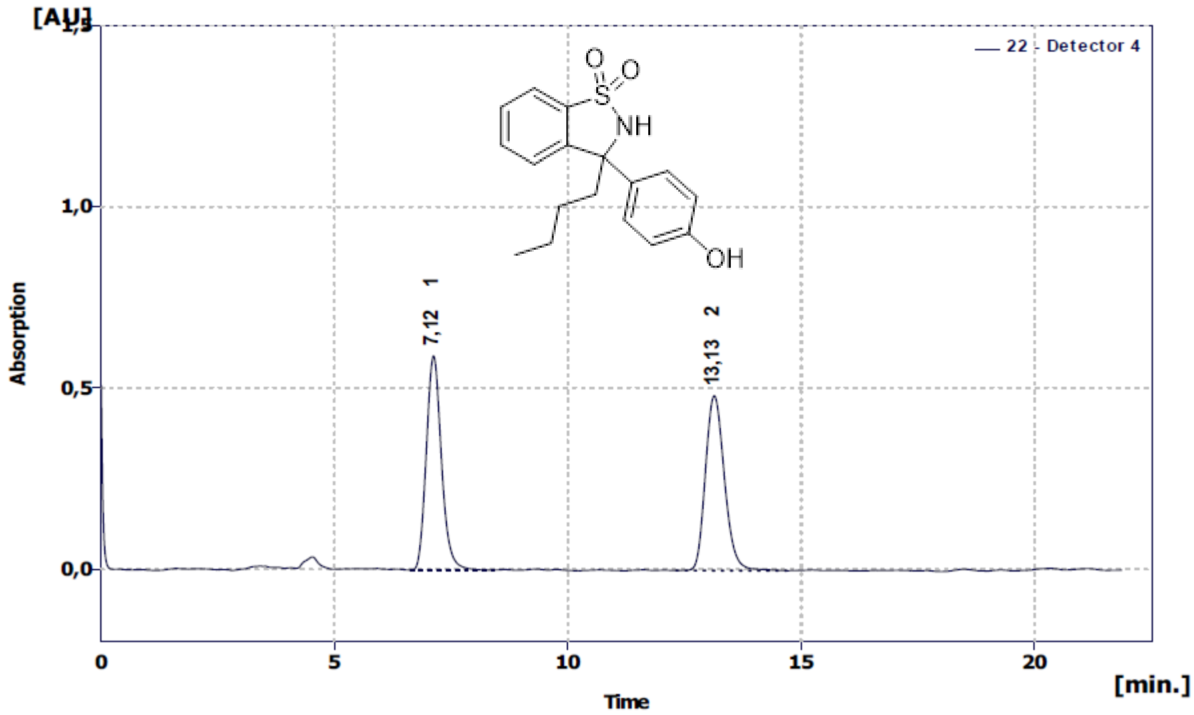

Result Table (Uncal - 22 - Detector 4)

	Reten. Time [min]	Area [mAU.s]	Height [mAU]	Area [%]	Height [%]	W05 [min]	Peak Purity [-]	Compound Name
1	7,117	13363,945	590,925	49,4	55,1	0,35	685	
2	13,133	13693,387	482,421	50,6	44,9	0,44	619	
	Total	27057,332	1073,346	100,0	100,0			

Column	: Chiralpak IA		Detection	:	
Mobile Phase	: hexan/IPA 70/30		Temperature	:	
Flow Rate	: 1,0 ml/min		Pressure	: 54 bar	
Note	:				

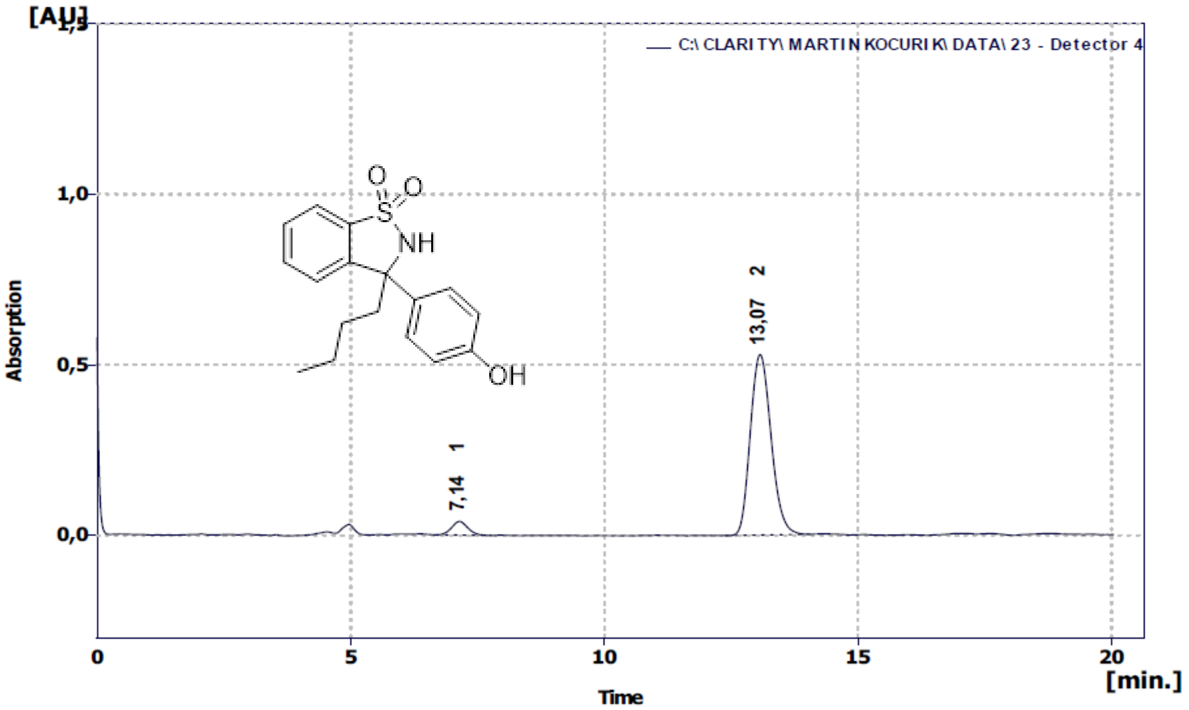

Result Table (Uncal - C:\CLARITY\MARTIN KOCURIK\DATA\23 - Detector 4)

	Reten. Time [min]	Area [mAU.s]	Height [mAU]	Area [%]	Height [%]	W05 [min]	Peak Purity [-]	Compound Name
1	7,142	896,690	40,264	5,6	7,1	0,34	935	
2	13,067	15062,658	529,432	94,4	92,9	0,46	819	
	Total	15959,348	569,696	100,0	100,0			

Column : Chiralpak IA
Mobile Phase : hexan/IPA 70/30
Flow Rate : 1,0 ml/min
Note :

Detection :
Temperature :
Pressure : 54 bar

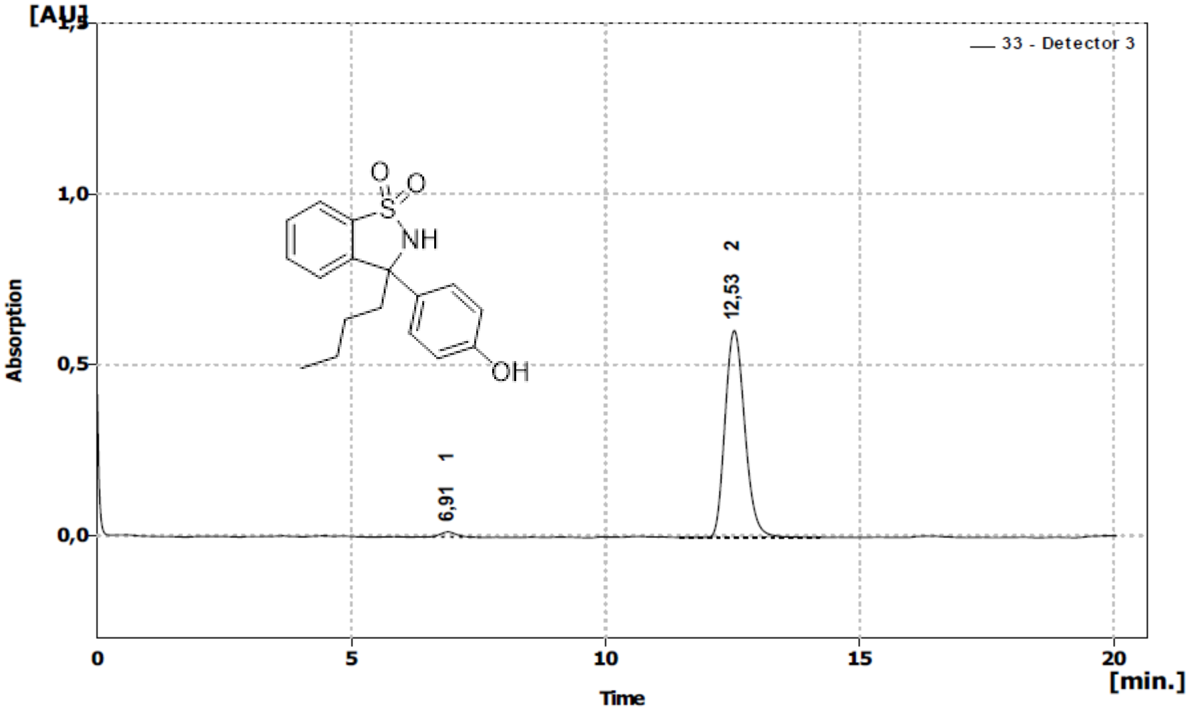

Result Table (Uncal - 33 - Detector 3)

	Reten. Time [min]	Area [mAU.s]	Height [mAU]	Area [%]	Height [%]	W05 [min]	Peak Purity [-]	Compound Name
1	6,908	354,392	15,567	2,1	2,5	0,35	580	
2	12,533	16400,300	605,893	97,9	97,5	0,42	654	
	Total	16754,692	621,461	100,0	100,0			

Column	: Chiralpak IA	Detection	:
Mobile Phase	: hexan/IPA 70/30	Temperature	:
Flow Rate	: 1,0 ml/min	Pressure	: 54 bar
Note	:		

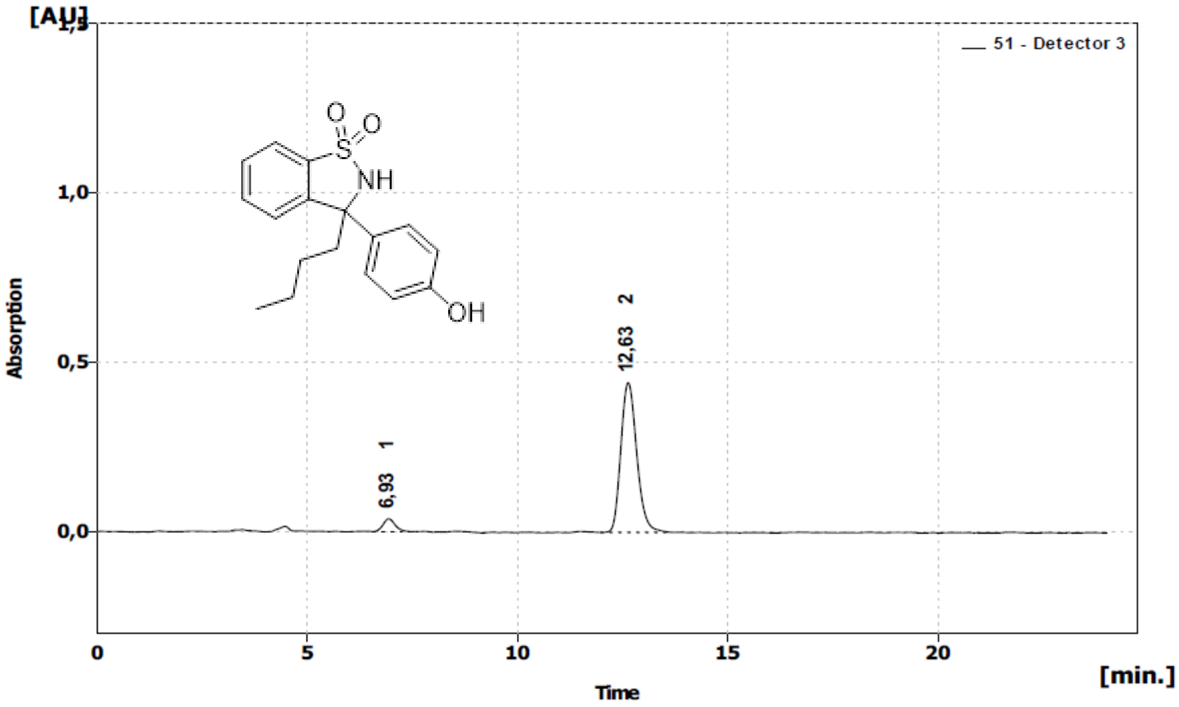

Result Table (Uncal - 51 - Detector 3)

	Reten. Time [min]	Area [mAU.s]	Height [mAU]	Area [%]	Height [%]	W05 [min]	Peak Purity [-]	Compound Name
1	6,933	750,880	37,746	6,0	7,9	0,32	977	
2	12,633	11673,208	442,785	94,0	92,1	0,41	779	
	Total	12424,088	480,531	100,0	100,0			

Column	: Chiralpak AD-H		Detection	:	
Mobile Phase	: hexan/IPA 70/30		Temperature	:	
Flow Rate	: 0,5 ml/min		Pressure	: 30 bar	
Note	:				

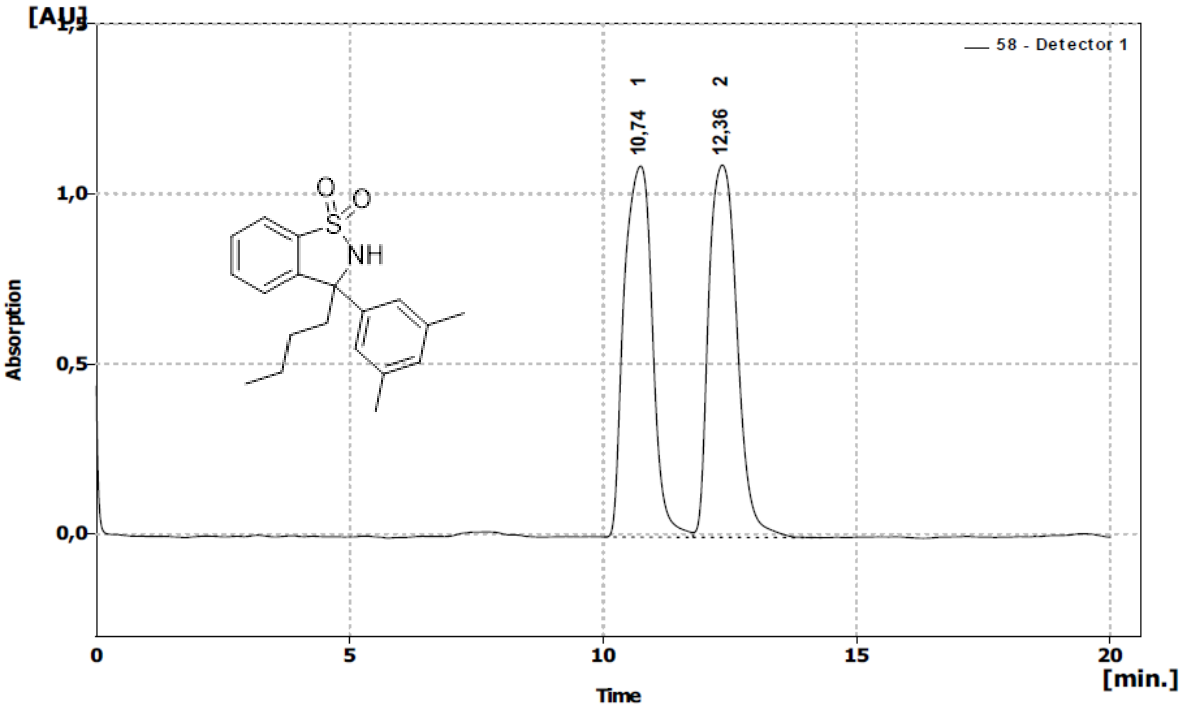

Result Table (Uncal - 58 - Detector 1)

	Reten. Time [min]	Area [mAU.s]	Height [mAU]	Area [%]	Height [%]	W05 [min]	Peak Purity [-]	Compound Name
1	10,742	42328,324	1091,261	49,8	49,9	0,64	797	
2	12,358	42731,826	1094,689	50,2	50,1	0,63	636	
	Total	85060,151	2185,951	100,0	100,0			

Column	: Chiralpak AD-H		Detection	:	
Mobile Phase	: hexan/IPA 70/30		Temperature	:	
Flow Rate	: 0,5 ml/min		Pressure	: 30 bar	
Note	:				

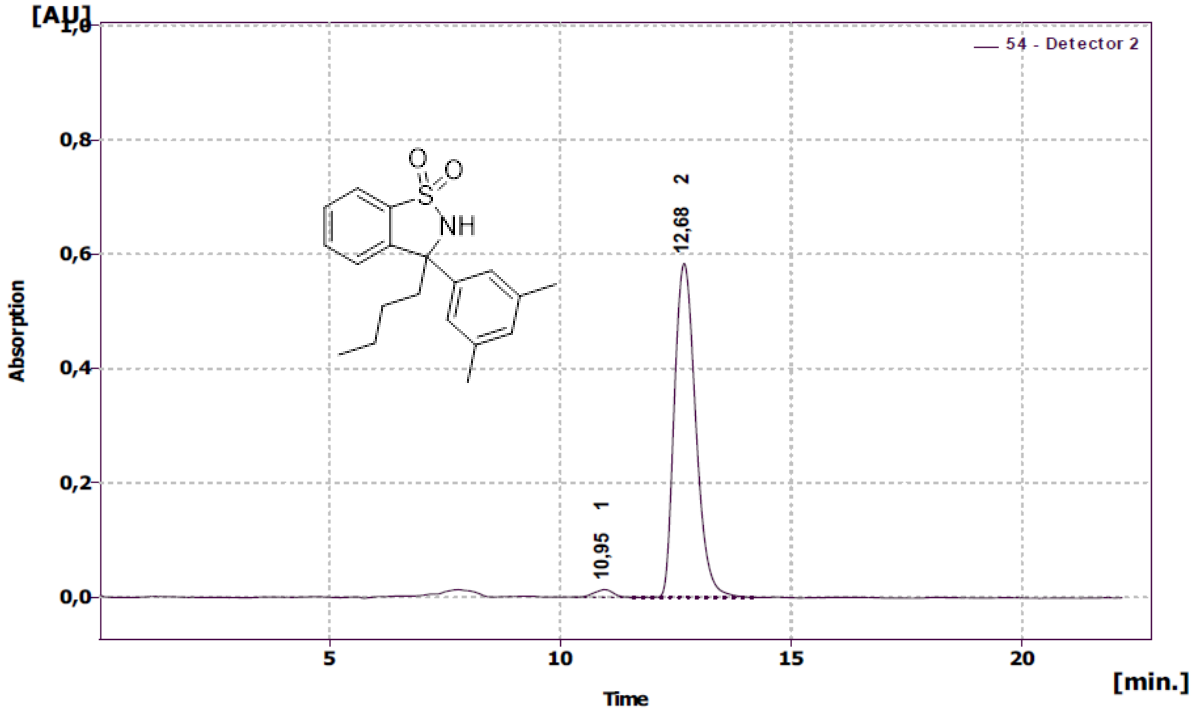

Result Table (Uncal - 54 - Detector 2)

	Reten. Time [min]	Area [mAU.s]	Height [mAU]	Area [%]	Height [%]	W05 [min]	Peak Purity [-]	Compound Name
1	10,950	390,326	13,485	2,0	2,3	0,47	961	
2	12,683	18808,269	584,277	98,0	97,7	0,51	717	
	Total	19198,595	597,762	100,0	100,0			

Column	: Chiralpak AD-H	Detection	:
Mobile Phase	: hexan/IPA 70/30	Temperature	:
Flow Rate	: 0,5 ml/min	Pressure	: 30 bar
Note	:		

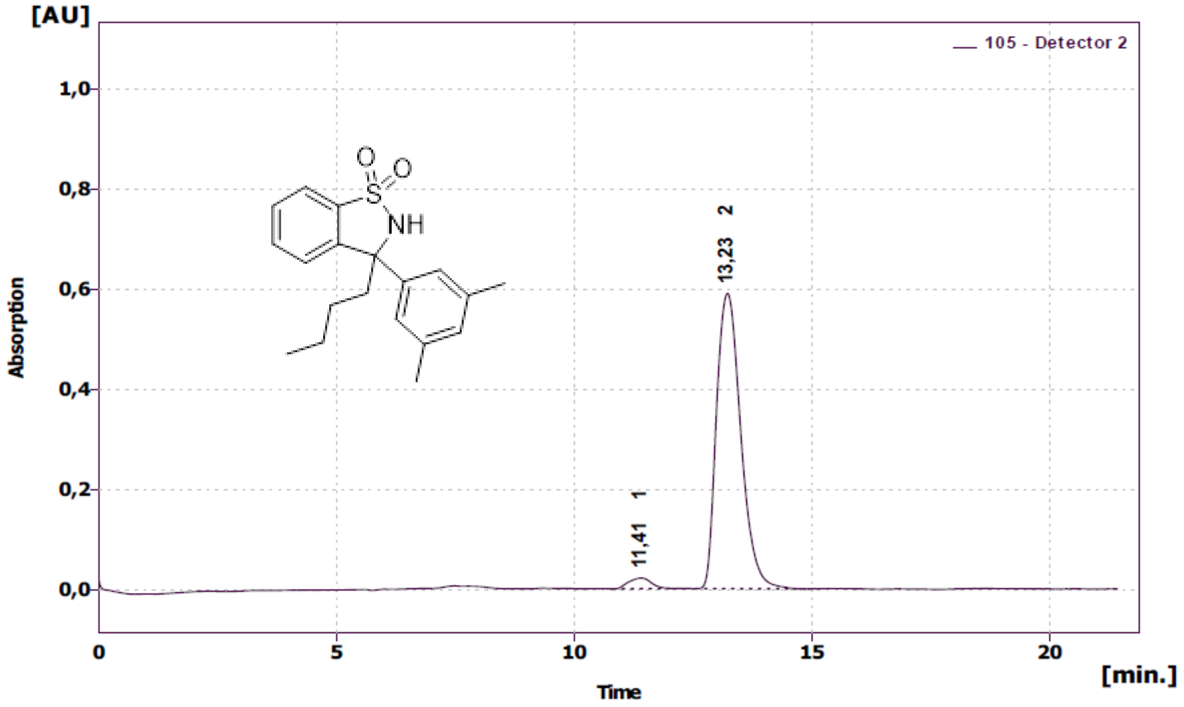

Result Table (Uncal - 105 - Detector 2)

	Reten. Time [min]	Area [mAU.s]	Height [mAU]	Area [%]	Height [%]	W05 [min]	Peak Purity [-]	Compound Name
1	11,408	706,839	21,381	3,3	3,5	0,56	989	
2	13,225	20896,451	588,399	96,7	96,5	0,56	842	
	Total	21603,290	609,780	100,0	100,0			

Column	: Chiralpak IA		Detection	:
Mobile Phase	: hexan/IPA 70/30		Temperature	:
Flow Rate	: 1,0 ml/min		Pressure	: 54 bar
Note	:			

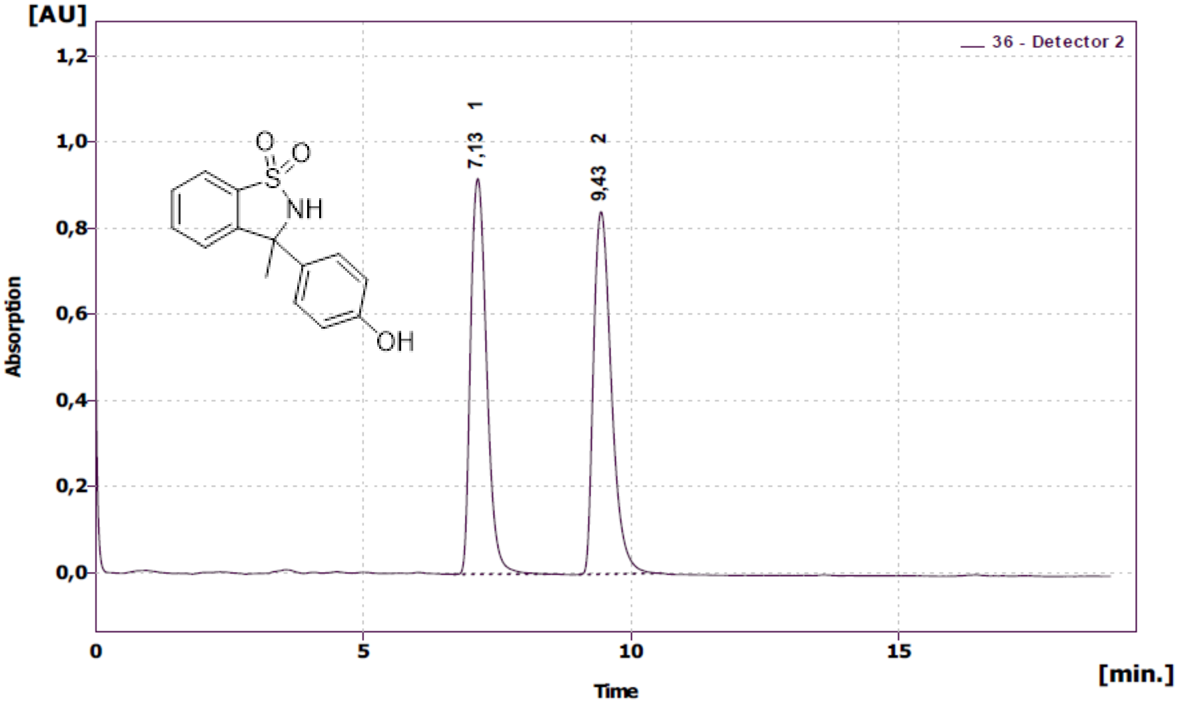

Result Table (Uncal - 36 - Detector 2)

	Reten. Time [min]	Area [mAU.s]	Height [mAU]	Area [%]	Height [%]	W05 [min]	Peak Purity [-]	Compound Name
1	7,133	19156,740	918,652	49,4	52,2	0,33	691	
2	9,433	19589,790	841,616	50,6	47,8	0,37	773	
	Total	38746,531	1760,269	100,0	100,0			

Column	: Chiralpak IA	Detection	:
Mobile Phase	: hexan/IPA 70/30	Temperature	:
Flow Rate	: 1,0 ml/min	Pressure	: 54 bar
Note	:		

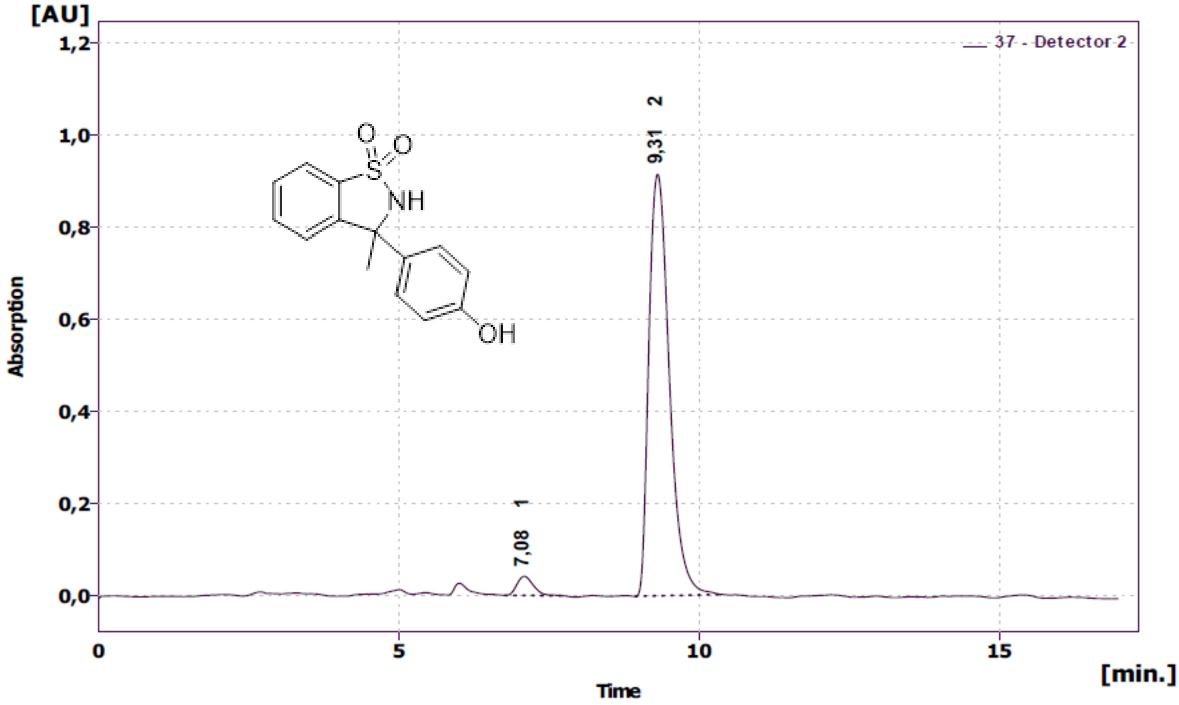

Result Table (Uncal - 37 - Detector 2)

	Reten. Time [min]	Area [mAU.s]	Height [mAU]	Area [%]	Height [%]	W05 [min]	Peak Purity [-]	Compound Name
1	7,083	793,789	41,581	3,5	4,3	0,30	920	
2	9,308	21661,958	915,456	96,5	95,7	0,38	892	
	Total	22455,747	957,037	100,0	100,0			

Column	: Chiralpak AD-H		Detection	:
Mobile Phase	: hexan/IPA 70/30		Temperature	:
Flow Rate	: 0,8 ml/min		Pressure	: 43 bar
Note	:			

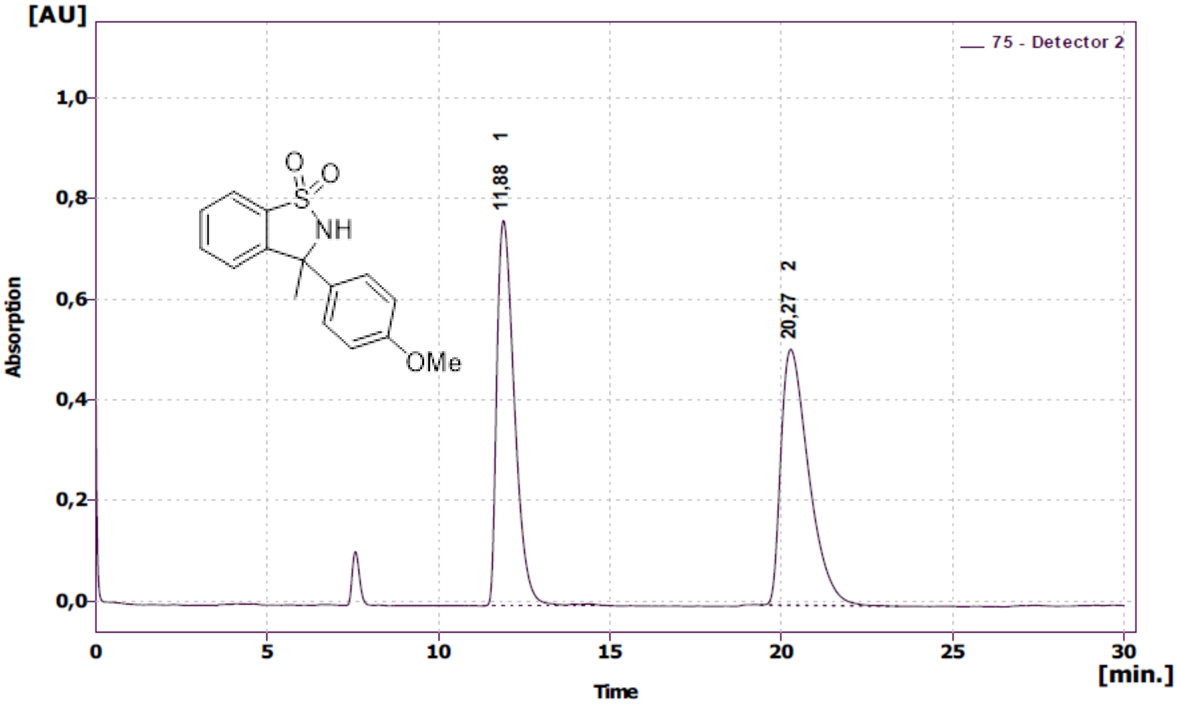

Result Table (Uncal - 75 - Detector 2)

	Reten. Time [min]	Area [mAU.s]	Height [mAU]	Area [%]	Height [%]	W05 [min]	Peak Purity [-]	Compound Name
1	11,883	27489,796	765,306	48,9	60,1	0,57	881	
2	20,275	28760,169	508,655	51,1	39,9	0,88	834	
	Total	56249,965	1273,960	100,0	100,0			

Column	: Chiralpak AD-H	Detection	:
Mobile Phase	: hexan/IPA 70/30	Temperature	:
Flow Rate	: 0,8 ml/min	Pressure	: 43 bar
Note	:		

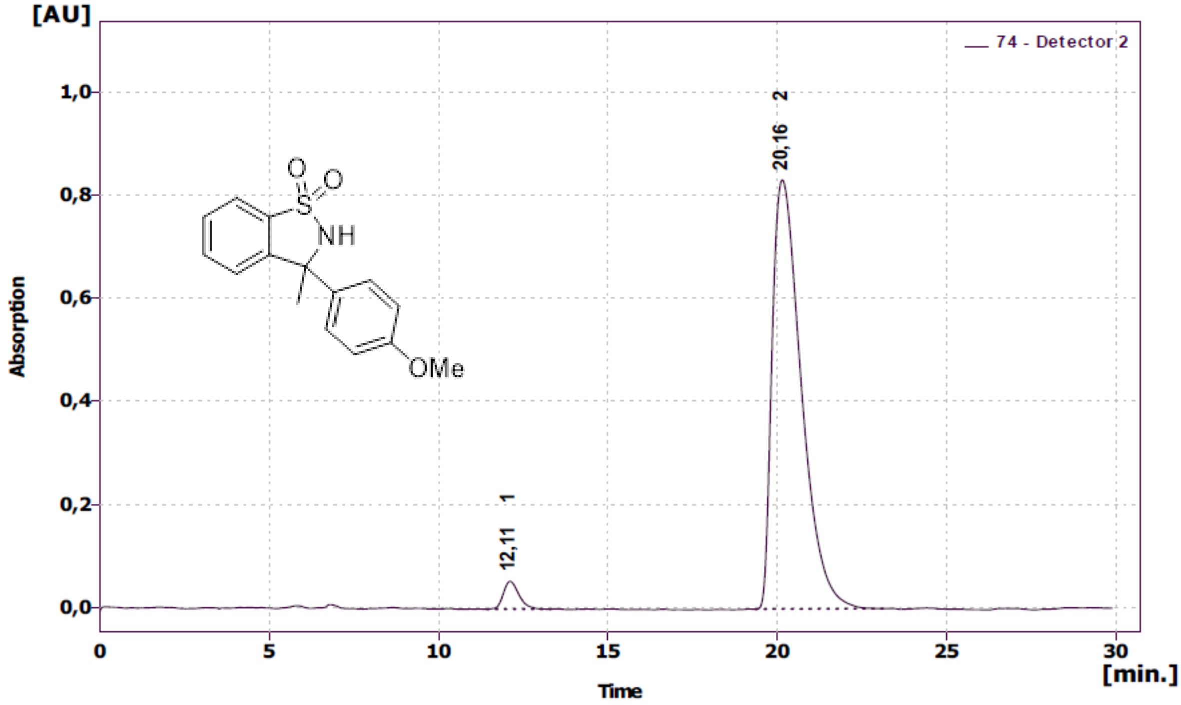

Result Table (Uncal - 74 - Detector 2)

	Reten. Time [min]	Area [mAU.s]	Height [mAU]	Area [%]	Height [%]	W05 [min]	Peak Purity [-]	Compound Name
1	12,108	1749,077	54,135	3,4	6,1	0,49	824	
2	20,158	49808,488	831,792	96,6	93,9	0,93	852	
	Total	51557,565	885,927	100,0	100,0			

Column	: Chiralcel OD-H	Detection	:
Mobile Phase	: hexan/IPA 70/30	Temperature	:
Flow Rate	: 0,8 ml/min	Pressure	: 43 bar
Note	:		

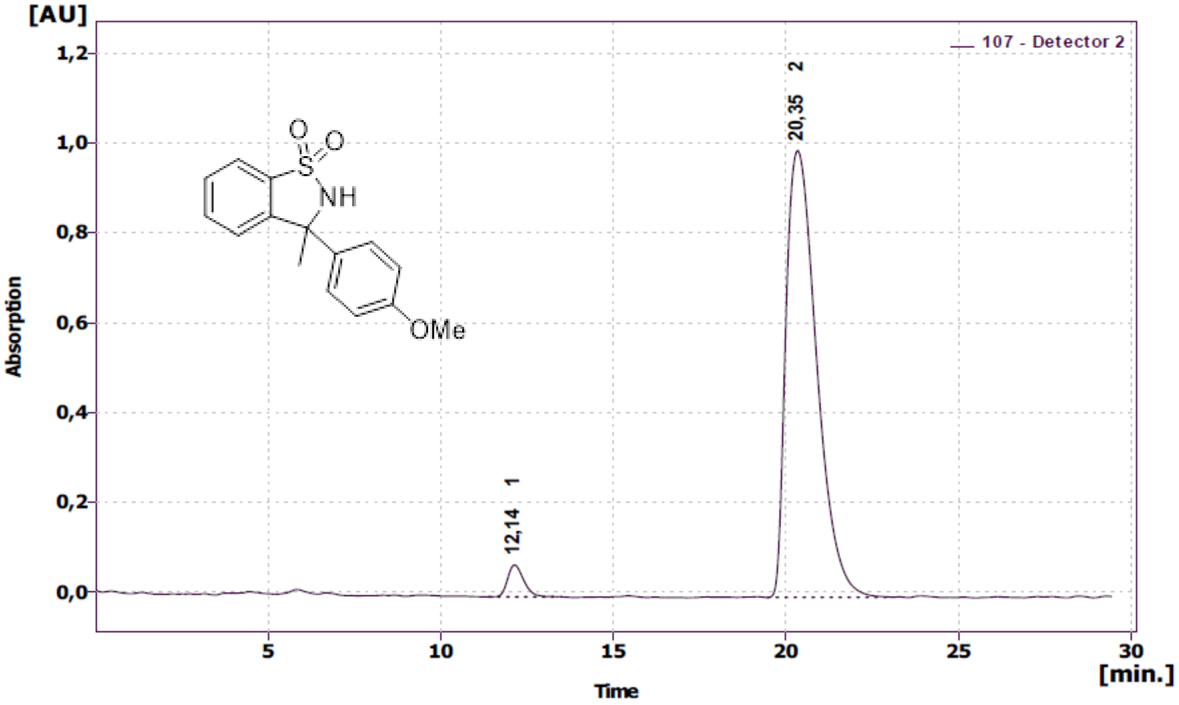

Result Table (Uncal - 107 - Detector 2)

	Reten. Time [min]	Area [mAU.s]	Height [mAU]	Area [%]	Height [%]	W05 [min]	Peak Purity [-]	Compound Name
1	12,142	2310,776	71,250	3,6	6,7	0,50	713	
2	20,350	61431,280	994,793	96,4	93,3	0,97	752	
	Total	63742,055	1066,042	100,0	100,0			

Column	: Chiralcel OD-H		Detection	:
Mobile Phase	: hexan/IPA 70/30		Temperature	:
Flow Rate	: 0,8 ml/min		Pressure	: 43 bar
Note	:			

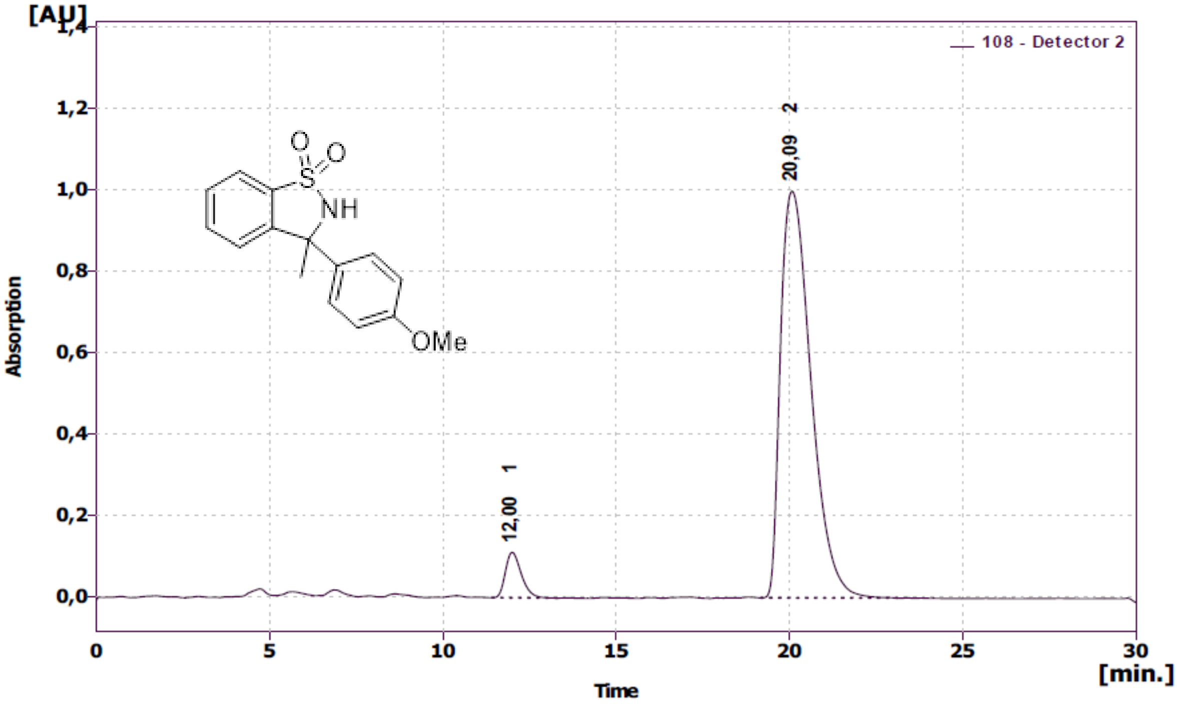

Result Table (Uncal - 108 - Detector 2)

	Reten. Time [min]	Area [mAU.s]	Height [mAU]	Area [%]	Height [%]	W05 [min]	Peak Purity [-]	Compound Name
1	12,000	3582,123	111,777	5,5	10,1	0,50	908	
2	20,092	61031,623	997,928	94,5	89,9	0,96	830	
	Total	64613,746	1109,705	100,0	100,0			

Column	: Chiralpak IA	Detection	:
Mobile Phase	: hexan/IPA 70/30	Temperature	:
Flow Rate	: 1,0 ml/min	Pressure	: 54 bar
Note	:		

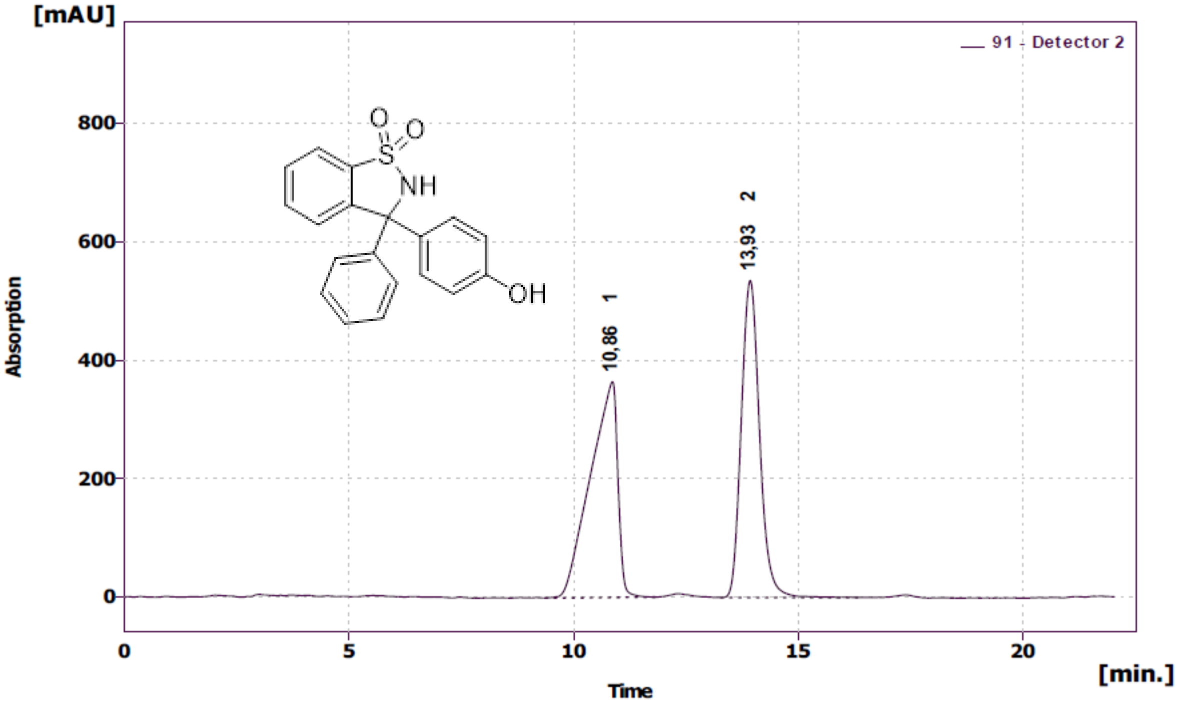

Result Table (Uncal - 91 - Detector 2)

	Reten. Time [min]	Area [mAU.s]	Height [mAU]	Area [%]	Height [%]	W05 [min]	Peak Purity [-]	Compound Name
1	10,858	15498,027	364,253	50,5	40,5	0,70	883	
2	13,933	15178,120	535,411	49,5	59,5	0,44	829	
	Total	30676,147	899,664	100,0	100,0			

Column	: Chiralpak IA	Detection	:
Mobile Phase	: hexan/IPA 70/30	Temperature	:
Flow Rate	: 1,0 ml/min	Pressure	: 54 bar
Note	:		

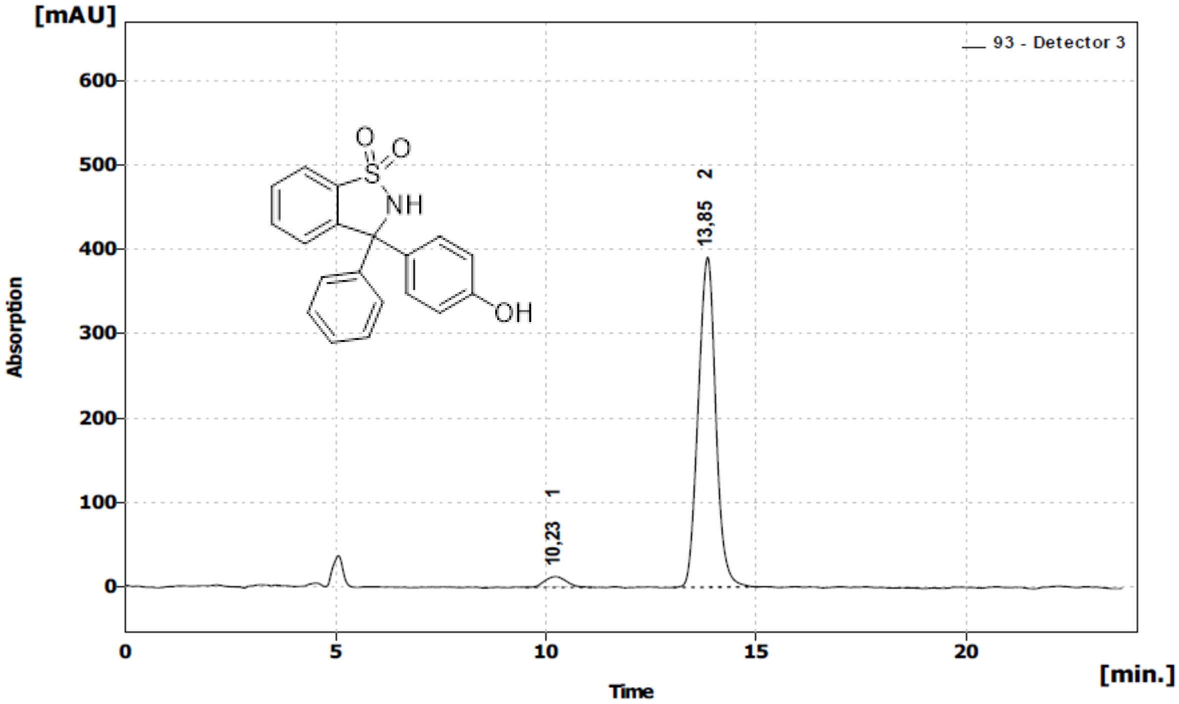

Result Table (Uncal - 93 - Detector 3)

	Reten. Time [min]	Area [mAU.s]	Height [mAU]	Area [%]	Height [%]	W05 [min]	Peak Purity [-]	Compound Name
1	10,225	470,601	12,876	4,0	3,2	0,59	883	
2	13,850	11382,988	391,642	96,0	96,8	0,45	806	
	Total	11853,589	404,518	100,0	100,0			

Column	: Chiralcel OD-H	Detection	:
Mobile Phase	: hexan/IPA 80/20	Temperature	:
Flow Rate	: 0,6 ml/min	Pressure	: 29 bar
Note	:		

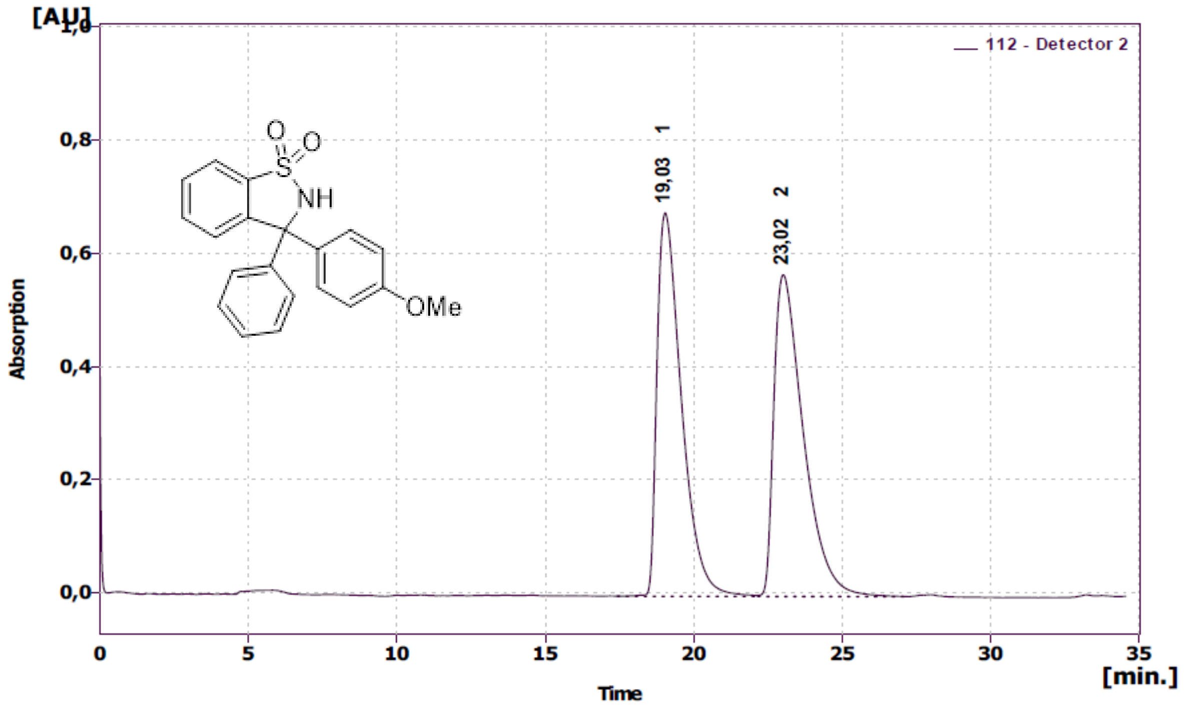

Result Table (Uncal - 112 - Detector 2)

	Reten. Time [min]	Area [mAU.s]	Height [mAU]	Area [%]	Height [%]	W05 [min]	Peak Purity [-]	Compound Name
1	19,033	38834,409	677,640	49,7	54,4	0,88	765	
2	23,025	39302,511	568,434	50,3	45,6	1,05	758	
	Total	78136,920	1246,074	100,0	100,0			

Column	: Chiralcel OD-H		Detection	:	
Mobile Phase	: hexan/IPA 80/20		Temperature	:	
Flow Rate	: 0,6 ml/min		Pressure	: 29 bar	
Note	:				

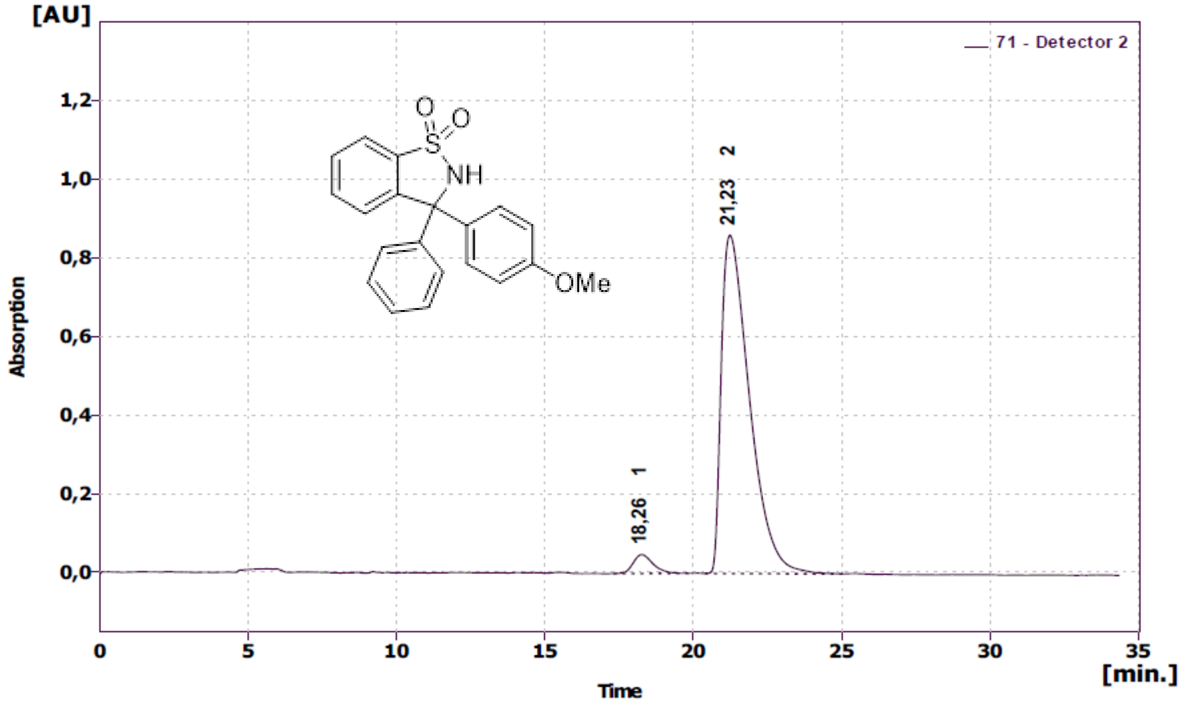

Result Table (Uncal - 71 - Detector 2)

	Reten. Time [min]	Area [mAU.s]	Height [mAU]	Area [%]	Height [%]	W05 [min]	Peak Purity [-]	Compound Name
1	18,258	2228,136	48,206	3,8	5,3	0,71	908	
2	21,233	56822,909	861,431	96,2	94,7	1,02	776	
	Total	59051,046	909,637	100,0	100,0			

Column	: Chiralpak IA	Detection	:
Mobile Phase	: hexan/IPA 70/30	Temperature	:
Flow Rate	: 1,0 ml/min	Pressure	: 54 bar
Note	:		

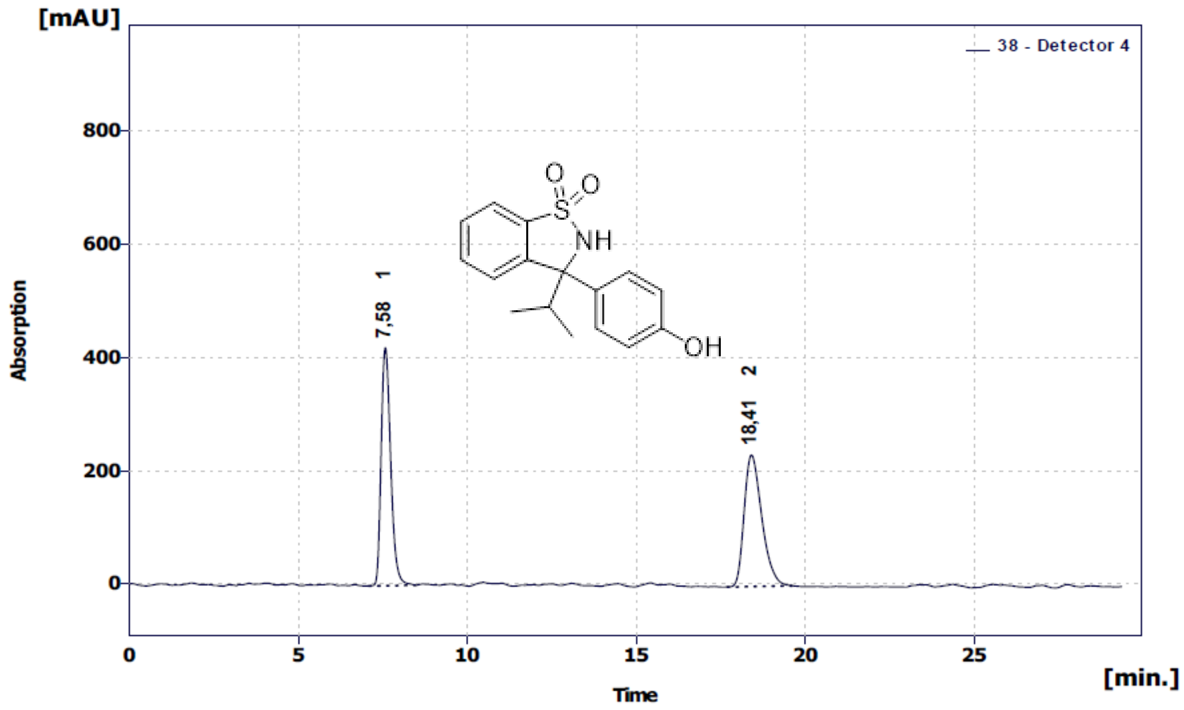

Result Table (Uncal - 38 - Detector 4)

	Reten. Time [min]	Area [mAU.s]	Height [mAU]	Area [%]	Height [%]	W05 [min]	Peak Purity [-]	Compound Name
1	7,575	8231,725	420,304	49,4	64,4	0,31	749	
2	18,408	8441,758	232,296	50,6	35,6	0,57	793	
	Total	16673,483	652,600	100,0	100,0			

Column	: Chiralpak IA	Detection	:
Mobile Phase	: hexan/IPA 70/30	Temperature	:
Flow Rate	: 1,0 ml/min	Pressure	: 54 bar
Note	:		

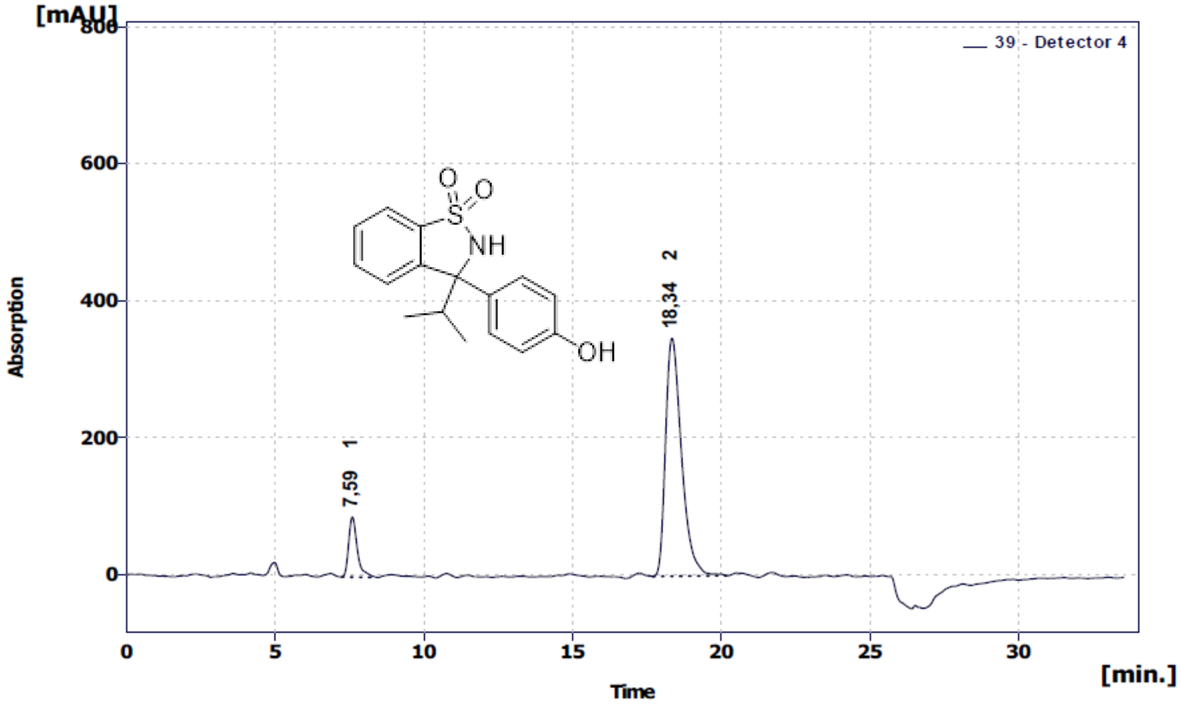

Result Table (Uncal - 39 - Detector 4)

	Reten. Time [min]	Area [mAU.s]	Height [mAU]	Area [%]	Height [%]	W05 [min]	Peak Purity [-]	Compound Name
1	7,592	1785,607	87,860	12,1	20,2	0,30	780	
2	18,342	13023,760	347,486	87,9	79,8	0,57	854	
	Total	14809,367	435,346	100,0	100,0			

Column	: Chiralpak AD-H	Detection	:
Mobile Phase	: hexan/IPA 90/10	Temperature	:
Flow Rate	: 1,0 ml/min	Pressure	: 41 bar
Note	:		

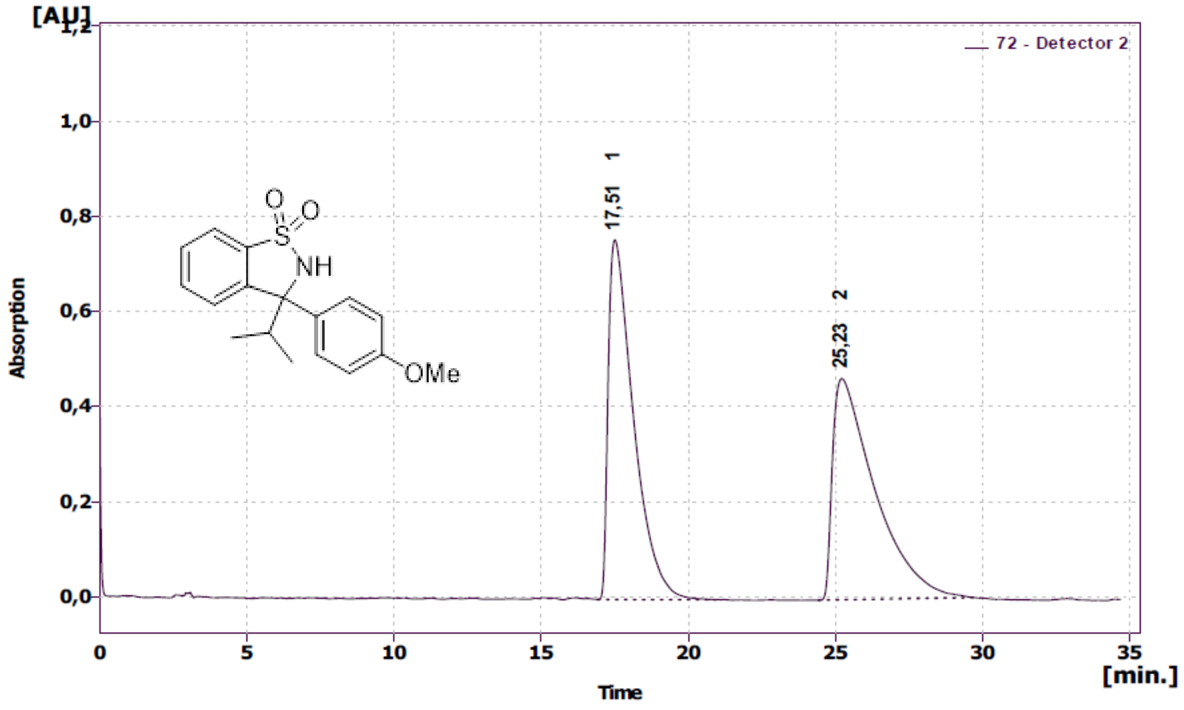

Result Table (Uncal - 72 - Detector 2)

	Reten. Time [min]	Area [mAU.s]	Height [mAU]	Area [%]	Height [%]	W05 [min]	Peak Purity [-]	Compound Name
1	17,508	45200,547	756,526	49,2	61,9	0,91	749	
2	25,233	46588,538	465,465	50,8	38,1	1,49	913	
	Total	91789,085	1221,991	100,0	100,0			

Column	: Chiralpak AD-H	Detection	:
Mobile Phase	: hexan/IPA 90/10	Temperature	:
Flow Rate	: 1,0 ml/min	Pressure	: 41 bar
Note	:		

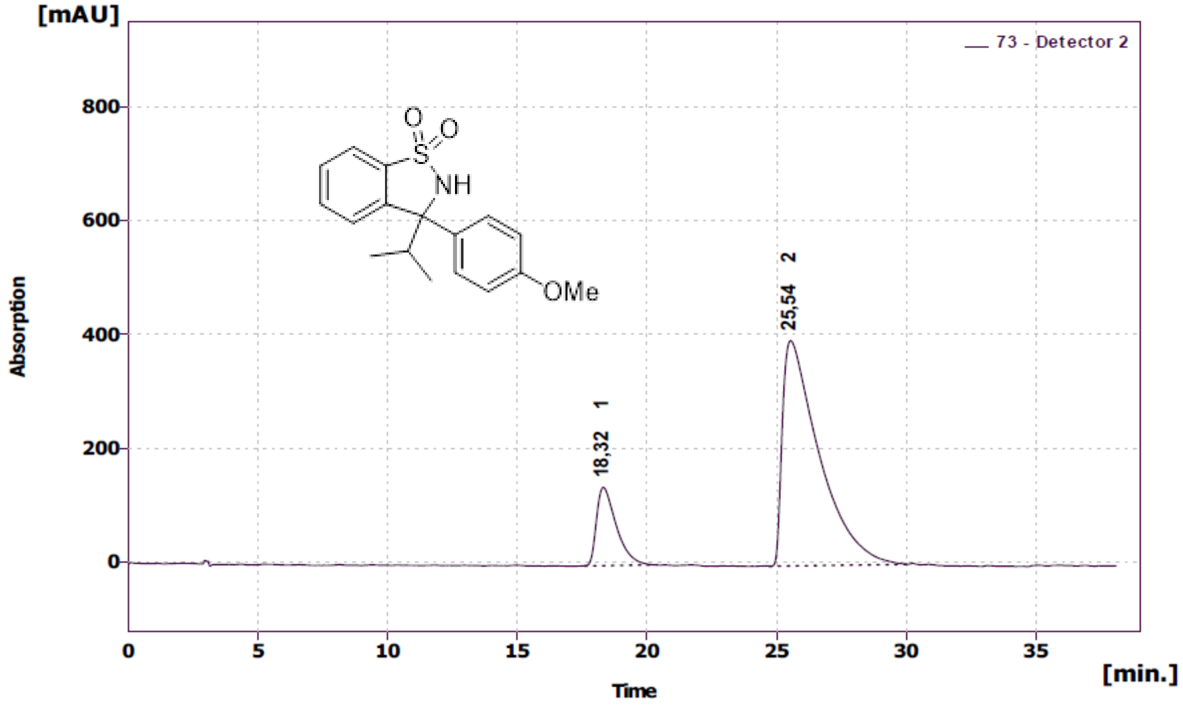

Result Table (Uncal - 73 - Detector 2)

	Reten. Time [min]	Area [mAU.s]	Height [mAU]	Area [%]	Height [%]	W05 [min]	Peak Purity [-]	Compound Name
1	18,317	7307,457	137,449	15,8	25,8	0,82	812	
2	25,542	39035,784	395,848	84,2	74,2	1,47	905	
	Total	46343,241	533,297	100,0	100,0			

References

(1) Bouillon, I.; Soural, M.; Miller, M. J.; Krchňák, V. Resins with Identical Specifications Are Not Identical. Identifying a Useful Solid-Phase Resin. *J. Comb. Chem.* **2009**, *11* (2), 213–215. https://doi.org/10.1021/cc800143e.

(2) Varrà, M. O.; Husáková, L.; Patočka, J.; Ghidini, S.; Zanardi, E. Classification of Transformed Anchovy Products Based on the Use of Element Patterns and Decision Trees to Assess Traceability and Country of Origin Labelling. *Food Chem.* **2021**, *360*, 129790. https://doi.org/10.1016/j.foodchem.2021.129790.

(3) Pezzetta, C.; Bonifazi, D.; Davidson, R. W. M. Enantioselective Synthesis of N-Benzylic Heterocycles: A Nickel and Photoredox Dual Catalysis Approach. *Org. Lett.* **2019**, *21* (22), 8957–8961. https://doi.org/10.1021/acs.orglett.9b03338.

(4) Yang, G.; Zhang, W. A Palladium-Catalyzed Enantioselective Addition of Arylboronic Acids to Cyclic Ketimines. *Angew. Chem. Int. Ed.* **2013**, *52* (29), 7540–7544. https://doi.org/10.1002/anie.201302861.

(5) Yang, Q.; Shang, G.; Gao, W.; Deng, J.; Zhang, X. A Highly Enantioselective, Pd–TangPhos-Catalyzed Hydrogenation of N-Tosylimines. *Angew. Chem. Int. Ed.* **2006**, *45* (23), 3832–3835. https://doi.org/10.1002/anie.200600263.

(6) Schrapel, C.; Peters, R. Exogenous-Base-Free Palladacycle-Catalyzed Highly Enantioselective Arylation of Imines with Arylboroxines. *Angew. Chem. Int. Ed.* **2015**, *54* (35), 10289–10293. https://doi.org/10.1002/anie.201501846.

(7) Page, P. C. B.; Bethell, D.; Stocks, P. A.; Heer, J. P.; Graham, A. E.; Vahedi, H.; Healy, M.; Collington, E. W.; Andrews, D. M. Sulfur Oxidation Mediated by Imine Derivatives. *Synlett* **1997**, *12* (12), 1355–1358. https://doi.org/10.1055/s-1997-1051.

(8) Qiu, Z.; Li, Y.; Zhang, Z.; Teng, D. Spiro Indane-Based Phosphine–Oxazoline Ligands for Palladium-Catalyzed Asymmetric Arylation of Cyclic N-Sulfonyl Imines. *Transit. Met. Chem.* **2019**, *44* (7), 649–654. https://doi.org/10.1007/s11243-019-00329-z.

(9) Jiang, T.; Wang, Z.; Xu, M.-H. Rhodium-Catalyzed Asymmetric Arylation of Cyclic N-Sulfonyl Aryl Alkyl Ketimines: Efficient Access to Highly Enantioenriched α-Tertiary Amines. *Org. Lett.* **2015**, *17* (3), 528–531. https://doi.org/10.1021/ol503537w.

(10) Frisch, M. J.; Trucks, G. W.; Schlegel, H. B.; Scuseria, G. E.; Robb, M. A.; Cheeseman, J. R.; Scalmani, G.; Barone, V.; Petersson, G. A.; Nakatsuji, H.; Li, X.; Caricato, M.; Marenich, A. V.; Bloino, J.; Janesko, B. G.; Gomperts, R.; Mennucci, B.; Hratchian, H. P.; Ortiz, J. V.; Izmaylov, A. F.; Sonnenberg, J. L.; Williams; Ding, F.; Lipparini, F.; Egidi, F.; Goings, J.; Peng, B.; Petrone, A.; Henderson, T.; Ranasinghe, D.; Zakrzewski, V. G.; Gao, J.; Rega, N.; Zheng, G.; Liang, W.; Hada, M.; Ehara, M.; Toyota, K.; Fukuda, R.; Hasegawa, J.; Ishida, M.; Nakajima, T.; Honda, Y.; Kitao, O.; Nakai, H.; Vreven, T.; Throssell, K.; Montgomery Jr., J. A.; Peralta, J. E.; Ogliaro, F.; Bearpark, M. J.; Heyd, J. J.; Brothers, E. N.; Kudin, K. N.; Staroverov, V. N.; Keith, T. A.; Kobayashi, R.; Normand, J.; Raghavachari, K.; Rendell, A. P.; Burant, J. C.; Iyengar, S. S.; Tomasi, J.; Cossi, M.; Millam, J. M.; Klene, M.; Adamo, C.; Cammi, R.; Ochterski, J. W.; Martin, R. L.; Morokuma, K.; Farkas, O.; Foresman, J. B.; Fox, D. J. Gaussian 16 Rev. C.01, 2016.

(11) Quan, M.; Yang, G.; Xie, F.; D. Gridnev, I.; Zhang, W. Pd(Ii)-Catalyzed Asymmetric Addition of Arylboronic Acids to Cyclic N -Sulfonyl Ketimine Esters and a DFT Study of Its Mechanism. *Org. Chem. Front.* **2015**, *2* (4), 398–402. https://doi.org/10.1039/C4QO00347K.
